# Supplementary material for: Theoretical morphospace reveals mixed optimisation of the avian wing planform for flight style
Source: Nat Commun. 2026 Mar 31;17:3902. doi: 10.1038/s41467-026-70692-w (PMC13128903; doi:10.1038/s41467-026-70692-w)
Supplement: Supplementary file 1 — Supplementary Information [file 41467_2026_70692_MOESM1_ESM.pdf]

Supplementary Materials:

Theoretical Morphospace Reveals Mixed Optimisation of the Avian Wing Planform for Flight Style

Benton Walters[1]*, Yuming Liu[1], Emily J. Rayfield[1], Philip C. J. Donoghue[1]
Corresponding Author: oz22244@bristol.ac.uk

Containing:

Fig. S1

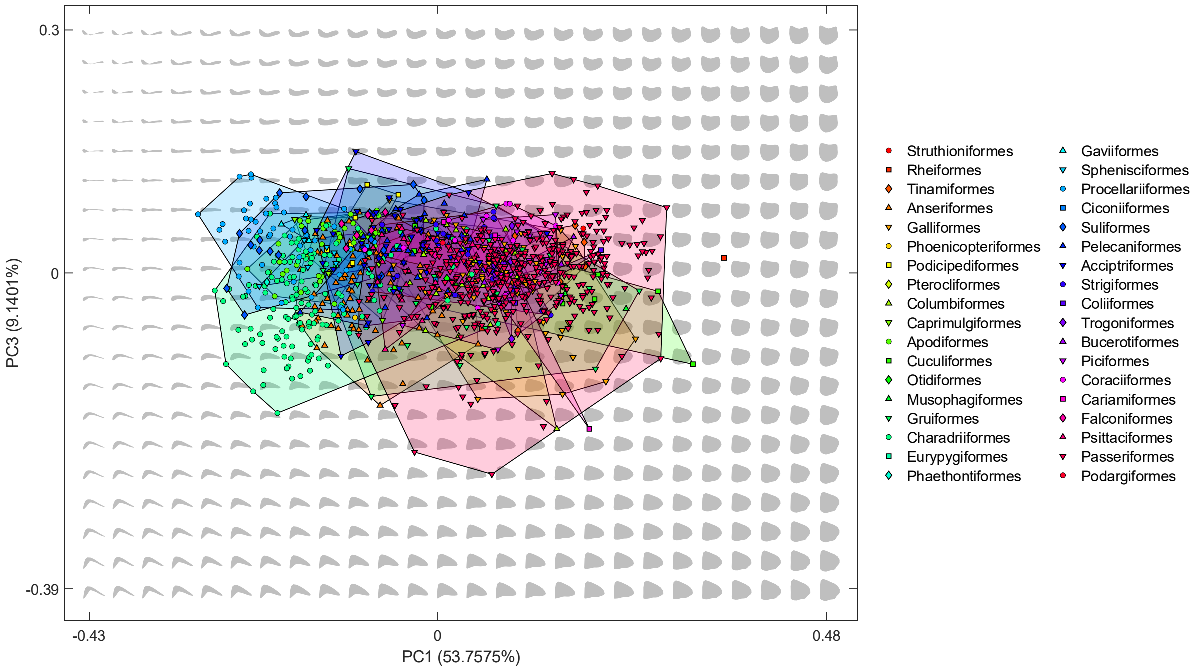

Fig. S1

Theoretical morphospace of PC1 and PC3 with taxa separated by order. Coloured shapes represent positioning of empirical wing shapes separated at order level.
