# Supplementary material for: Theoretical morphospace reveals mixed optimisation of the avian wing planform for flight style
Source: Nat Commun. 2026 Mar 31;17:3902. doi: 10.1038/s41467-026-70692-w (PMC13128903; doi:10.1038/s41467-026-70692-w)
Supplement: Supplementary file 2 — Descriptions of Additional Supplementary Files [file 41467_2026_70692_MOESM2_ESM.pdf]

Descriptions of Additional Supplementary Files

Supplementary Data 1

Folder containing all taxa set files for the main analysis, divided into:

'Taxa Set' – List of taxa used in the analysis and their citations

'Bird_TDS2' and 'BirdCladeNames' - the main files used to load the dataset

All other documents pertain to specific flight style groupings used for generating the convex hulls in figs. 5 and 7b.

Supplementary Data 2

The original tree used in phylomorphosace and ancestral shape analyses along with all dependency and output files for phylomorphospace analysis in R and transfer back to MATLAB for plotting Fig. 7a.

Supplementary Data 3

Additional taxa and tree files for reconstruction of the Wang and Clarke and Baumgart et al. analyses, used to test phylogenetic signal.

Supplementary Data 4

.tif outline images of all taxa used in the analysis.

Supplementary Data 5

Dataset from Rayner (1988), reconstructed and migrated to excel from original files provided to the authors

Supplementary Code 1

MATLAB and R code files for running analyses and producing figs 2-7. Additionally, for the second moment of area landscape, the 'shapespace.m' and 'TheoShapeN.m' were modified from the original 'theofun' code (Deakin, W. J., Rayfield, E. J., & Donoghue, P. C. theofun (Version 0.0.1) [Computer software]. https://github.com/Bristol-Palaeobiology/theofun) to produce the non-dimentioanlised second moment of area explained in methods. To generate non-dimentionalised 2MA replace these two files in your operating folder with the replacements provided (designated with '(2MA)') and rename to match the original files. Be sure to replace these files again with the default theofun files before completing the remaining analyses. For dimentionalised second moment of area, the published base code will work without alteration.
