# Supplementary figures and images for: Theoretical morphospace reveals mixed optimisation of the avian wing planform for flight style (part 2 of 2)
Source: Nat Commun. 2026 Mar 31;17:3902. doi: 10.1038/s41467-026-70692-w (PMC13128903; doi:10.1038/s41467-026-70692-w)

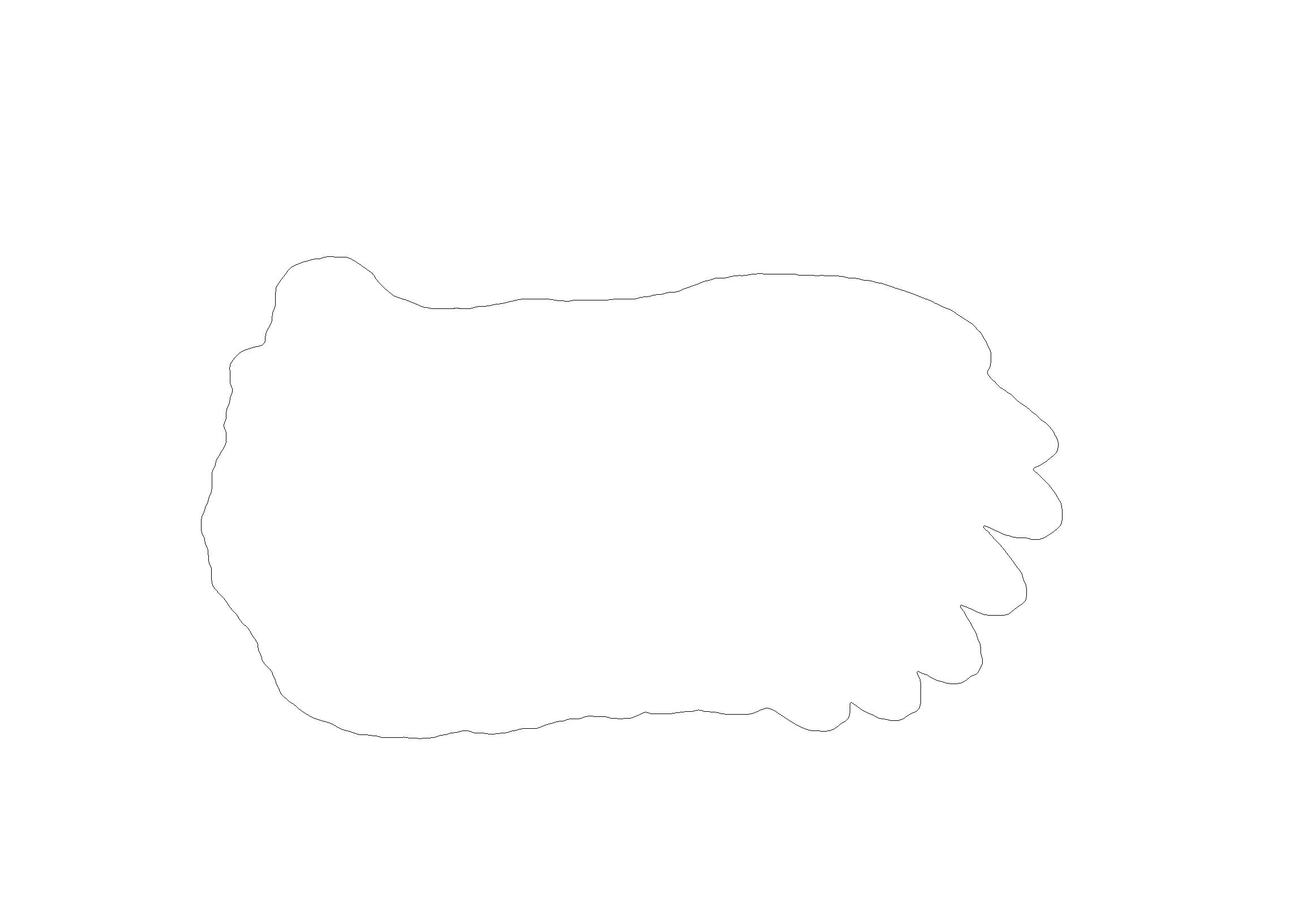

Supplement: Supplementary file 6 — Supplementary Data 4 [file 41467_2026_70692_MOESM6_ESM.zip › Supplementary Data 4/Bonasa_bonasia.tif]

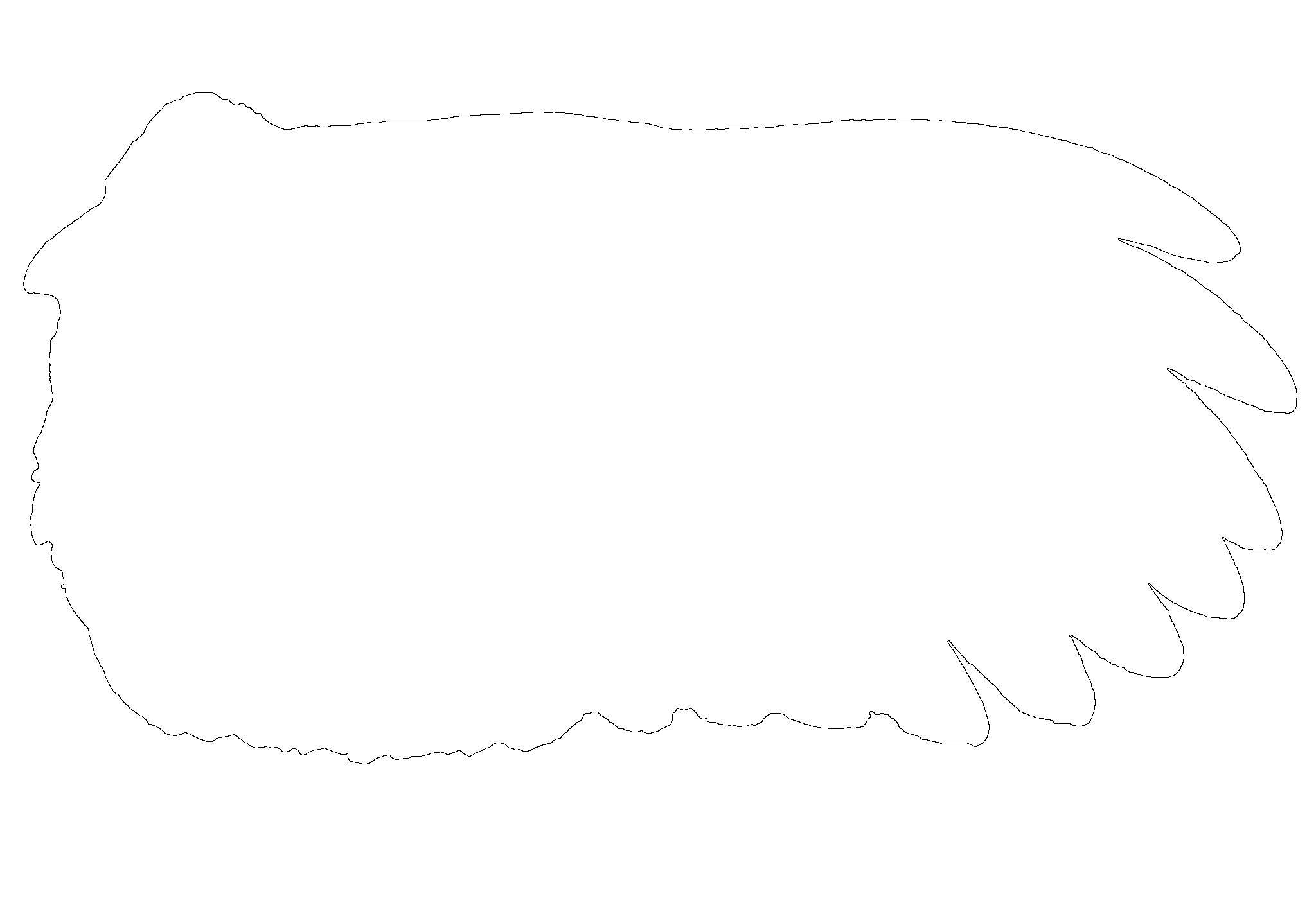

Supplement: Supplementary file 6 — Supplementary Data 4 [file 41467_2026_70692_MOESM6_ESM.zip › Supplementary Data 4/Bonasa_umbellus.tif]

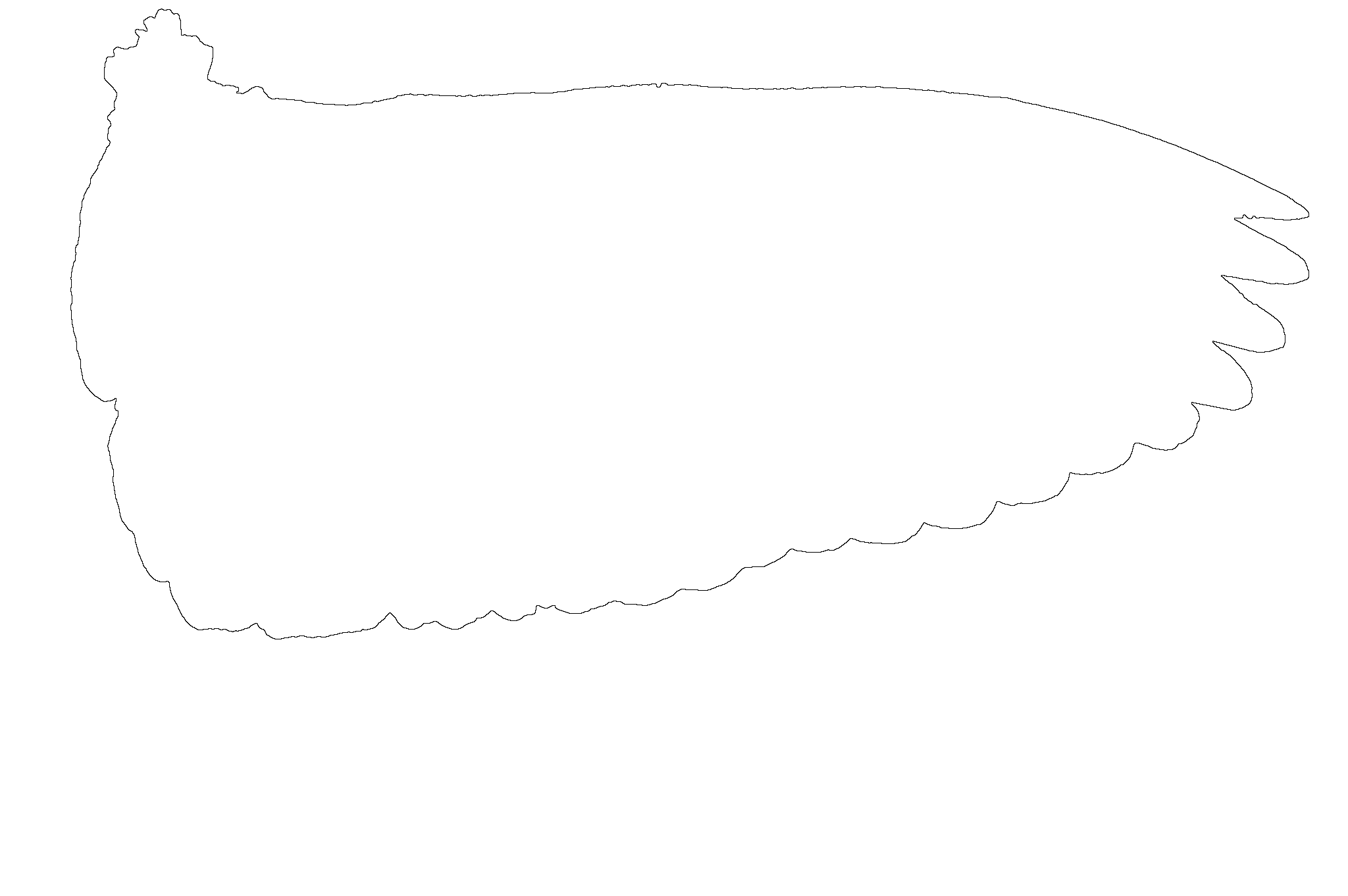

Supplement: Supplementary file 6 — Supplementary Data 4 [file 41467_2026_70692_MOESM6_ESM.zip › Supplementary Data 4/Botaurus_lentiginosus.tif]

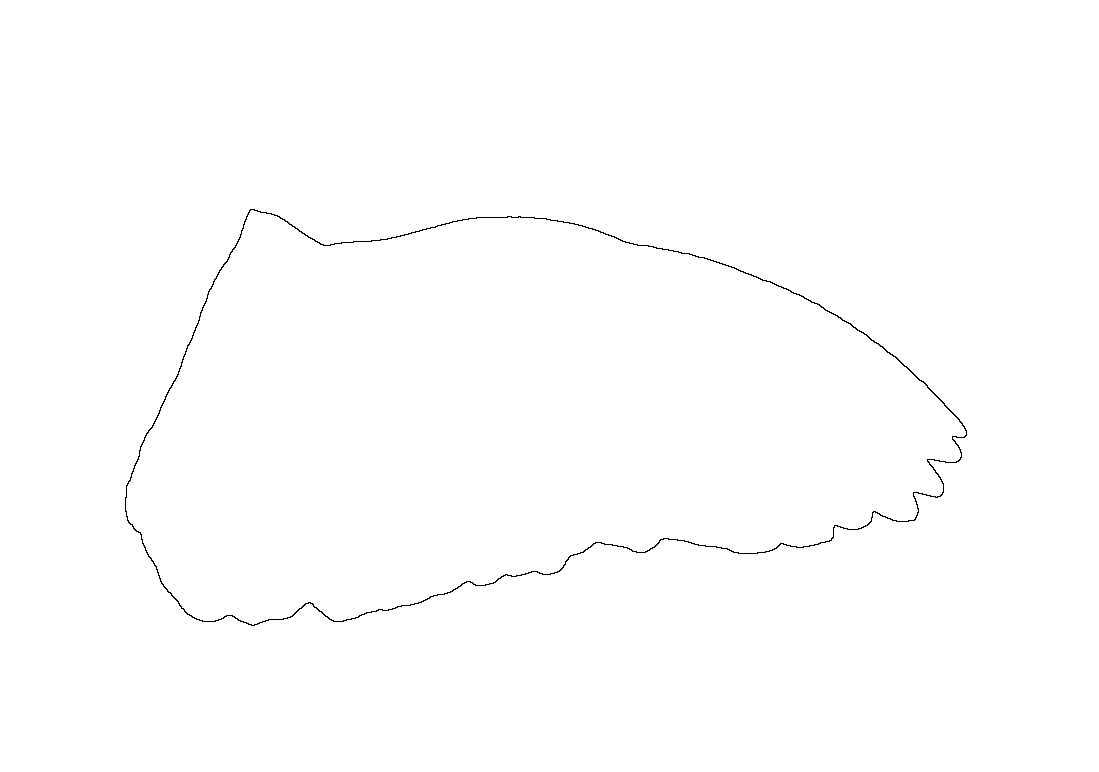

Supplement: Supplementary file 6 — Supplementary Data 4 [file 41467_2026_70692_MOESM6_ESM.zip › Supplementary Data 4/Botaurus_stellaris.tif]

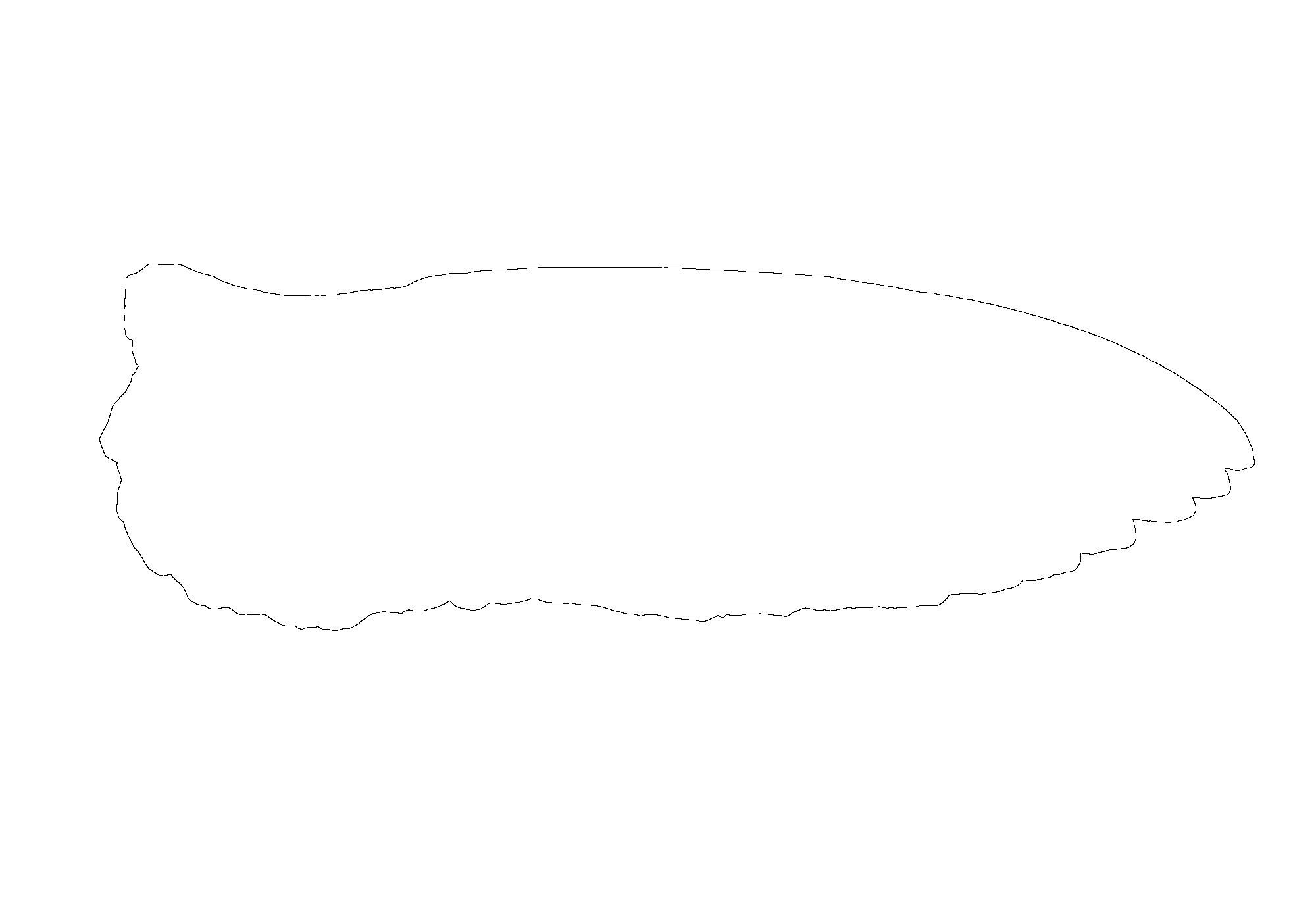

Supplement: Supplementary file 6 — Supplementary Data 4 [file 41467_2026_70692_MOESM6_ESM.zip › Supplementary Data 4/Brachyramphus_marmoratus.tif]

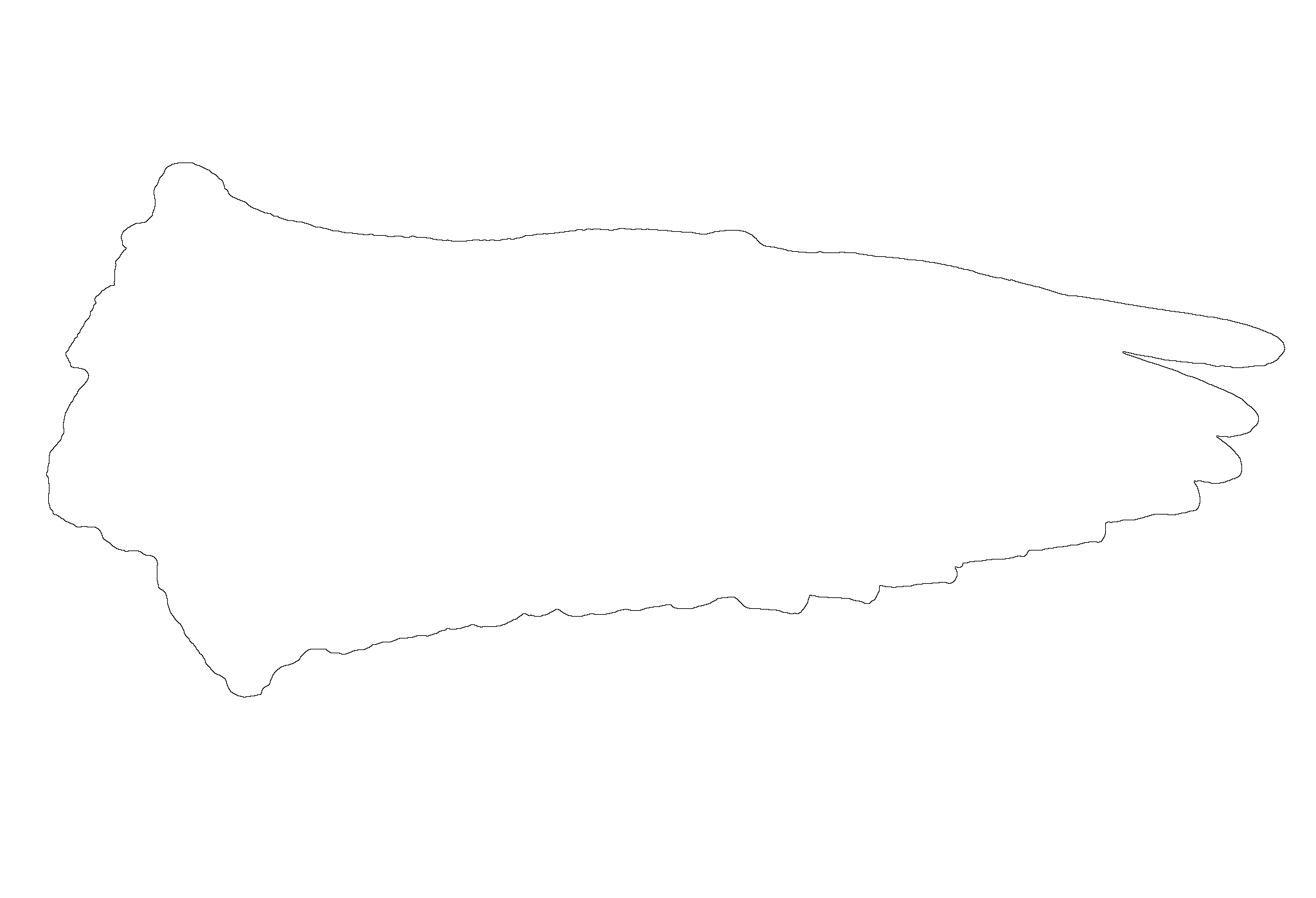

Supplement: Supplementary file 6 — Supplementary Data 4 [file 41467_2026_70692_MOESM6_ESM.zip › Supplementary Data 4/Branta_bernicla.tif]

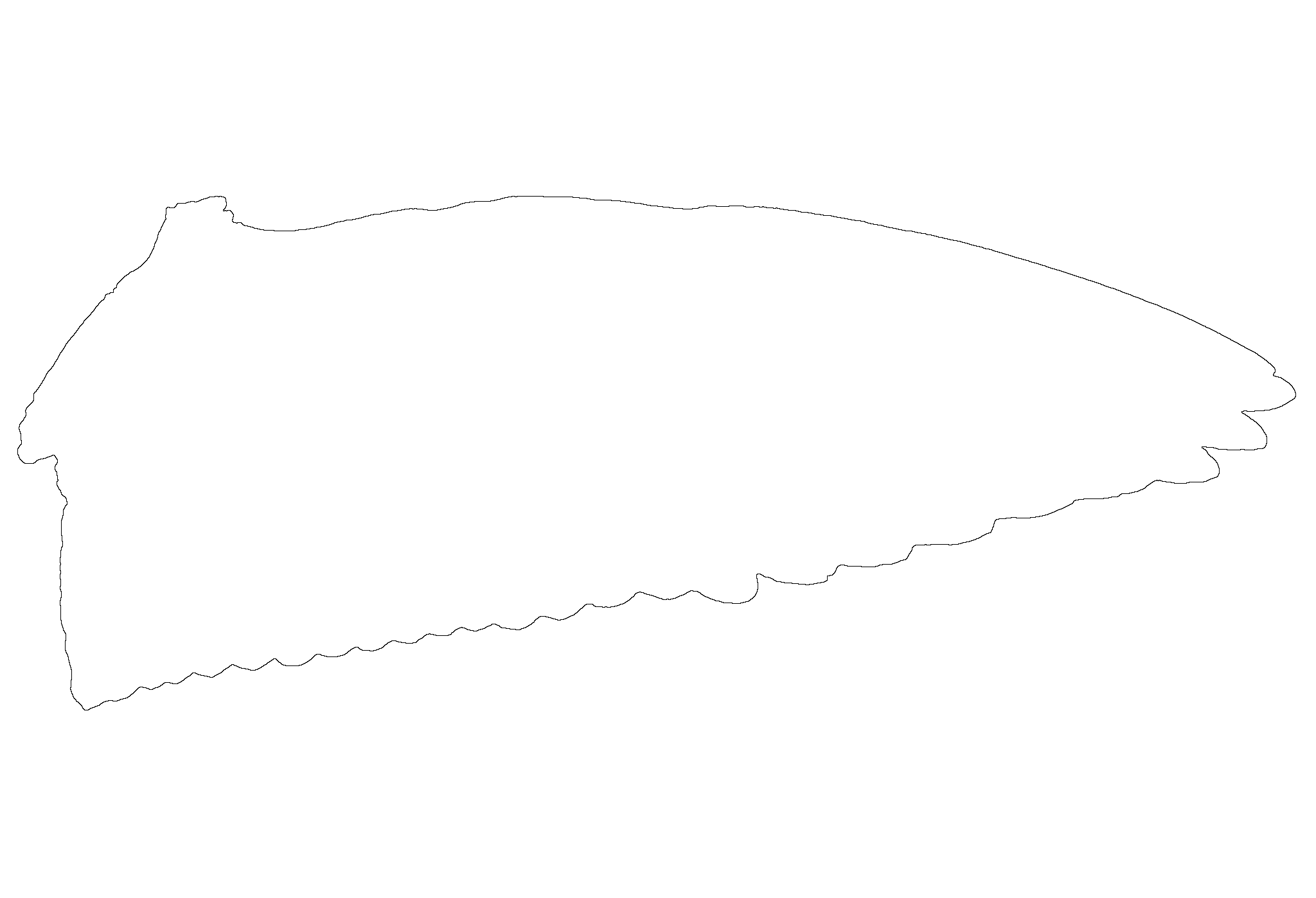

Supplement: Supplementary file 6 — Supplementary Data 4 [file 41467_2026_70692_MOESM6_ESM.zip › Supplementary Data 4/Branta_canadensis.tif]

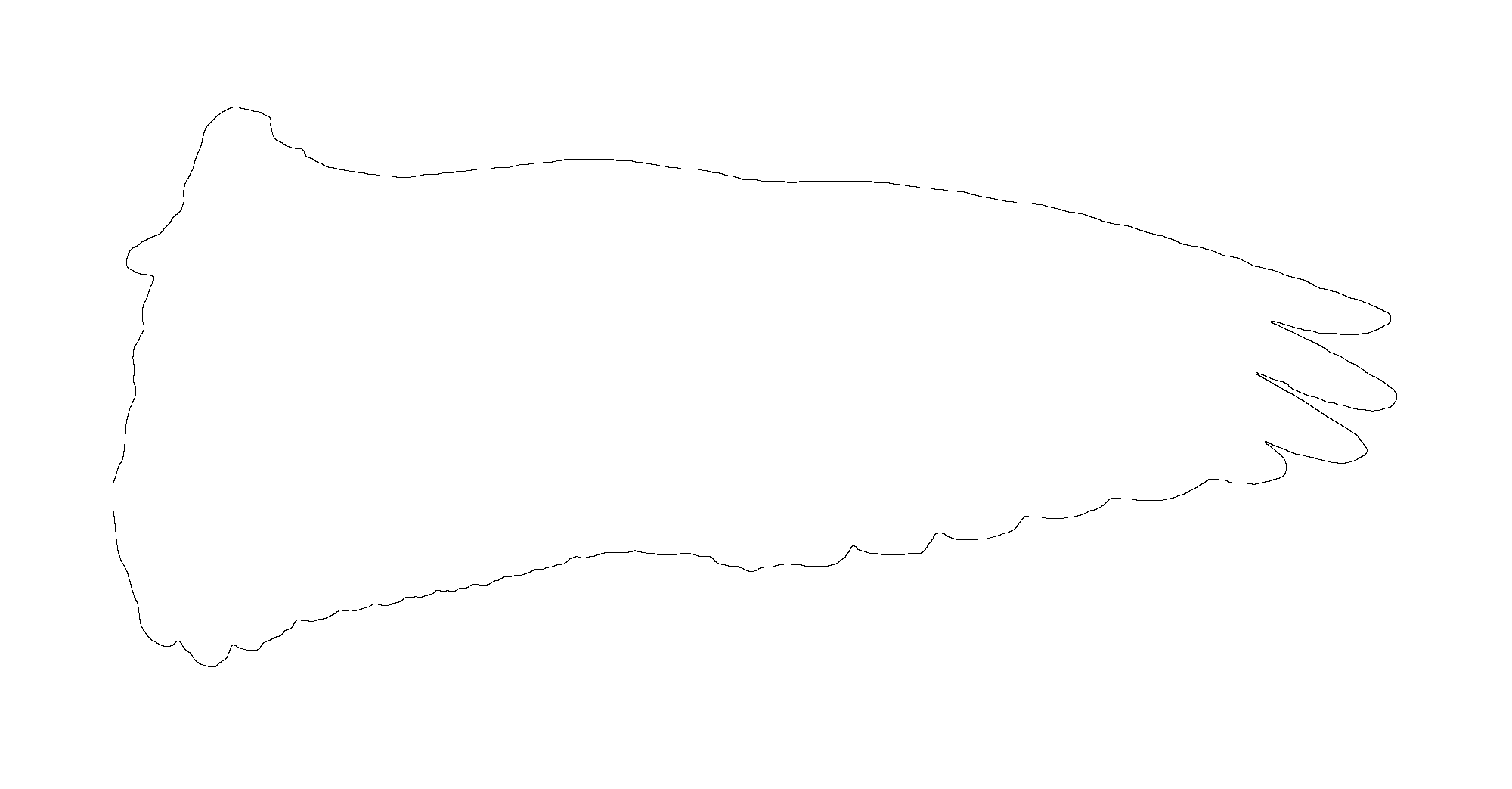

Supplement: Supplementary file 6 — Supplementary Data 4 [file 41467_2026_70692_MOESM6_ESM.zip › Supplementary Data 4/Branta_hutchinsii.tif]

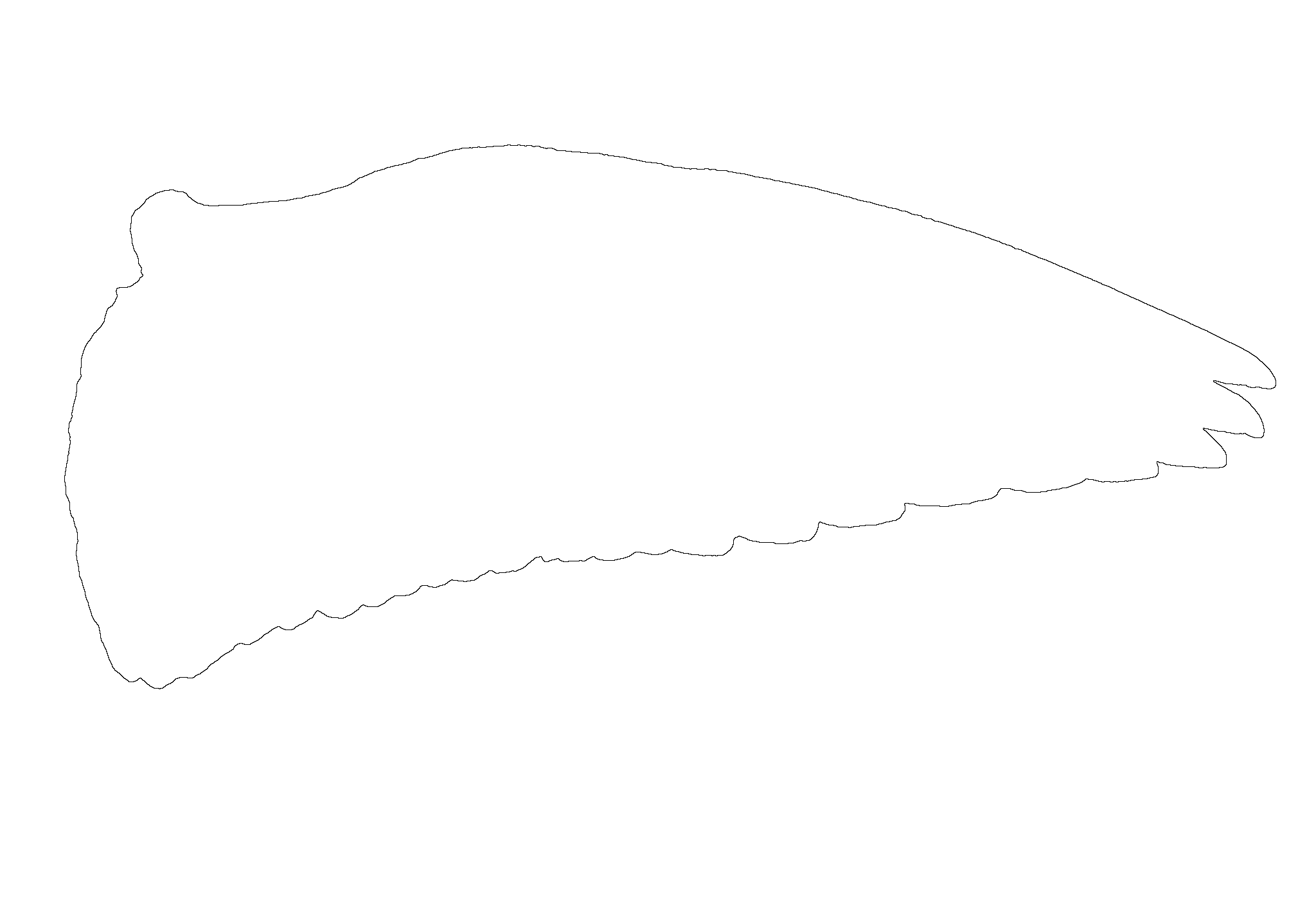

Supplement: Supplementary file 6 — Supplementary Data 4 [file 41467_2026_70692_MOESM6_ESM.zip › Supplementary Data 4/Branta_leucopsis.tif]

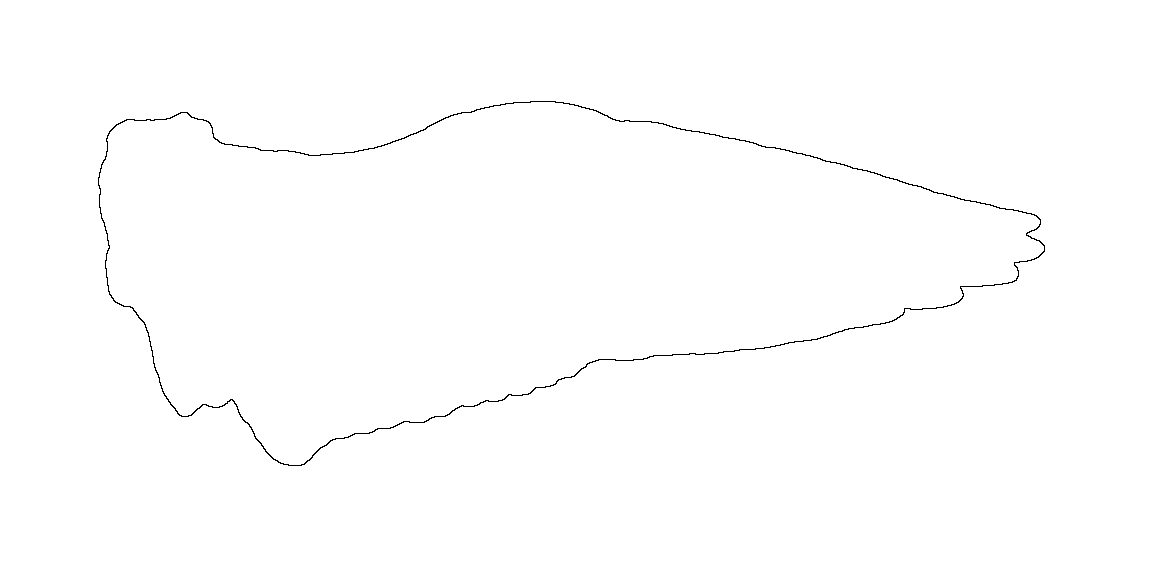

Supplement: Supplementary file 6 — Supplementary Data 4 [file 41467_2026_70692_MOESM6_ESM.zip › Supplementary Data 4/Branta_ruficollis.tif]

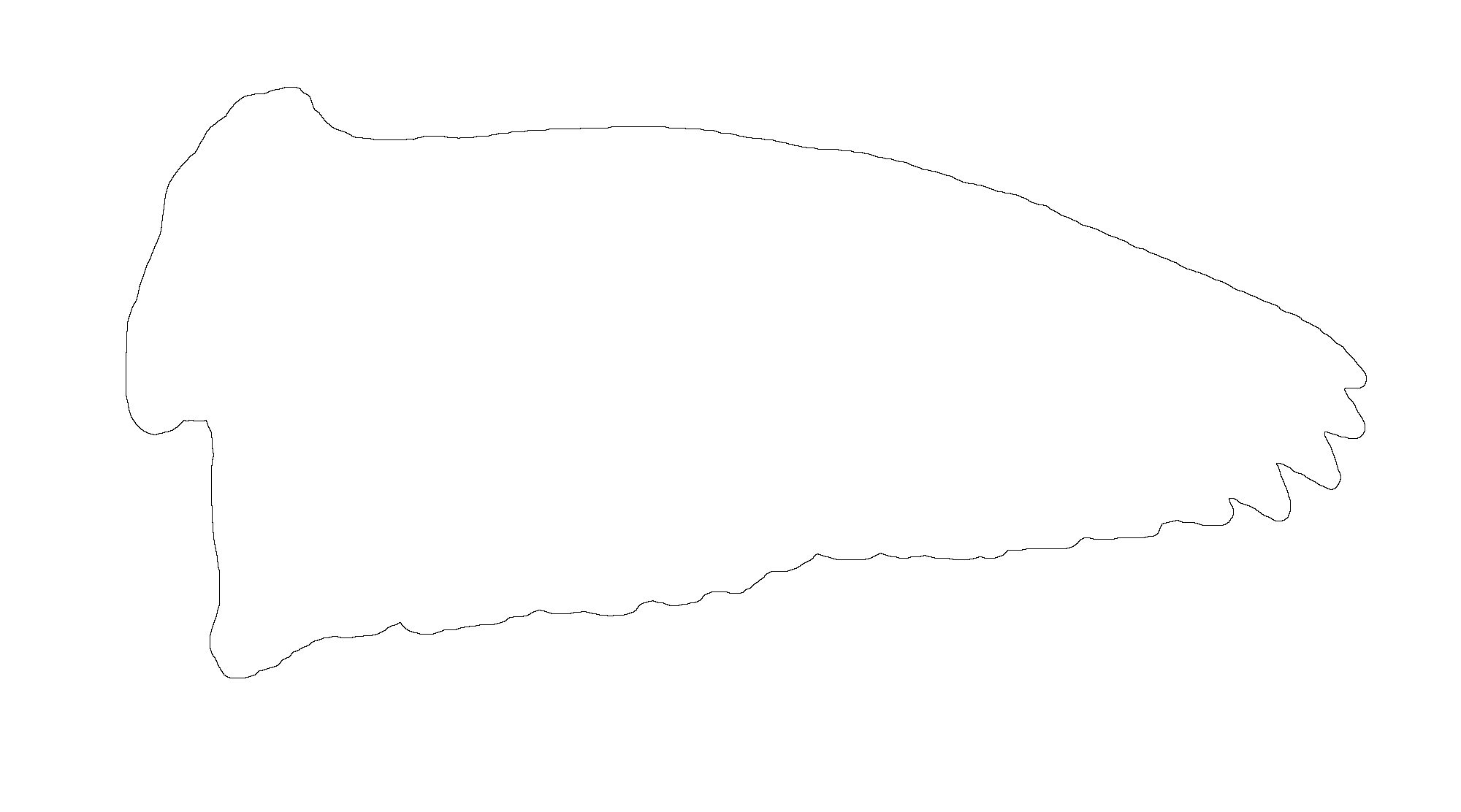

Supplement: Supplementary file 6 — Supplementary Data 4 [file 41467_2026_70692_MOESM6_ESM.zip › Supplementary Data 4/Branta_Sandvicensis.tif]

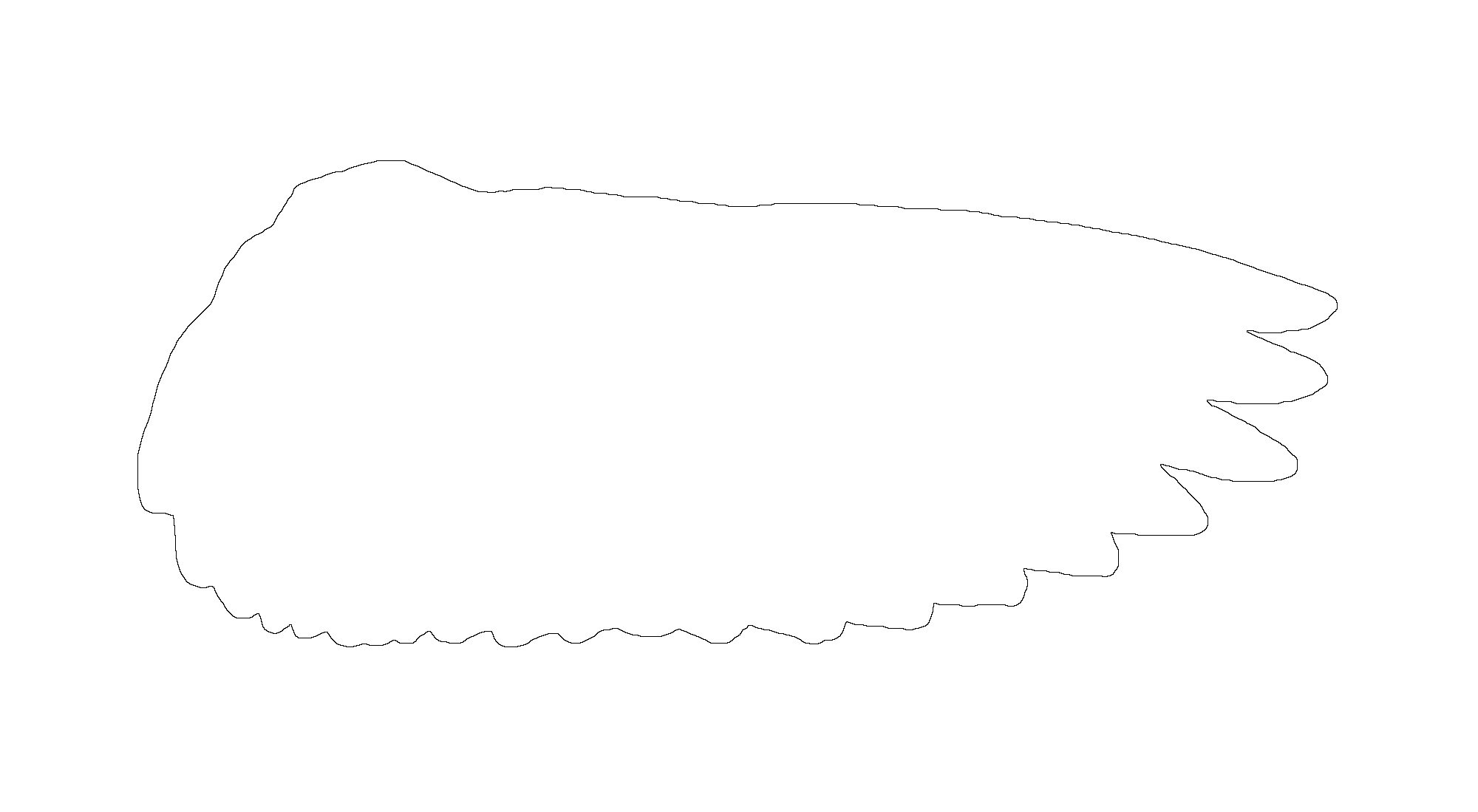

Supplement: Supplementary file 6 — Supplementary Data 4 [file 41467_2026_70692_MOESM6_ESM.zip › Supplementary Data 4/Brotogeris_jugularis.tif]

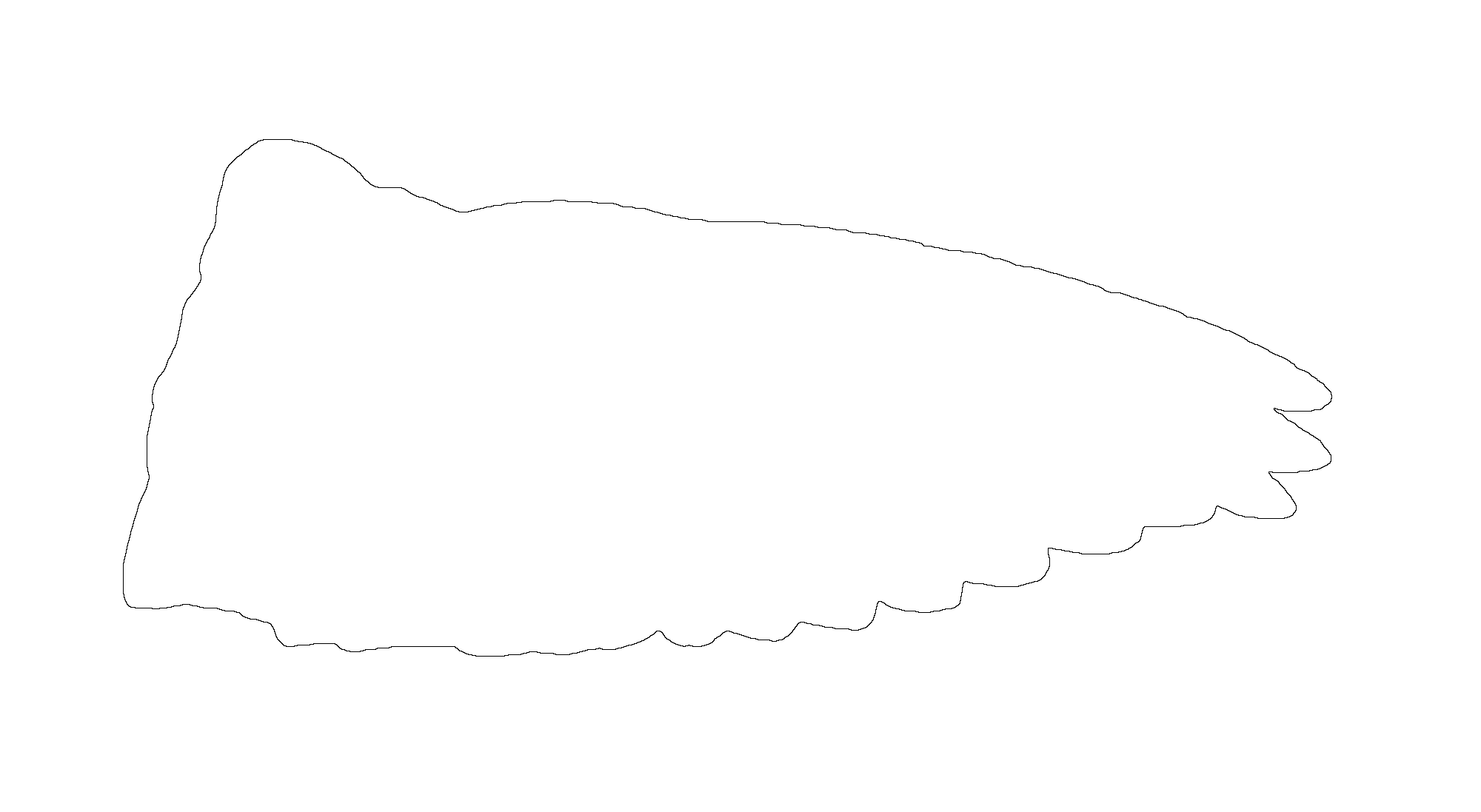

Supplement: Supplementary file 6 — Supplementary Data 4 [file 41467_2026_70692_MOESM6_ESM.zip › Supplementary Data 4/Brotogeris_versicolurus.tif]

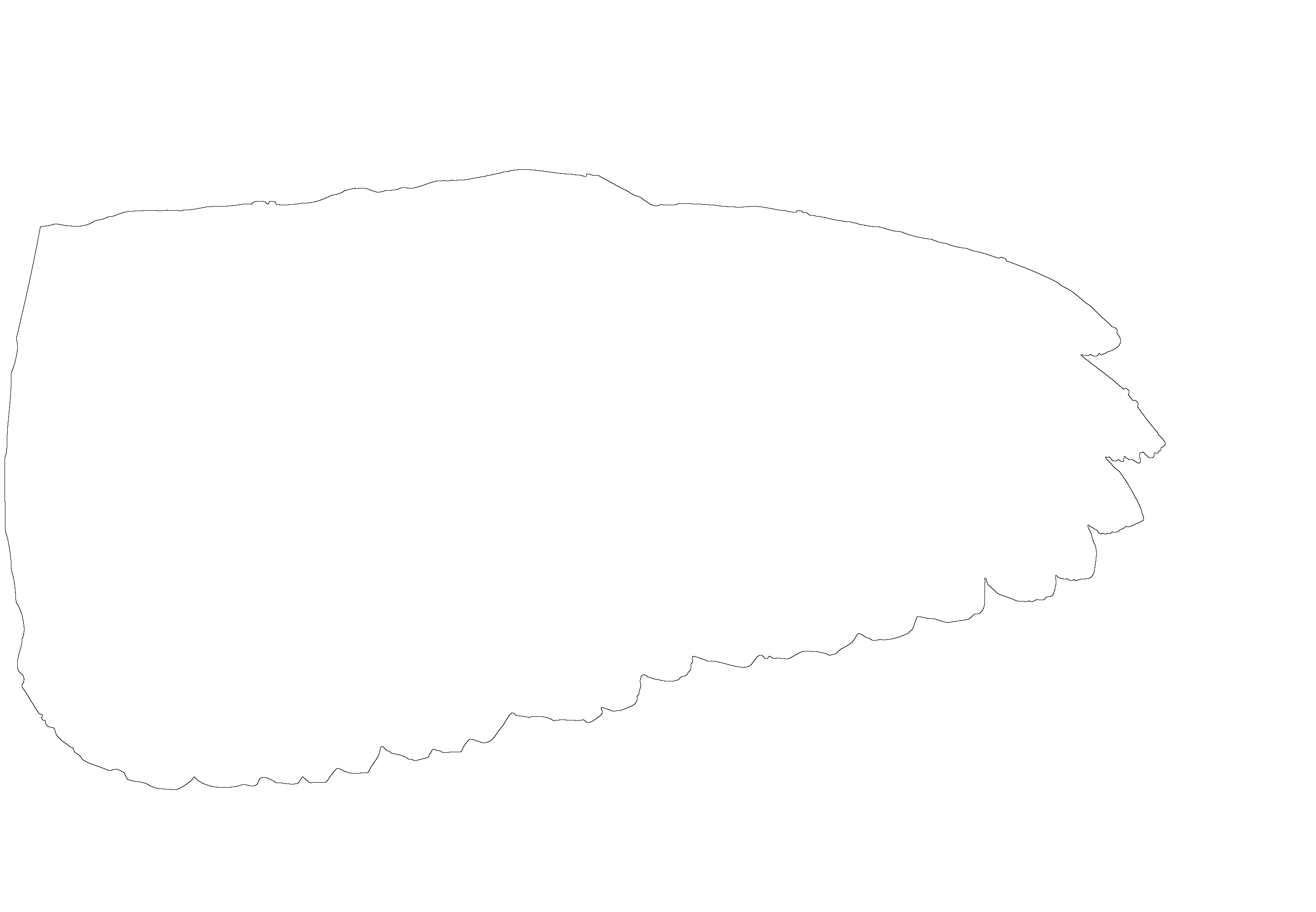

Supplement: Supplementary file 6 — Supplementary Data 4 [file 41467_2026_70692_MOESM6_ESM.zip › Supplementary Data 4/Bubo_bubo.tif]

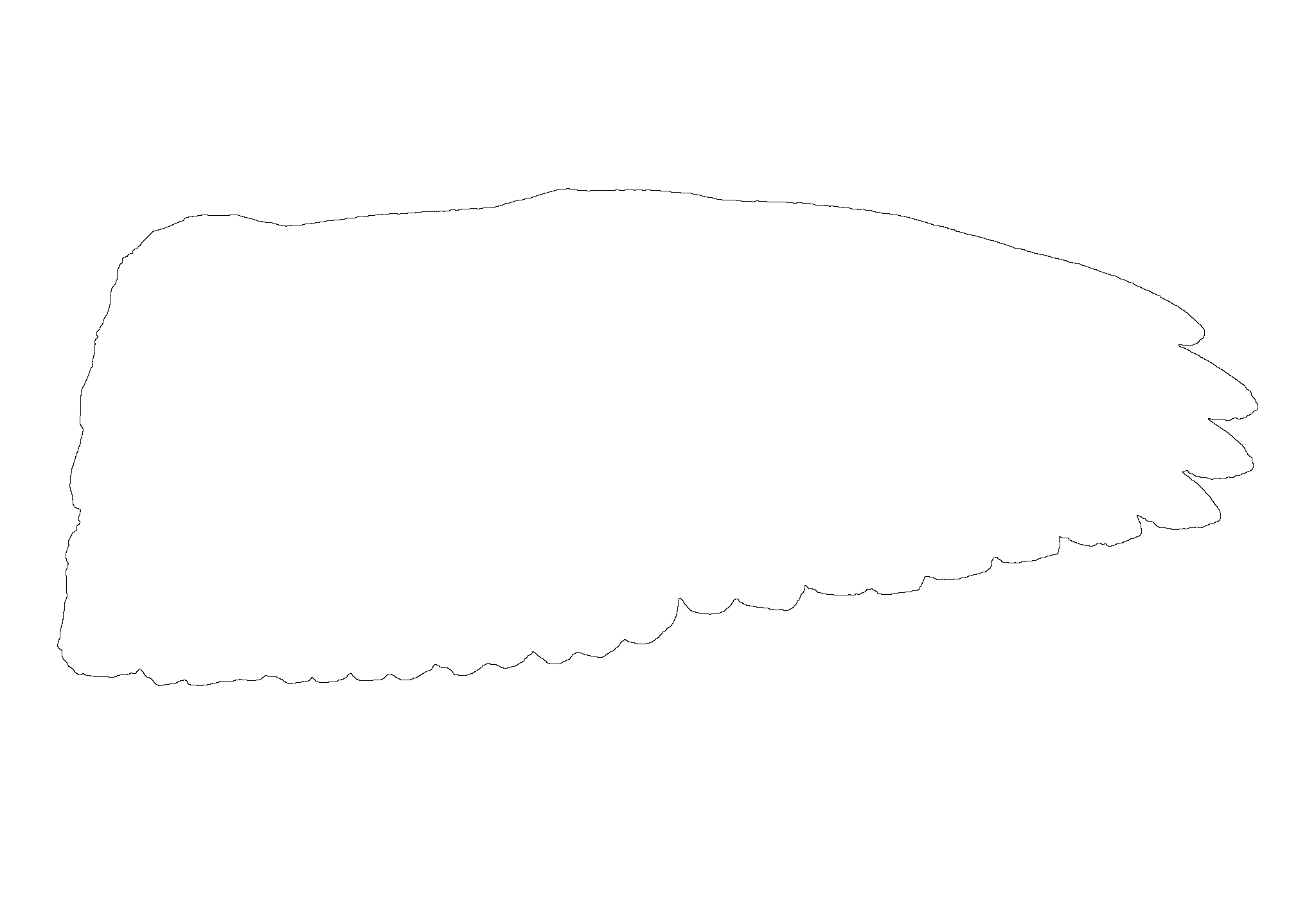

Supplement: Supplementary file 6 — Supplementary Data 4 [file 41467_2026_70692_MOESM6_ESM.zip › Supplementary Data 4/Bubo_scandiaca.tif]

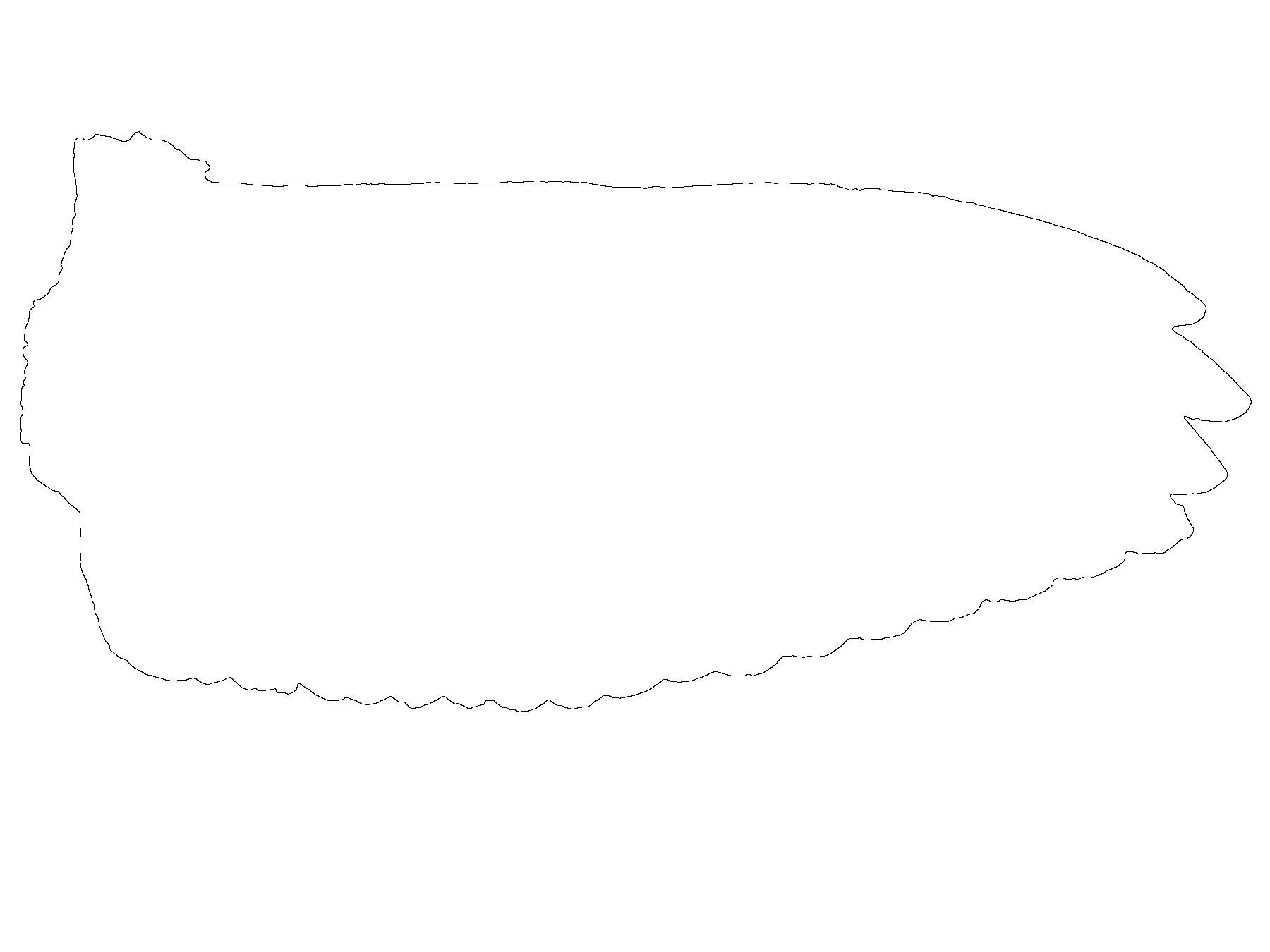

Supplement: Supplementary file 6 — Supplementary Data 4 [file 41467_2026_70692_MOESM6_ESM.zip › Supplementary Data 4/Bubo_virginianus.tif]

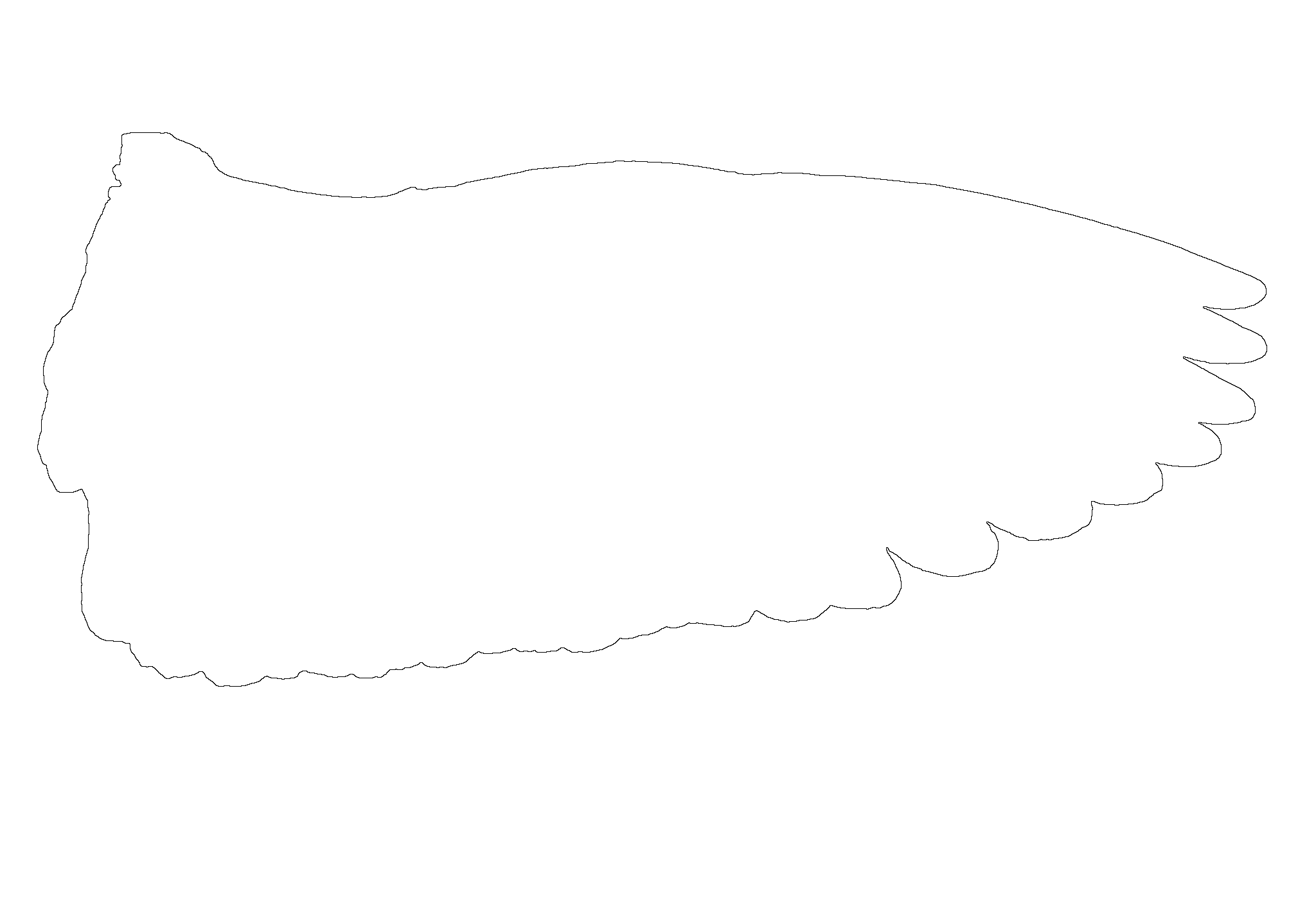

Supplement: Supplementary file 6 — Supplementary Data 4 [file 41467_2026_70692_MOESM6_ESM.zip › Supplementary Data 4/Bubulcus_ibis.tif]

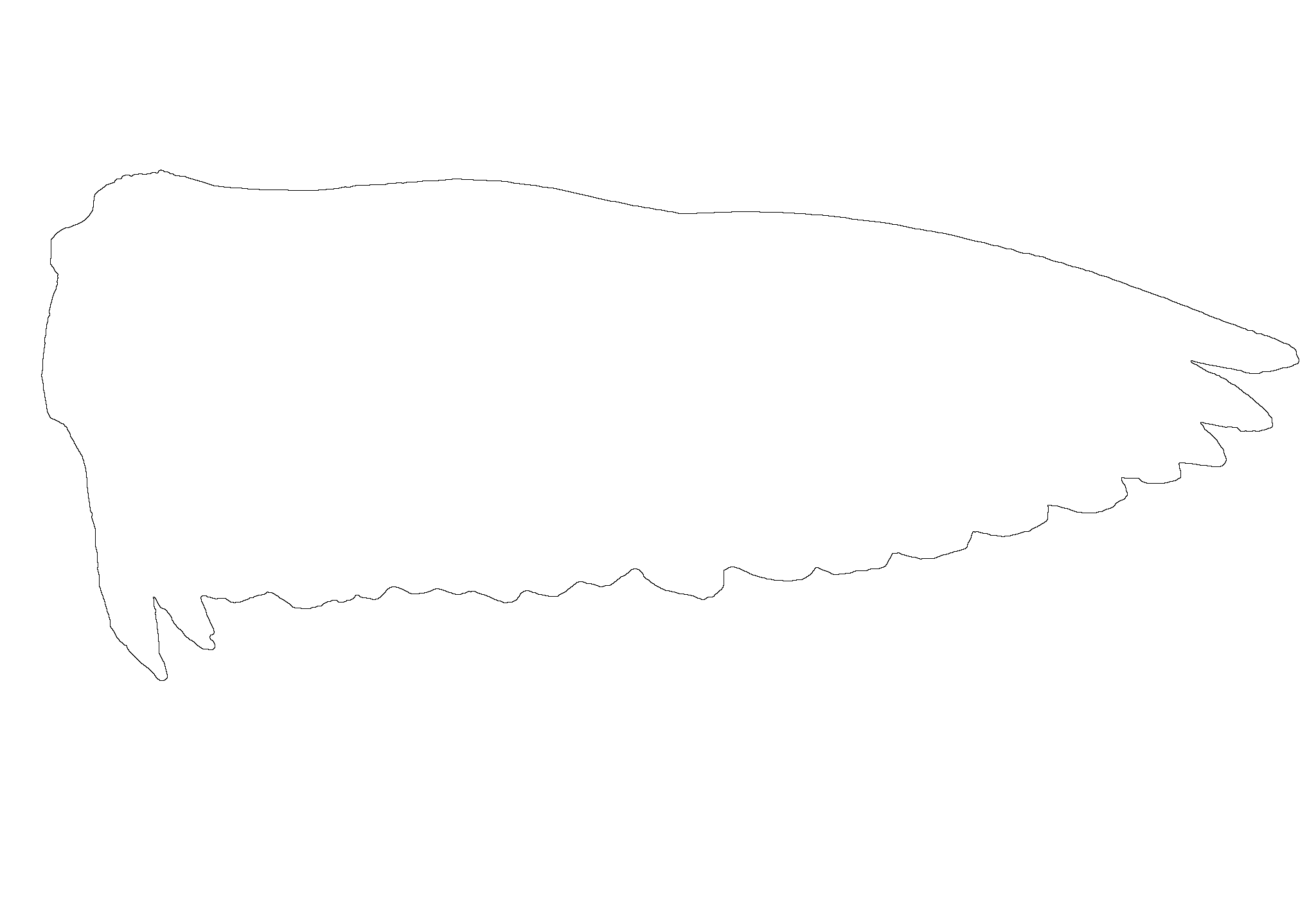

Supplement: Supplementary file 6 — Supplementary Data 4 [file 41467_2026_70692_MOESM6_ESM.zip › Supplementary Data 4/Bucephala_albeola.tif]

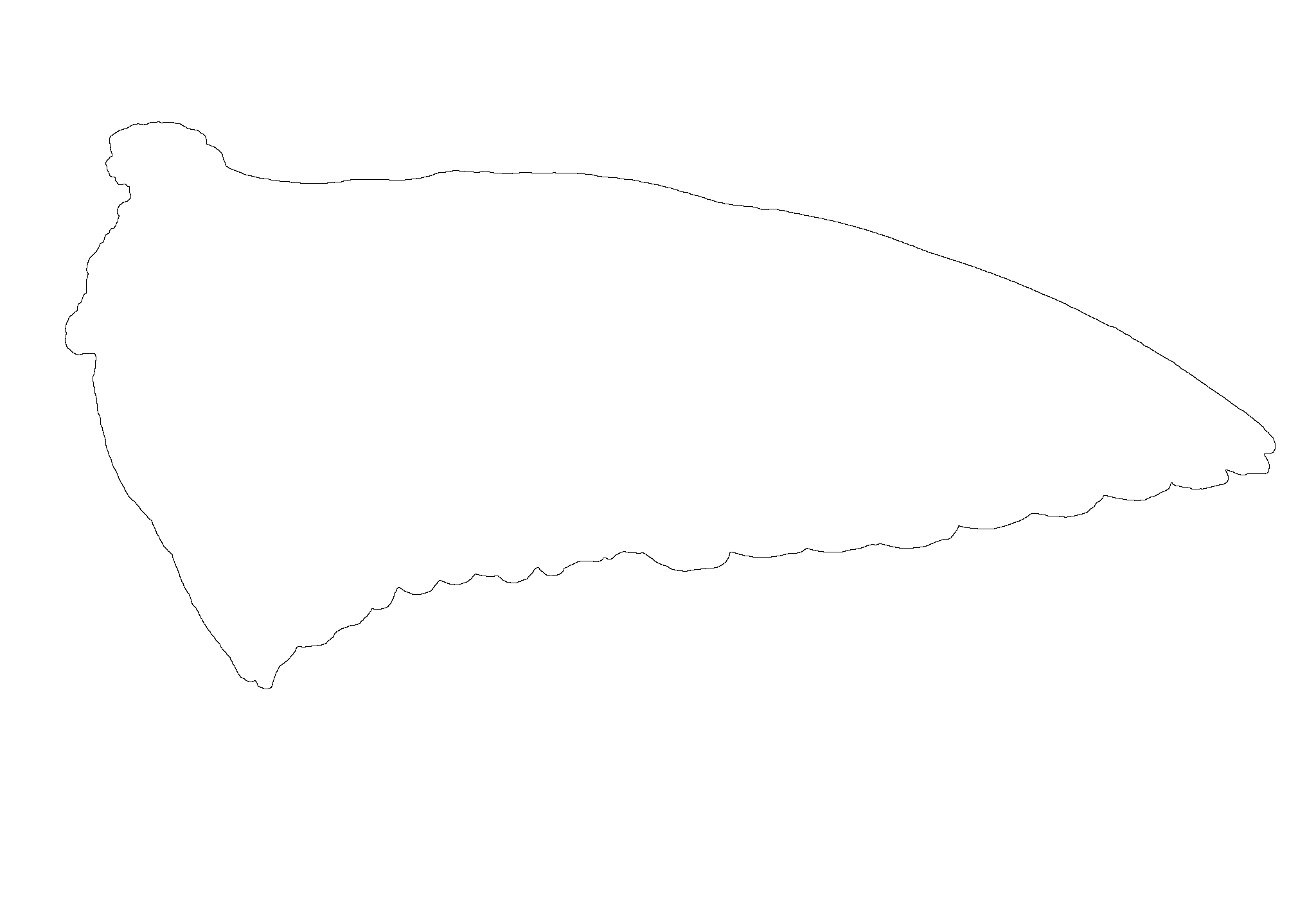

Supplement: Supplementary file 6 — Supplementary Data 4 [file 41467_2026_70692_MOESM6_ESM.zip › Supplementary Data 4/Bucephala_clangula.tif]

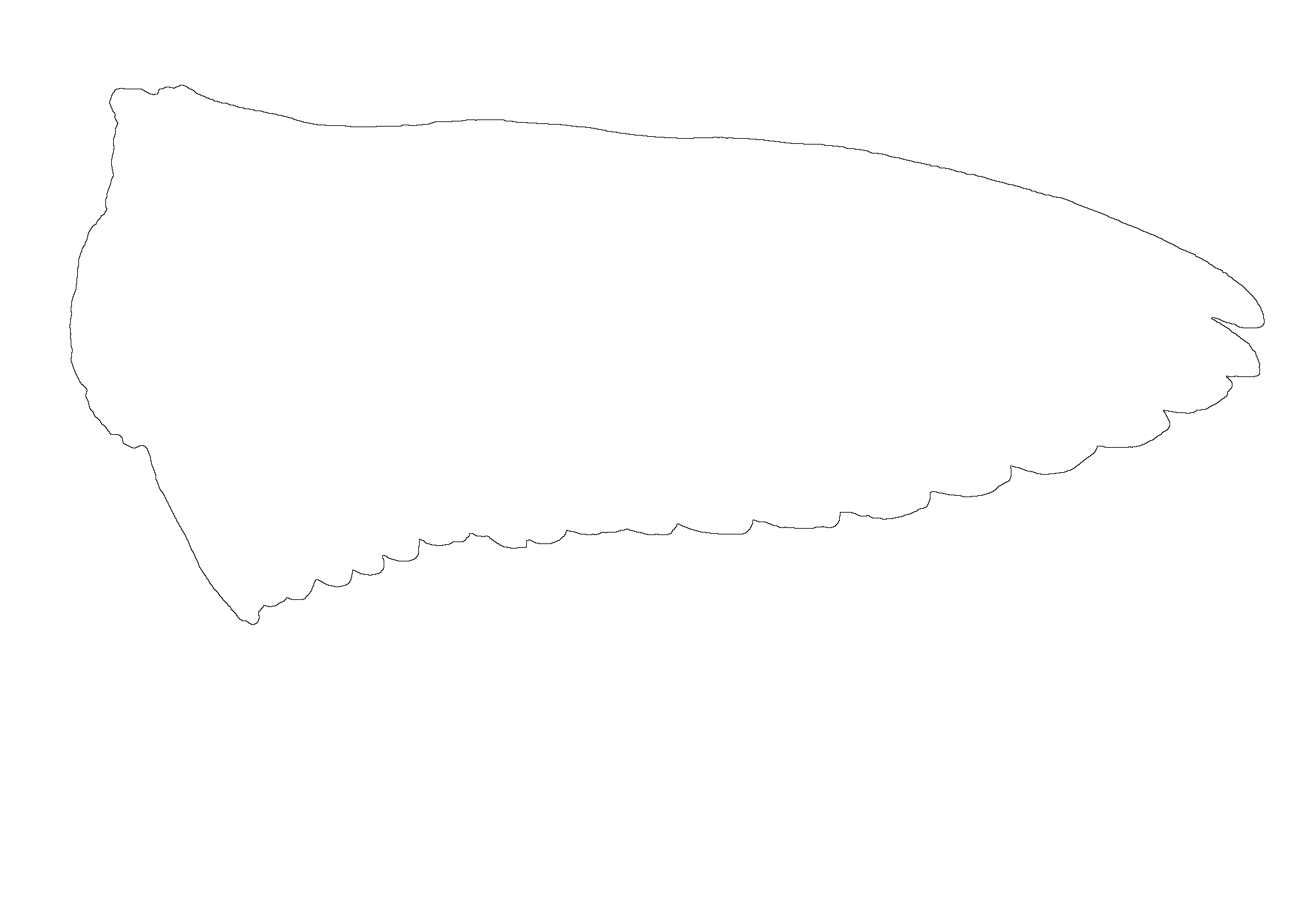

Supplement: Supplementary file 6 — Supplementary Data 4 [file 41467_2026_70692_MOESM6_ESM.zip › Supplementary Data 4/Bucephala_islandica.tif]

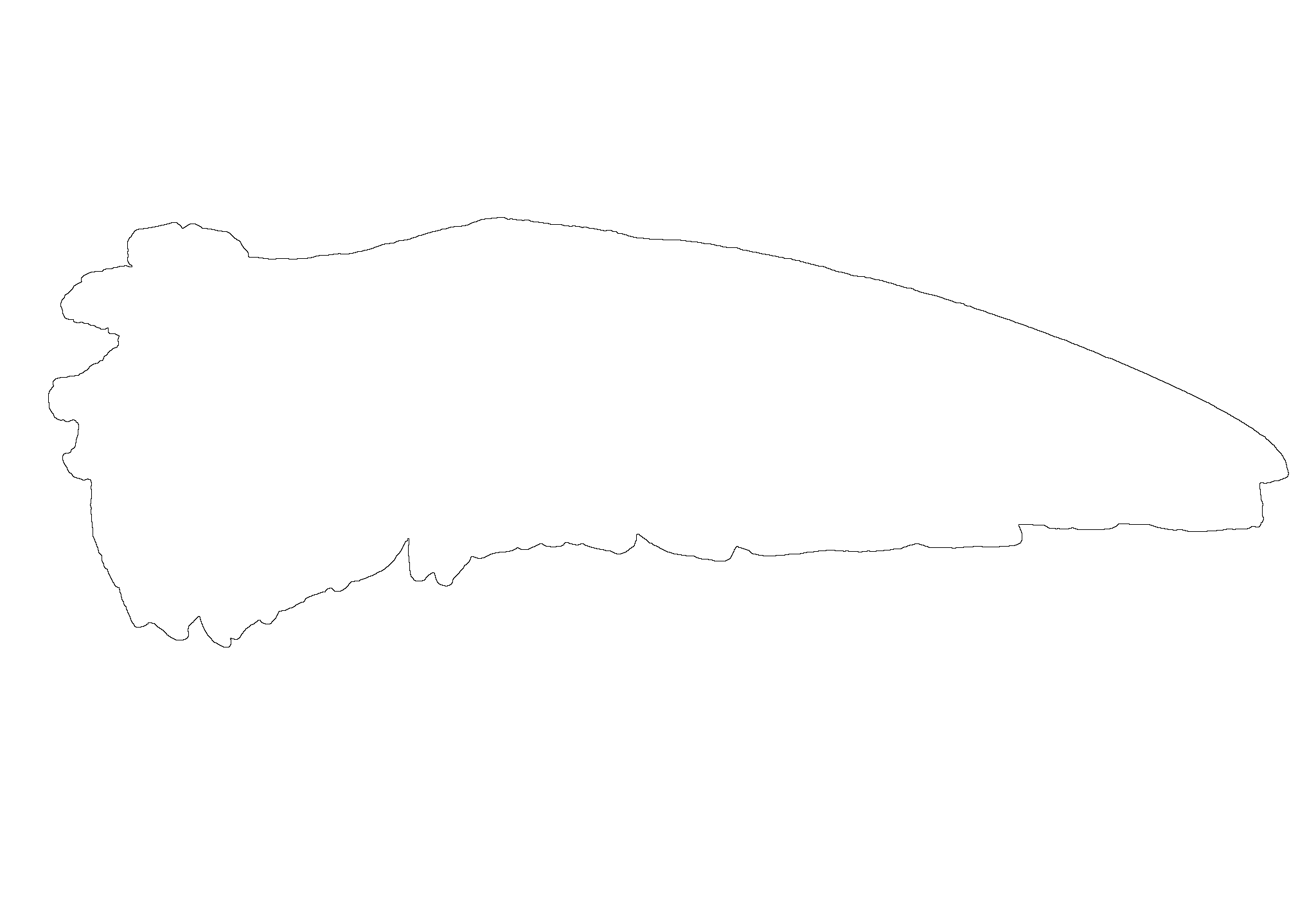

Supplement: Supplementary file 6 — Supplementary Data 4 [file 41467_2026_70692_MOESM6_ESM.zip › Supplementary Data 4/Bulweria_bulwerii.tif]

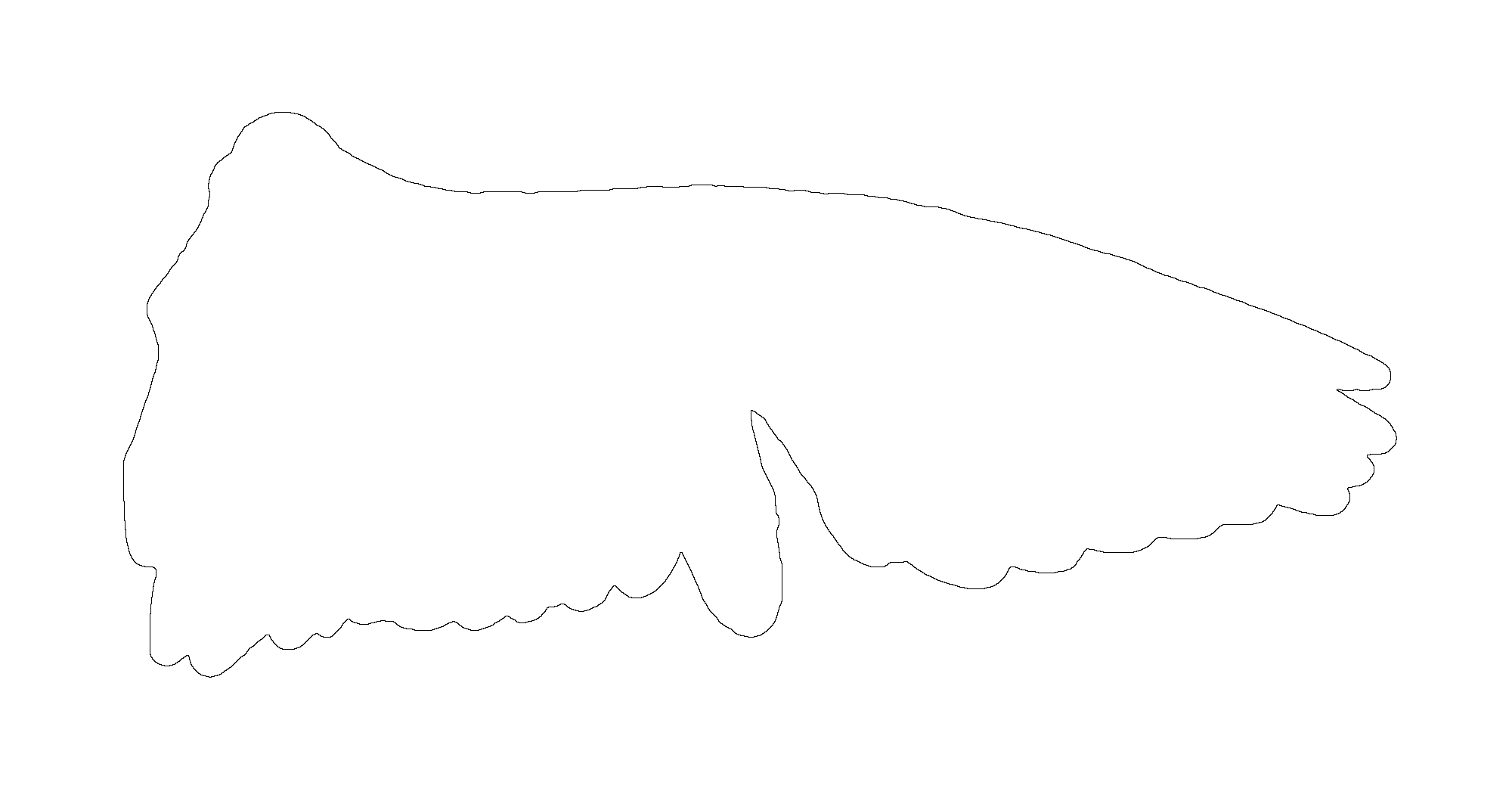

Supplement: Supplementary file 6 — Supplementary Data 4 [file 41467_2026_70692_MOESM6_ESM.zip › Supplementary Data 4/Burhinus_bistriatus.tif]

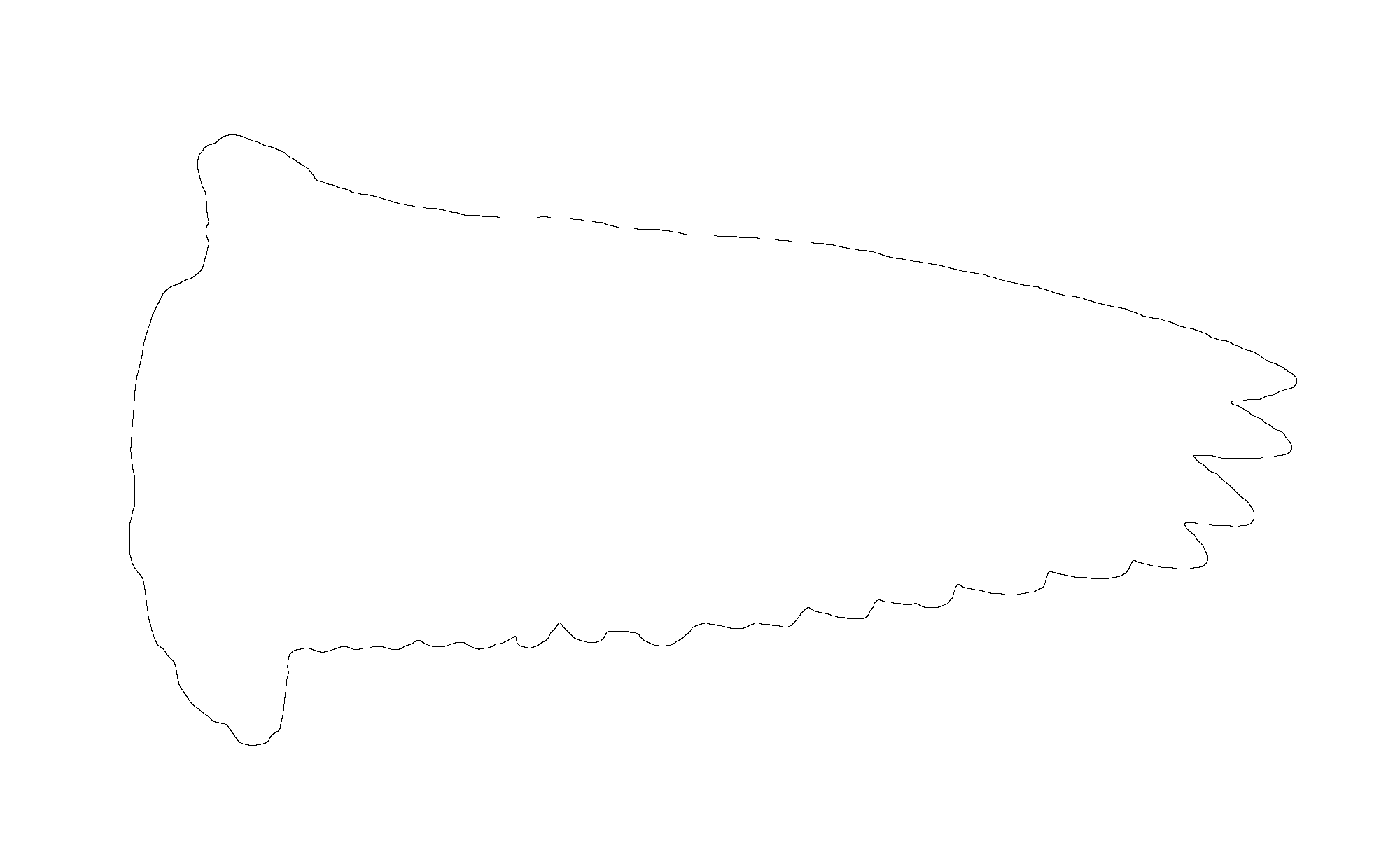

Supplement: Supplementary file 6 — Supplementary Data 4 [file 41467_2026_70692_MOESM6_ESM.zip › Supplementary Data 4/Burhinus_capensis.tif]

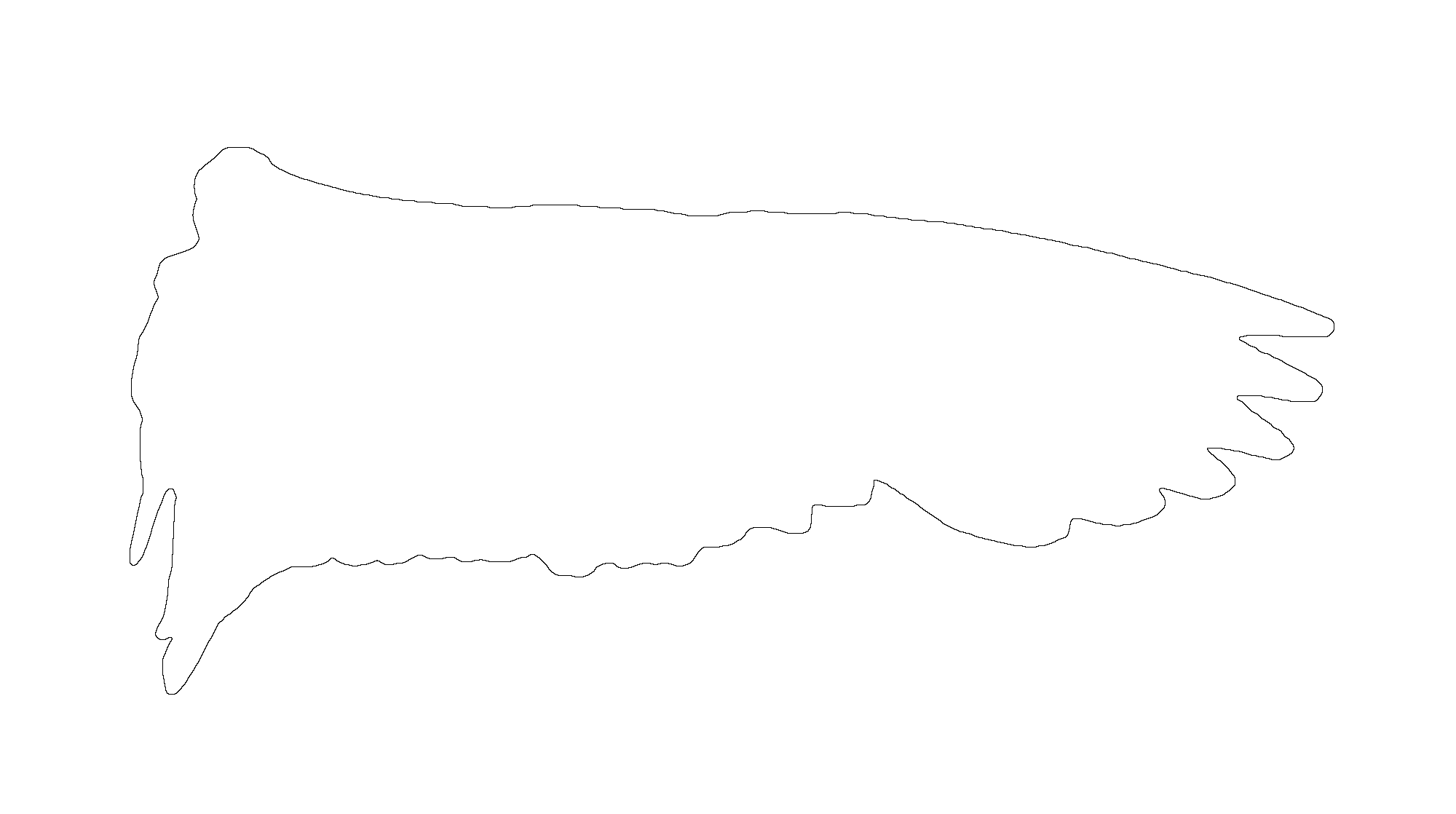

Supplement: Supplementary file 6 — Supplementary Data 4 [file 41467_2026_70692_MOESM6_ESM.zip › Supplementary Data 4/Burhinus_oedicnemus.tif]

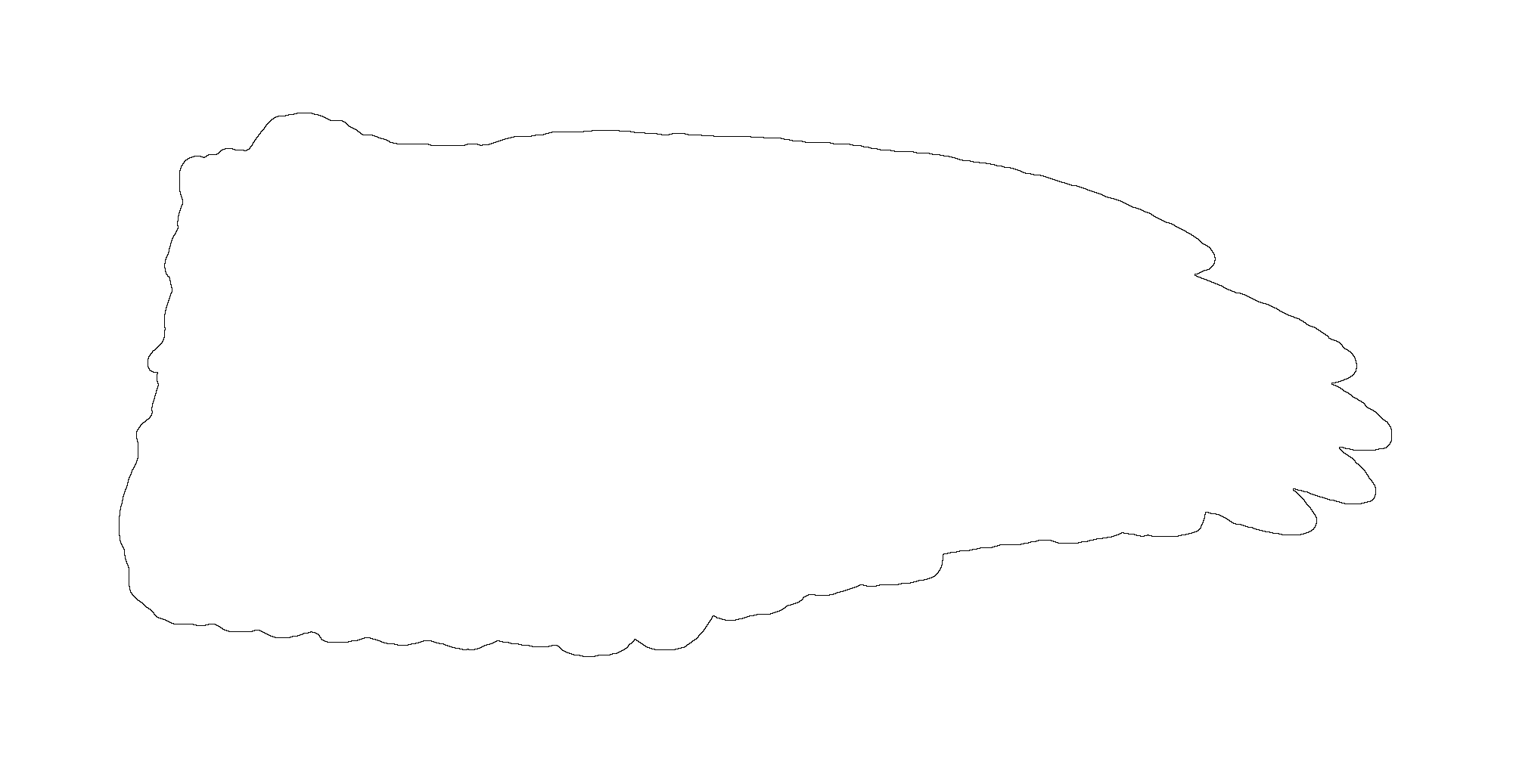

Supplement: Supplementary file 6 — Supplementary Data 4 [file 41467_2026_70692_MOESM6_ESM.zip › Supplementary Data 4/Buteo_buteo.tif]

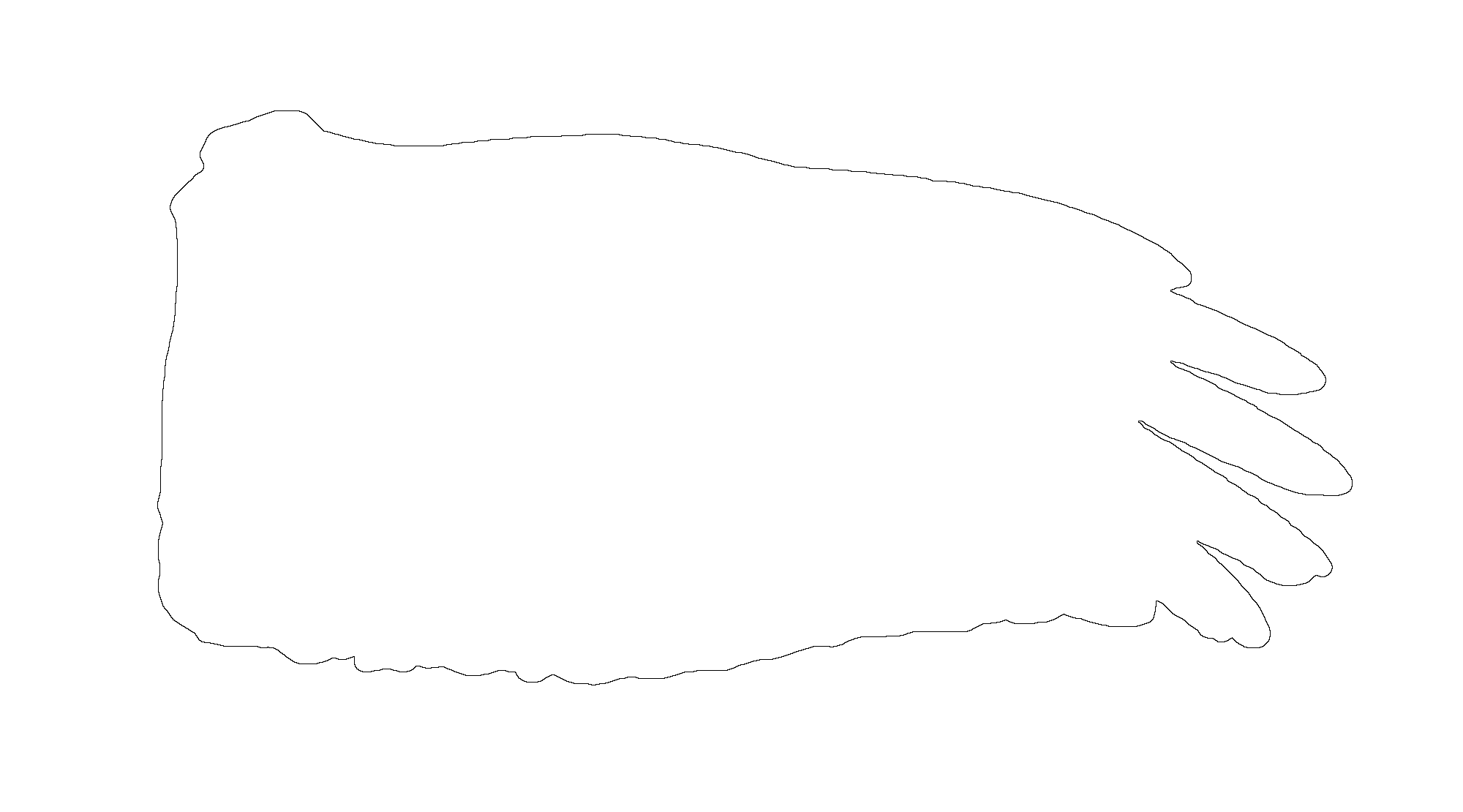

Supplement: Supplementary file 6 — Supplementary Data 4 [file 41467_2026_70692_MOESM6_ESM.zip › Supplementary Data 4/Buteo_jamaicensis.tif]

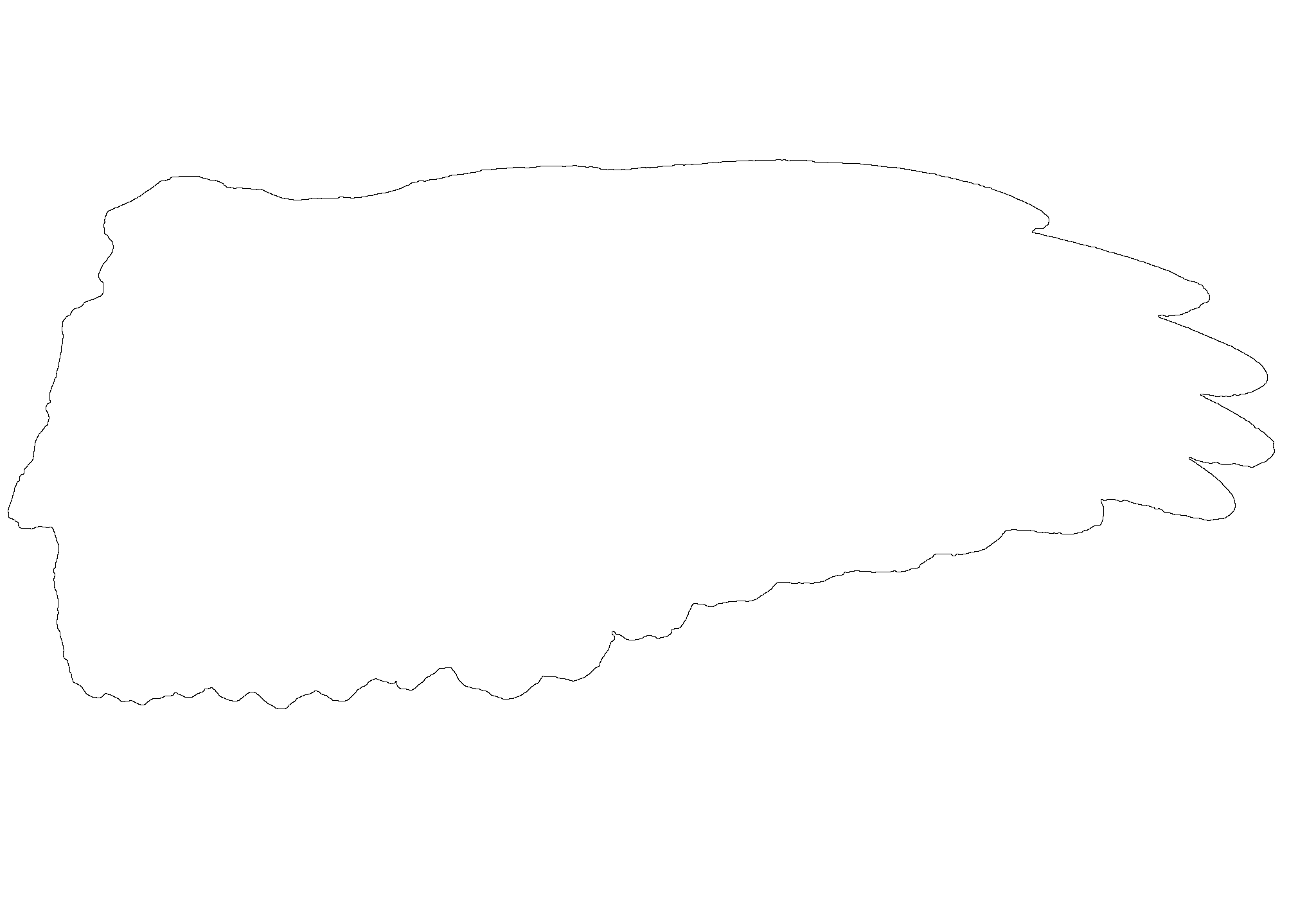

Supplement: Supplementary file 6 — Supplementary Data 4 [file 41467_2026_70692_MOESM6_ESM.zip › Supplementary Data 4/Buteo_lagopus.tif]

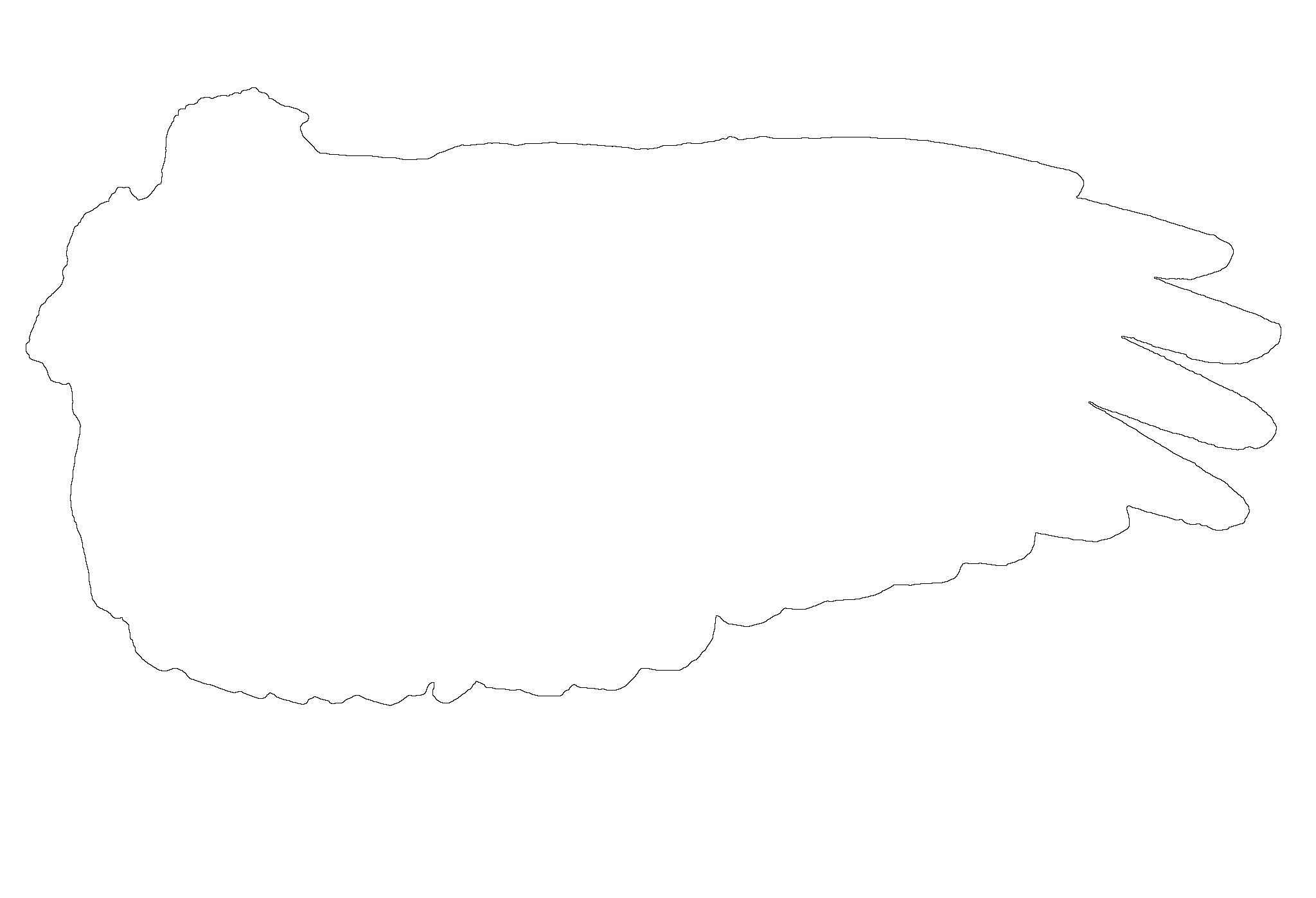

Supplement: Supplementary file 6 — Supplementary Data 4 [file 41467_2026_70692_MOESM6_ESM.zip › Supplementary Data 4/Buteo_lineatus.tif]

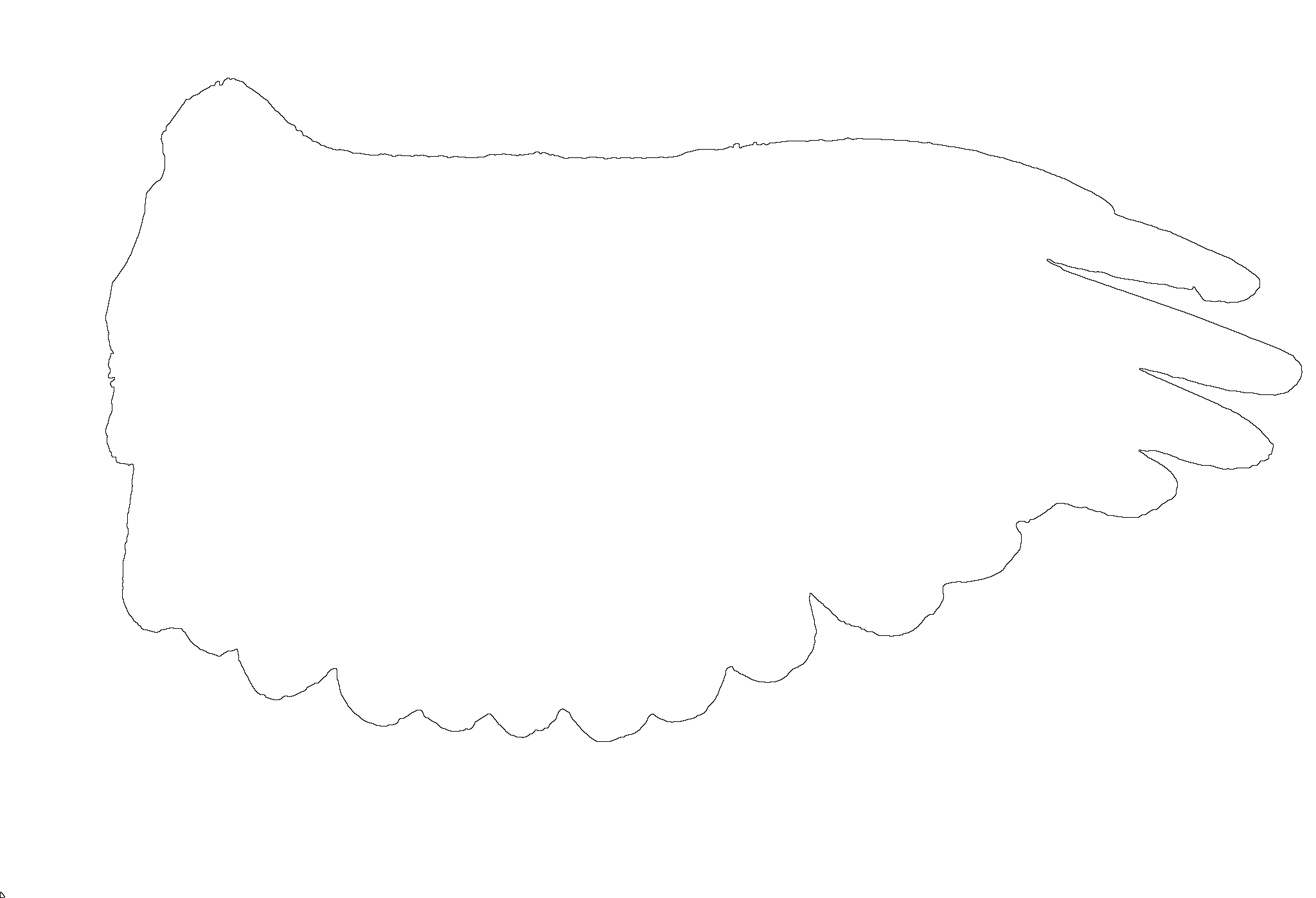

Supplement: Supplementary file 6 — Supplementary Data 4 [file 41467_2026_70692_MOESM6_ESM.zip › Supplementary Data 4/Buteo_nitidus.tif]

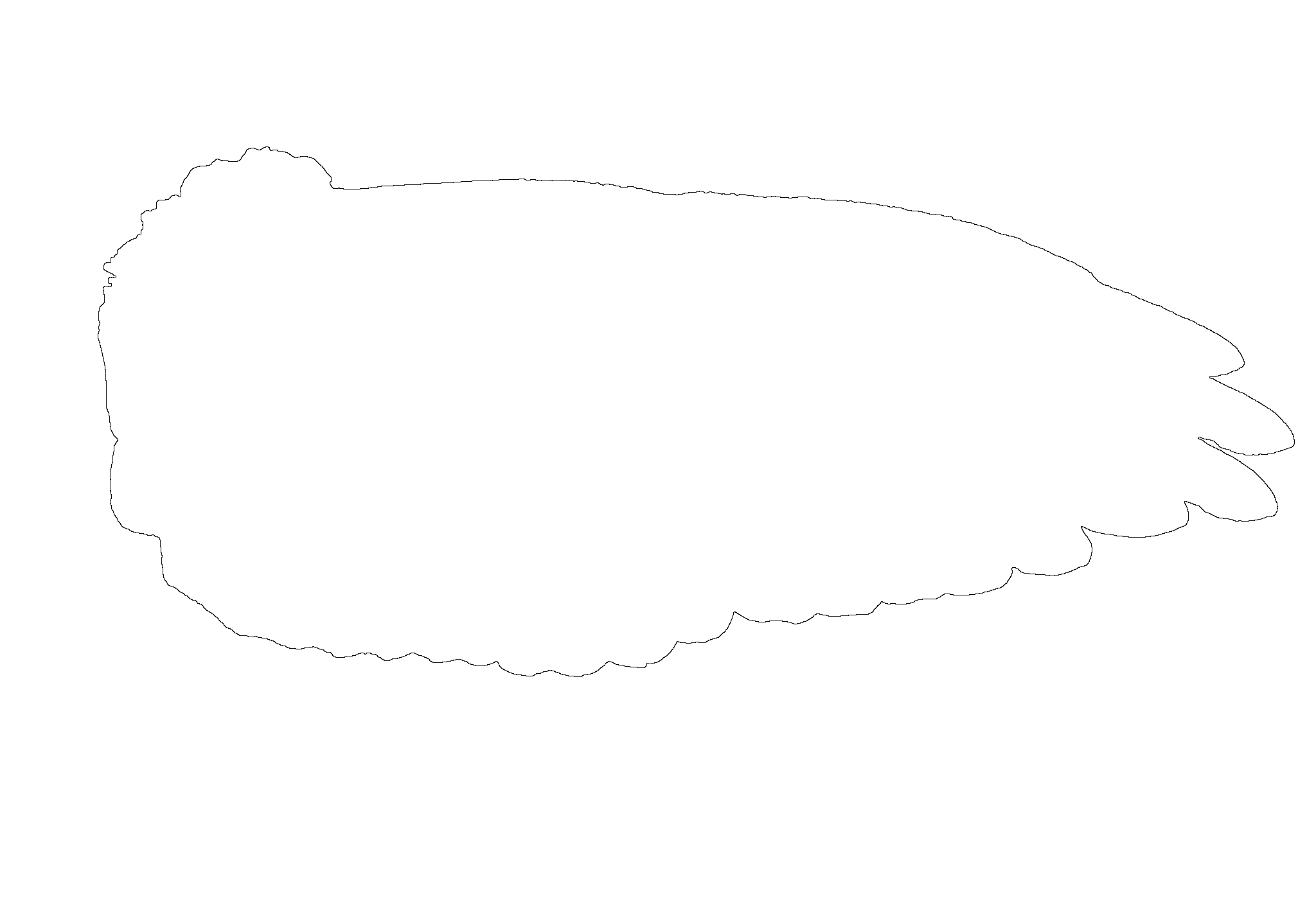

Supplement: Supplementary file 6 — Supplementary Data 4 [file 41467_2026_70692_MOESM6_ESM.zip › Supplementary Data 4/Buteo_platypterus.tif]

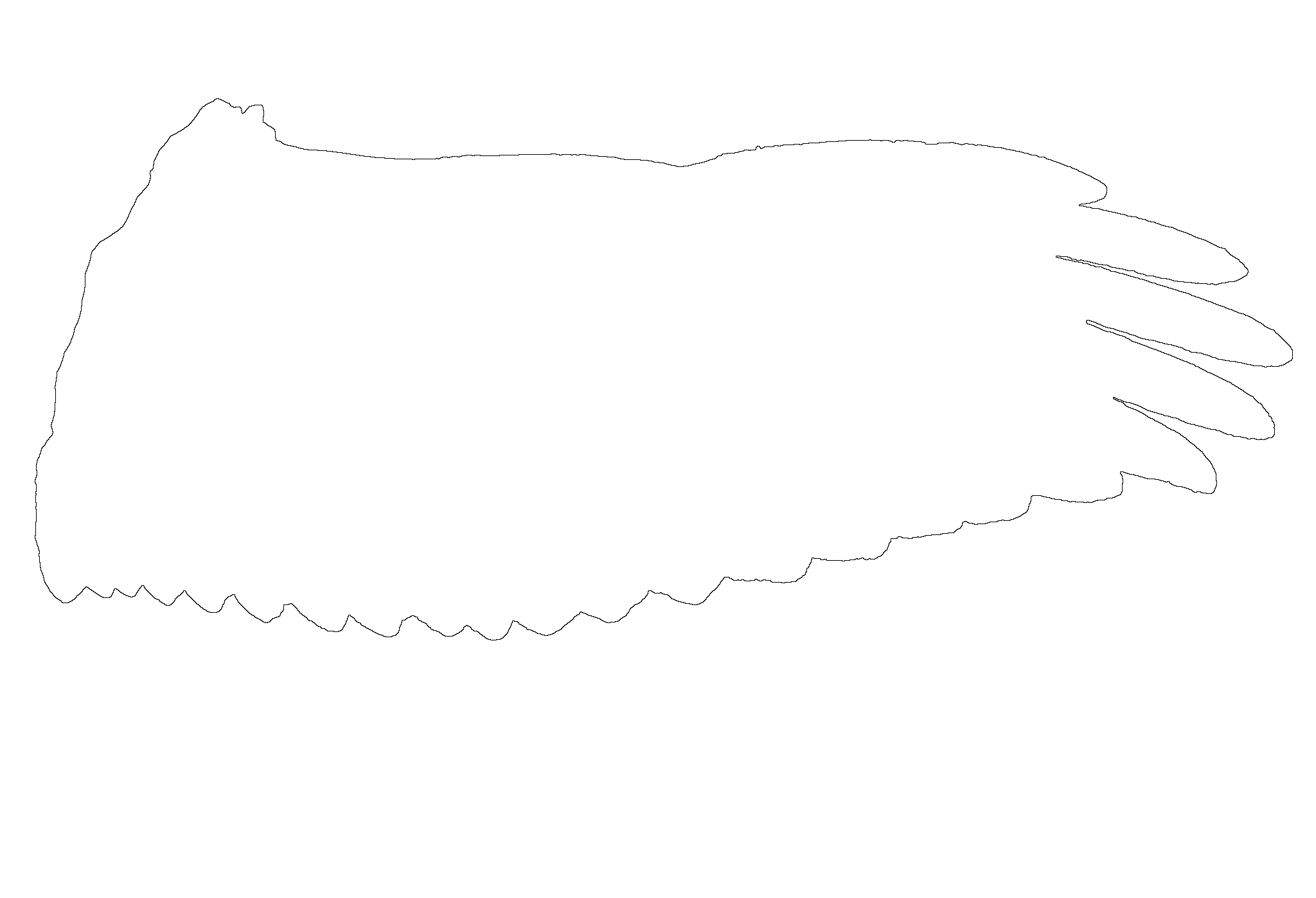

Supplement: Supplementary file 6 — Supplementary Data 4 [file 41467_2026_70692_MOESM6_ESM.zip › Supplementary Data 4/Buteo_regalis.tif]

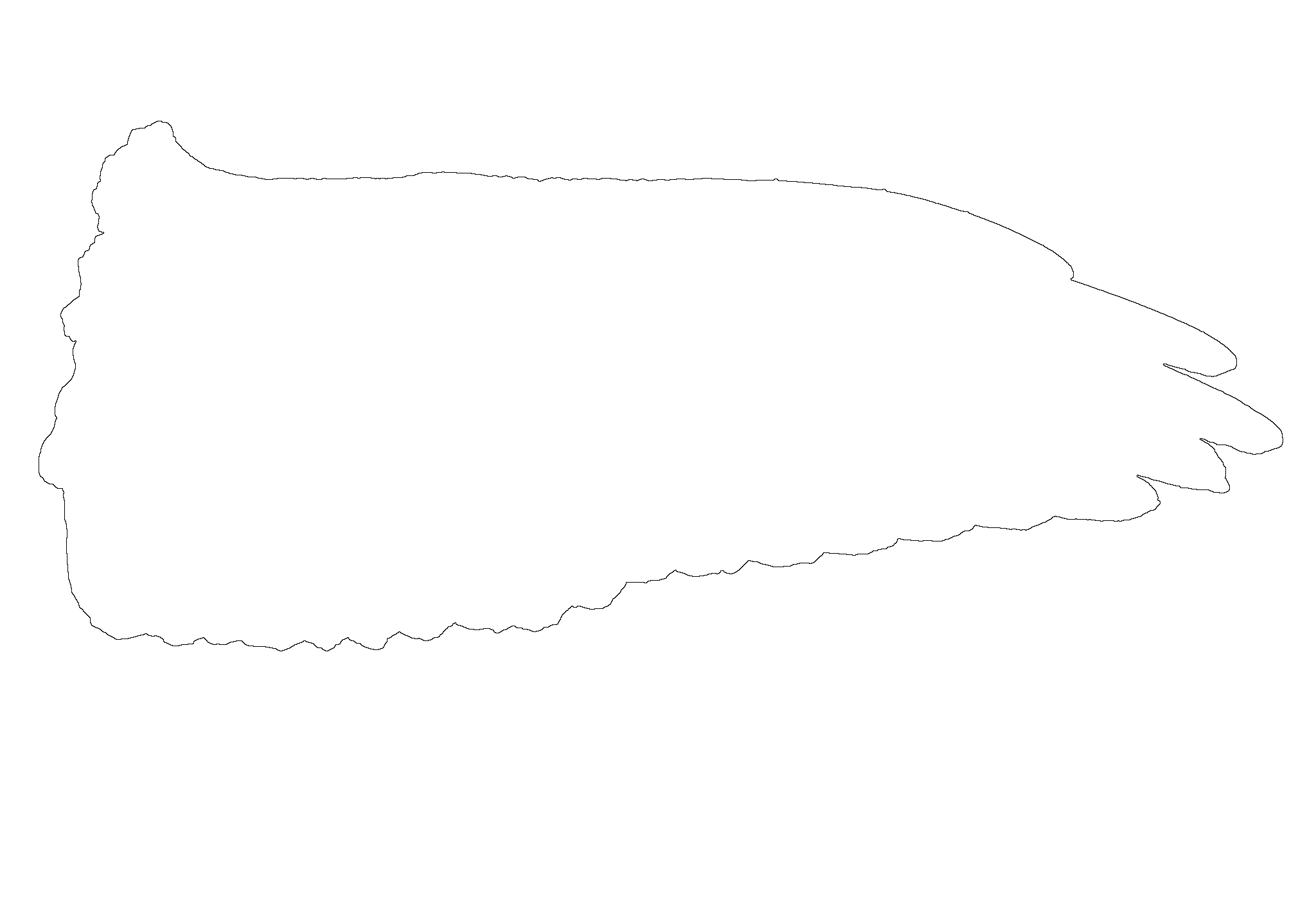

Supplement: Supplementary file 6 — Supplementary Data 4 [file 41467_2026_70692_MOESM6_ESM.zip › Supplementary Data 4/Buteo_swainsoni.tif]

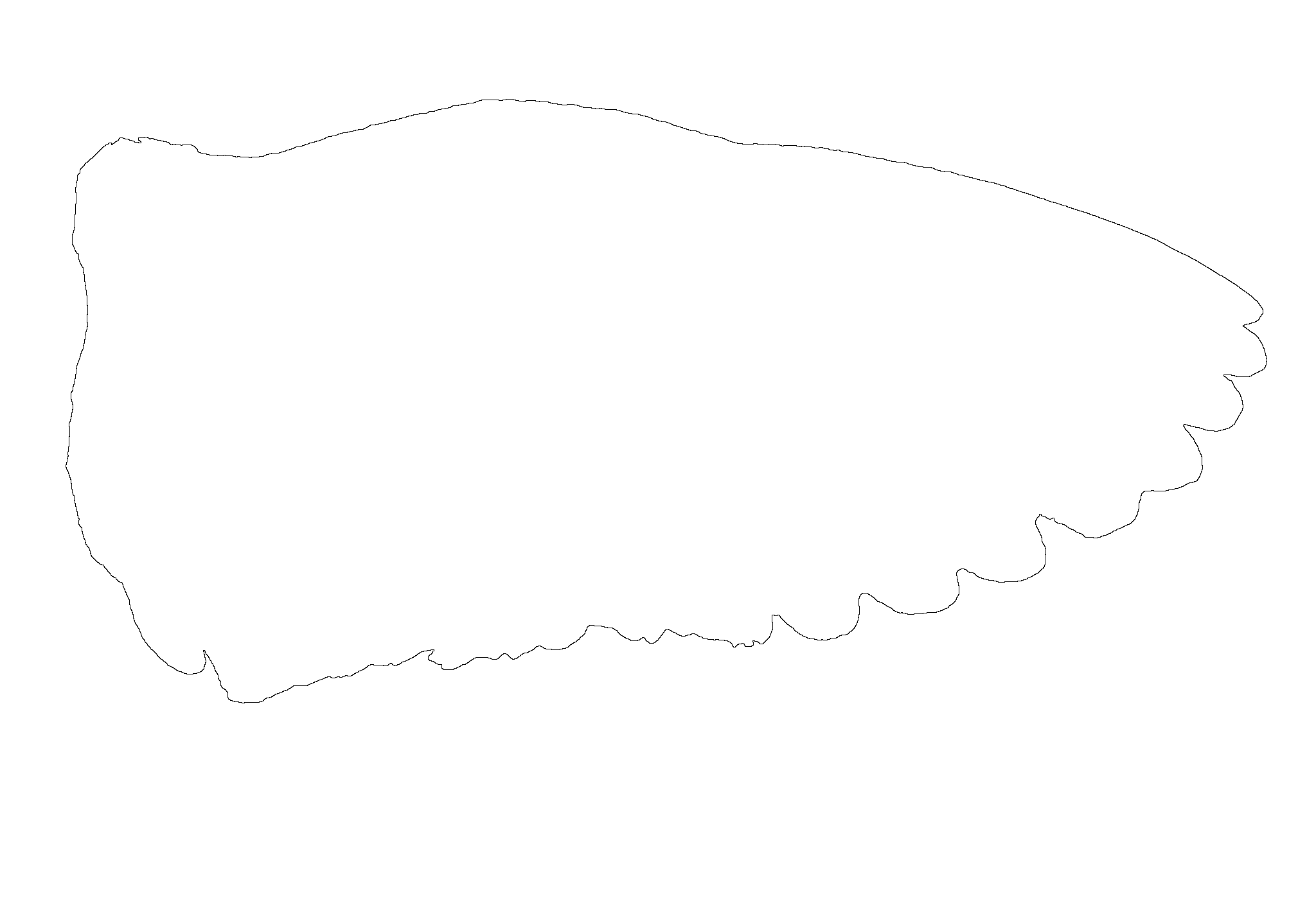

Supplement: Supplementary file 6 — Supplementary Data 4 [file 41467_2026_70692_MOESM6_ESM.zip › Supplementary Data 4/Butorides_virescens.tif]

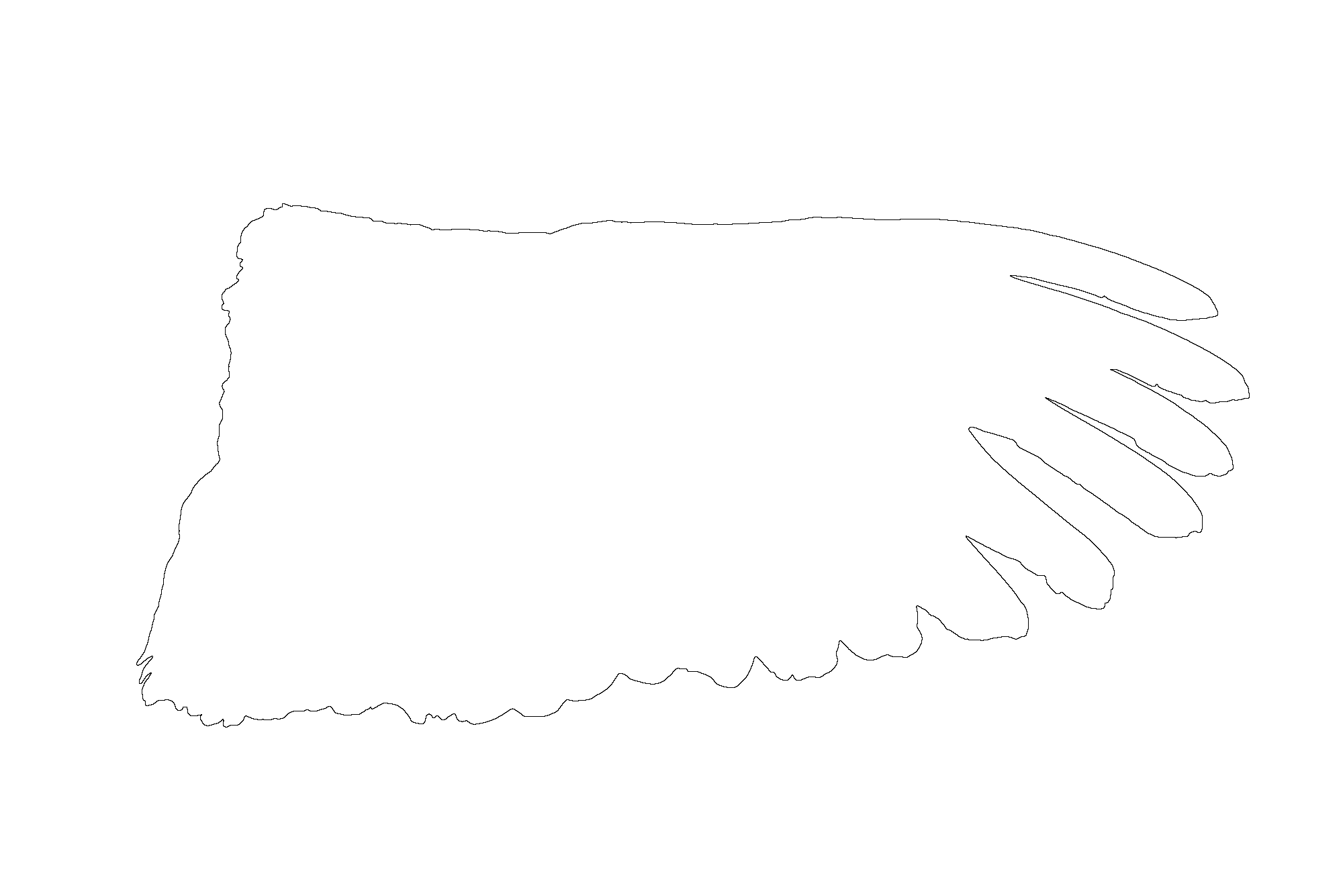

Supplement: Supplementary file 6 — Supplementary Data 4 [file 41467_2026_70692_MOESM6_ESM.zip › Supplementary Data 4/Cacatua_galerita.tif]

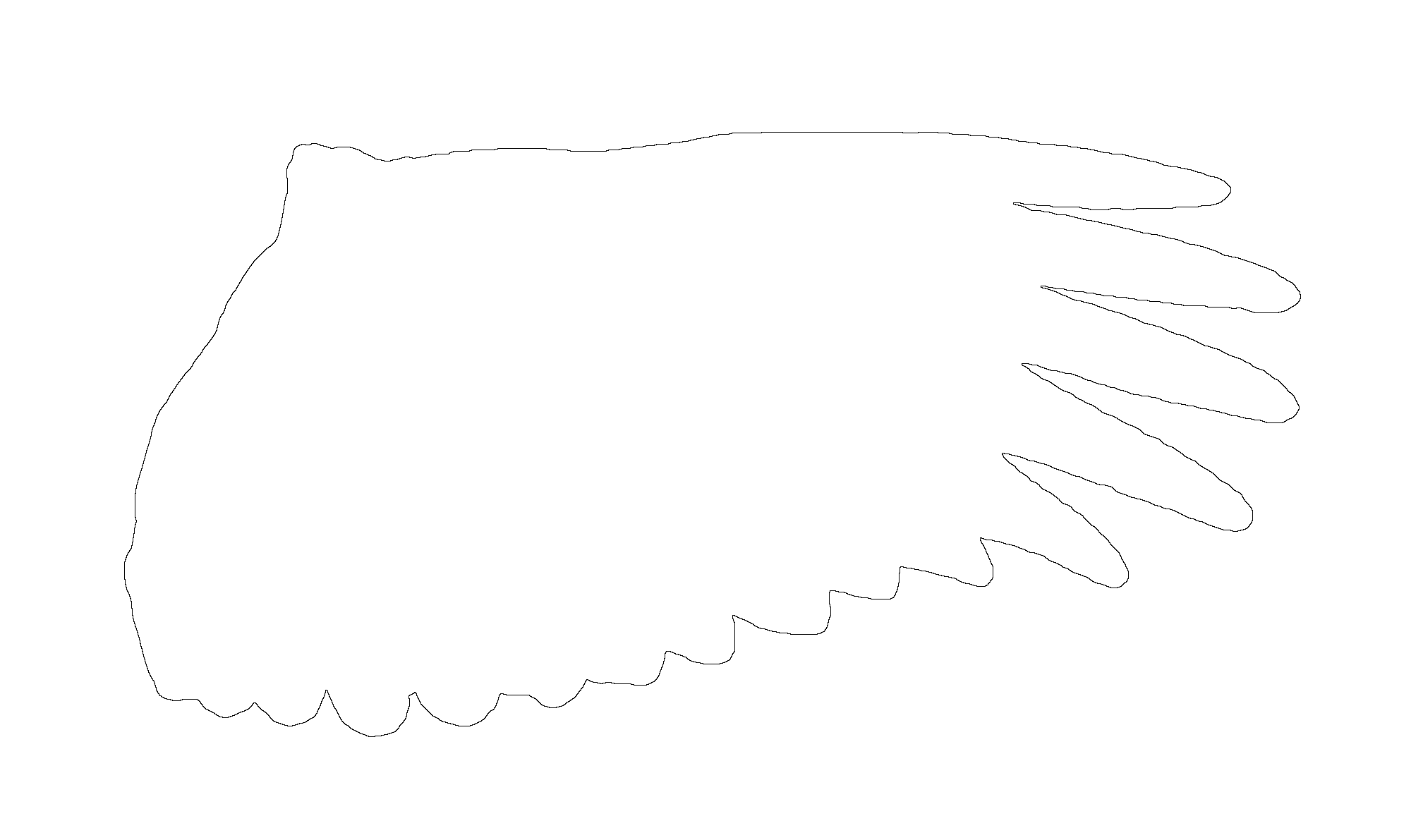

Supplement: Supplementary file 6 — Supplementary Data 4 [file 41467_2026_70692_MOESM6_ESM.zip › Supplementary Data 4/Cacicus_cela.tif]

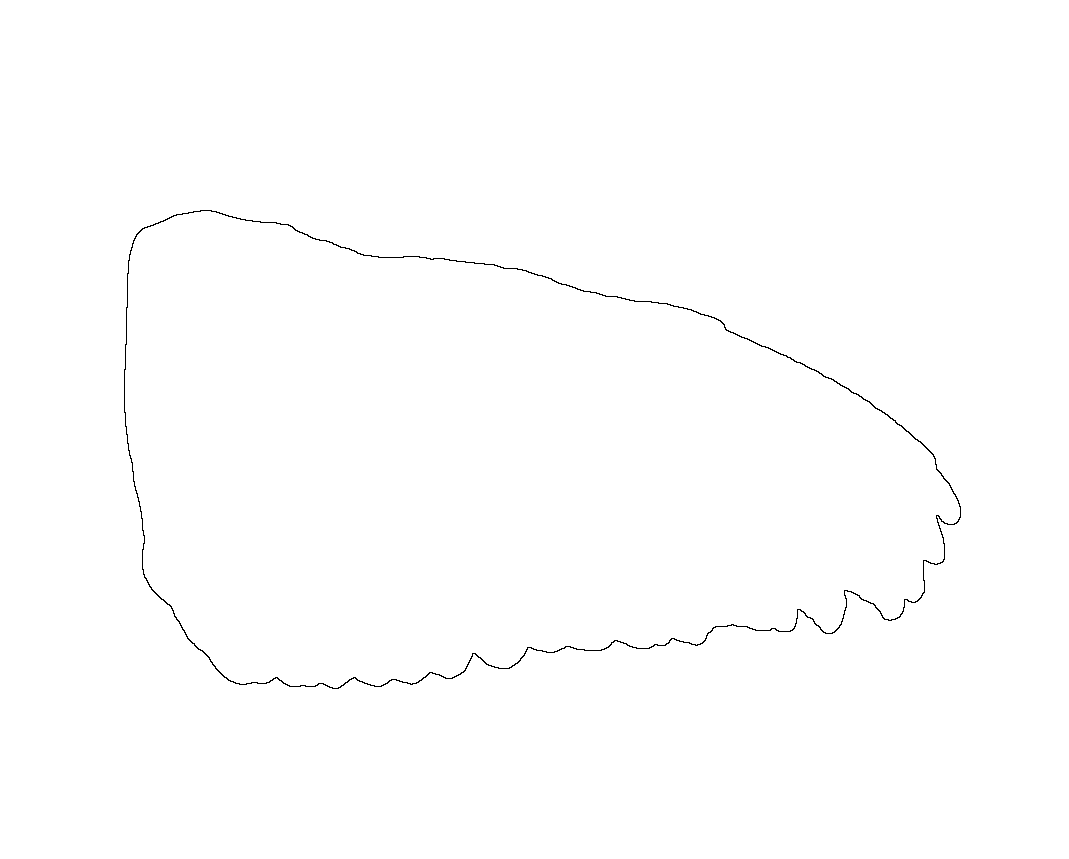

Supplement: Supplementary file 6 — Supplementary Data 4 [file 41467_2026_70692_MOESM6_ESM.zip › Supplementary Data 4/Cairina_moschata.tif]

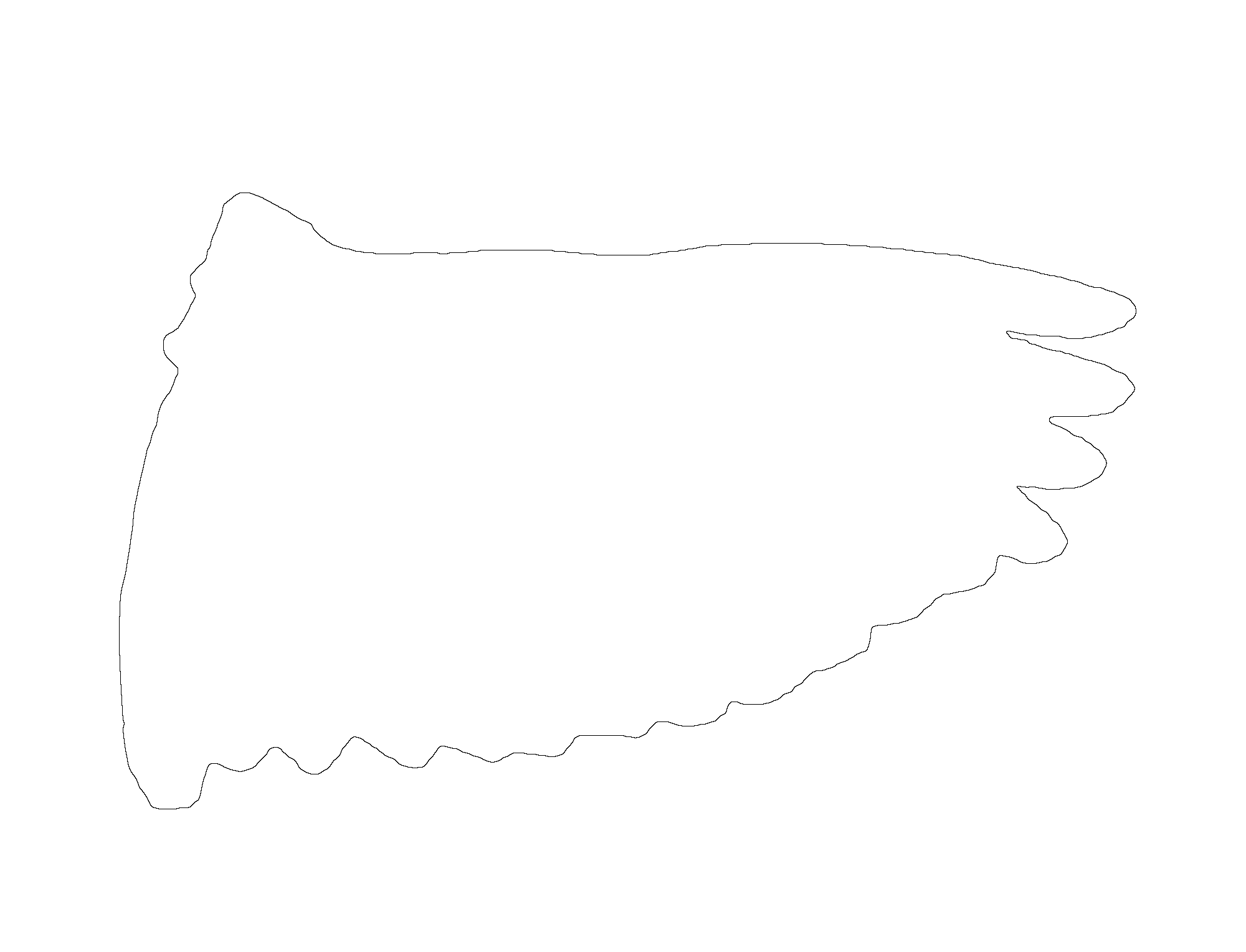

Supplement: Supplementary file 6 — Supplementary Data 4 [file 41467_2026_70692_MOESM6_ESM.zip › Supplementary Data 4/Calamospiza_melanocorys.tif]

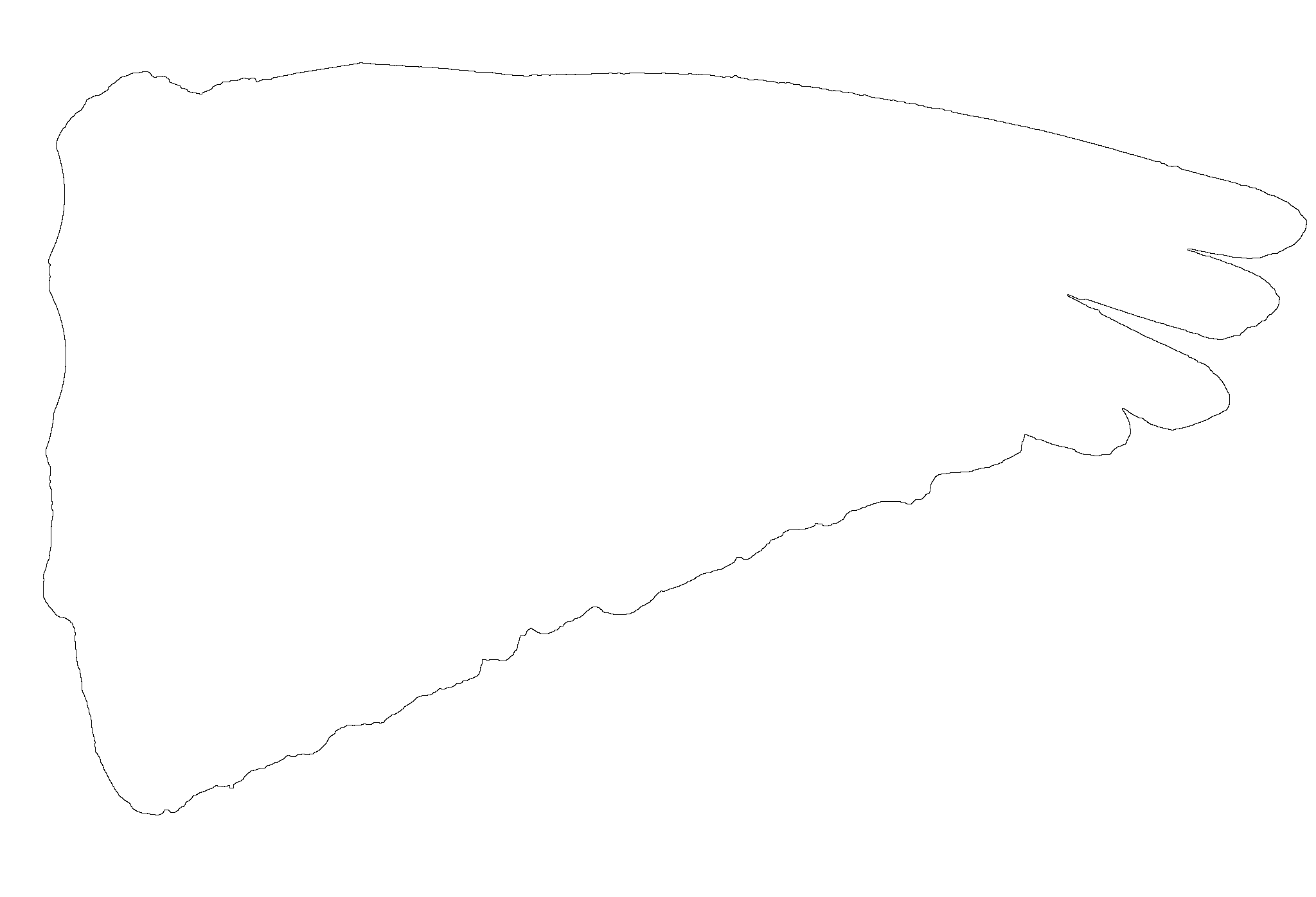

Supplement: Supplementary file 6 — Supplementary Data 4 [file 41467_2026_70692_MOESM6_ESM.zip › Supplementary Data 4/Calcarius_lapponicus.tif]

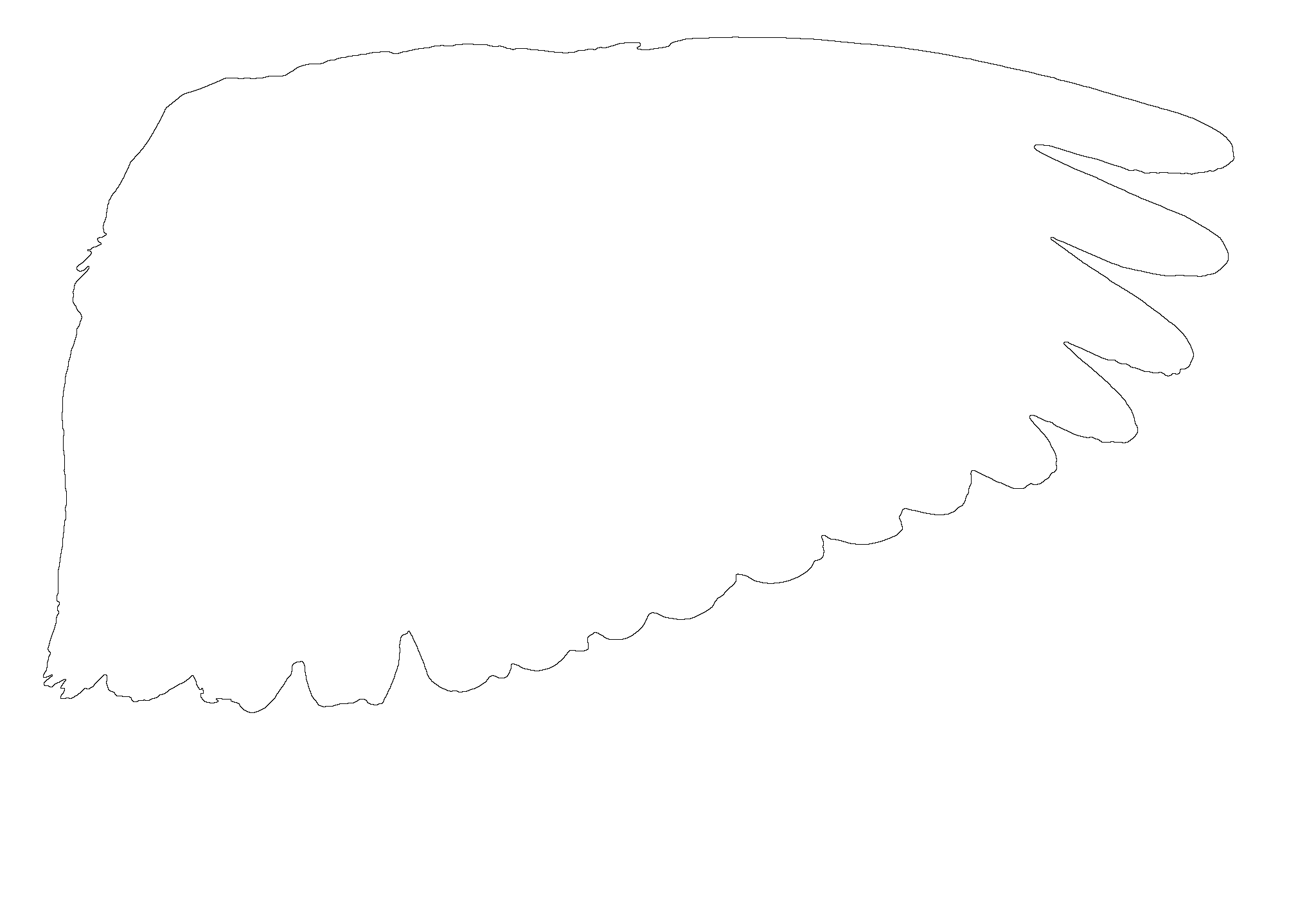

Supplement: Supplementary file 6 — Supplementary Data 4 [file 41467_2026_70692_MOESM6_ESM.zip › Supplementary Data 4/Calcarius_ornatus.tif]

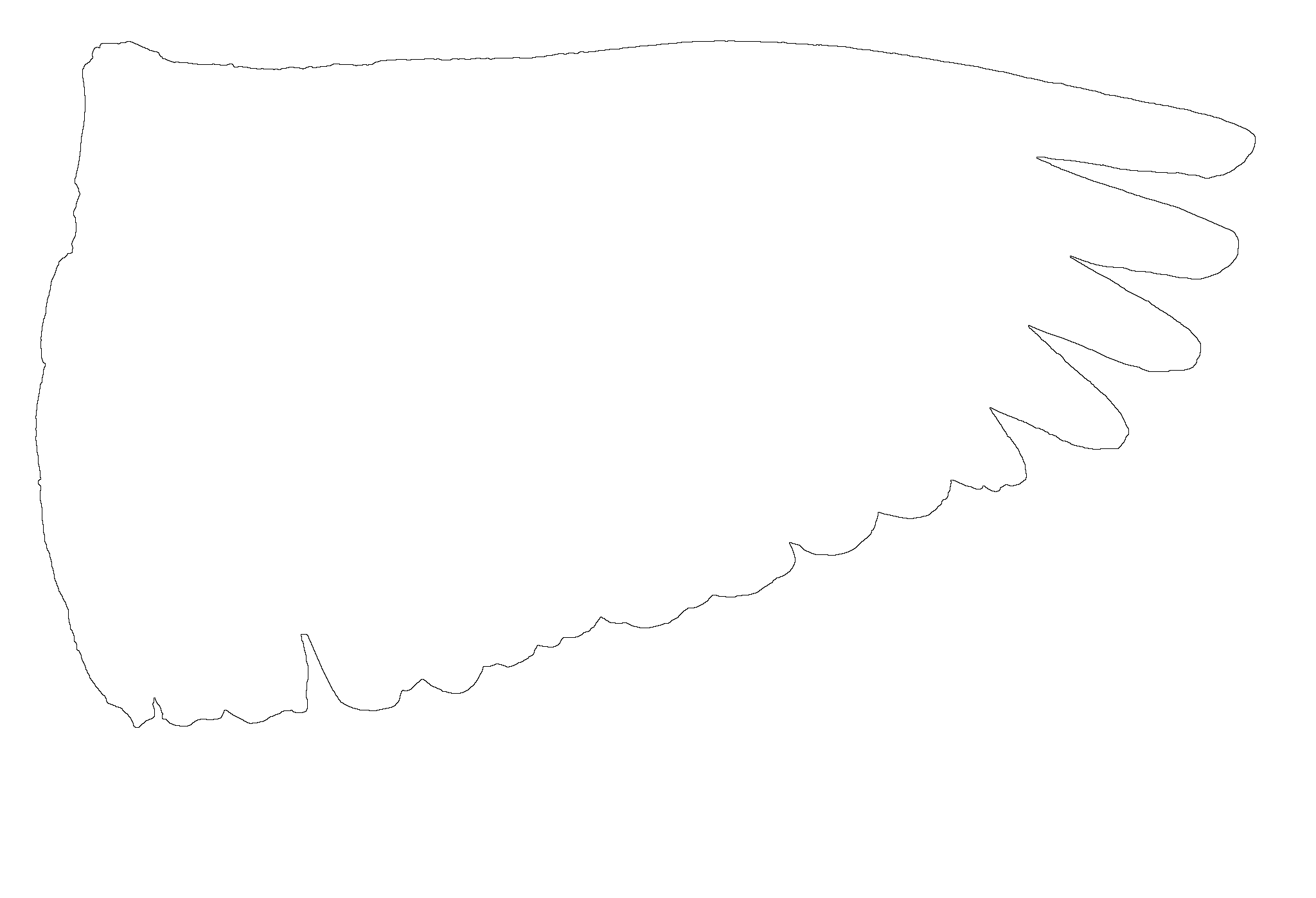

Supplement: Supplementary file 6 — Supplementary Data 4 [file 41467_2026_70692_MOESM6_ESM.zip › Supplementary Data 4/Calcarius_pictus.tif]

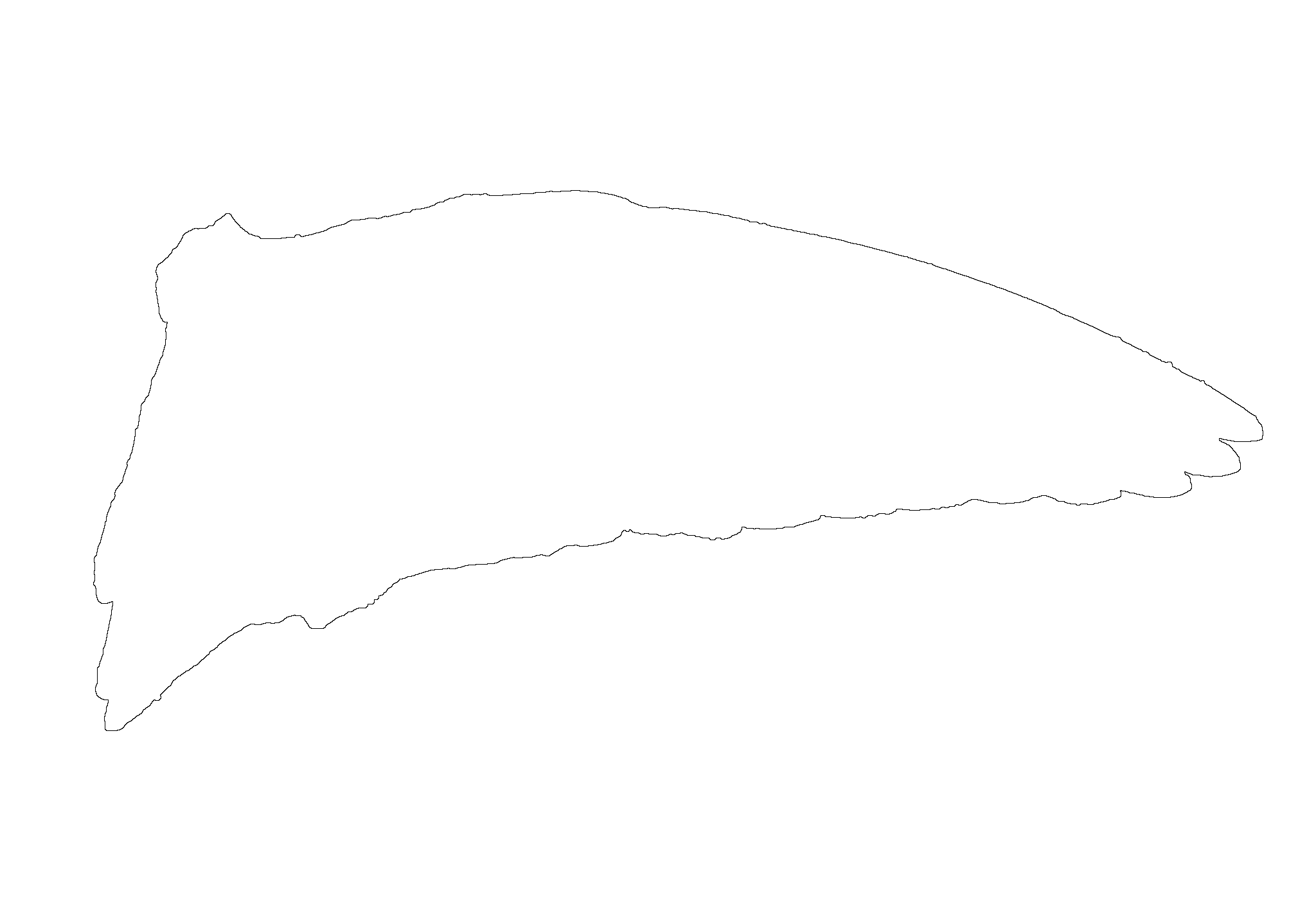

Supplement: Supplementary file 6 — Supplementary Data 4 [file 41467_2026_70692_MOESM6_ESM.zip › Supplementary Data 4/Calidris_acuminata.tif]

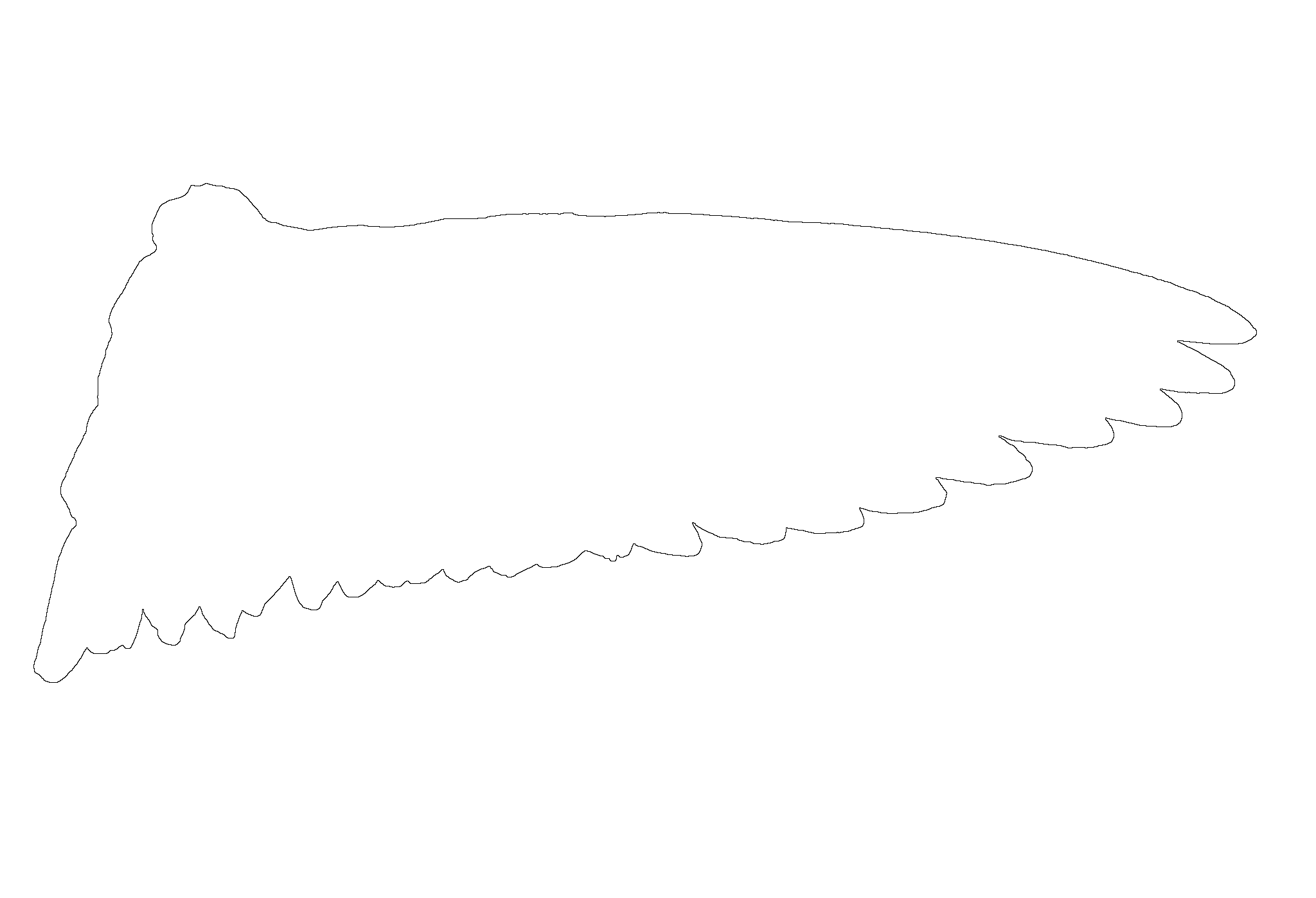

Supplement: Supplementary file 6 — Supplementary Data 4 [file 41467_2026_70692_MOESM6_ESM.zip › Supplementary Data 4/Calidris_alba.tif]

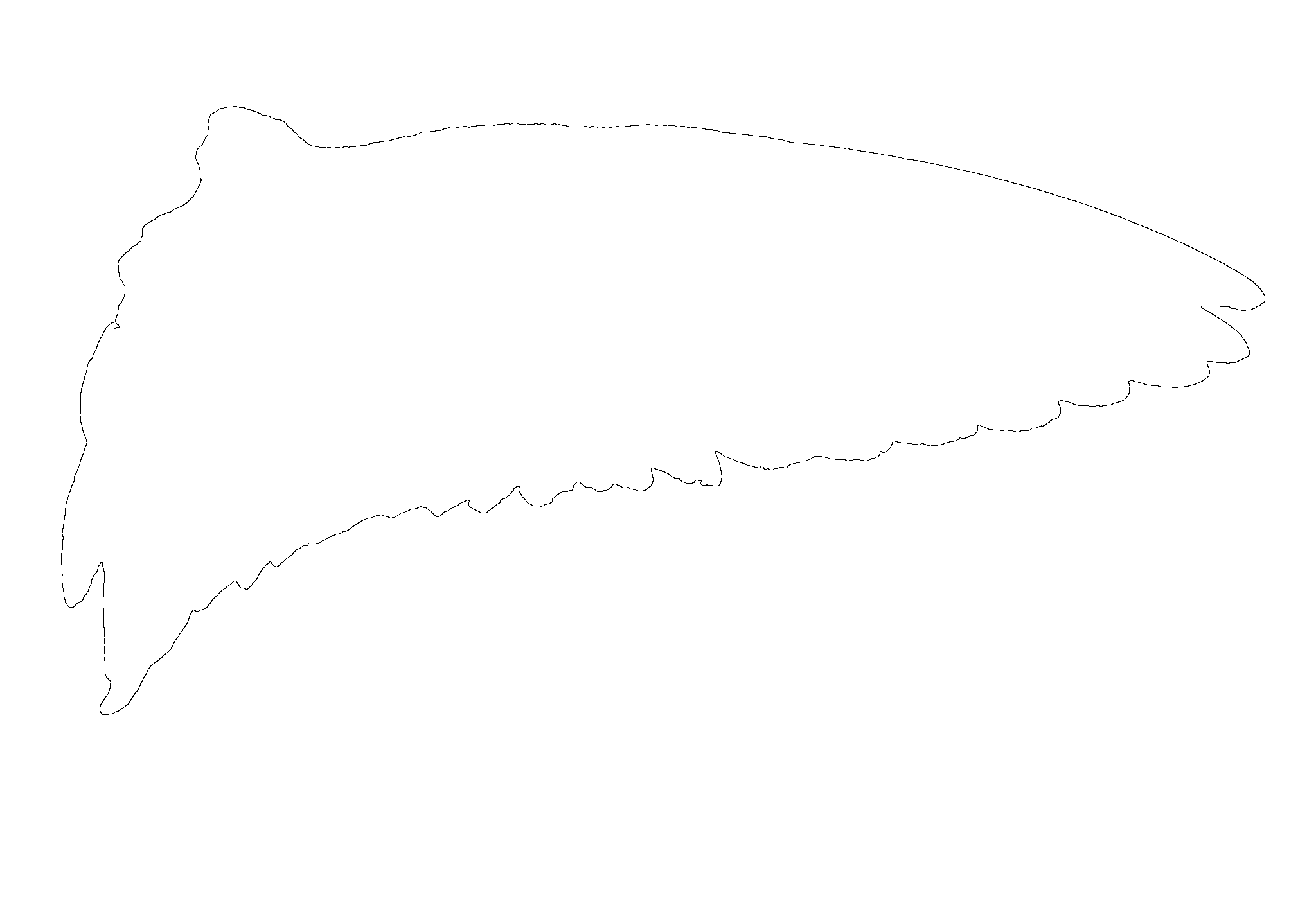

Supplement: Supplementary file 6 — Supplementary Data 4 [file 41467_2026_70692_MOESM6_ESM.zip › Supplementary Data 4/Calidris_alpina.tif]

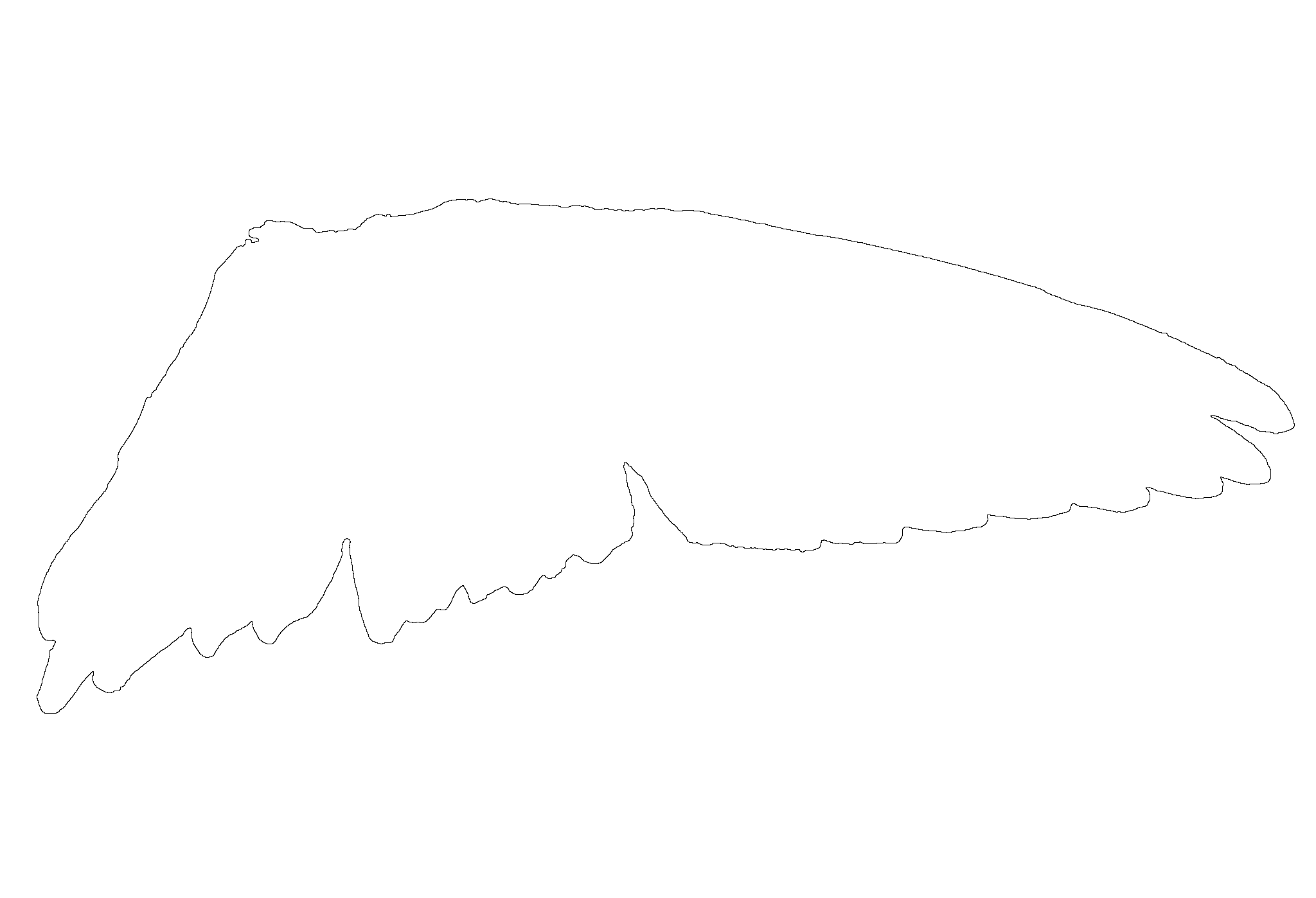

Supplement: Supplementary file 6 — Supplementary Data 4 [file 41467_2026_70692_MOESM6_ESM.zip › Supplementary Data 4/Calidris_bairdii.tif]

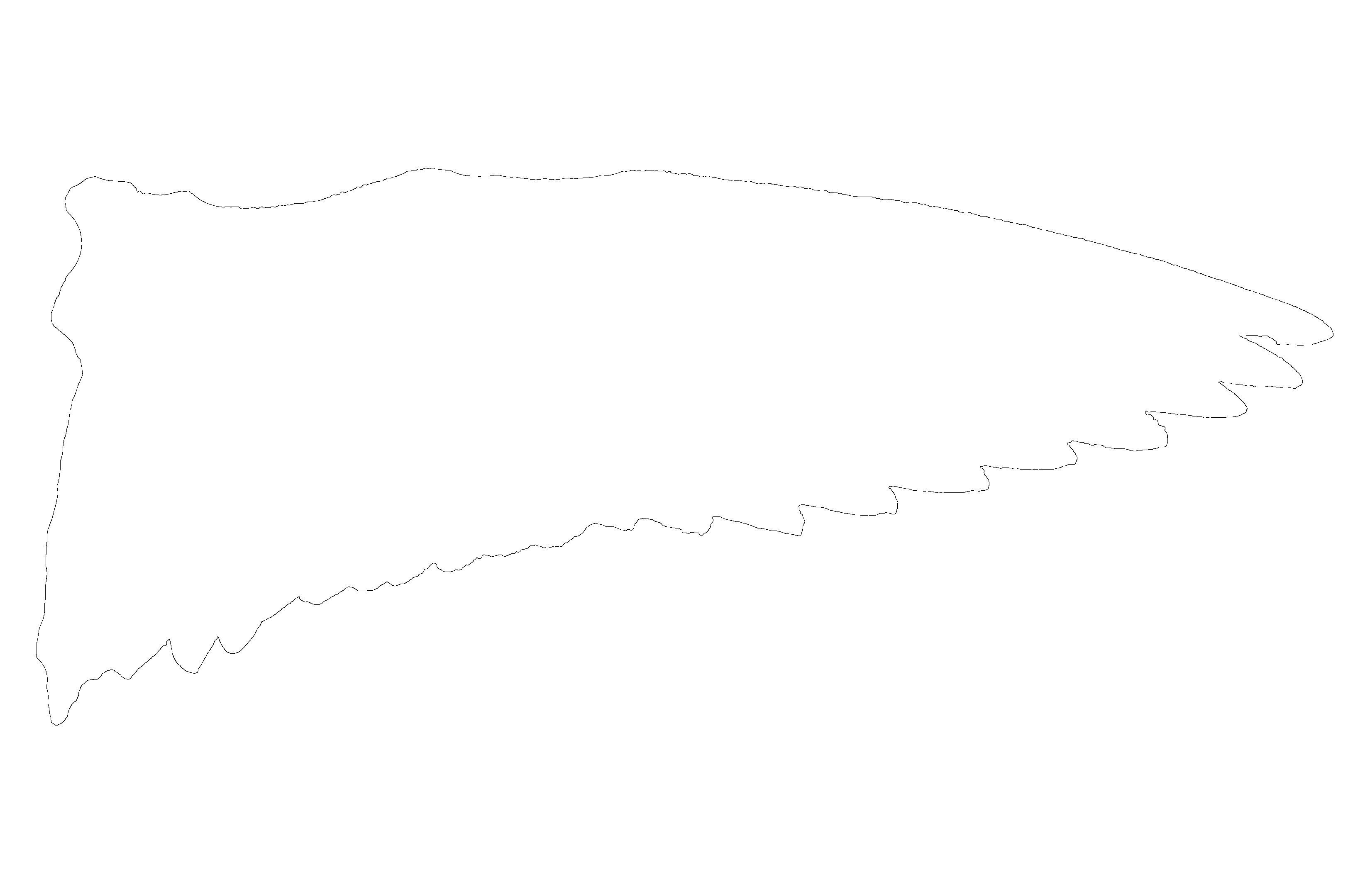

Supplement: Supplementary file 6 — Supplementary Data 4 [file 41467_2026_70692_MOESM6_ESM.zip › Supplementary Data 4/Calidris_canutus.tif]

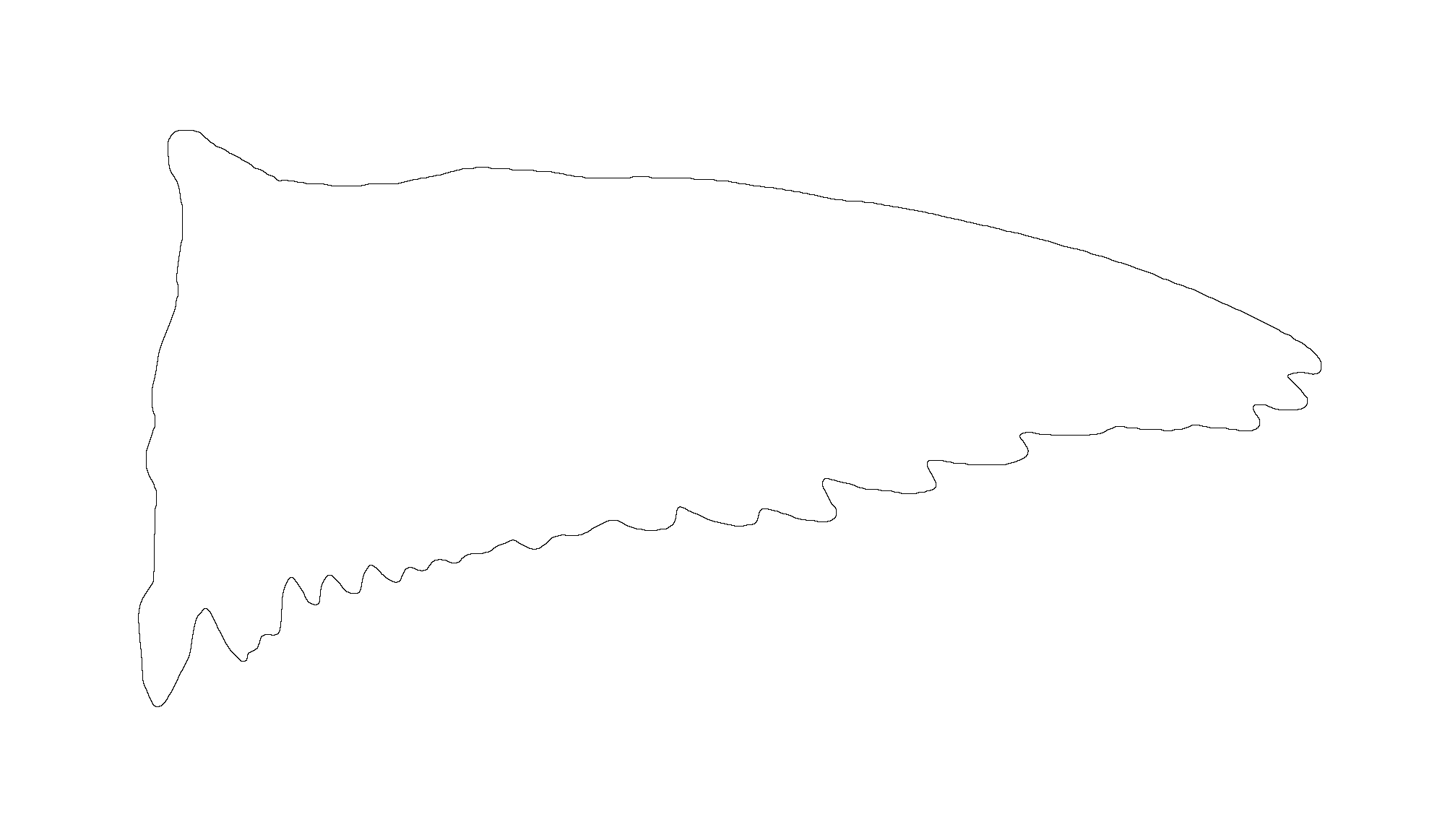

Supplement: Supplementary file 6 — Supplementary Data 4 [file 41467_2026_70692_MOESM6_ESM.zip › Supplementary Data 4/Calidris_ferruginea.tif]

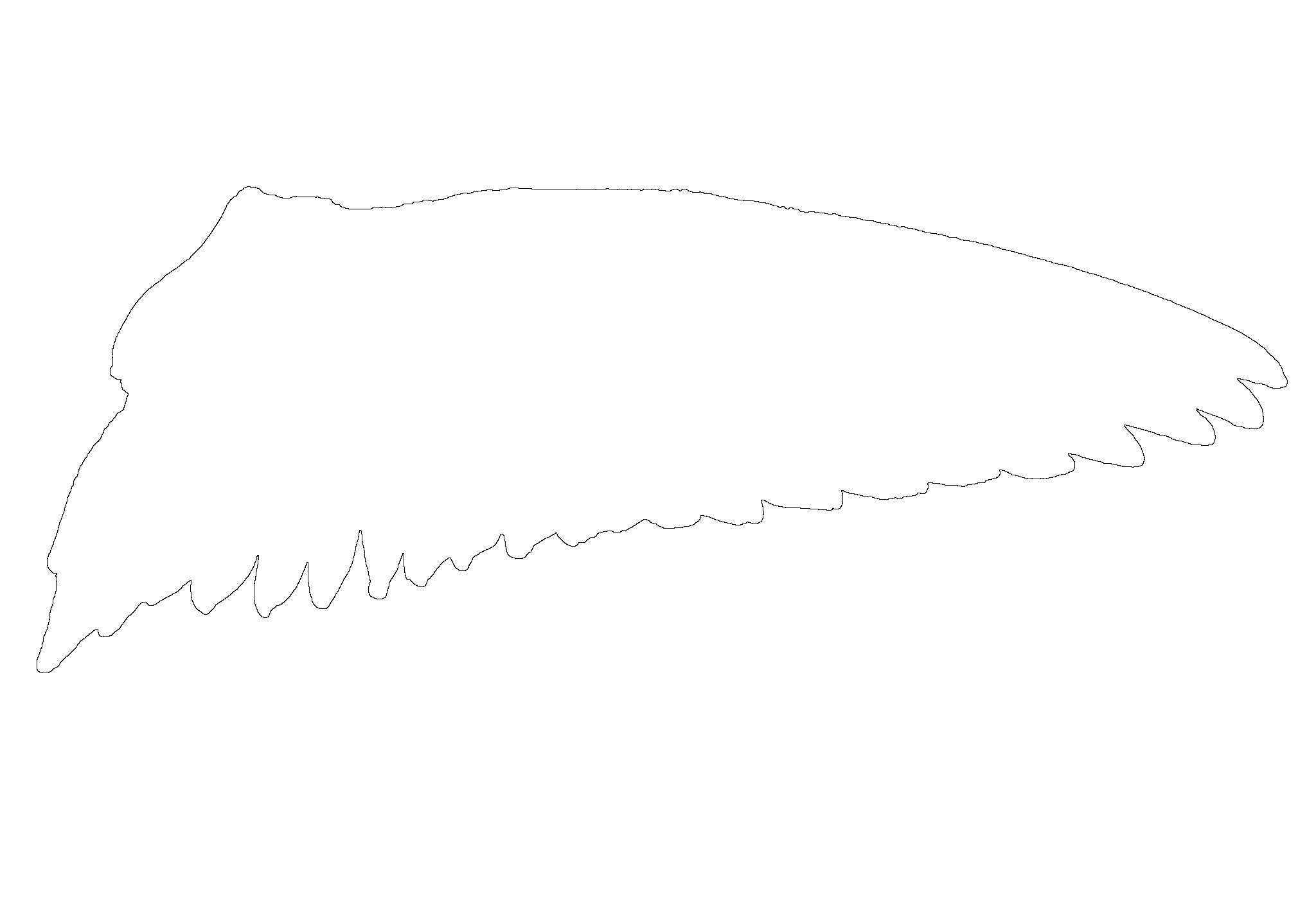

Supplement: Supplementary file 6 — Supplementary Data 4 [file 41467_2026_70692_MOESM6_ESM.zip › Supplementary Data 4/Calidris_fuscicollis.tif]

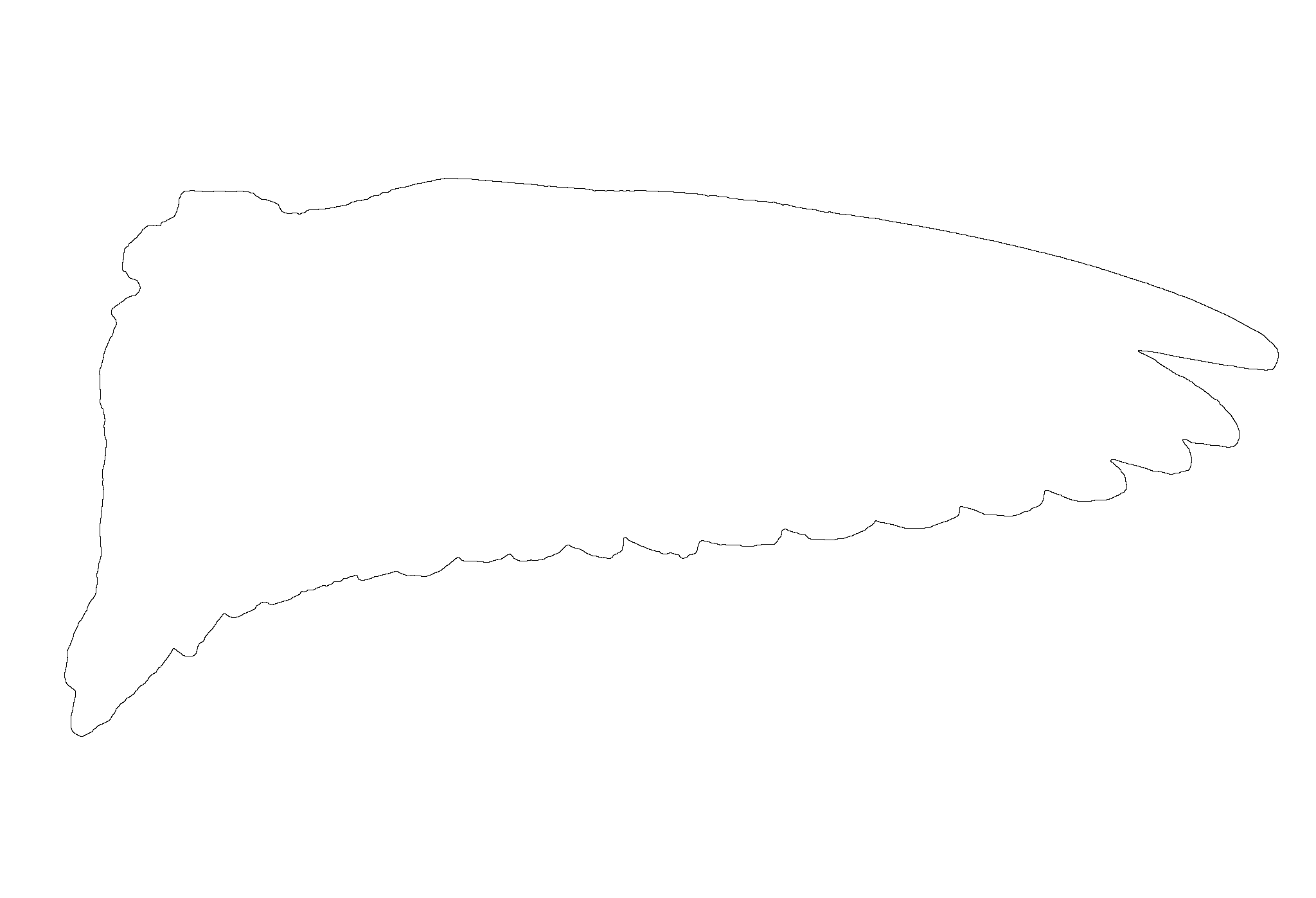

Supplement: Supplementary file 6 — Supplementary Data 4 [file 41467_2026_70692_MOESM6_ESM.zip › Supplementary Data 4/Calidris_himantopus.tif]

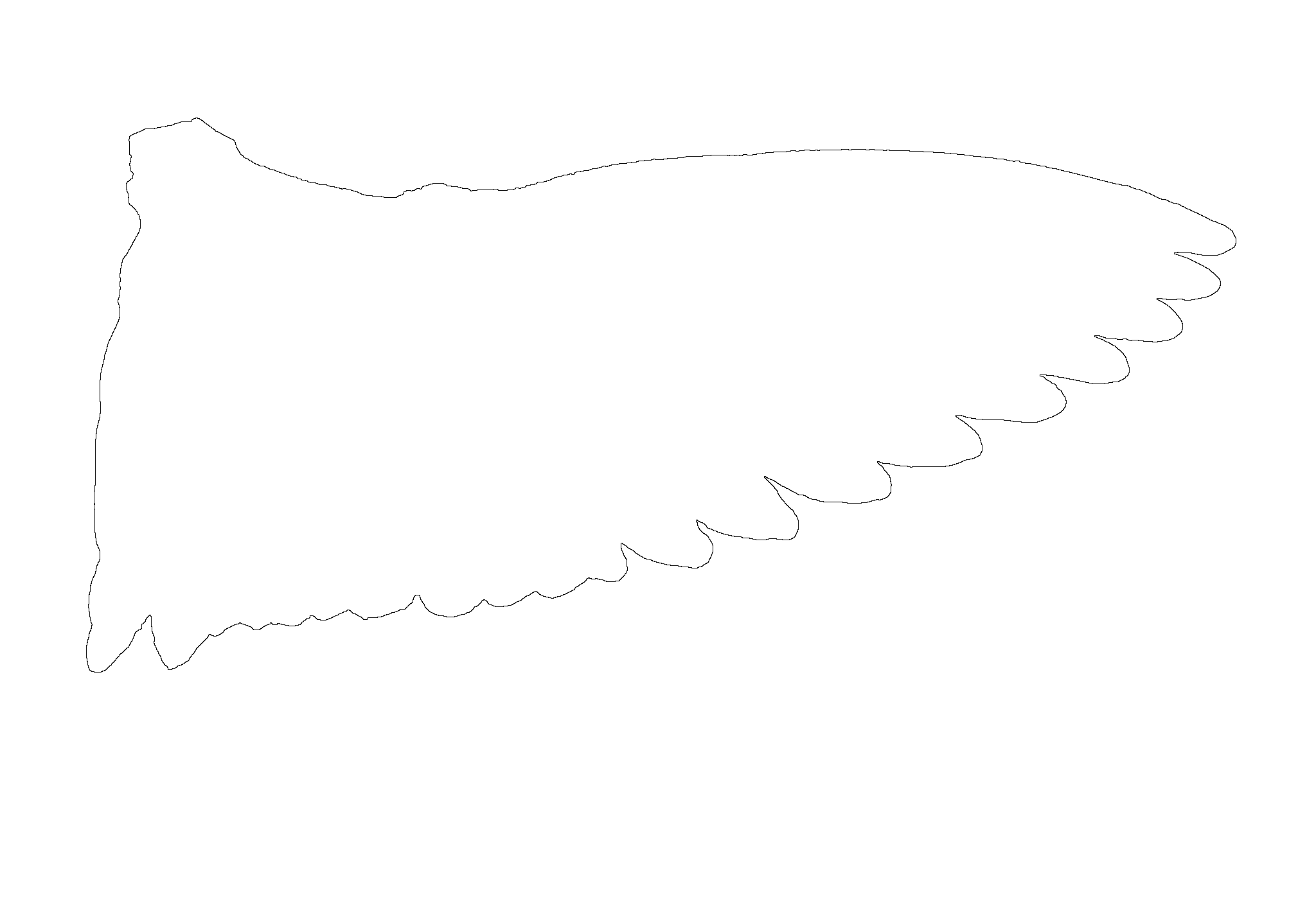

Supplement: Supplementary file 6 — Supplementary Data 4 [file 41467_2026_70692_MOESM6_ESM.zip › Supplementary Data 4/Calidris_maritima.tif]

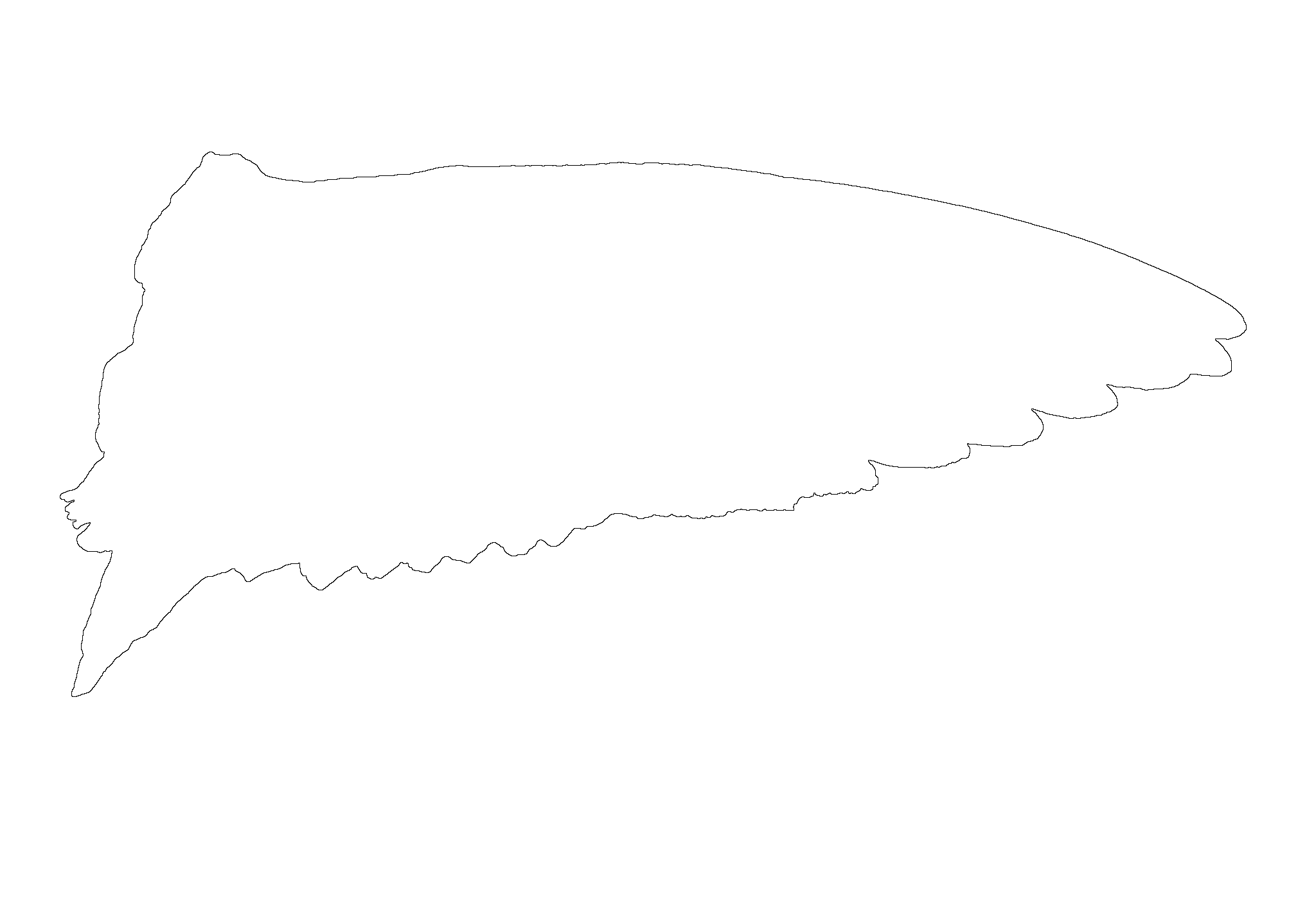

Supplement: Supplementary file 6 — Supplementary Data 4 [file 41467_2026_70692_MOESM6_ESM.zip › Supplementary Data 4/Calidris_mauri.tif]

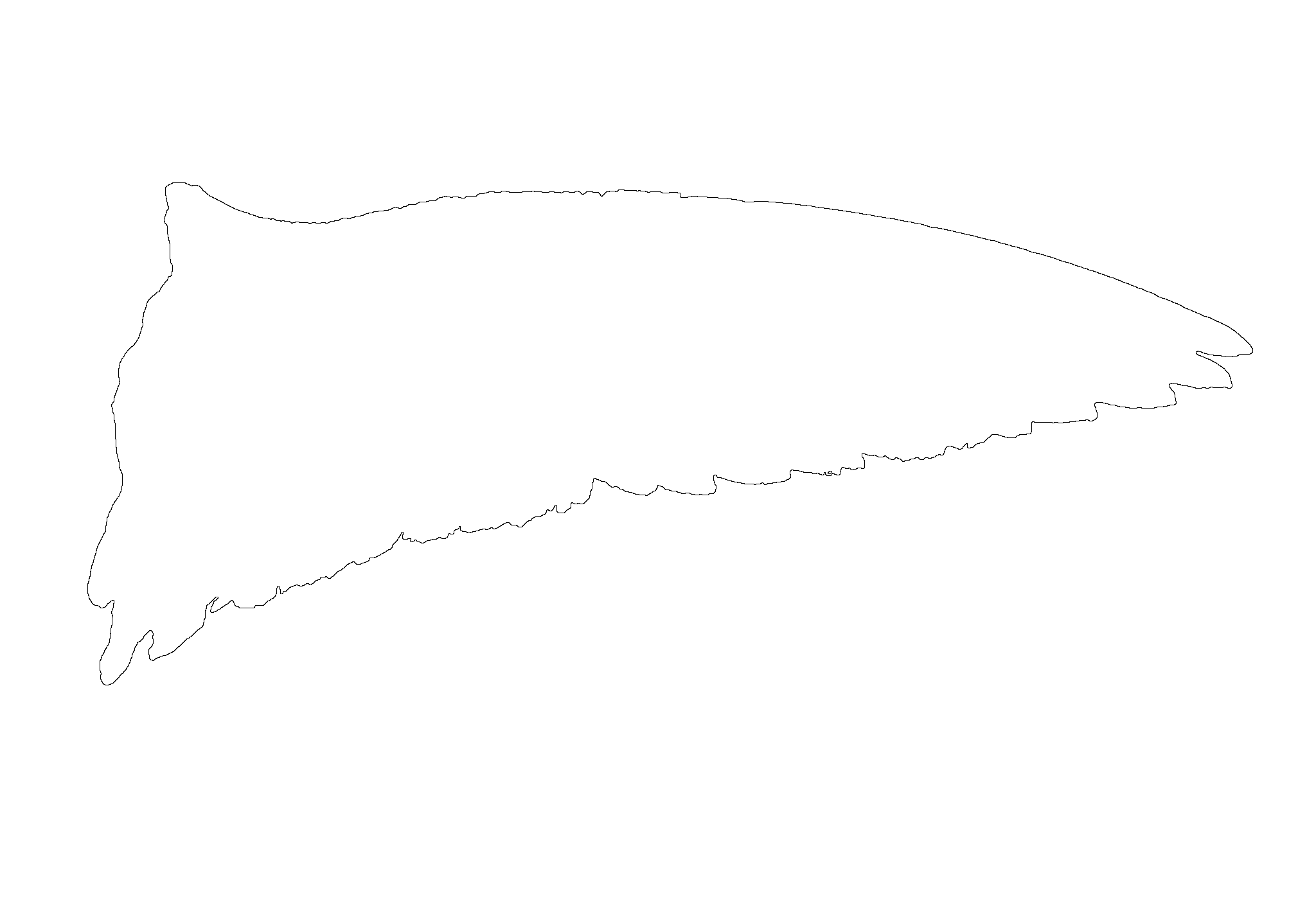

Supplement: Supplementary file 6 — Supplementary Data 4 [file 41467_2026_70692_MOESM6_ESM.zip › Supplementary Data 4/Calidris_melanotos.tif]

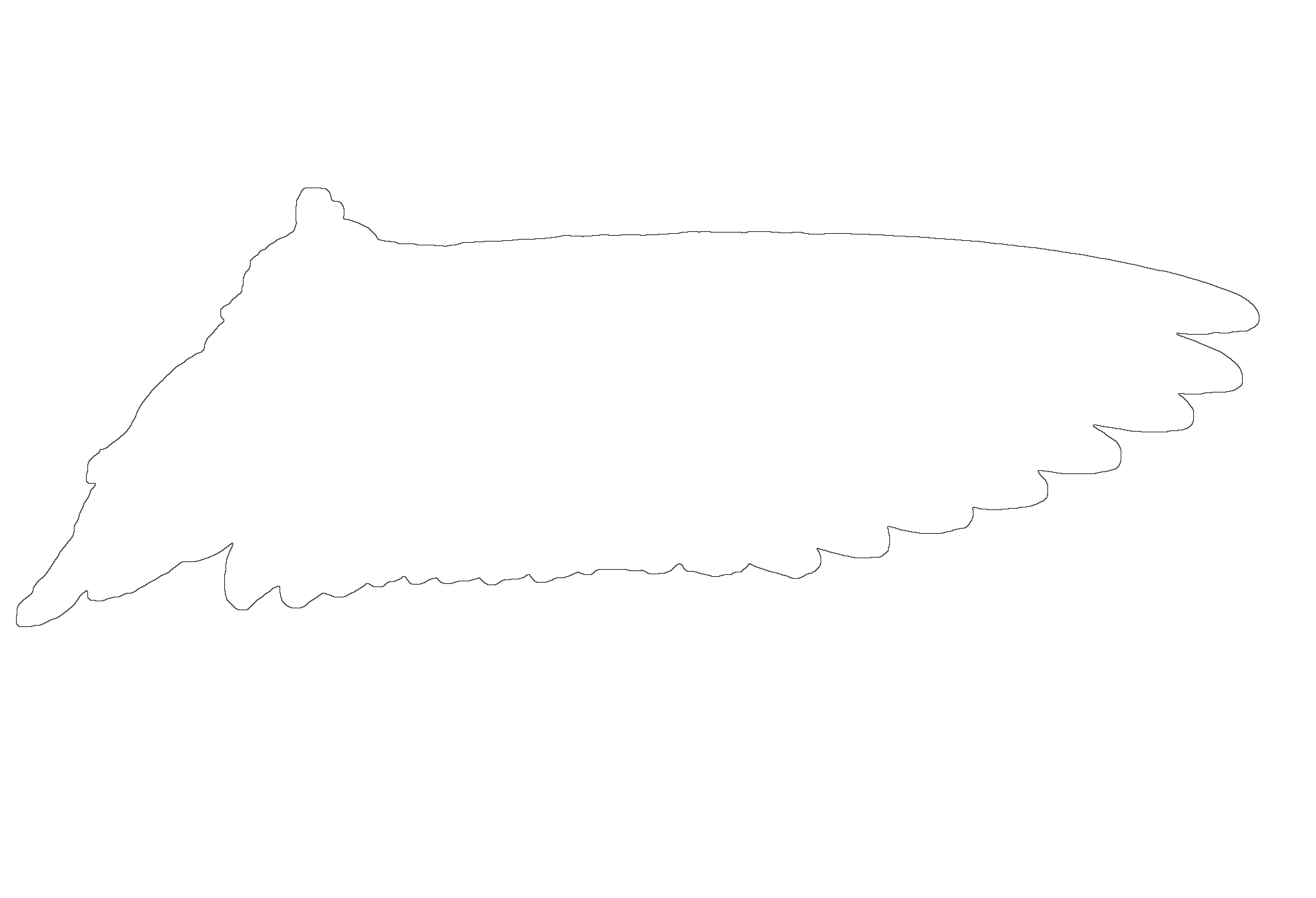

Supplement: Supplementary file 6 — Supplementary Data 4 [file 41467_2026_70692_MOESM6_ESM.zip › Supplementary Data 4/Calidris_minuta.tif]

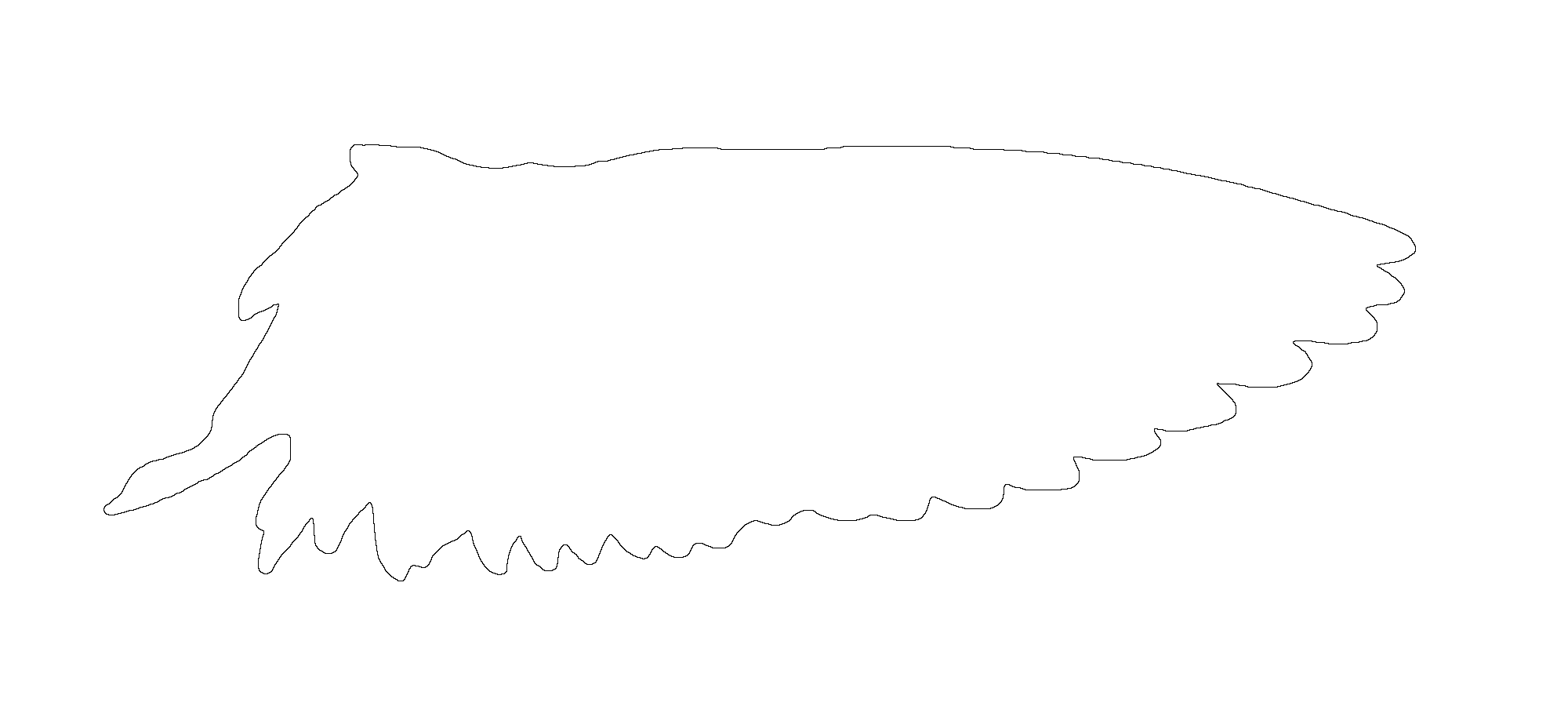

Supplement: Supplementary file 6 — Supplementary Data 4 [file 41467_2026_70692_MOESM6_ESM.zip › Supplementary Data 4/Calidris_minutilla.tif]

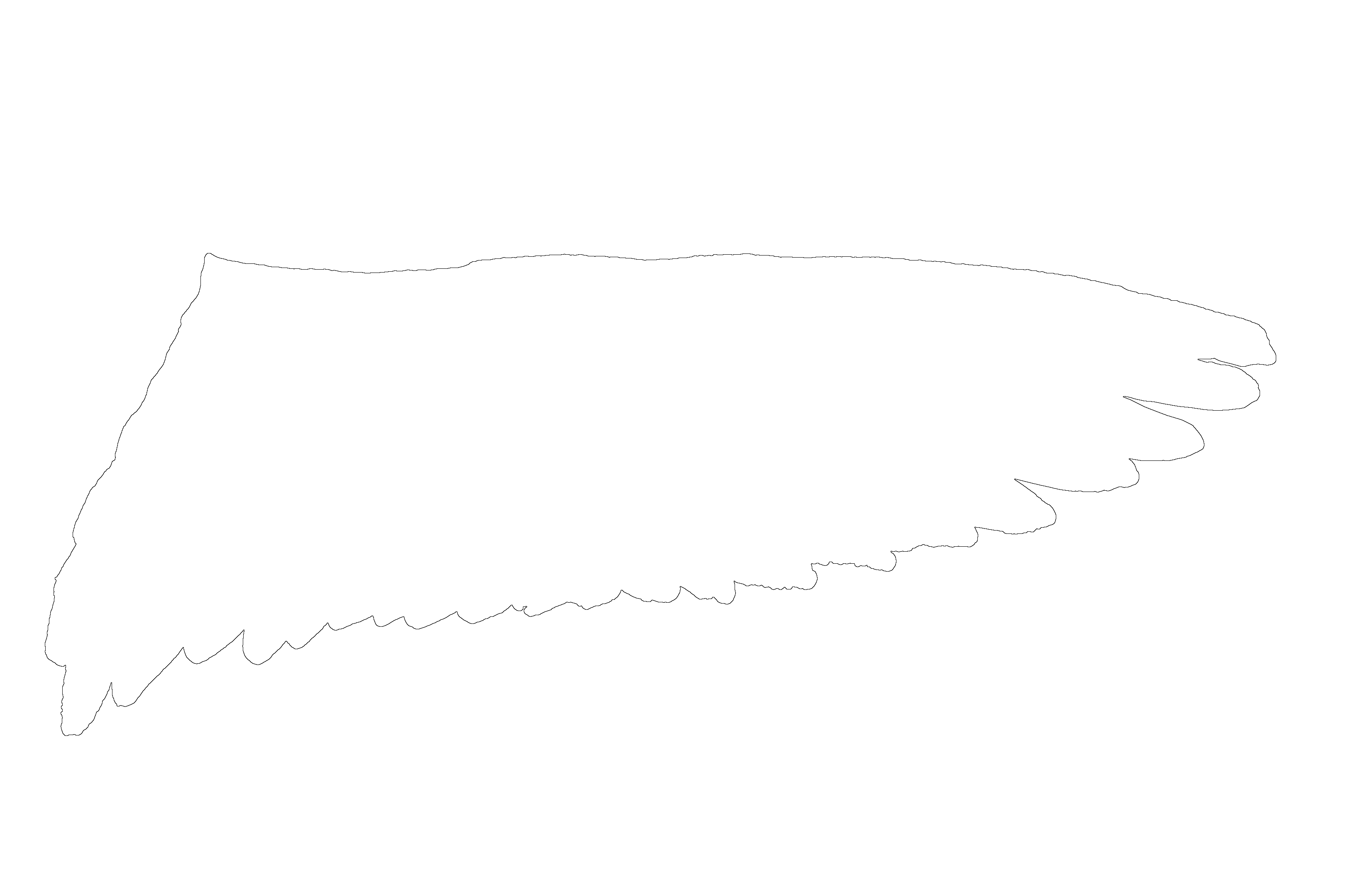

Supplement: Supplementary file 6 — Supplementary Data 4 [file 41467_2026_70692_MOESM6_ESM.zip › Supplementary Data 4/Calidris_pusilla.tif]

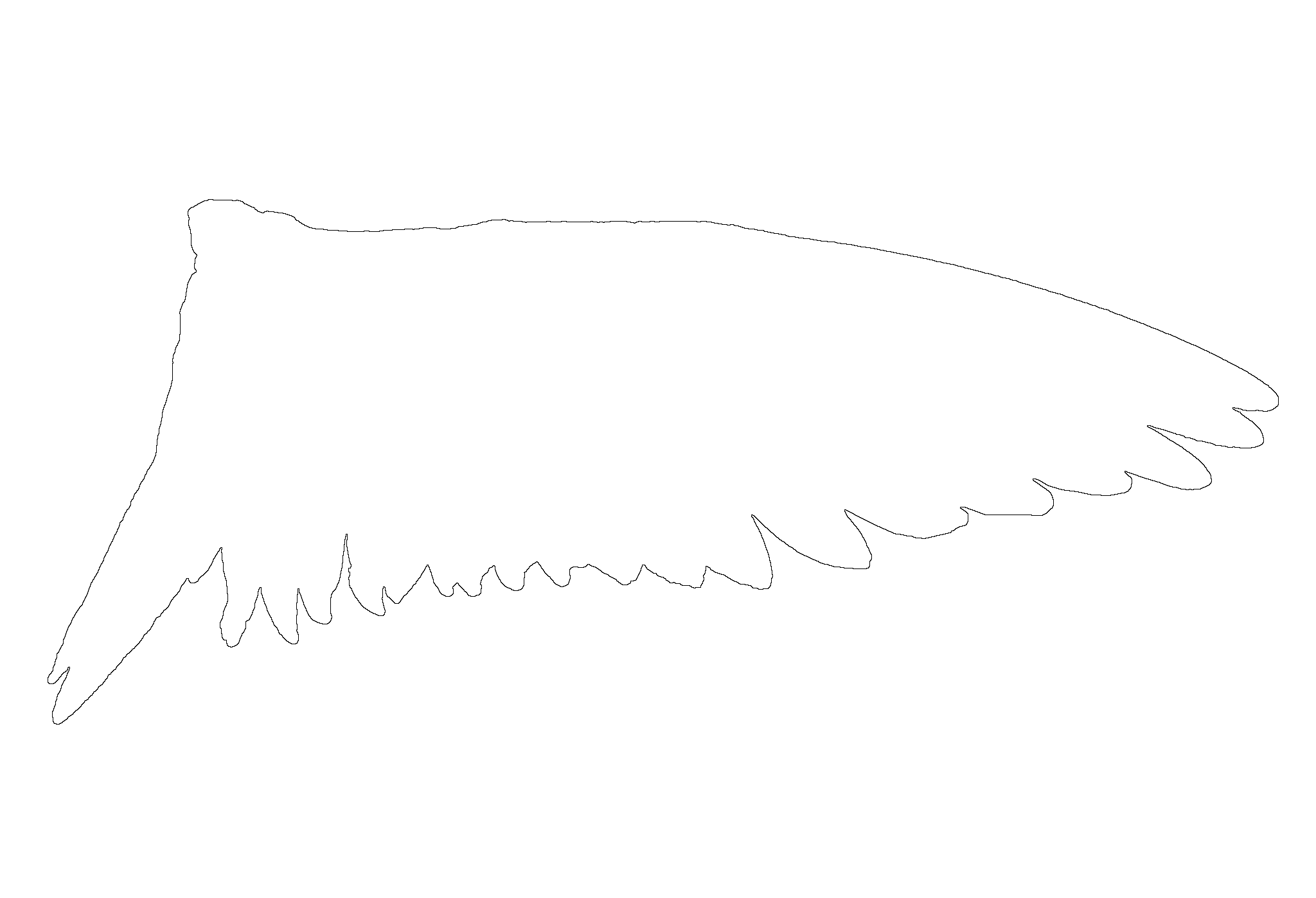

Supplement: Supplementary file 6 — Supplementary Data 4 [file 41467_2026_70692_MOESM6_ESM.zip › Supplementary Data 4/Calidris_ruficollis.tif]

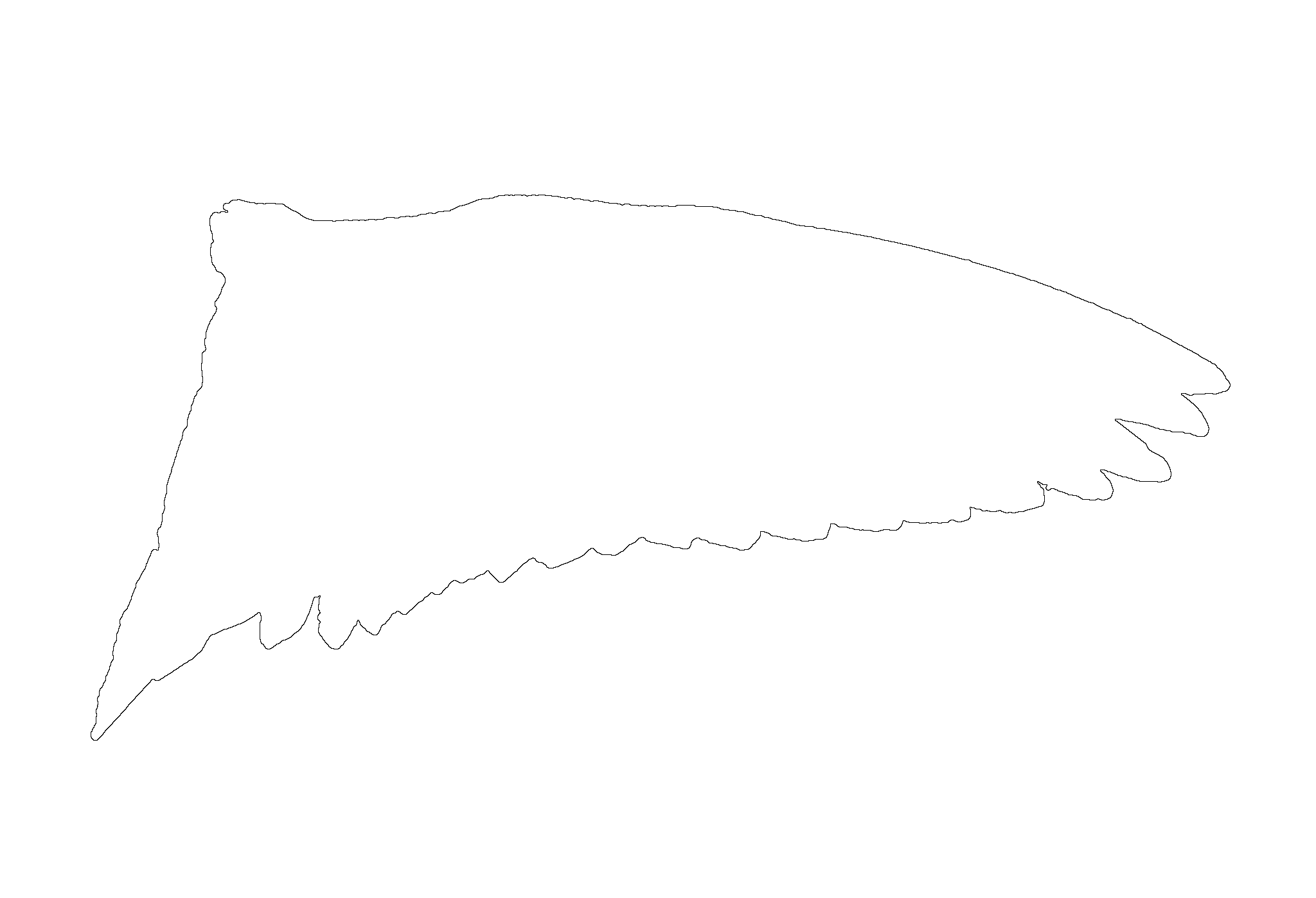

Supplement: Supplementary file 6 — Supplementary Data 4 [file 41467_2026_70692_MOESM6_ESM.zip › Supplementary Data 4/Calidris_subminuta.tif]

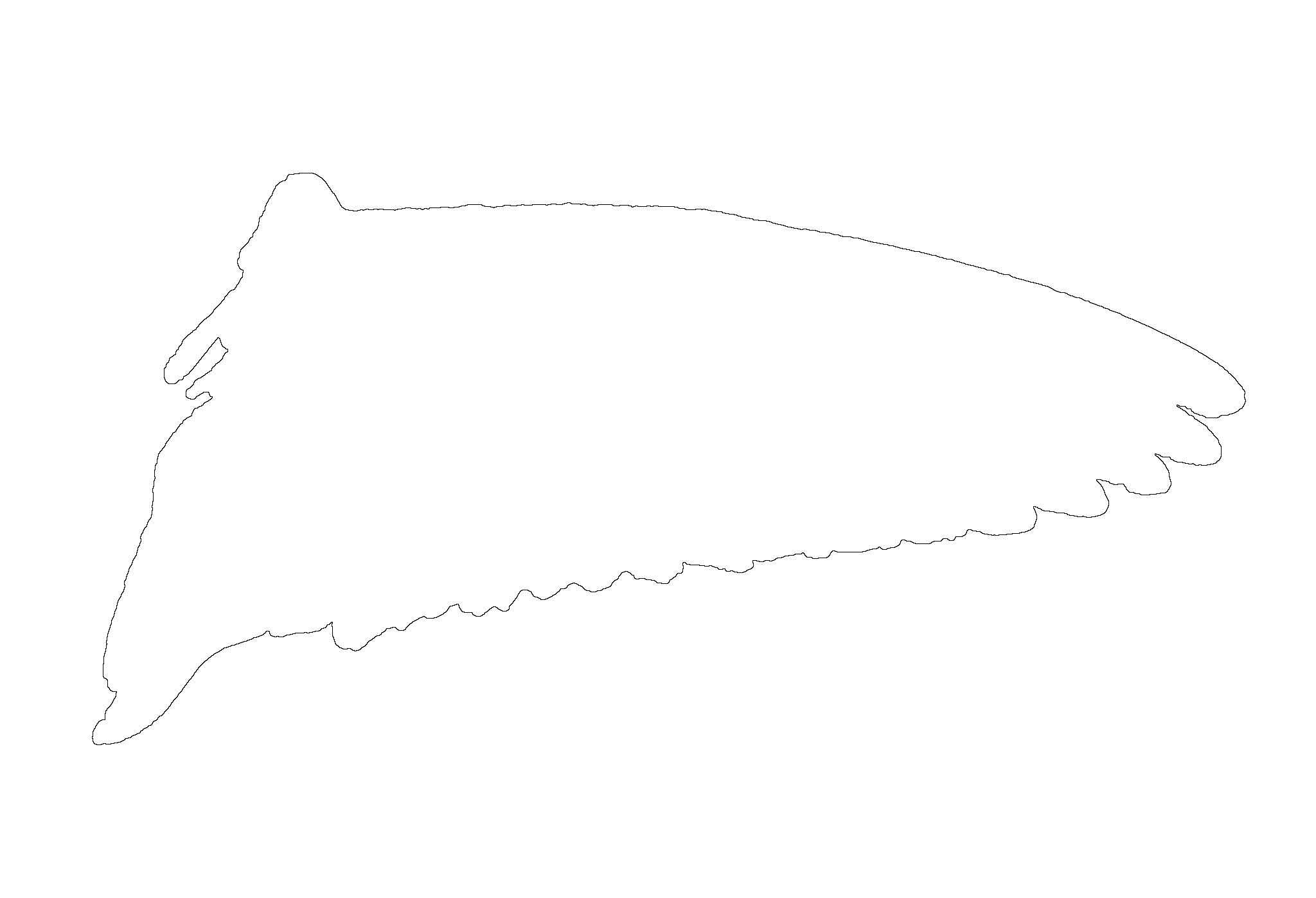

Supplement: Supplementary file 6 — Supplementary Data 4 [file 41467_2026_70692_MOESM6_ESM.zip › Supplementary Data 4/Calidris_temminckii.tif]

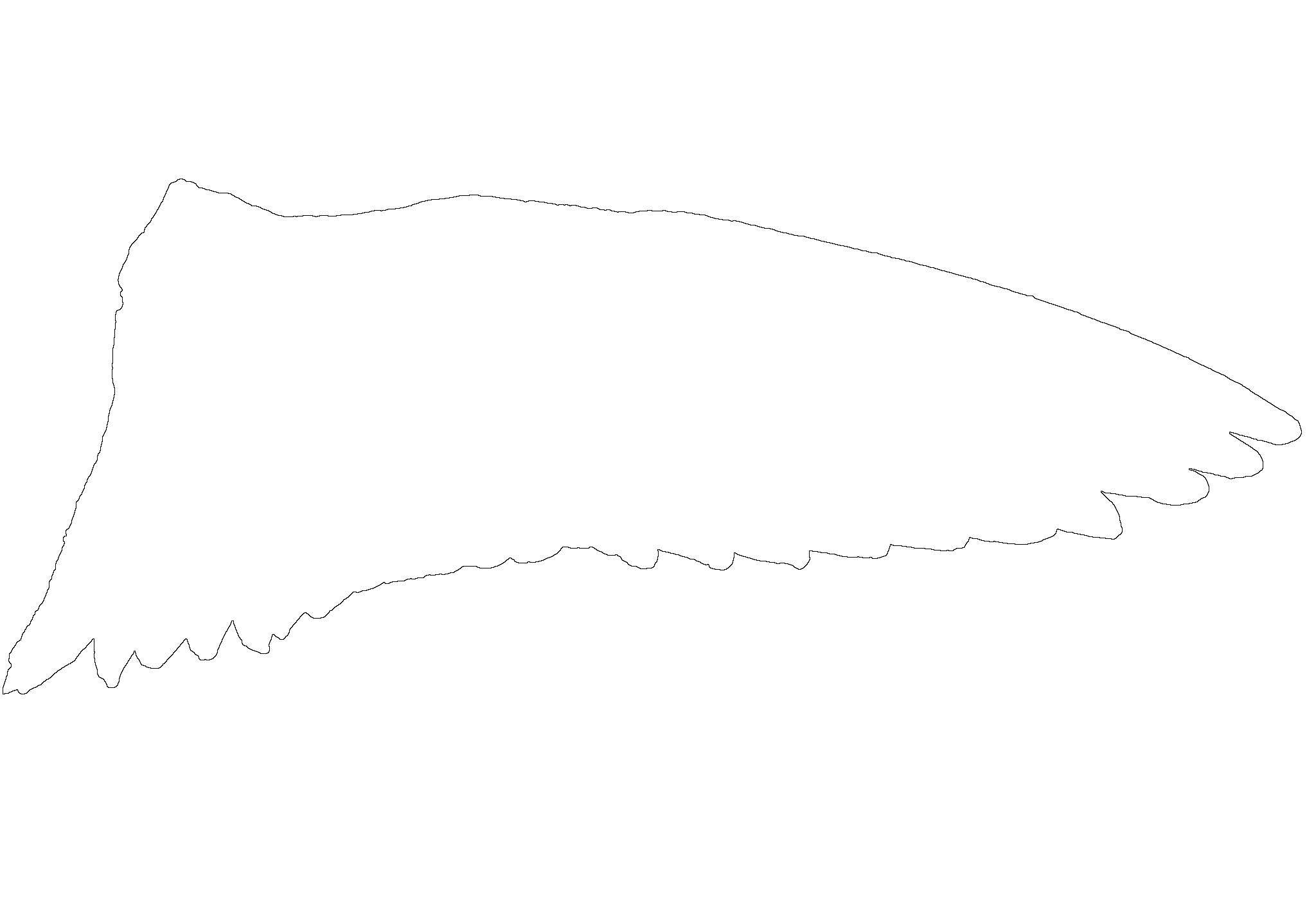

Supplement: Supplementary file 6 — Supplementary Data 4 [file 41467_2026_70692_MOESM6_ESM.zip › Supplementary Data 4/Calidris_tenuirostris.tif]

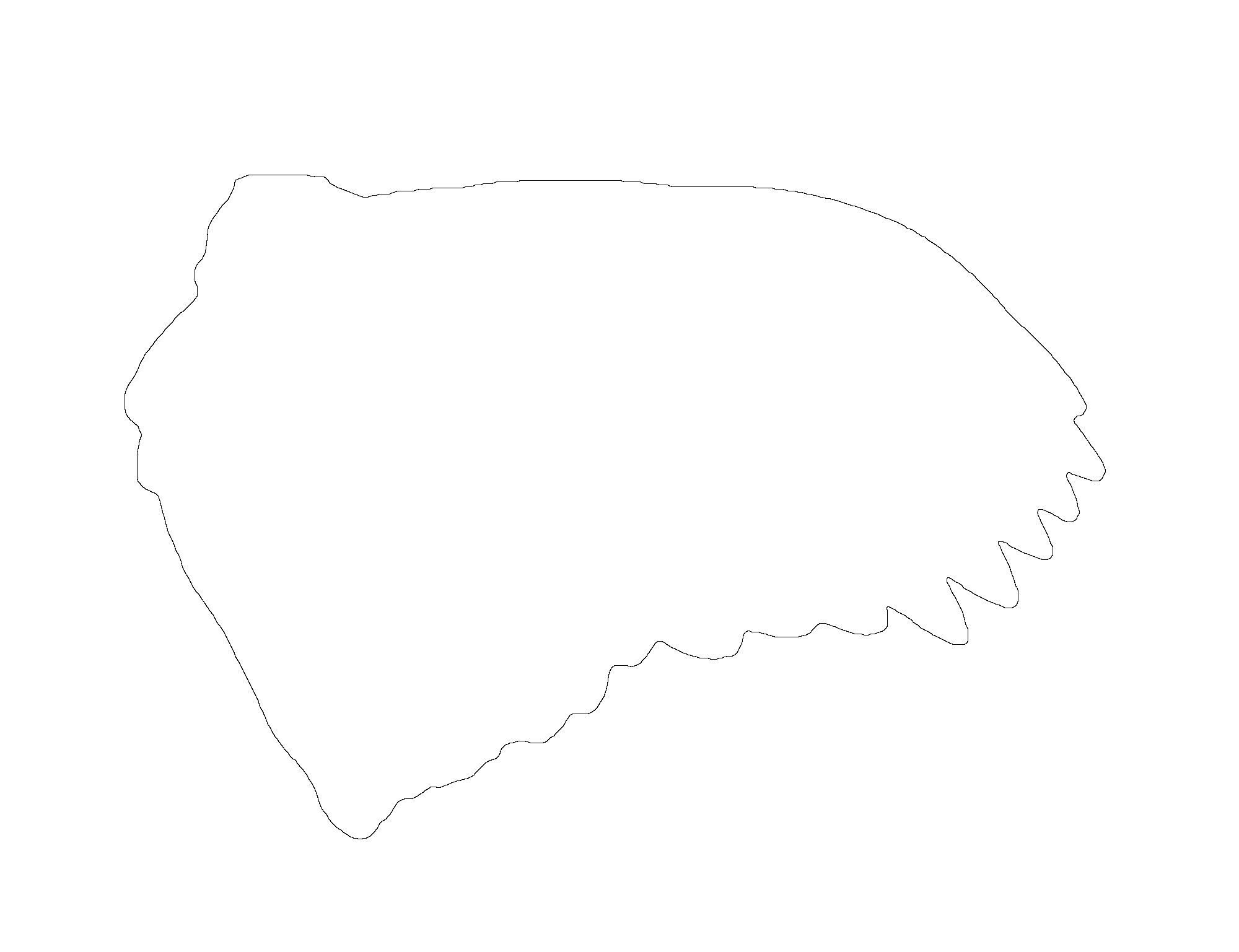

Supplement: Supplementary file 6 — Supplementary Data 4 [file 41467_2026_70692_MOESM6_ESM.zip › Supplementary Data 4/Callipepla_californica.tif]

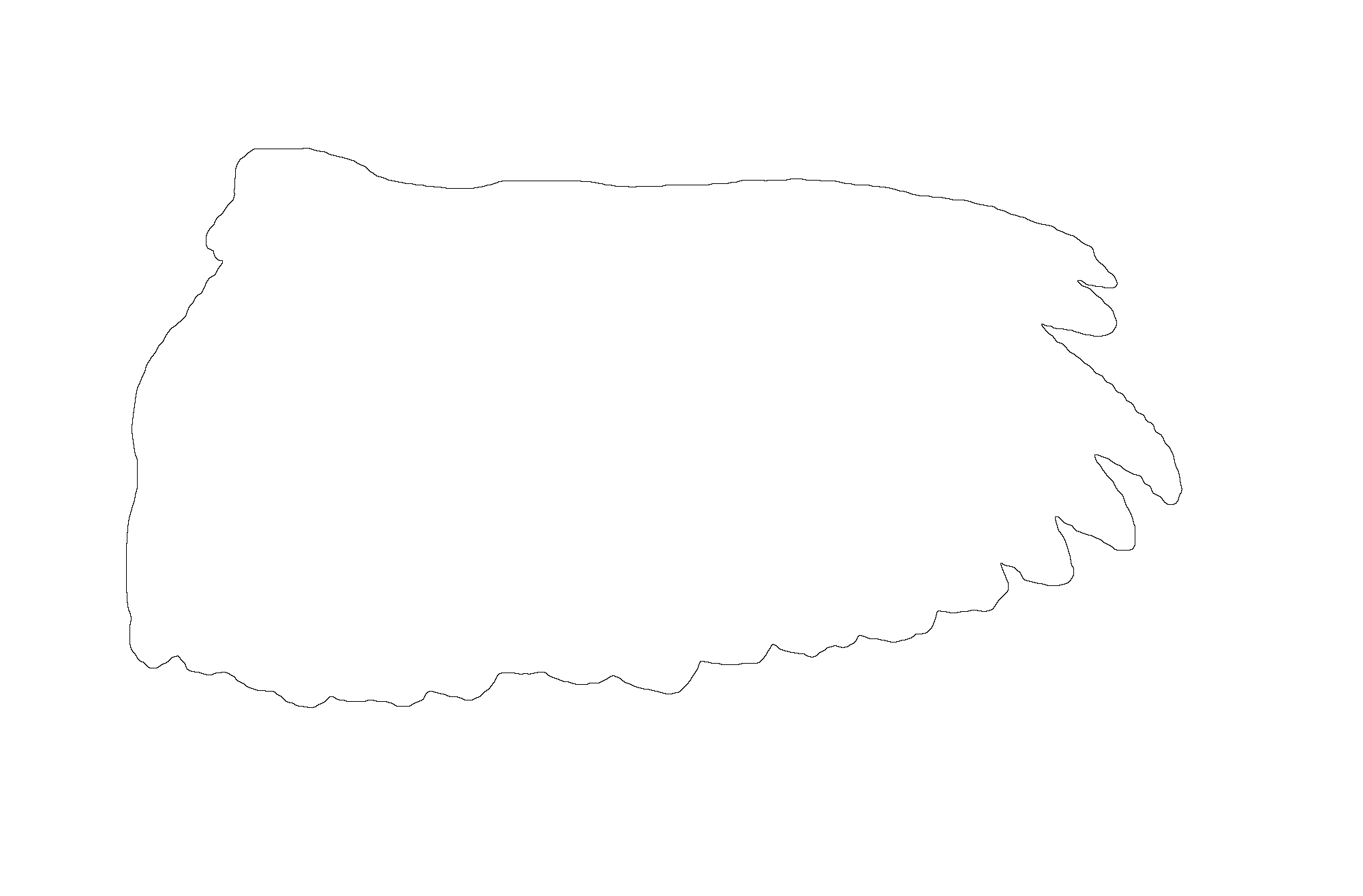

Supplement: Supplementary file 6 — Supplementary Data 4 [file 41467_2026_70692_MOESM6_ESM.zip › Supplementary Data 4/Caloenas_nicobarica.tif]

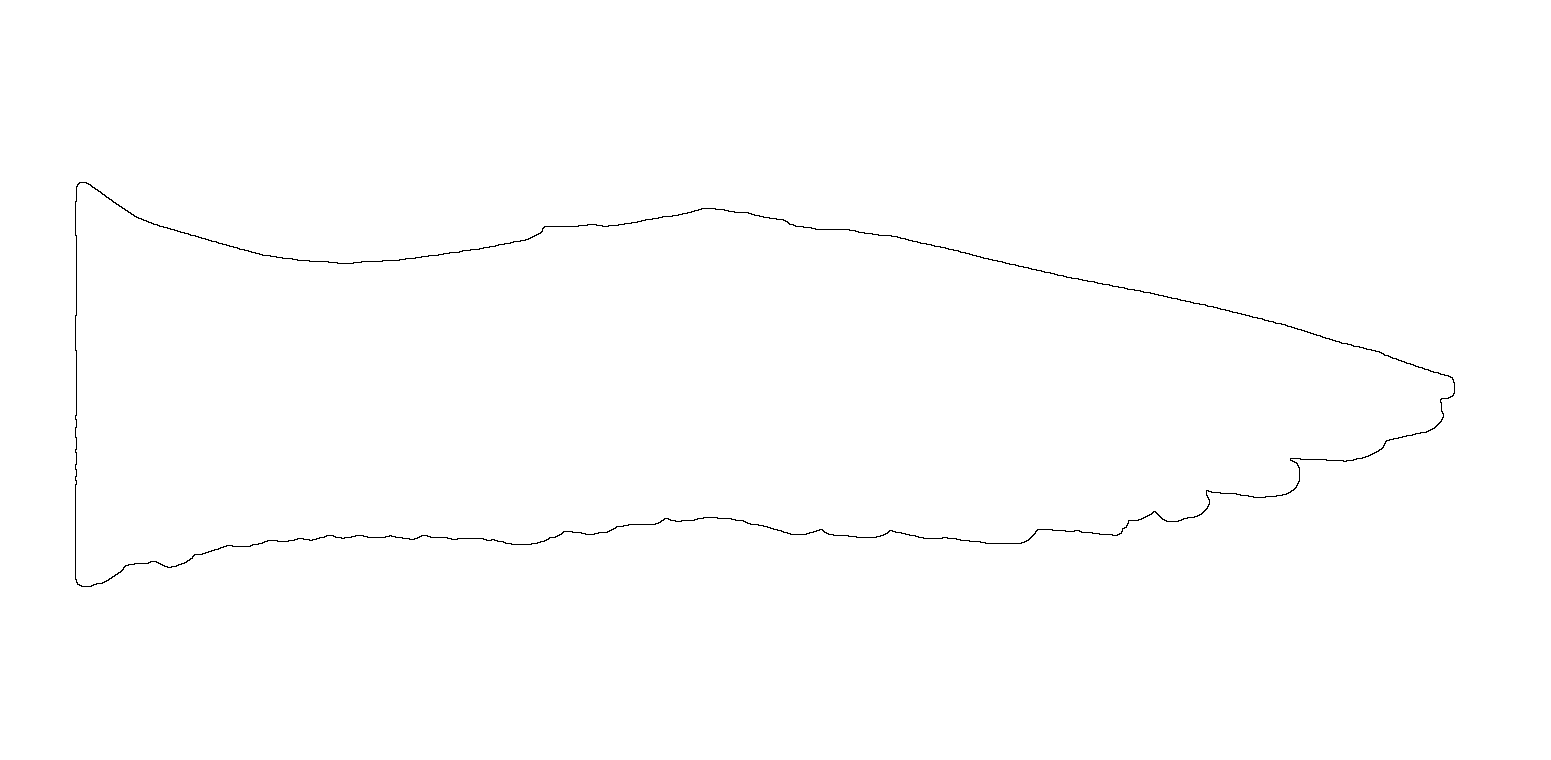

Supplement: Supplementary file 6 — Supplementary Data 4 [file 41467_2026_70692_MOESM6_ESM.zip › Supplementary Data 4/Calonectris_diomedea.tif]

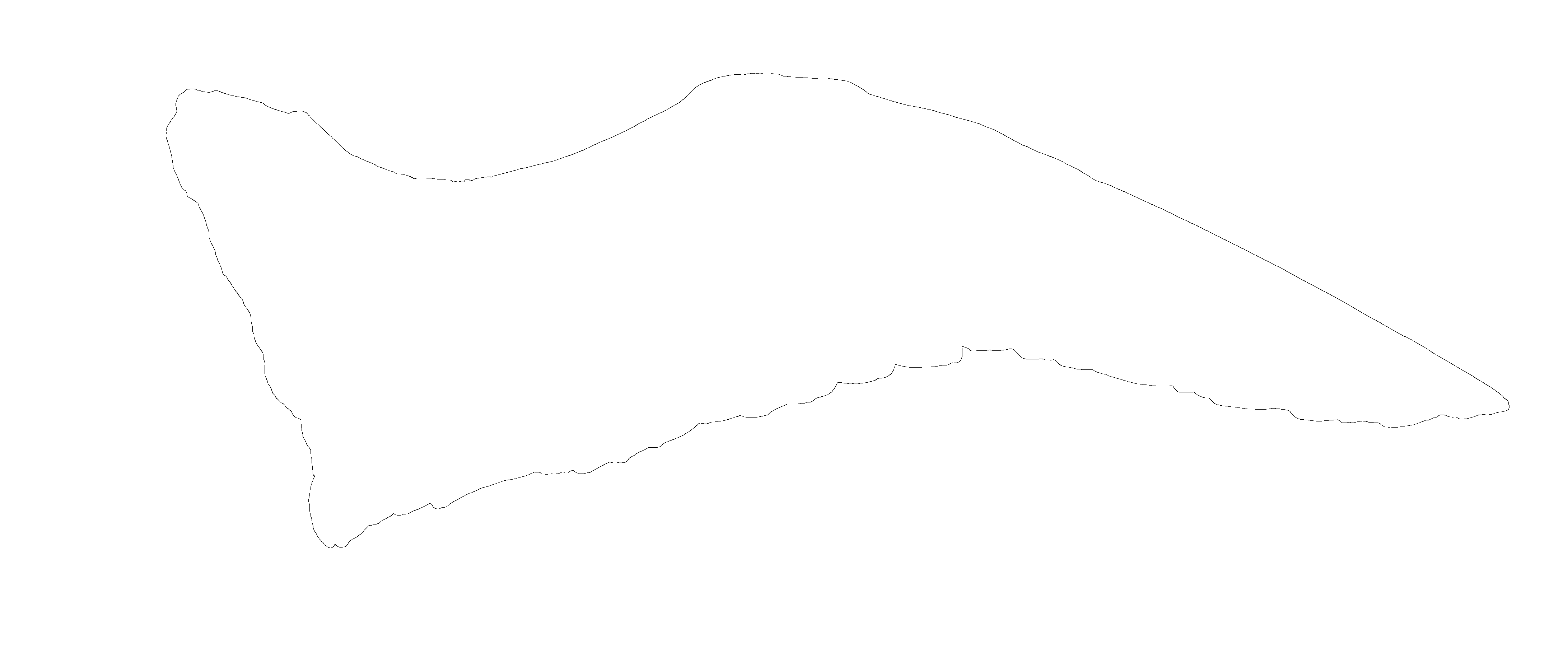

Supplement: Supplementary file 6 — Supplementary Data 4 [file 41467_2026_70692_MOESM6_ESM.zip › Supplementary Data 4/Calonectris_leucomelas.tif]

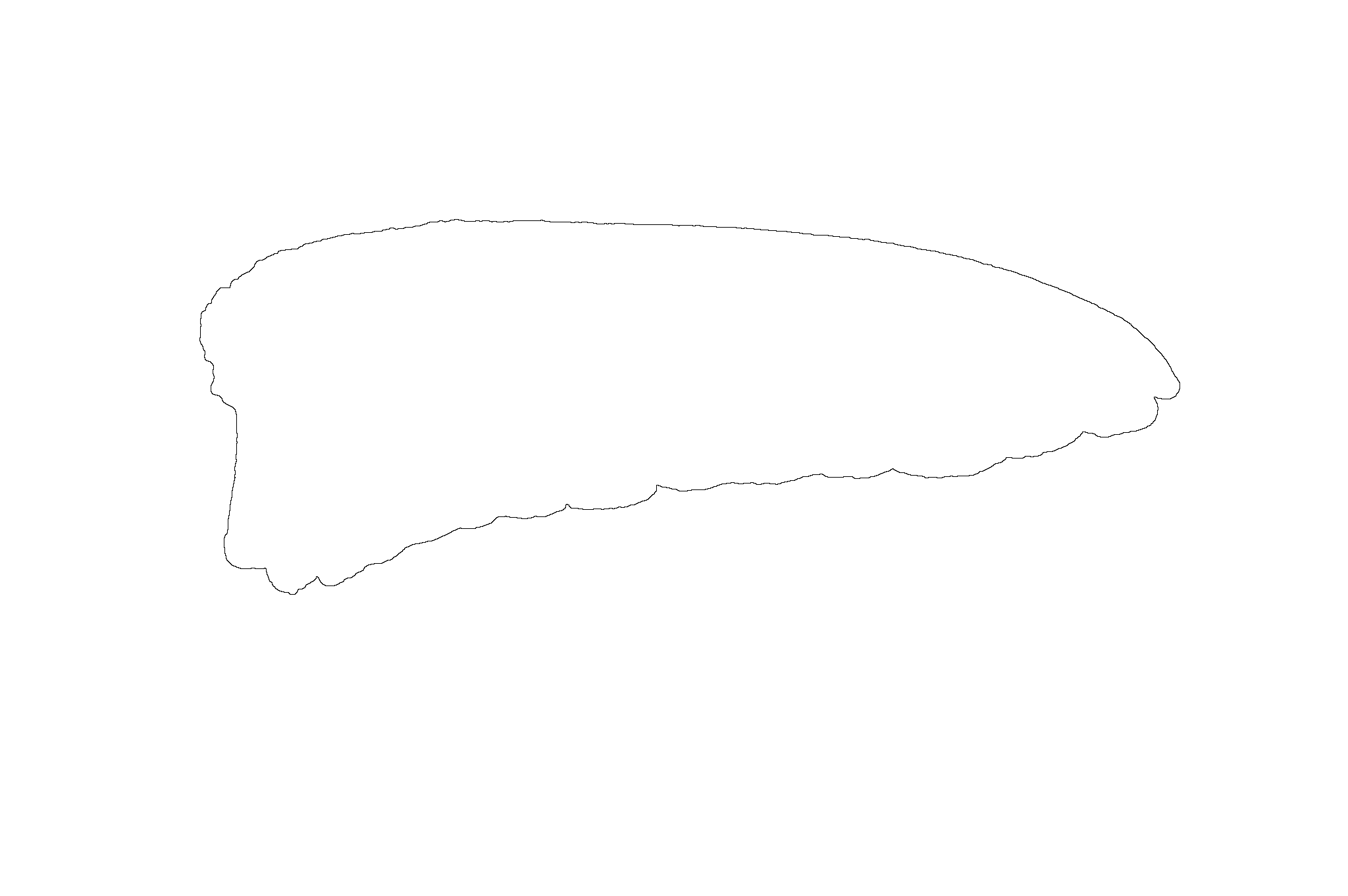

Supplement: Supplementary file 6 — Supplementary Data 4 [file 41467_2026_70692_MOESM6_ESM.zip › Supplementary Data 4/Calypte_anna.tif]

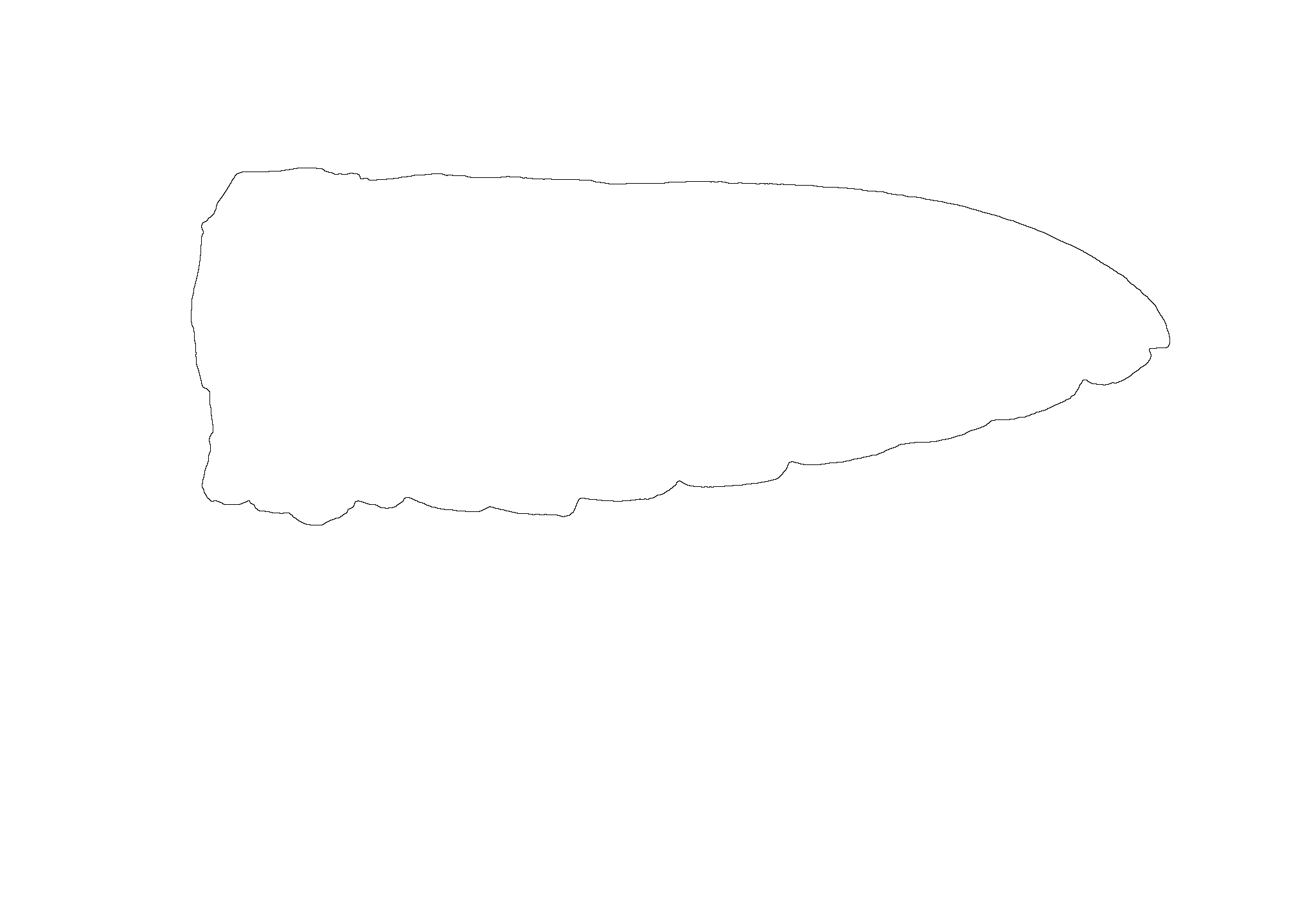

Supplement: Supplementary file 6 — Supplementary Data 4 [file 41467_2026_70692_MOESM6_ESM.zip › Supplementary Data 4/Calypte_costae.tif]
