# Supplementary figures and images for: Theoretical morphospace reveals mixed optimisation of the avian wing planform for flight style (part 1 of 2)
Source: Nat Commun. 2026 Mar 31;17:3902. doi: 10.1038/s41467-026-70692-w (PMC13128903; doi:10.1038/s41467-026-70692-w)

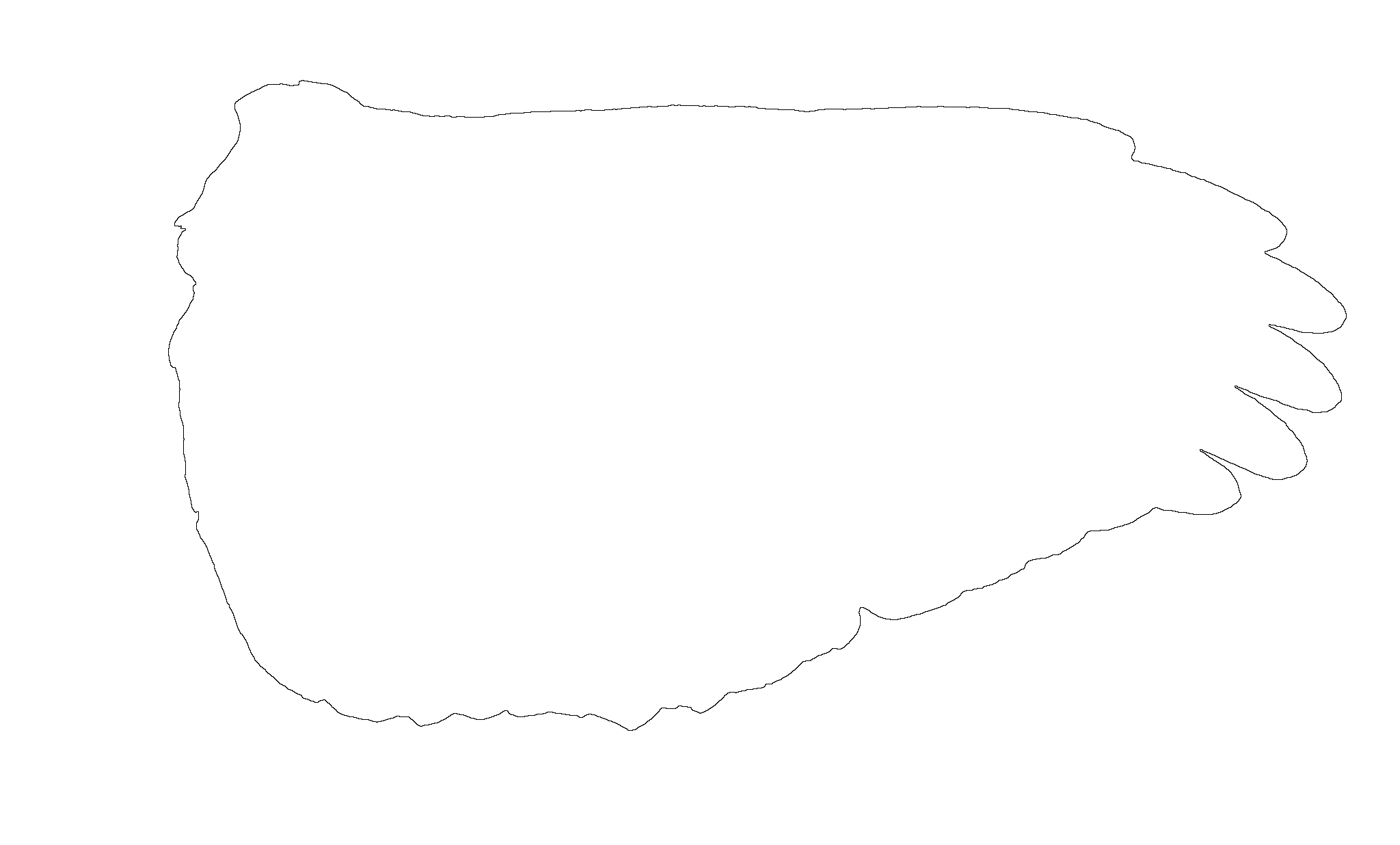

Supplement: Supplementary file 6 — Supplementary Data 4 [file 41467_2026_70692_MOESM6_ESM.zip › Supplementary Data 4/Accipiter_cooperii.tif]

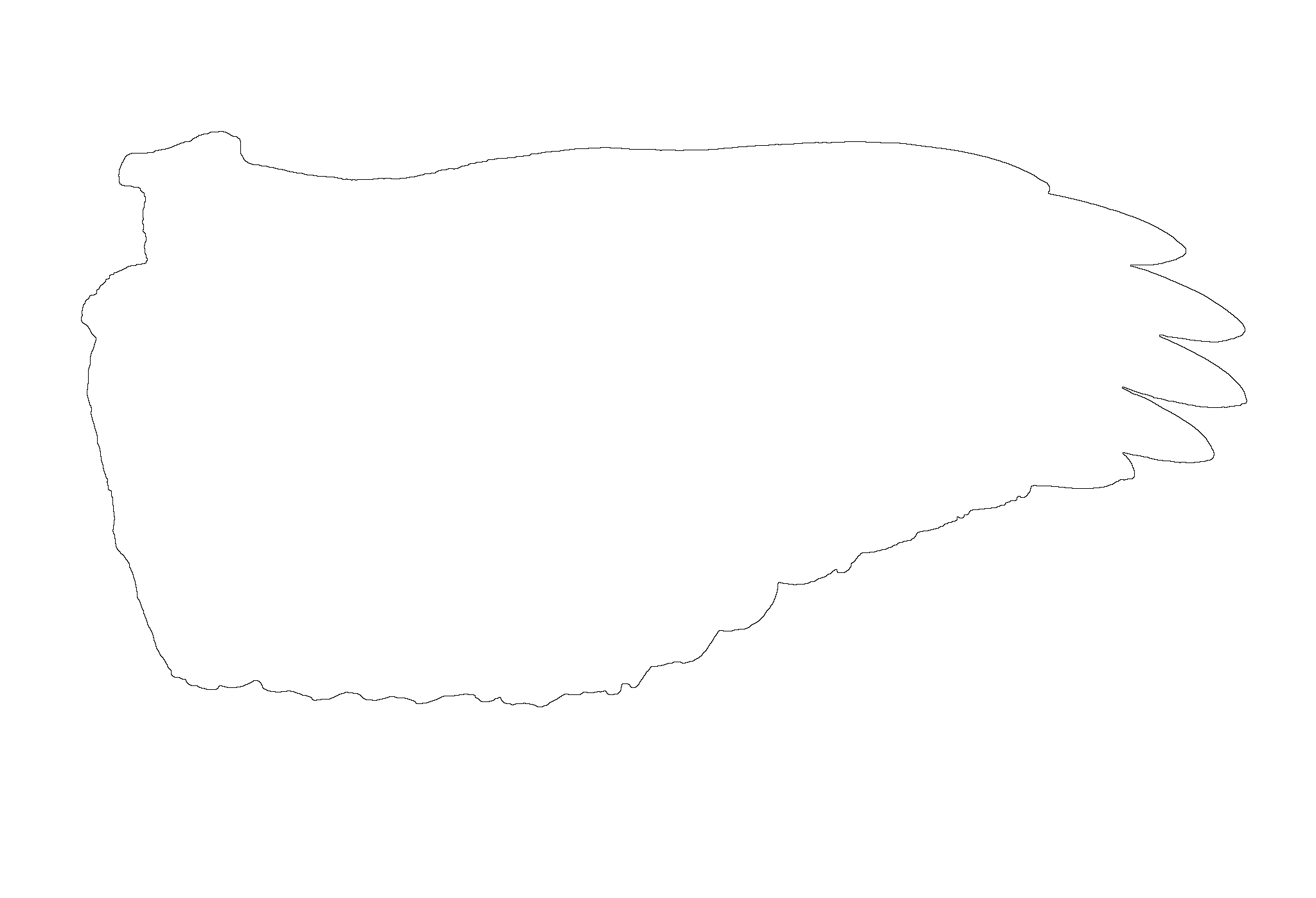

Supplement: Supplementary file 6 — Supplementary Data 4 [file 41467_2026_70692_MOESM6_ESM.zip › Supplementary Data 4/Accipiter_gentilis.tif]

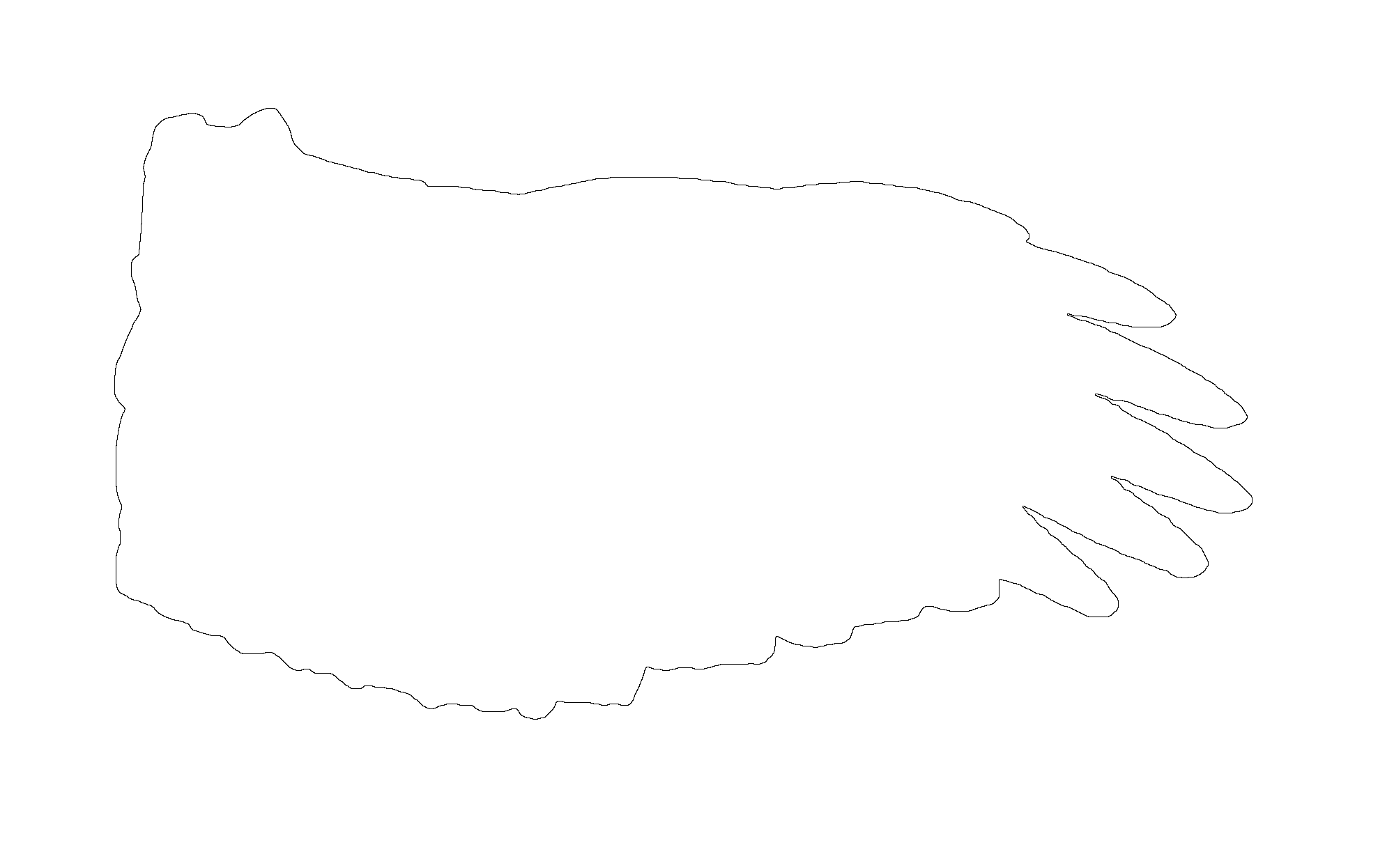

Supplement: Supplementary file 6 — Supplementary Data 4 [file 41467_2026_70692_MOESM6_ESM.zip › Supplementary Data 4/Accipiter_nisus.tif]

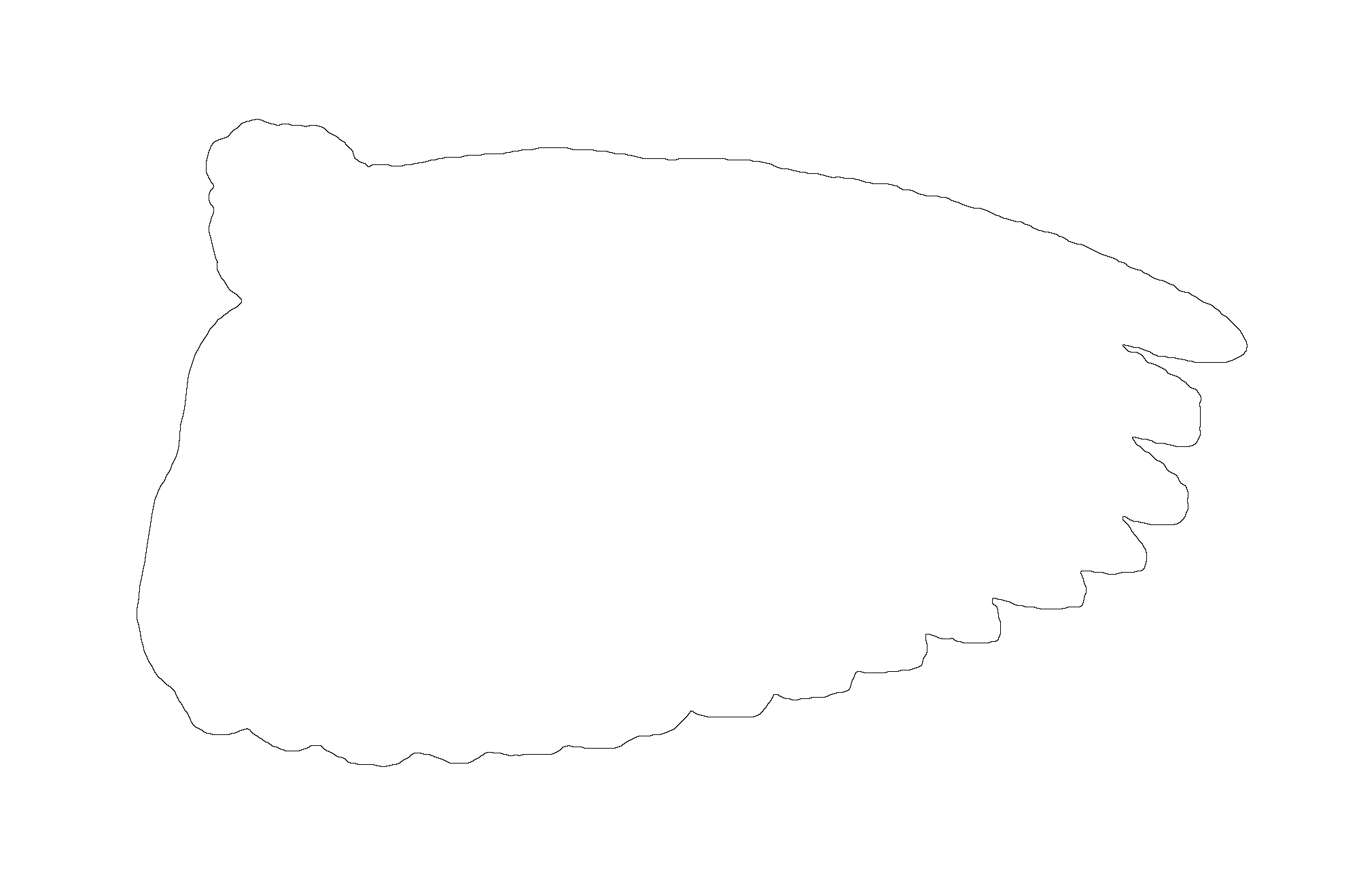

Supplement: Supplementary file 6 — Supplementary Data 4 [file 41467_2026_70692_MOESM6_ESM.zip › Supplementary Data 4/Acridotheres_tristis.tif]

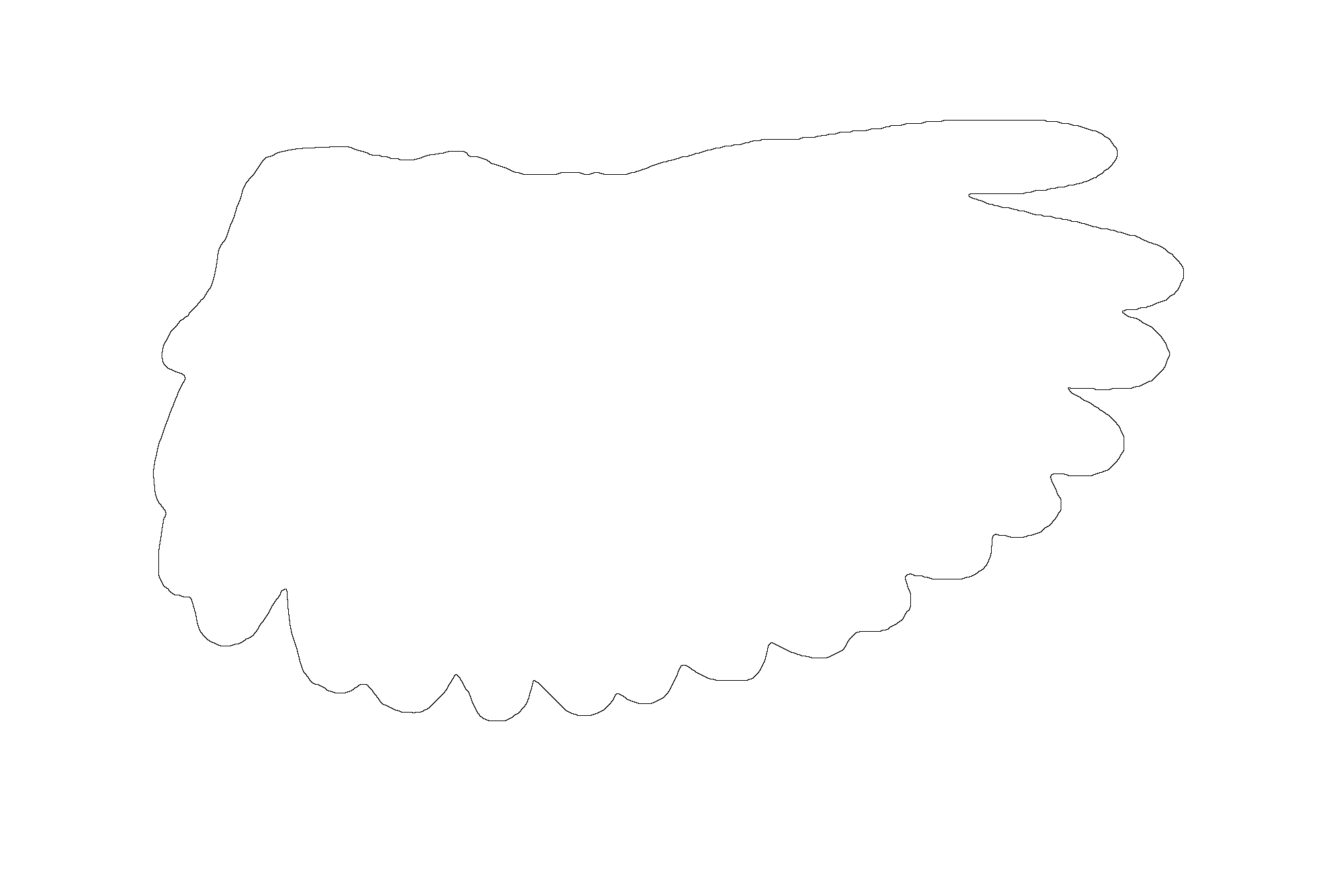

Supplement: Supplementary file 6 — Supplementary Data 4 [file 41467_2026_70692_MOESM6_ESM.zip › Supplementary Data 4/Acrocephalus_melanopogon.tif]

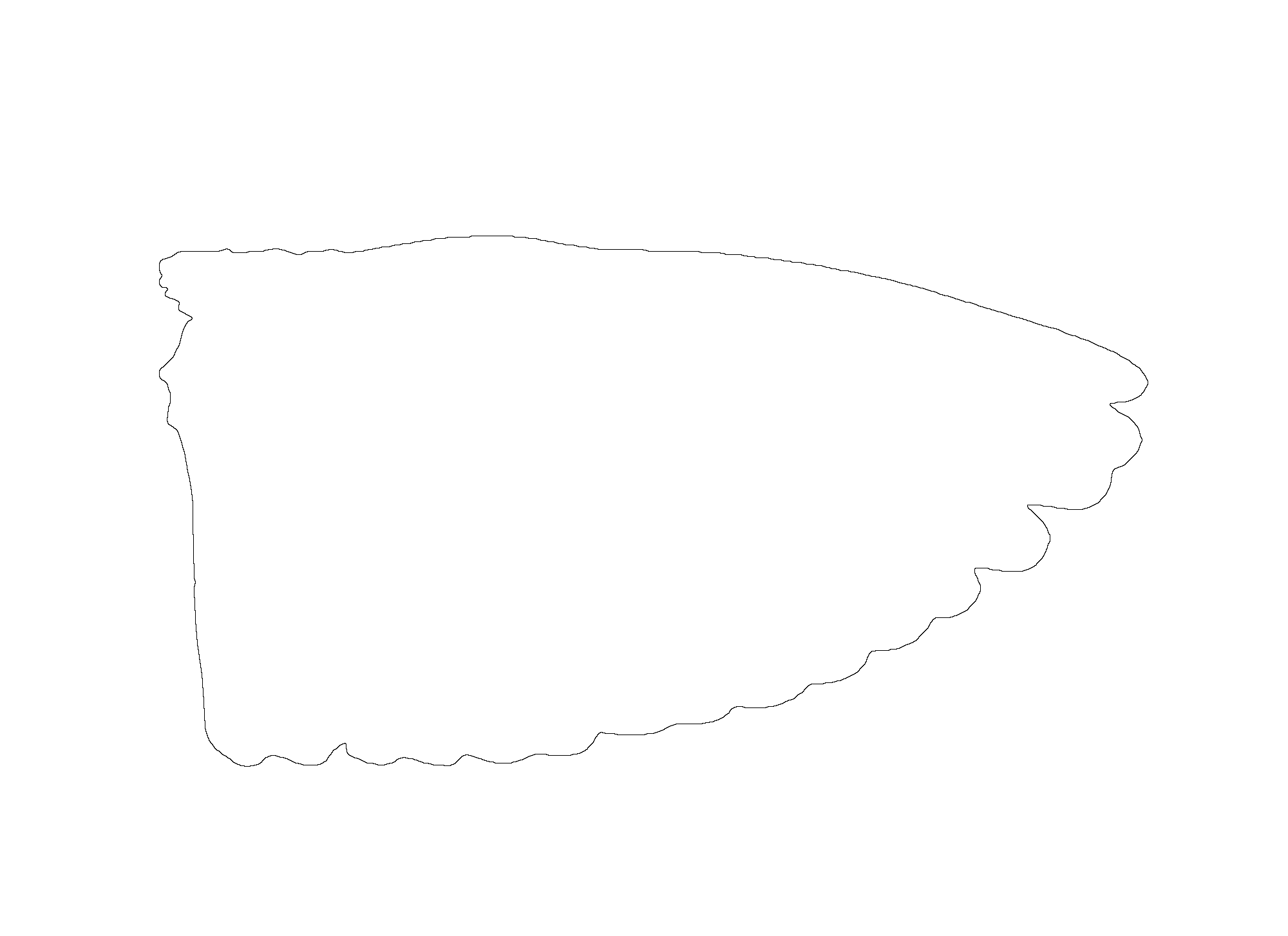

Supplement: Supplementary file 6 — Supplementary Data 4 [file 41467_2026_70692_MOESM6_ESM.zip › Supplementary Data 4/Acrocephalus_palustris.tif]

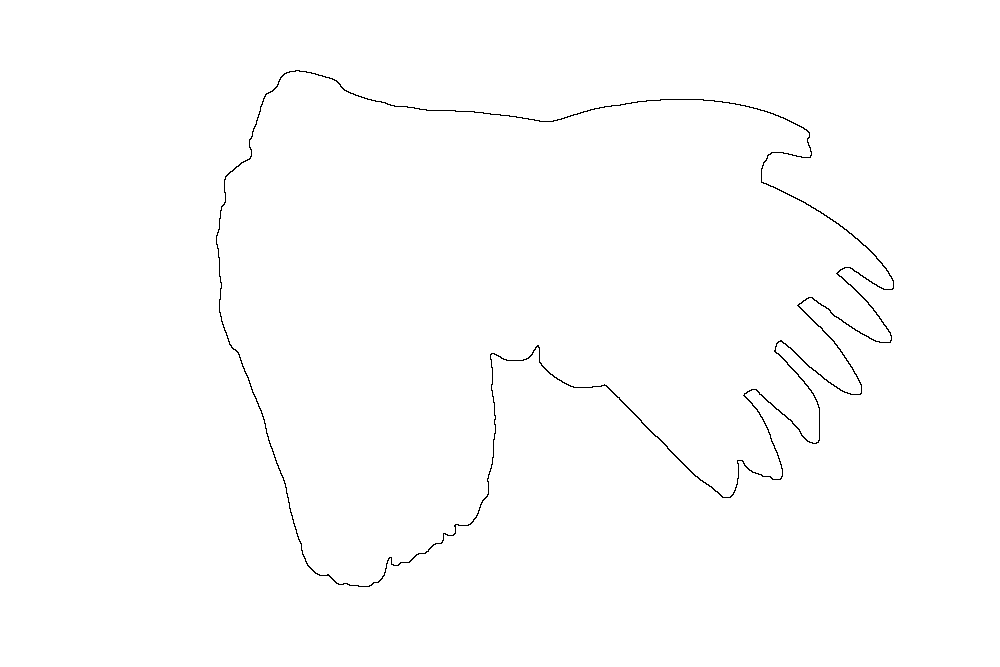

Supplement: Supplementary file 6 — Supplementary Data 4 [file 41467_2026_70692_MOESM6_ESM.zip › Supplementary Data 4/Acryllium_vulturinum.tif]

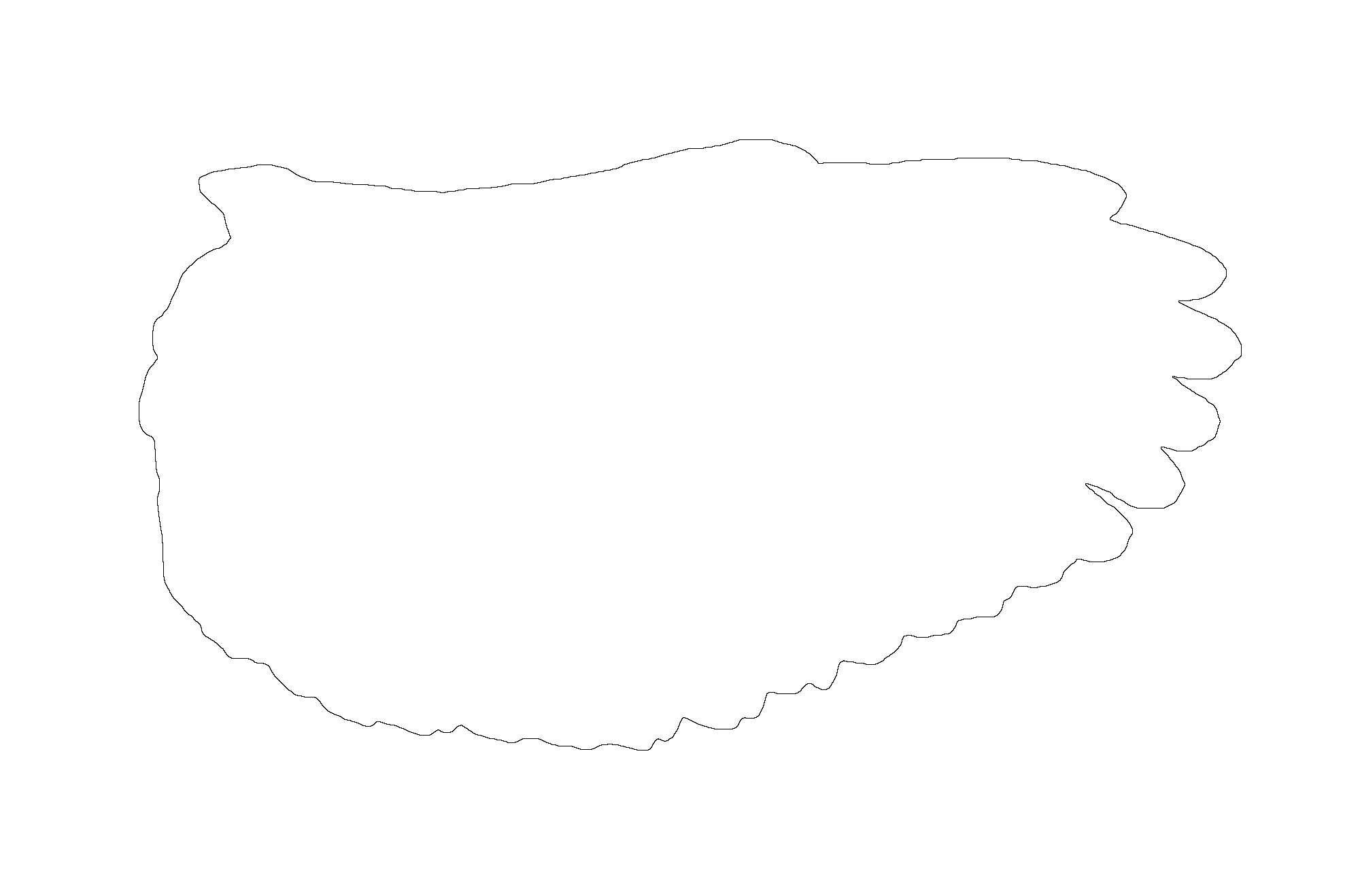

Supplement: Supplementary file 6 — Supplementary Data 4 [file 41467_2026_70692_MOESM6_ESM.zip › Supplementary Data 4/Actenoides_concretus.tif]

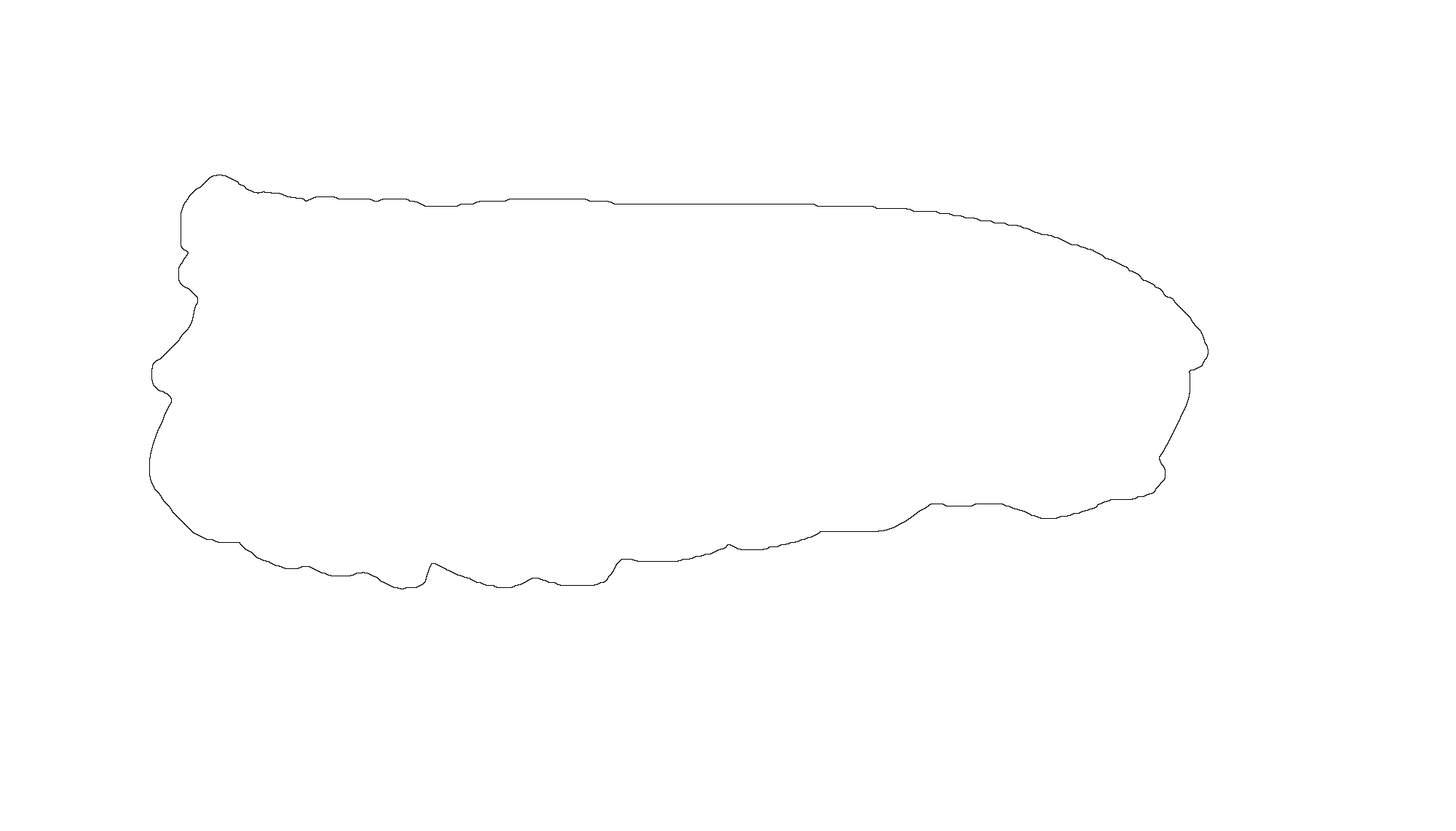

Supplement: Supplementary file 6 — Supplementary Data 4 [file 41467_2026_70692_MOESM6_ESM.zip › Supplementary Data 4/Adelomyia_melanogenys.tif]

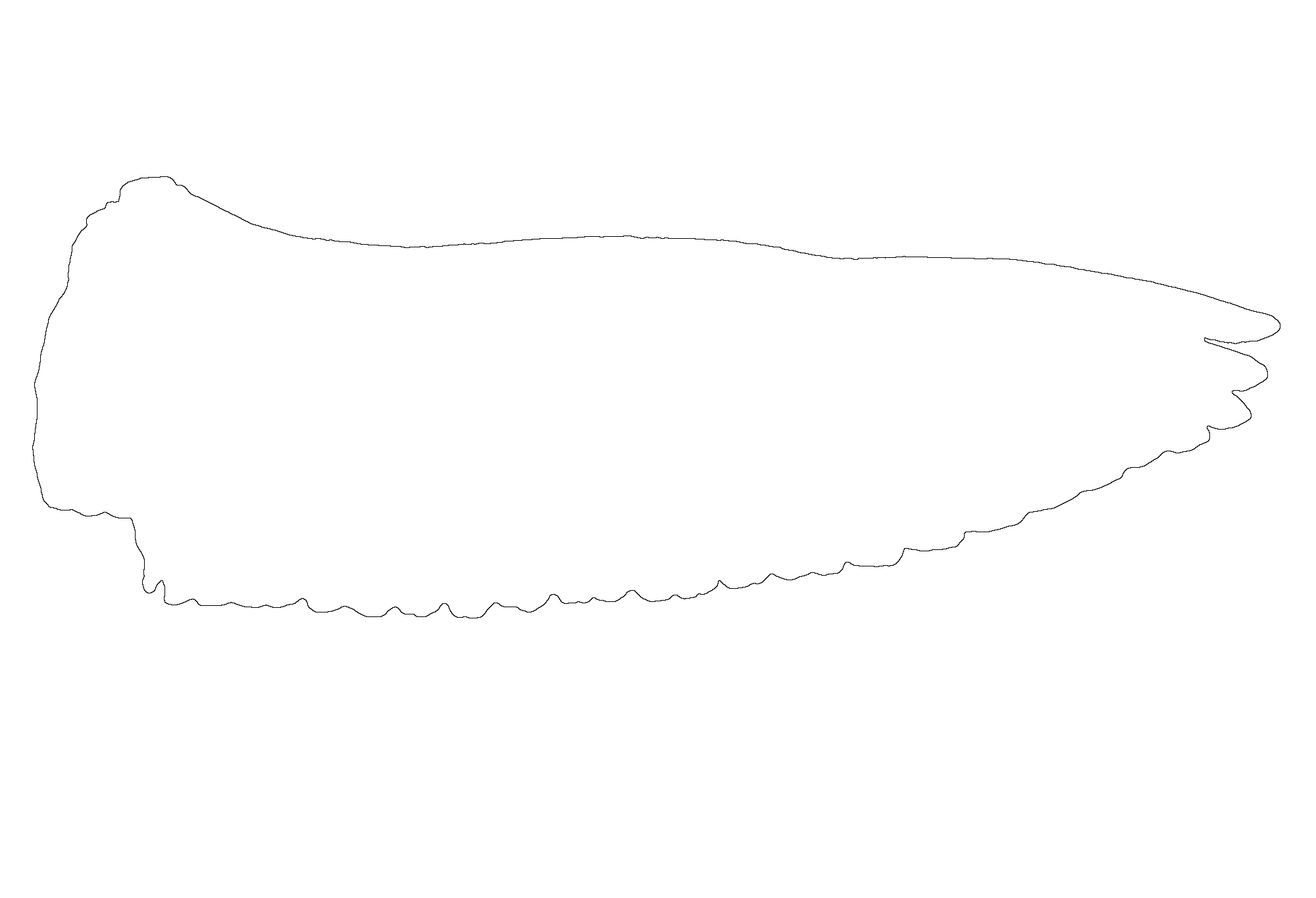

Supplement: Supplementary file 6 — Supplementary Data 4 [file 41467_2026_70692_MOESM6_ESM.zip › Supplementary Data 4/Aechmophorus_clarkii.tif]

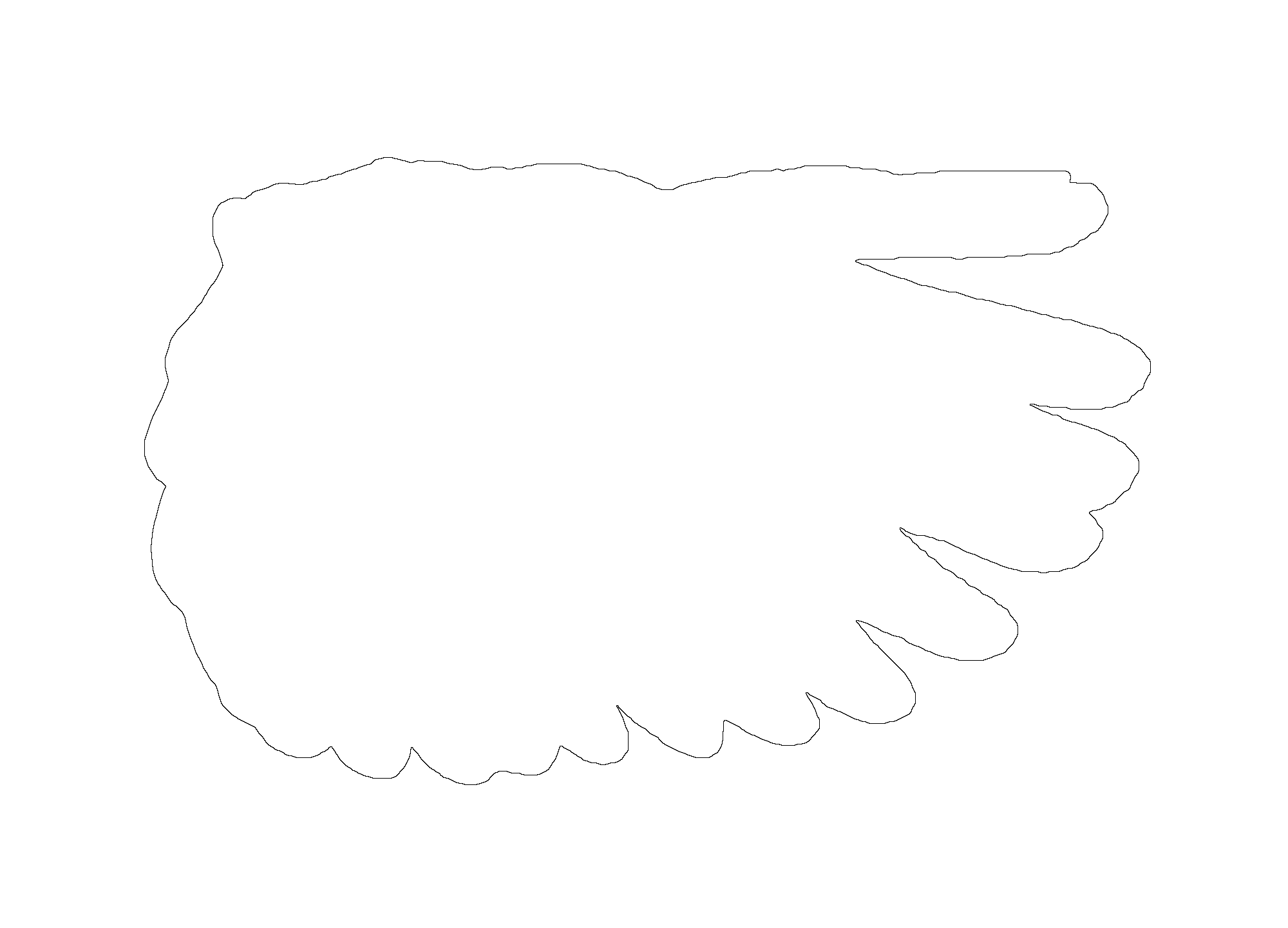

Supplement: Supplementary file 6 — Supplementary Data 4 [file 41467_2026_70692_MOESM6_ESM.zip › Supplementary Data 4/Aegithalos_caudatus.tif]

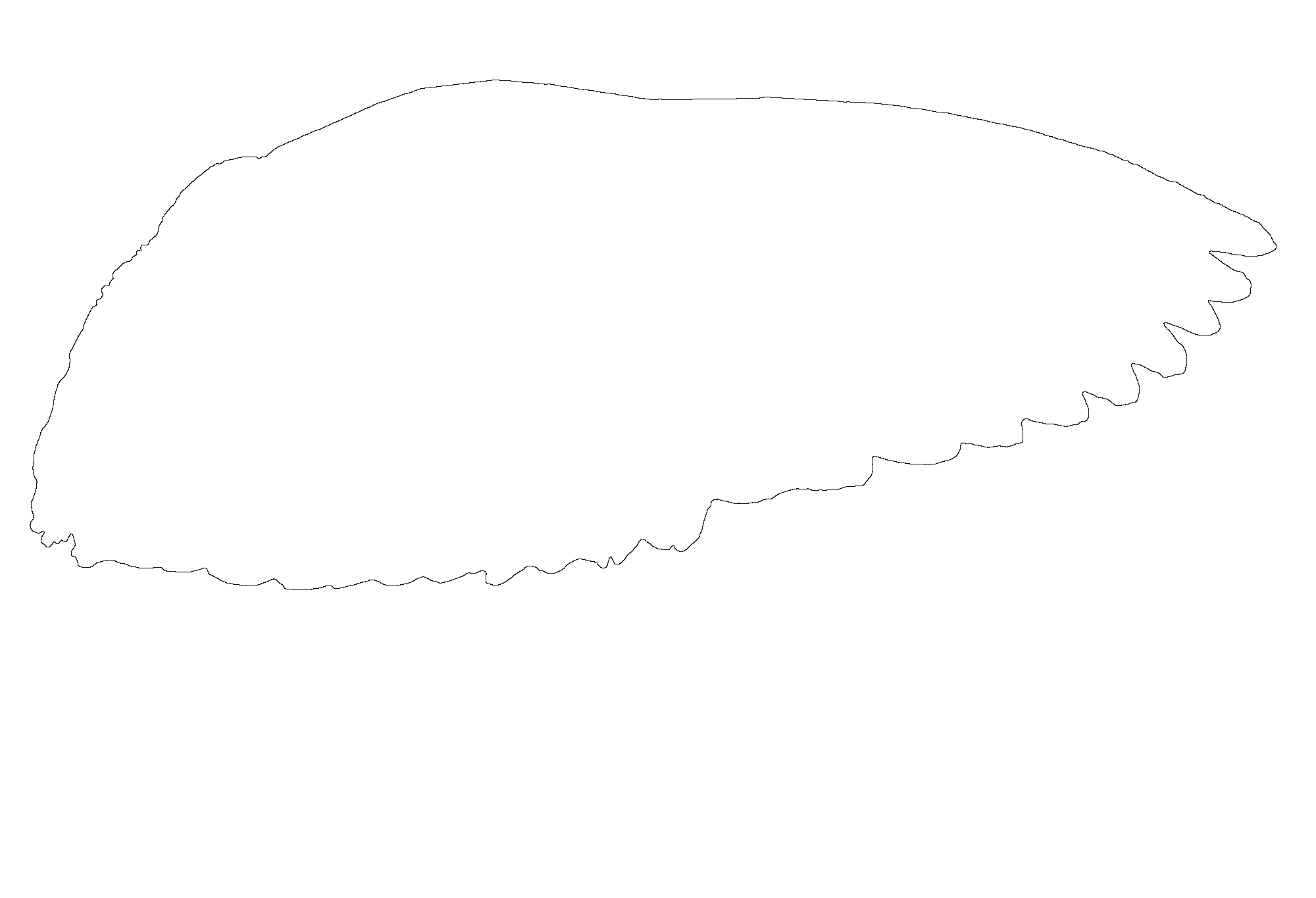

Supplement: Supplementary file 6 — Supplementary Data 4 [file 41467_2026_70692_MOESM6_ESM.zip › Supplementary Data 4/Aethia_psittacula.tif]

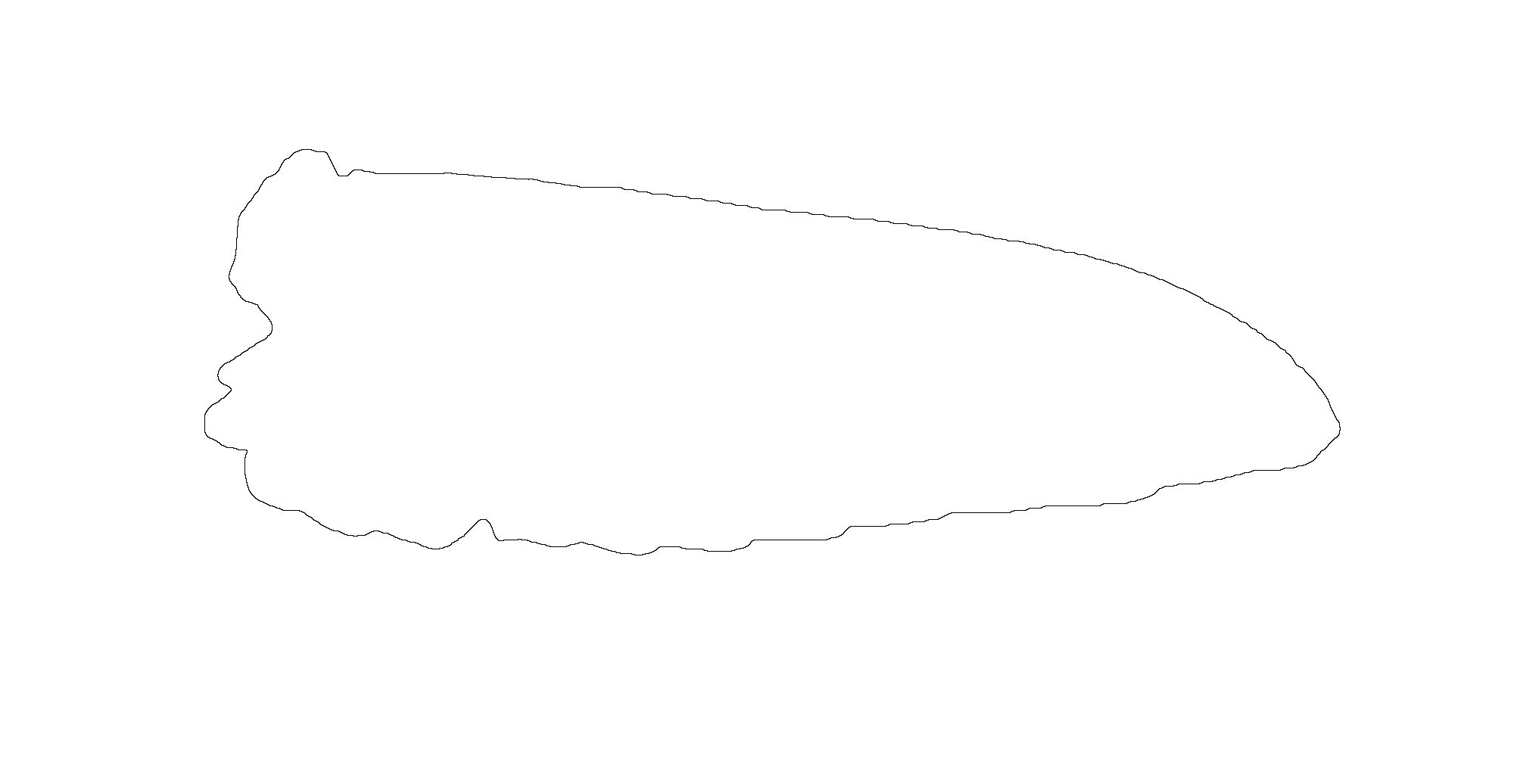

Supplement: Supplementary file 6 — Supplementary Data 4 [file 41467_2026_70692_MOESM6_ESM.zip › Supplementary Data 4/Aglaiocercus_kingi.tif]

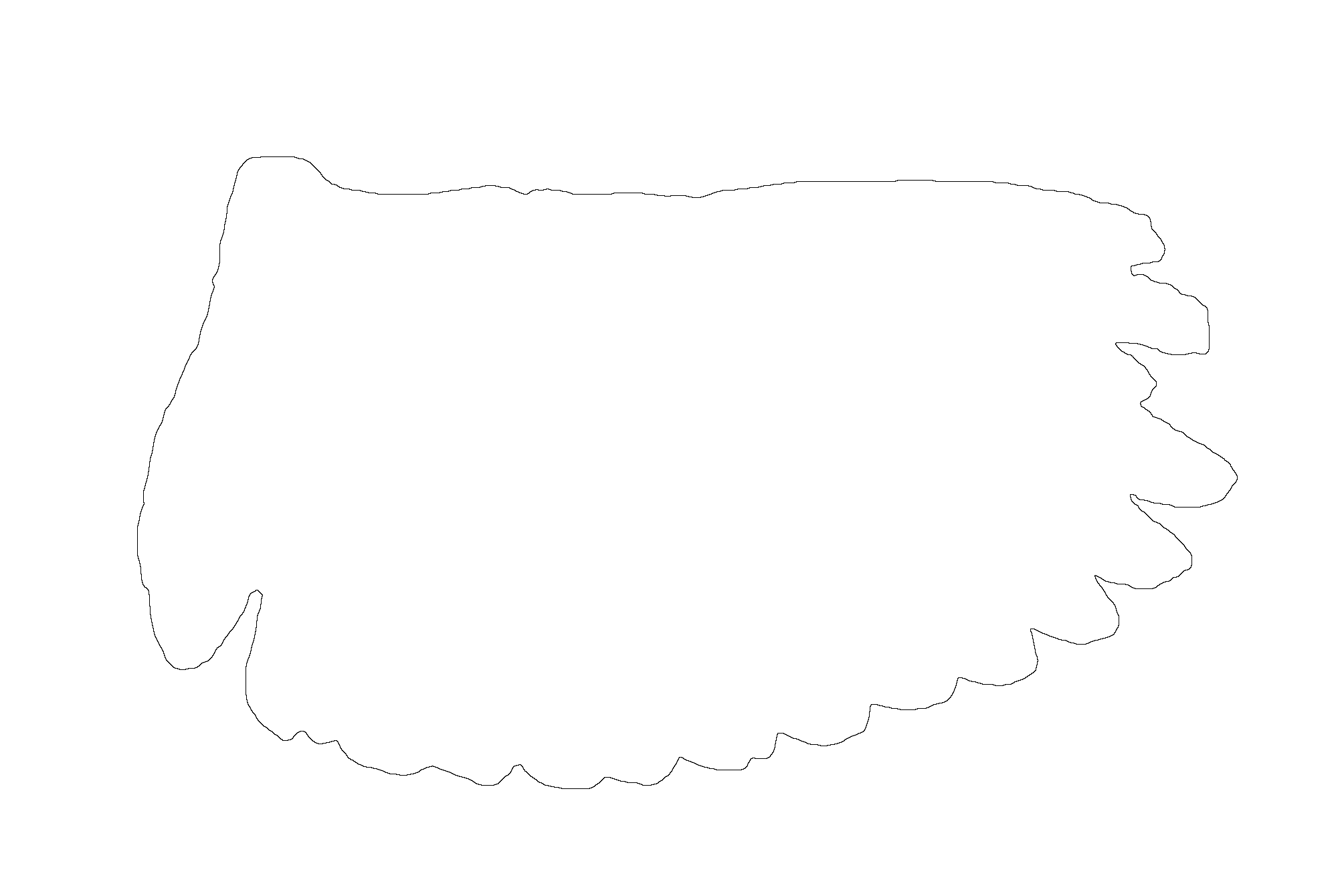

Supplement: Supplementary file 6 — Supplementary Data 4 [file 41467_2026_70692_MOESM6_ESM.zip › Supplementary Data 4/Ailuroedus_buccoides.tif]

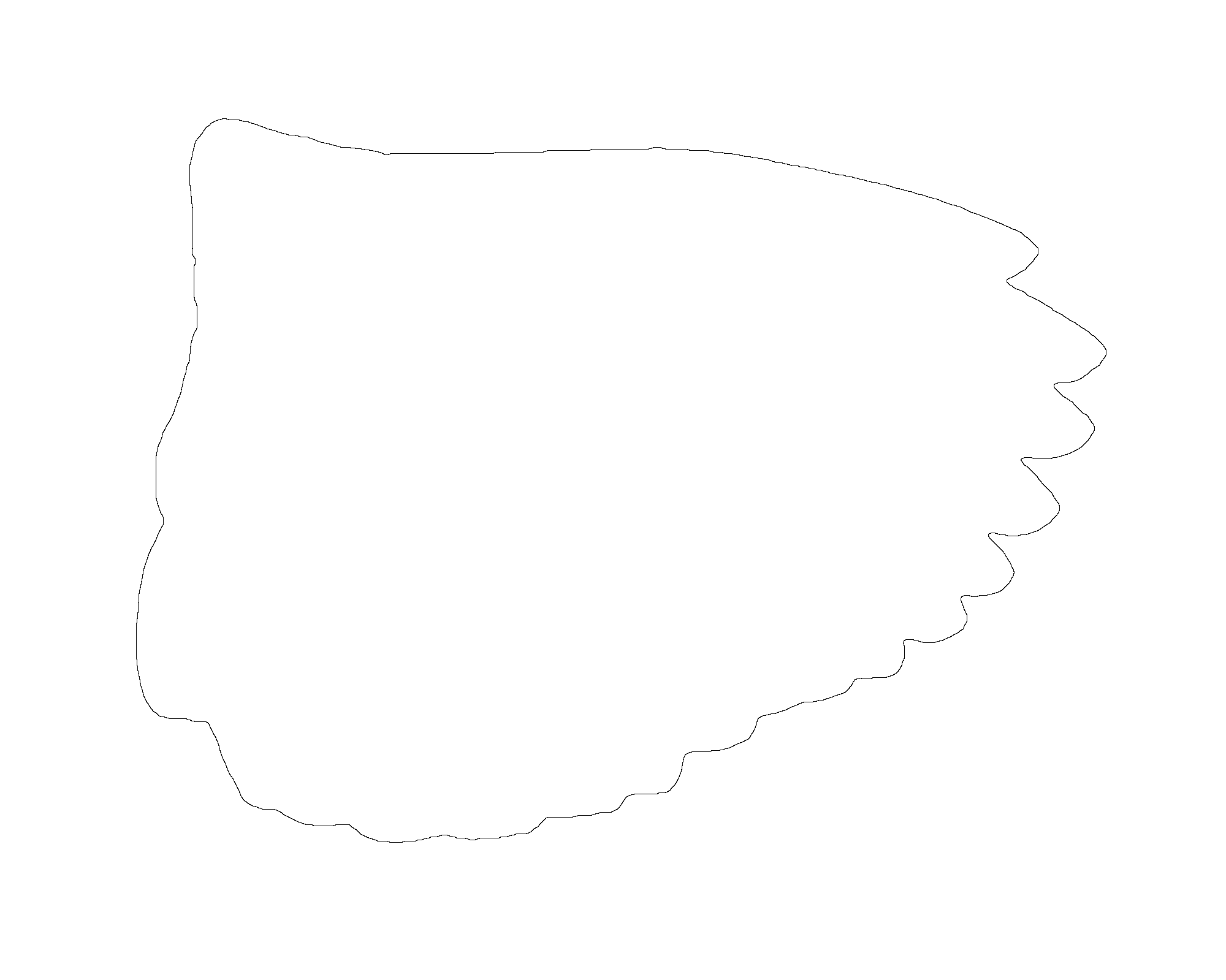

Supplement: Supplementary file 6 — Supplementary Data 4 [file 41467_2026_70692_MOESM6_ESM.zip › Supplementary Data 4/Aimophila_rufescens.tif]

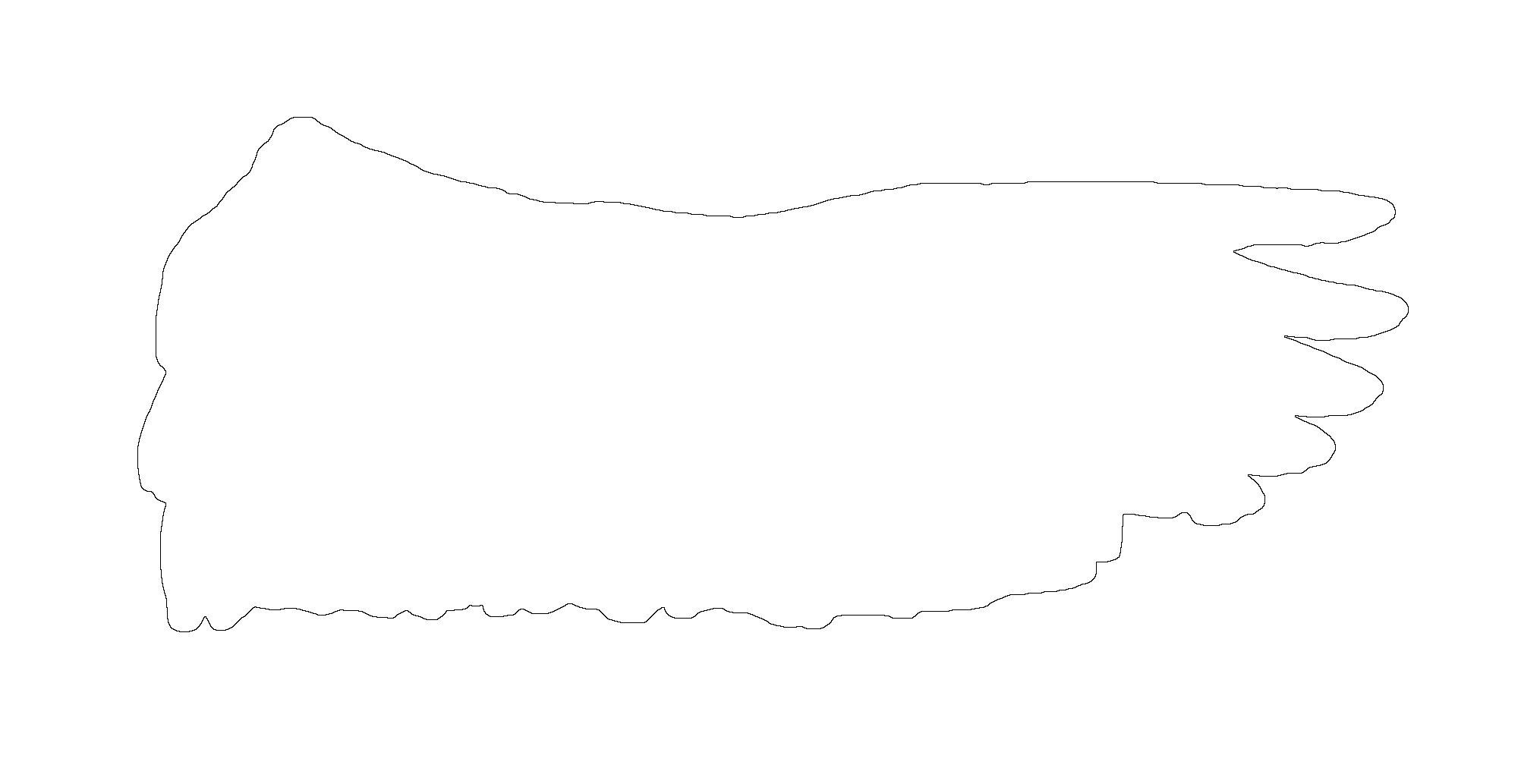

Supplement: Supplementary file 6 — Supplementary Data 4 [file 41467_2026_70692_MOESM6_ESM.zip › Supplementary Data 4/Aix_galericulata.tif]

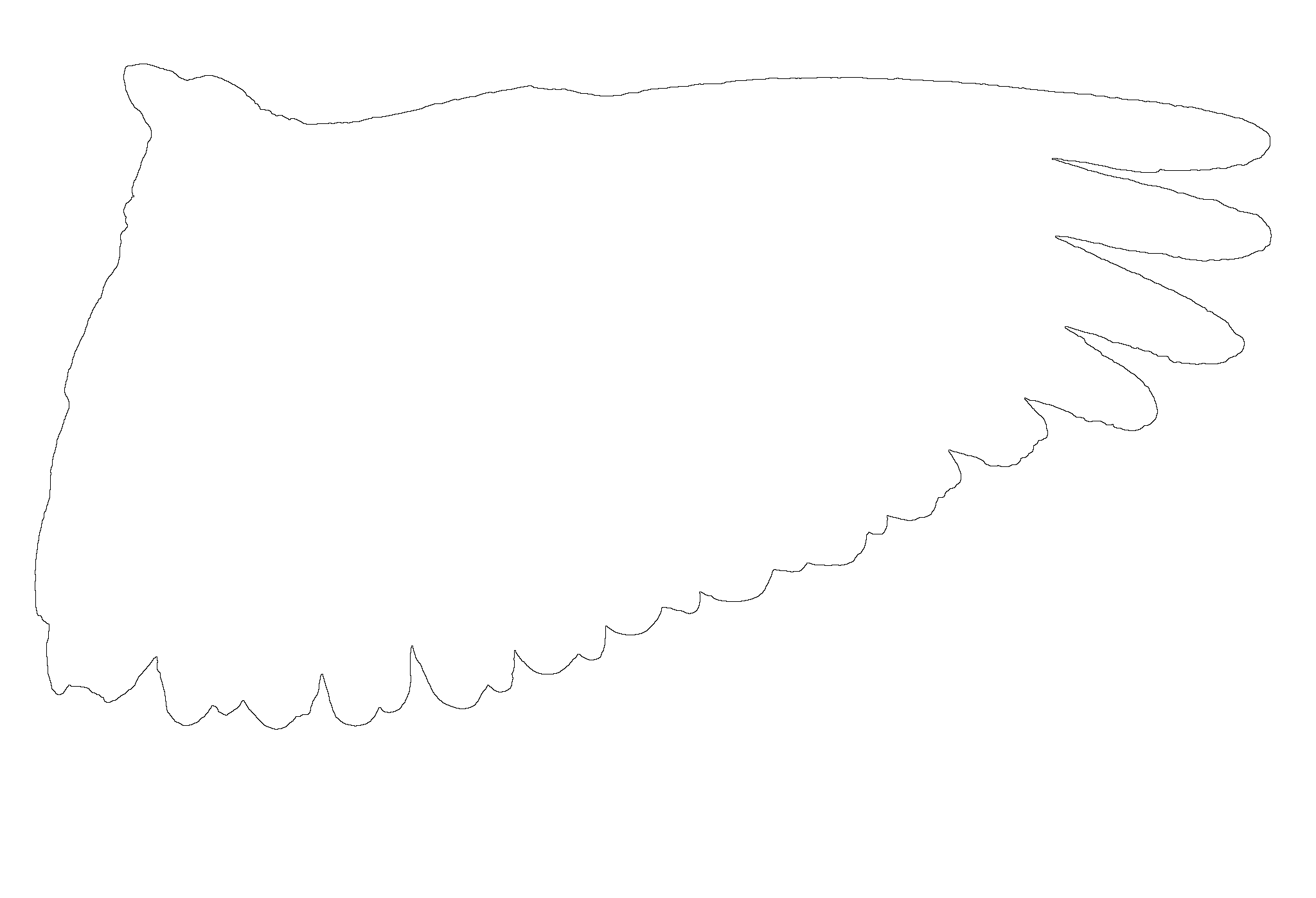

Supplement: Supplementary file 6 — Supplementary Data 4 [file 41467_2026_70692_MOESM6_ESM.zip › Supplementary Data 4/Alauda_arvensis.tif]

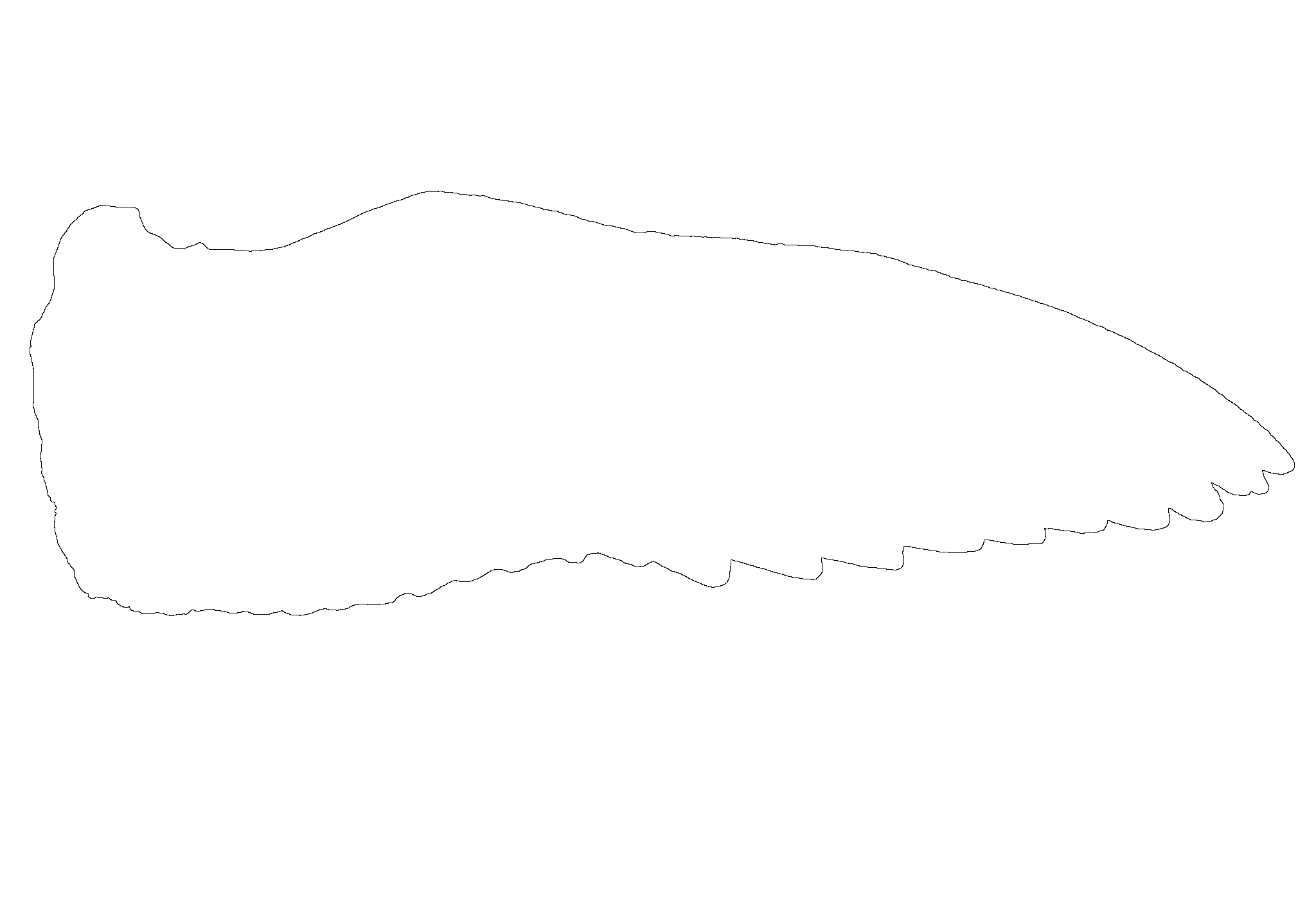

Supplement: Supplementary file 6 — Supplementary Data 4 [file 41467_2026_70692_MOESM6_ESM.zip › Supplementary Data 4/Alca_torda.tif]

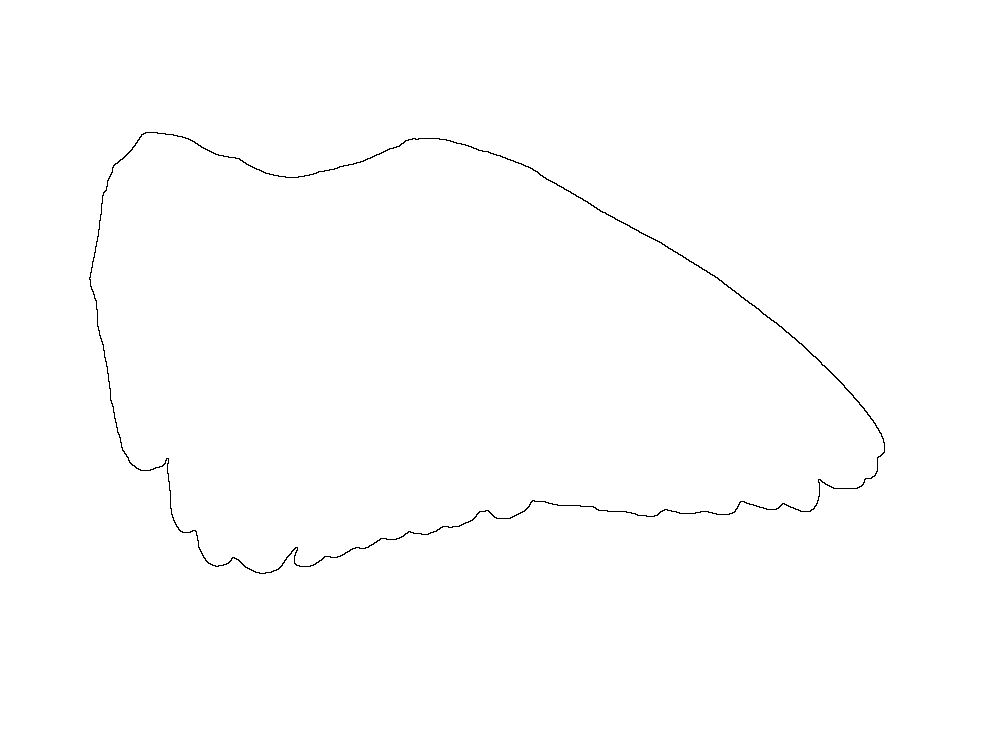

Supplement: Supplementary file 6 — Supplementary Data 4 [file 41467_2026_70692_MOESM6_ESM.zip › Supplementary Data 4/Alcedo_atthis.tif]

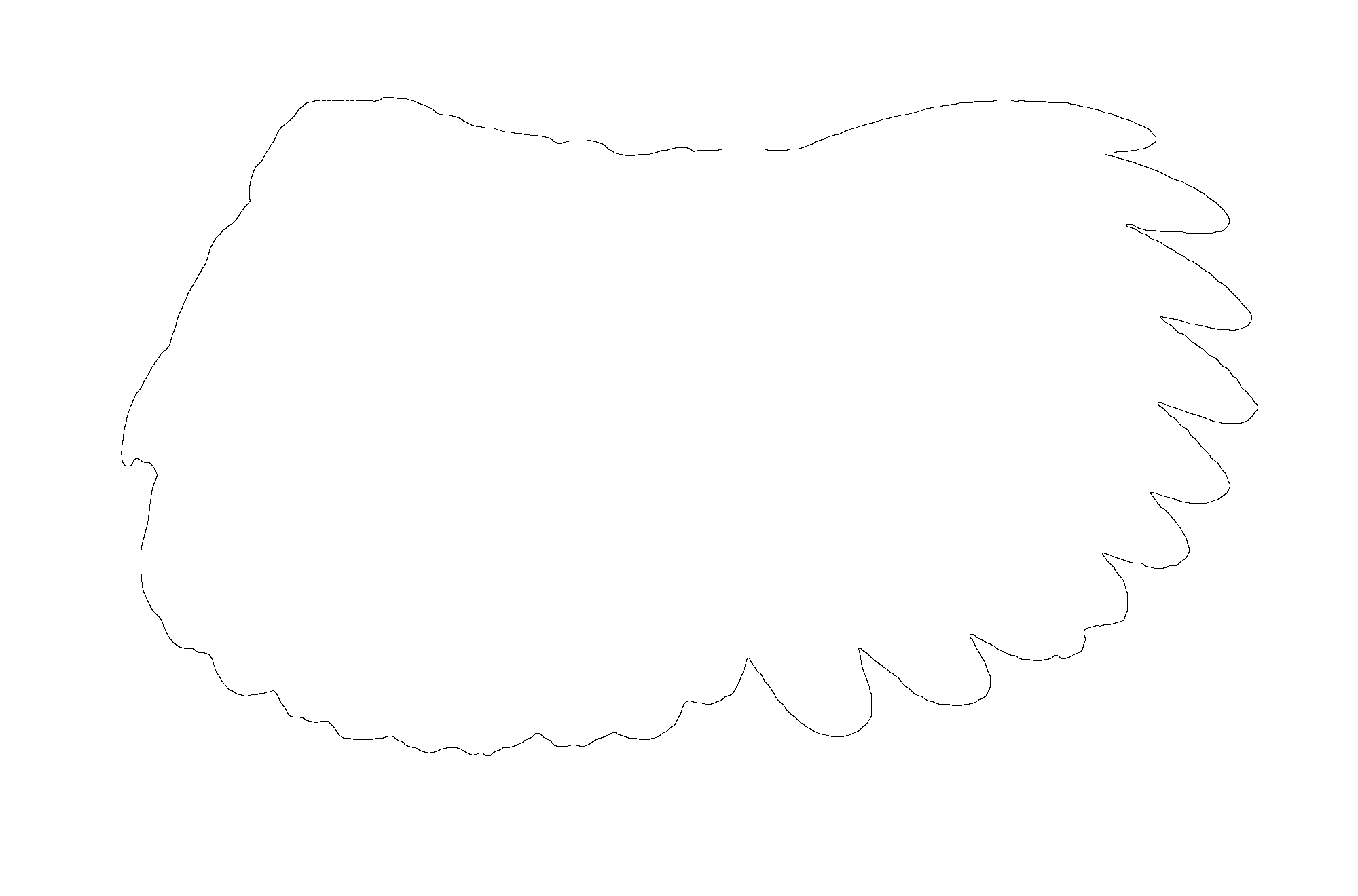

Supplement: Supplementary file 6 — Supplementary Data 4 [file 41467_2026_70692_MOESM6_ESM.zip › Supplementary Data 4/Alectura_lathami.tif]

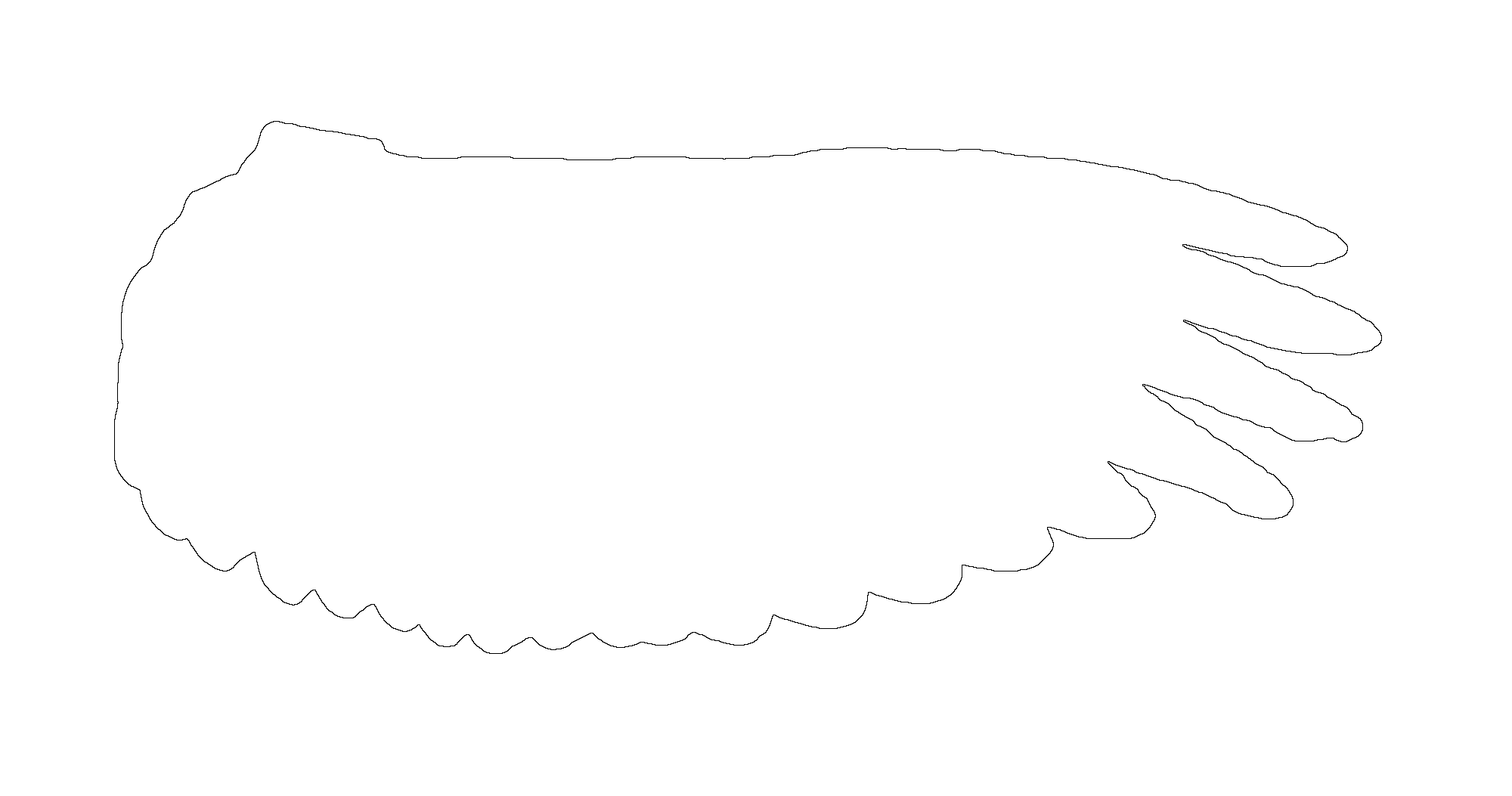

Supplement: Supplementary file 6 — Supplementary Data 4 [file 41467_2026_70692_MOESM6_ESM.zip › Supplementary Data 4/Alisterus_scapularis.tif]

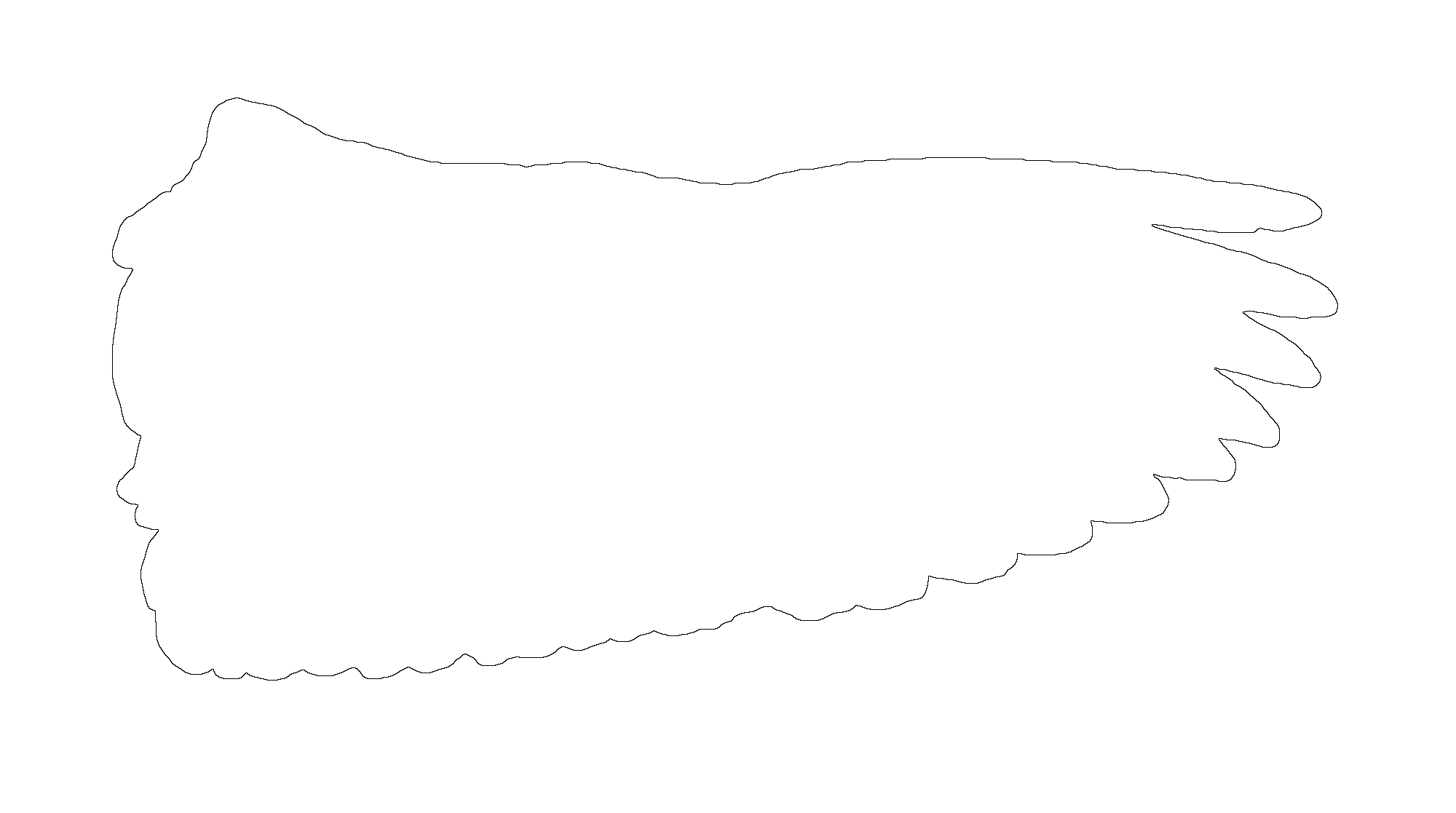

Supplement: Supplementary file 6 — Supplementary Data 4 [file 41467_2026_70692_MOESM6_ESM.zip › Supplementary Data 4/Alopochen_aegyptiaca.tif]

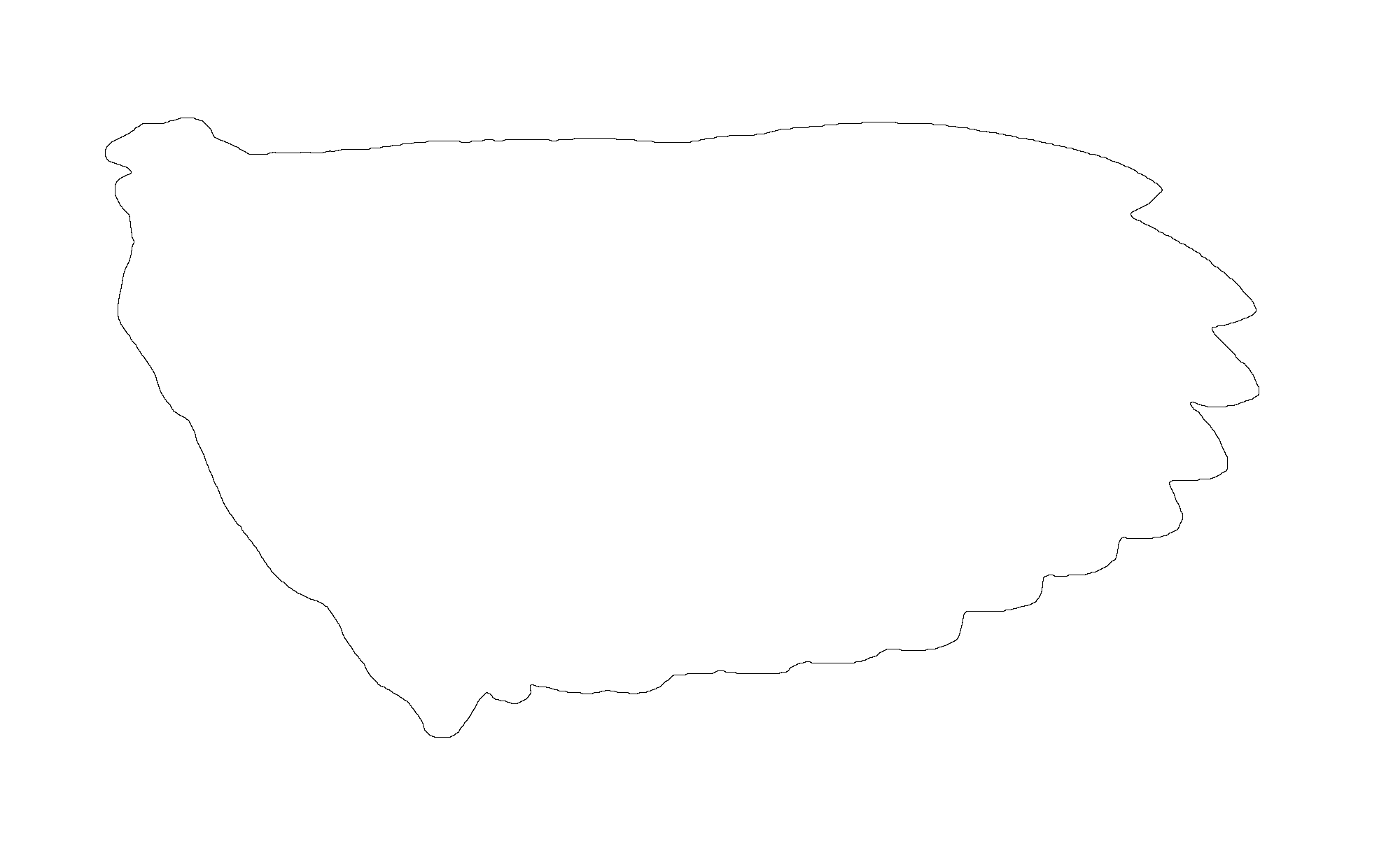

Supplement: Supplementary file 6 — Supplementary Data 4 [file 41467_2026_70692_MOESM6_ESM.zip › Supplementary Data 4/Amaurornis_phoenicurus.tif]

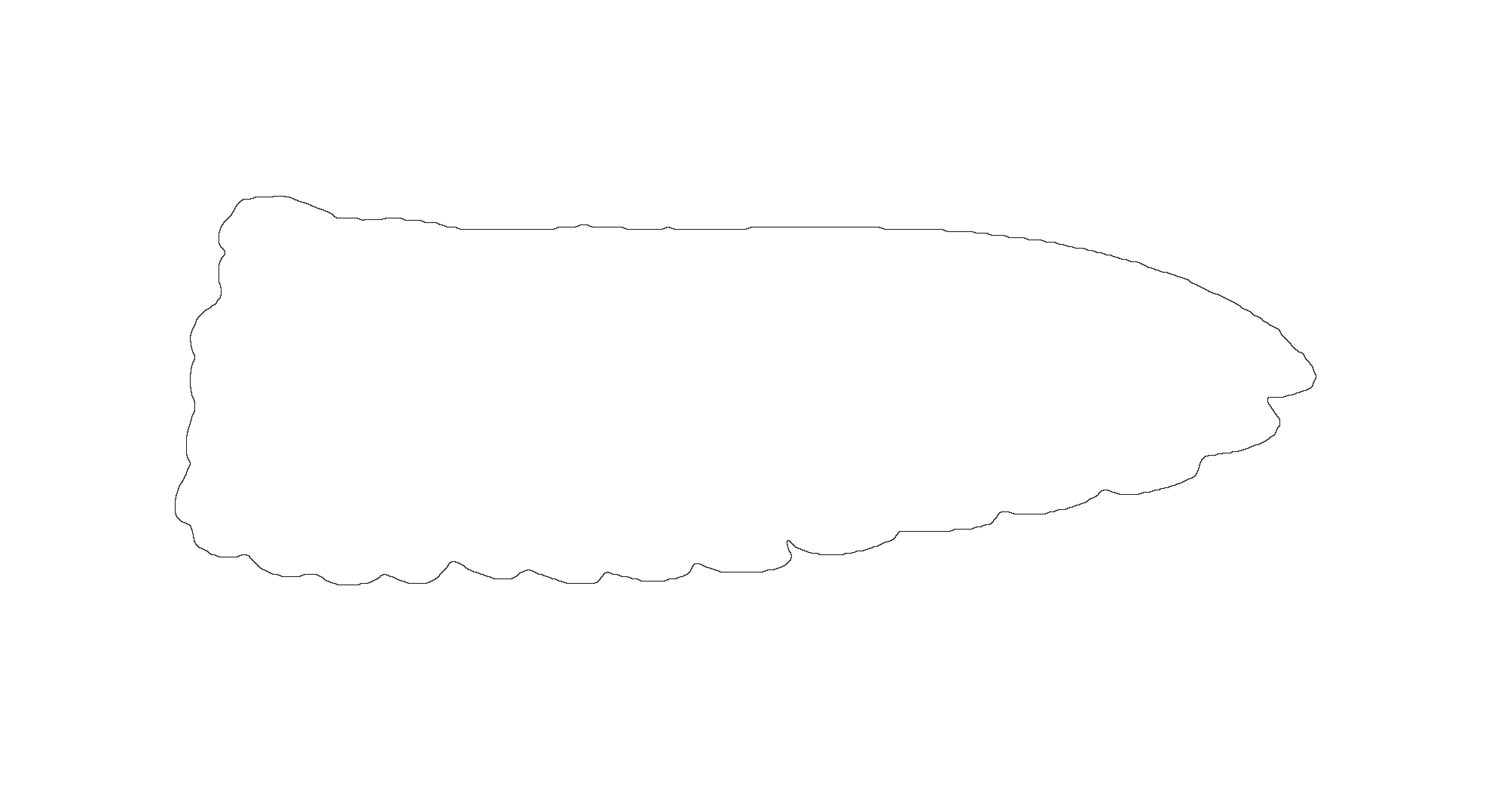

Supplement: Supplementary file 6 — Supplementary Data 4 [file 41467_2026_70692_MOESM6_ESM.zip › Supplementary Data 4/Amazilia_tzacatl.tif]

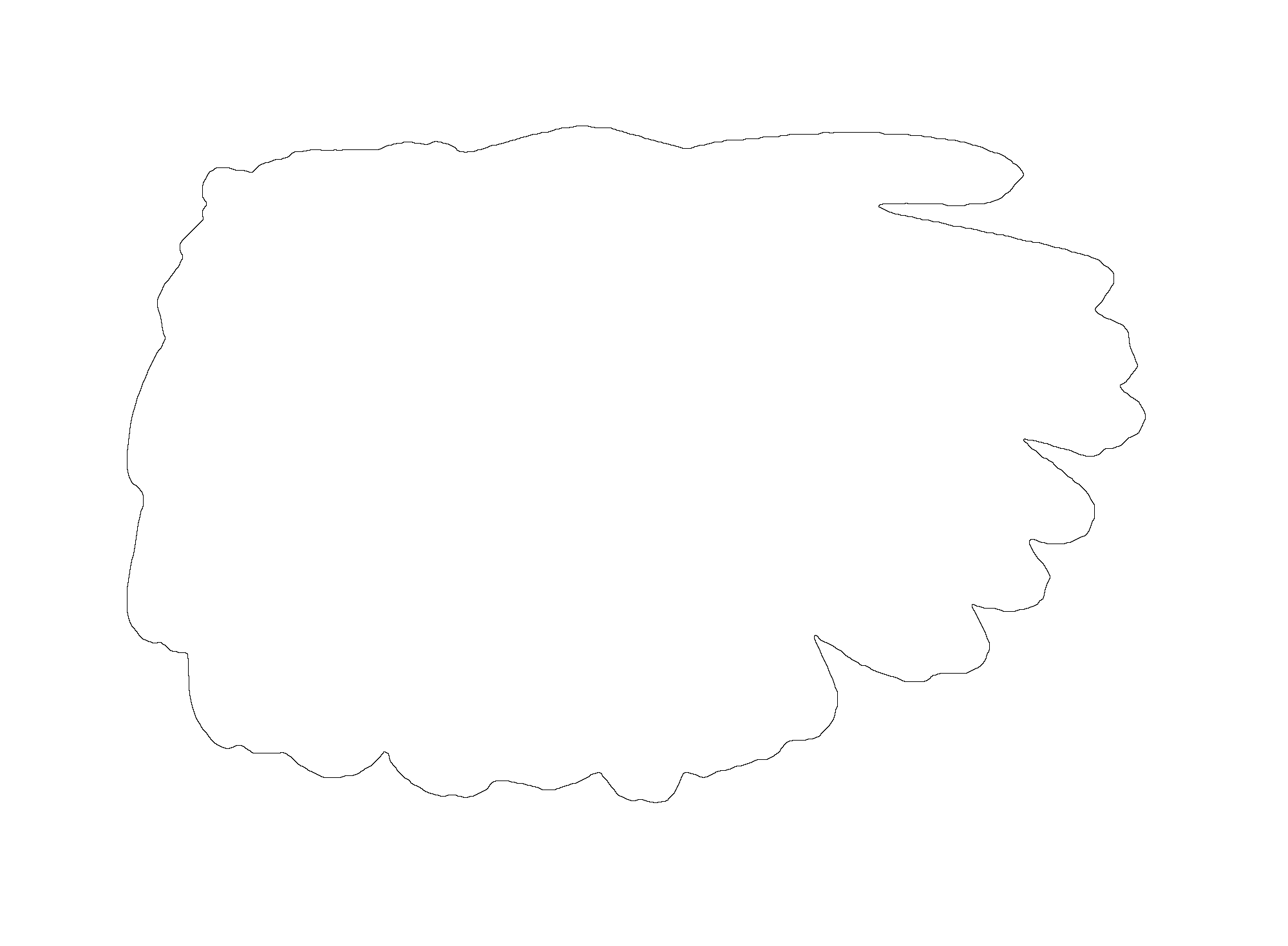

Supplement: Supplementary file 6 — Supplementary Data 4 [file 41467_2026_70692_MOESM6_ESM.zip › Supplementary Data 4/Amblycercus_holosericeus.tif]

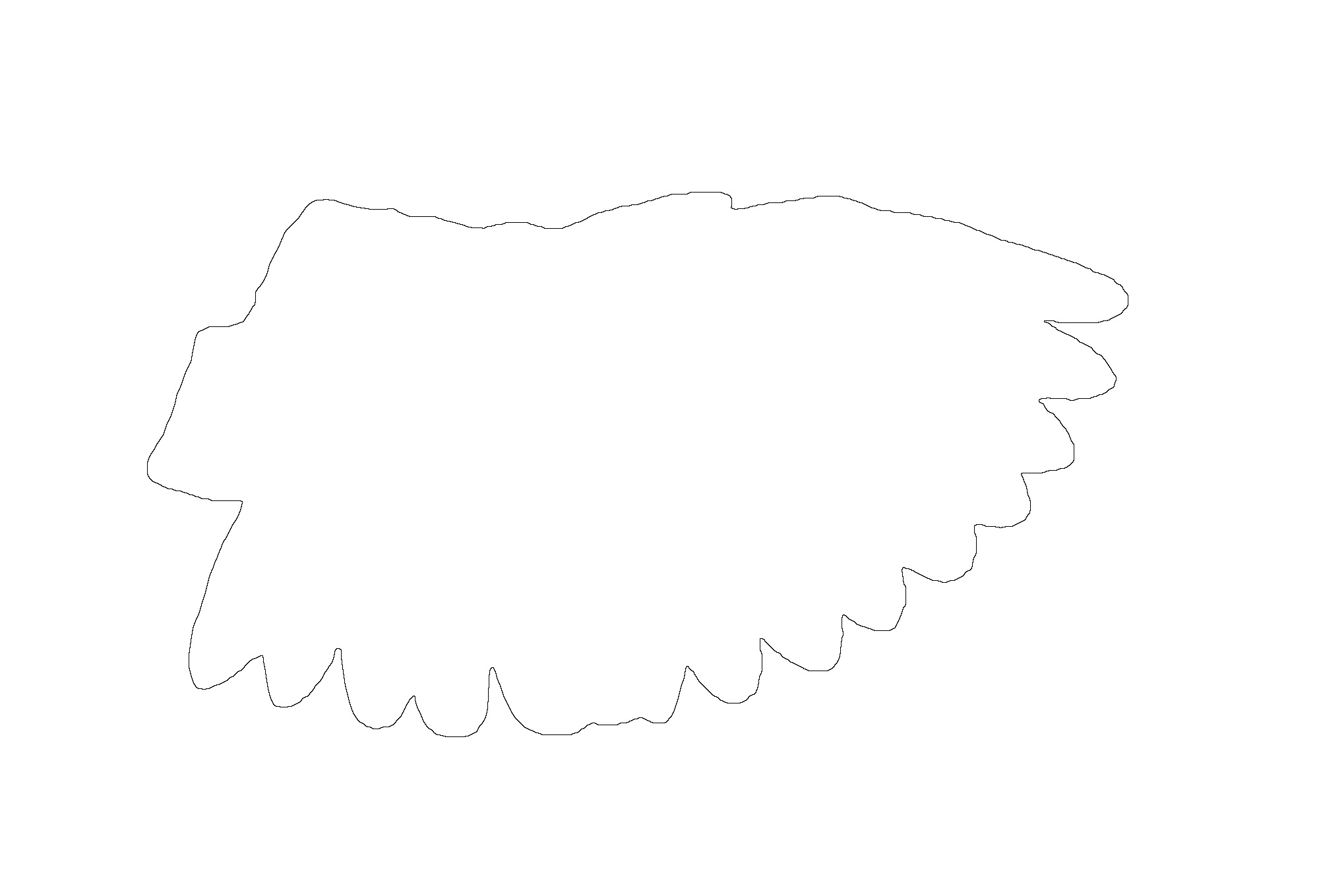

Supplement: Supplementary file 6 — Supplementary Data 4 [file 41467_2026_70692_MOESM6_ESM.zip › Supplementary Data 4/Ammodramus_leconteii.tif]

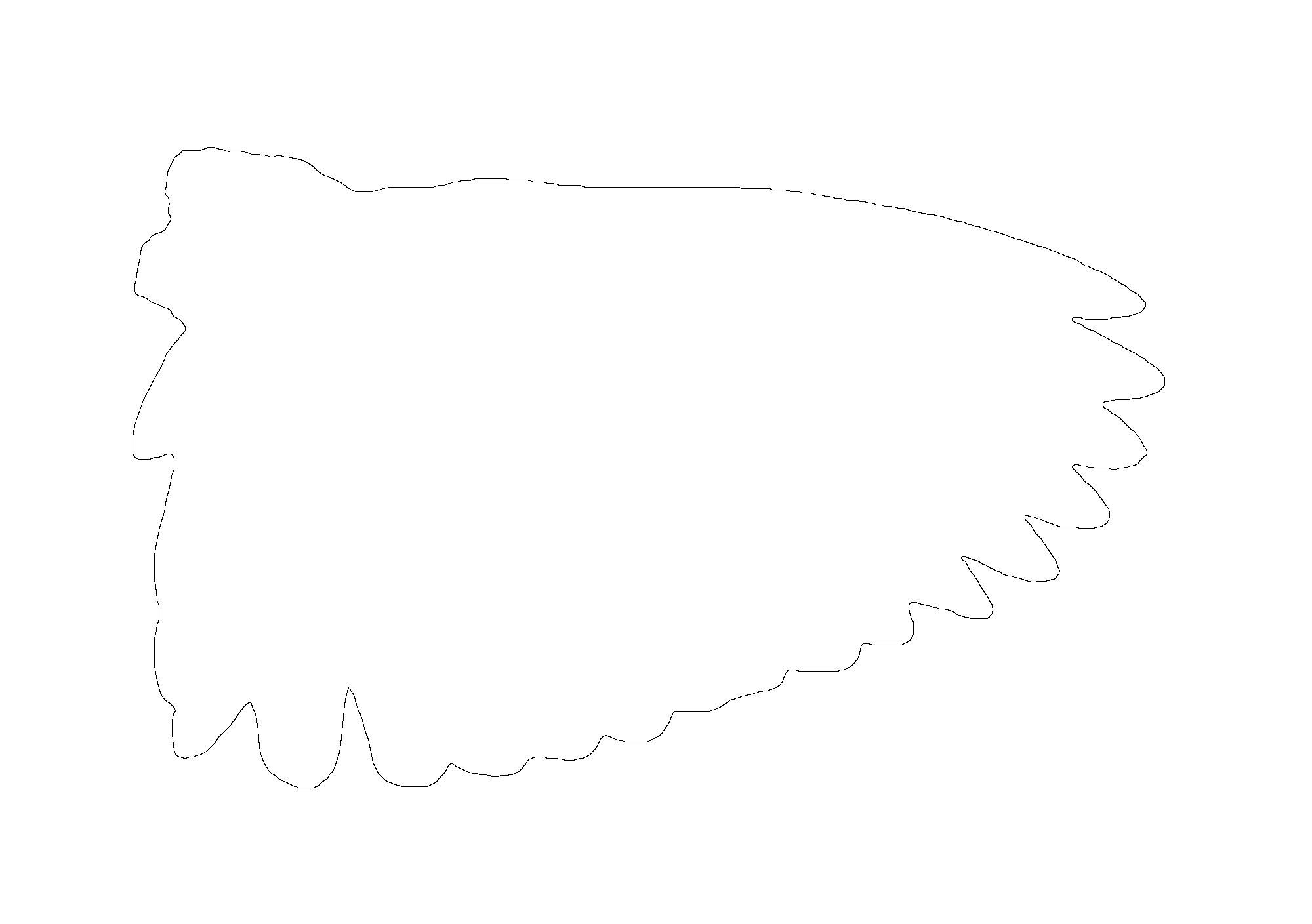

Supplement: Supplementary file 6 — Supplementary Data 4 [file 41467_2026_70692_MOESM6_ESM.zip › Supplementary Data 4/Amphispiza_belli.tif]

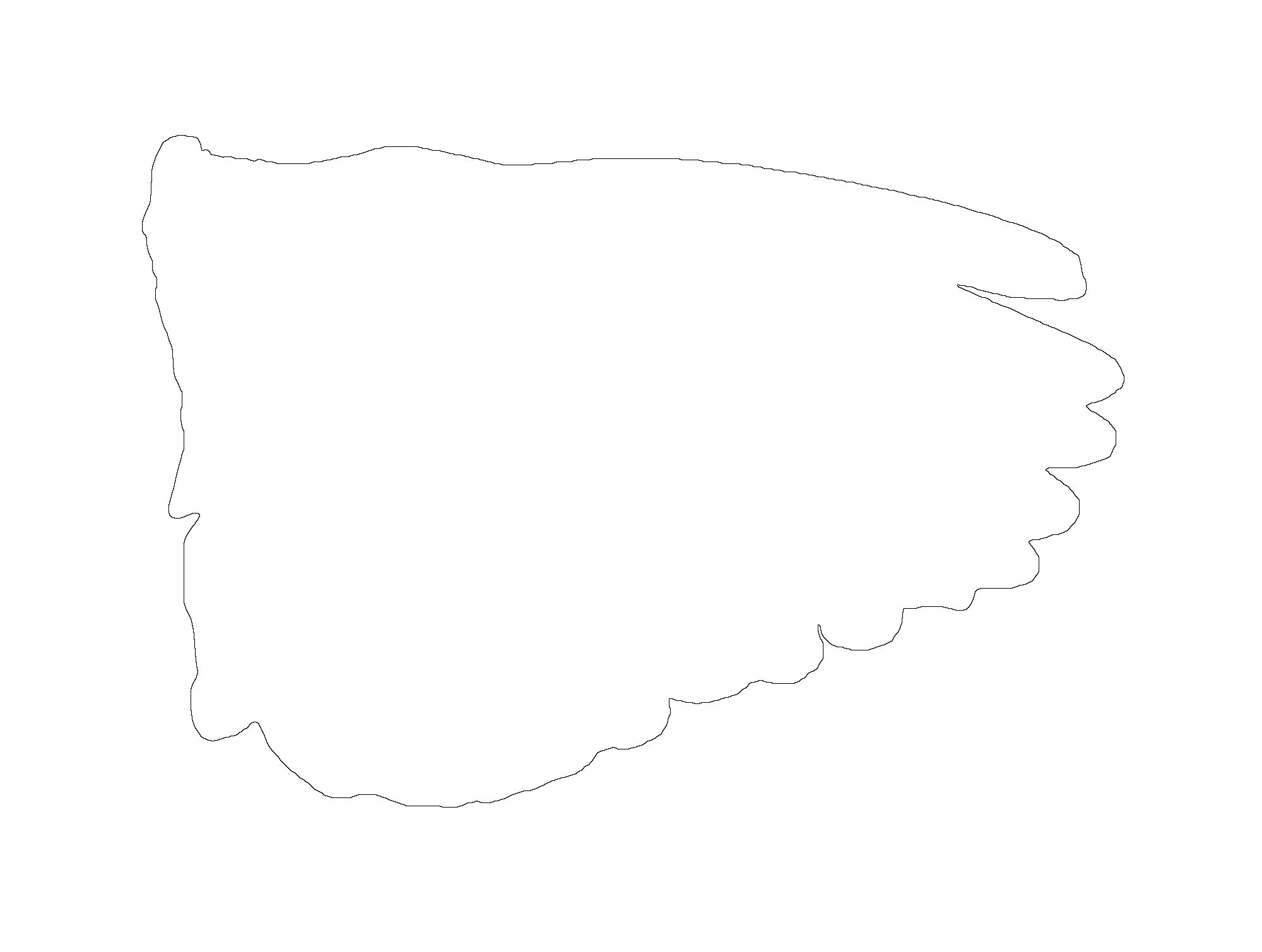

Supplement: Supplementary file 6 — Supplementary Data 4 [file 41467_2026_70692_MOESM6_ESM.zip › Supplementary Data 4/Amphispiza_bilineata.tif]

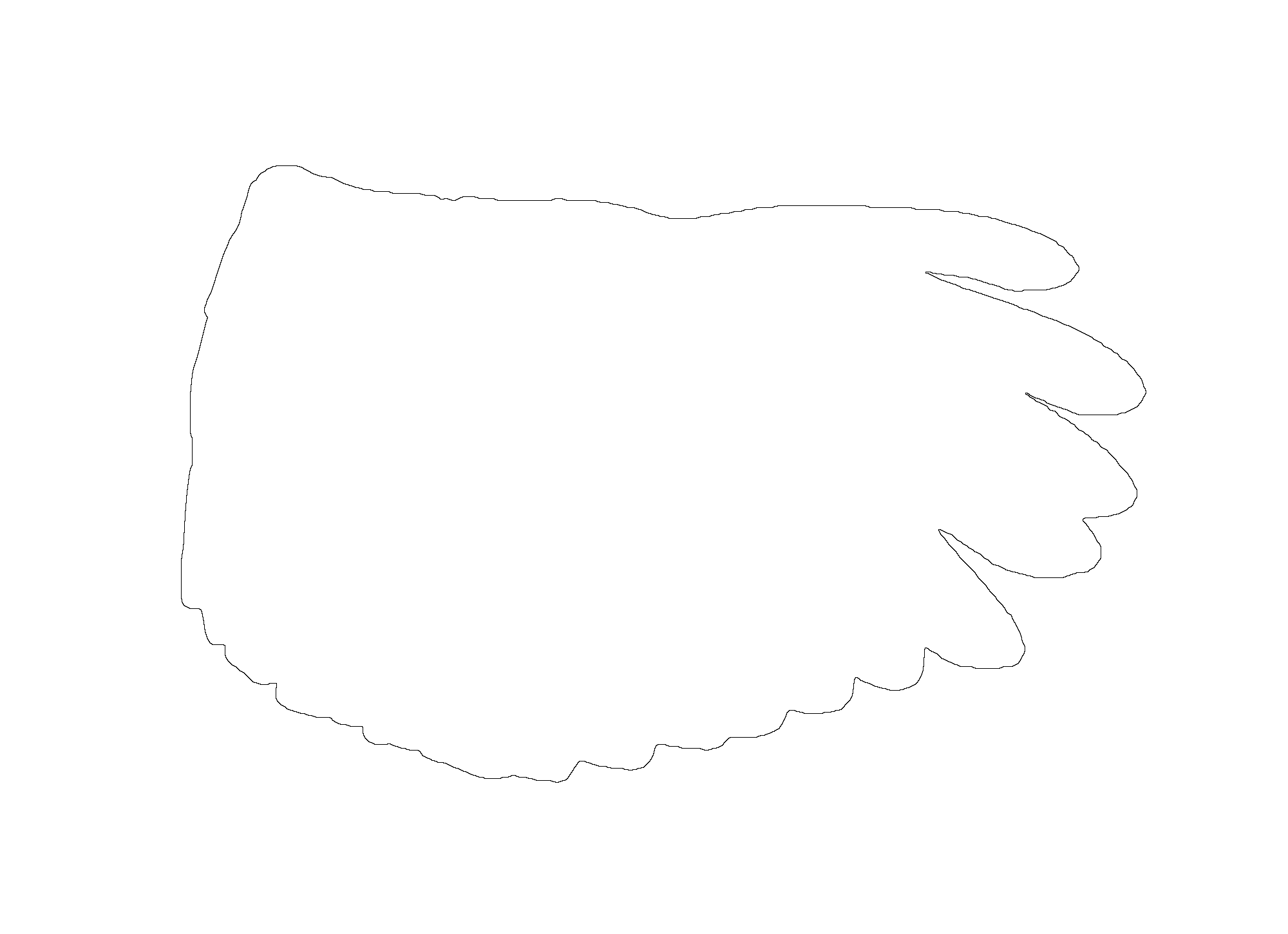

Supplement: Supplementary file 6 — Supplementary Data 4 [file 41467_2026_70692_MOESM6_ESM.zip › Supplementary Data 4/Anabacerthia_striaticollis.tif]

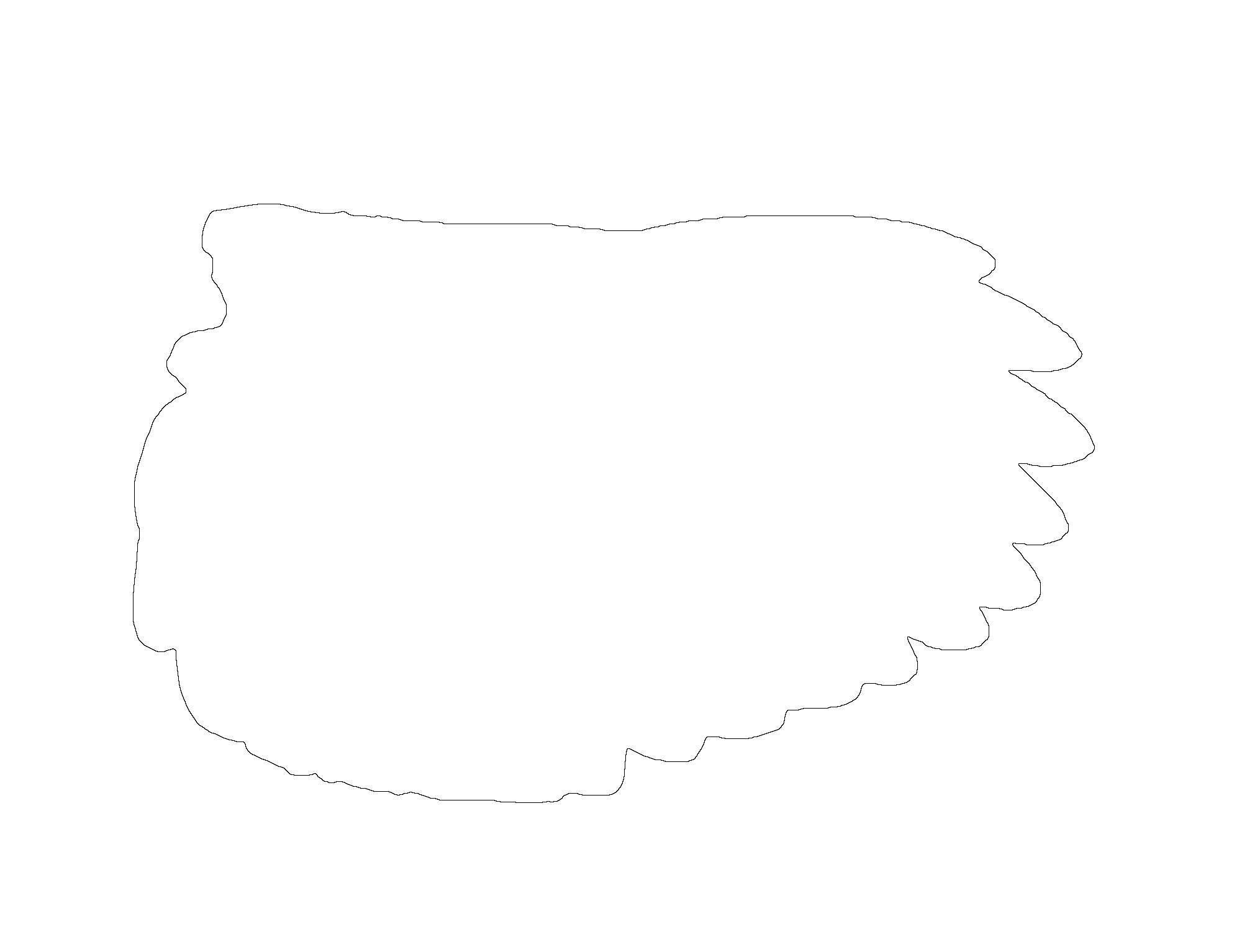

Supplement: Supplementary file 6 — Supplementary Data 4 [file 41467_2026_70692_MOESM6_ESM.zip › Supplementary Data 4/Anabacerthia_variegaticeps.tif]

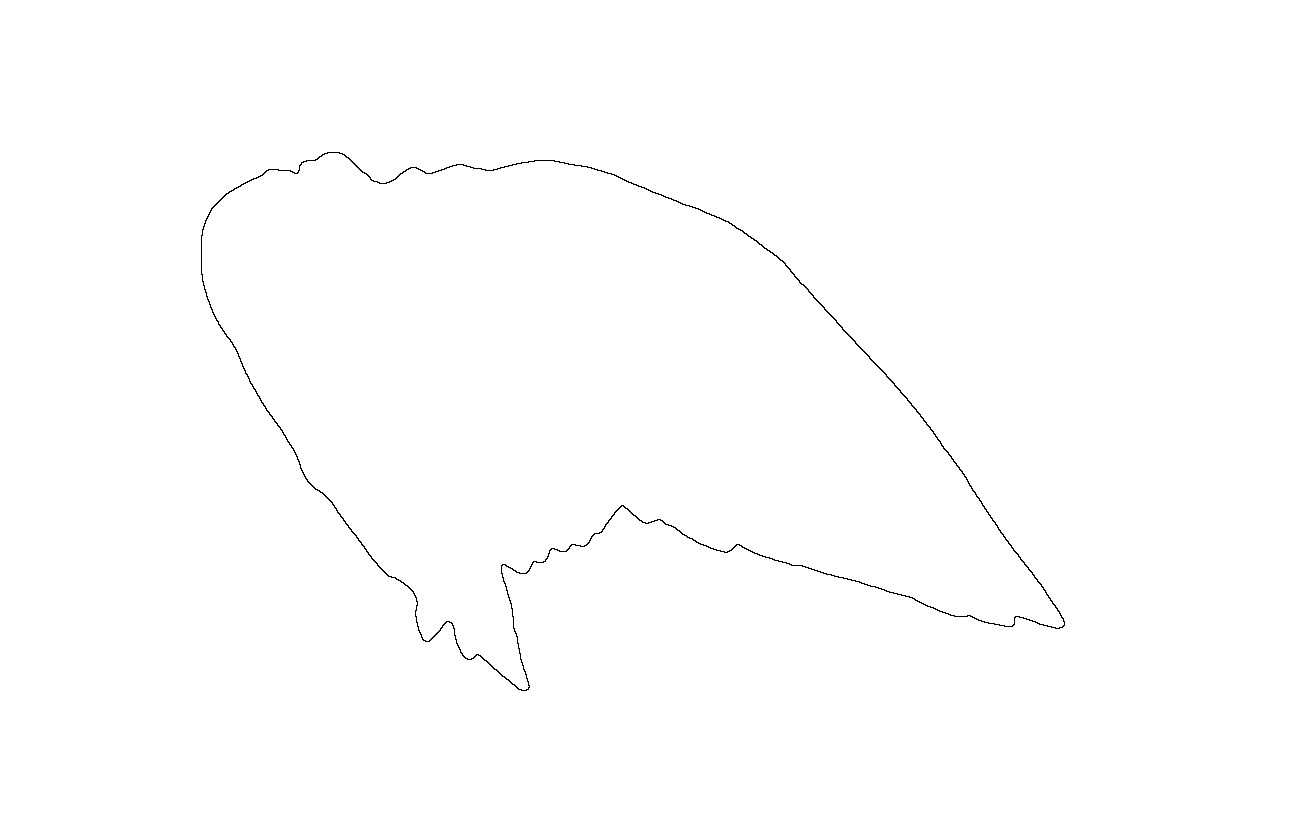

Supplement: Supplementary file 6 — Supplementary Data 4 [file 41467_2026_70692_MOESM6_ESM.zip › Supplementary Data 4/Anas_bahamensis.tif]

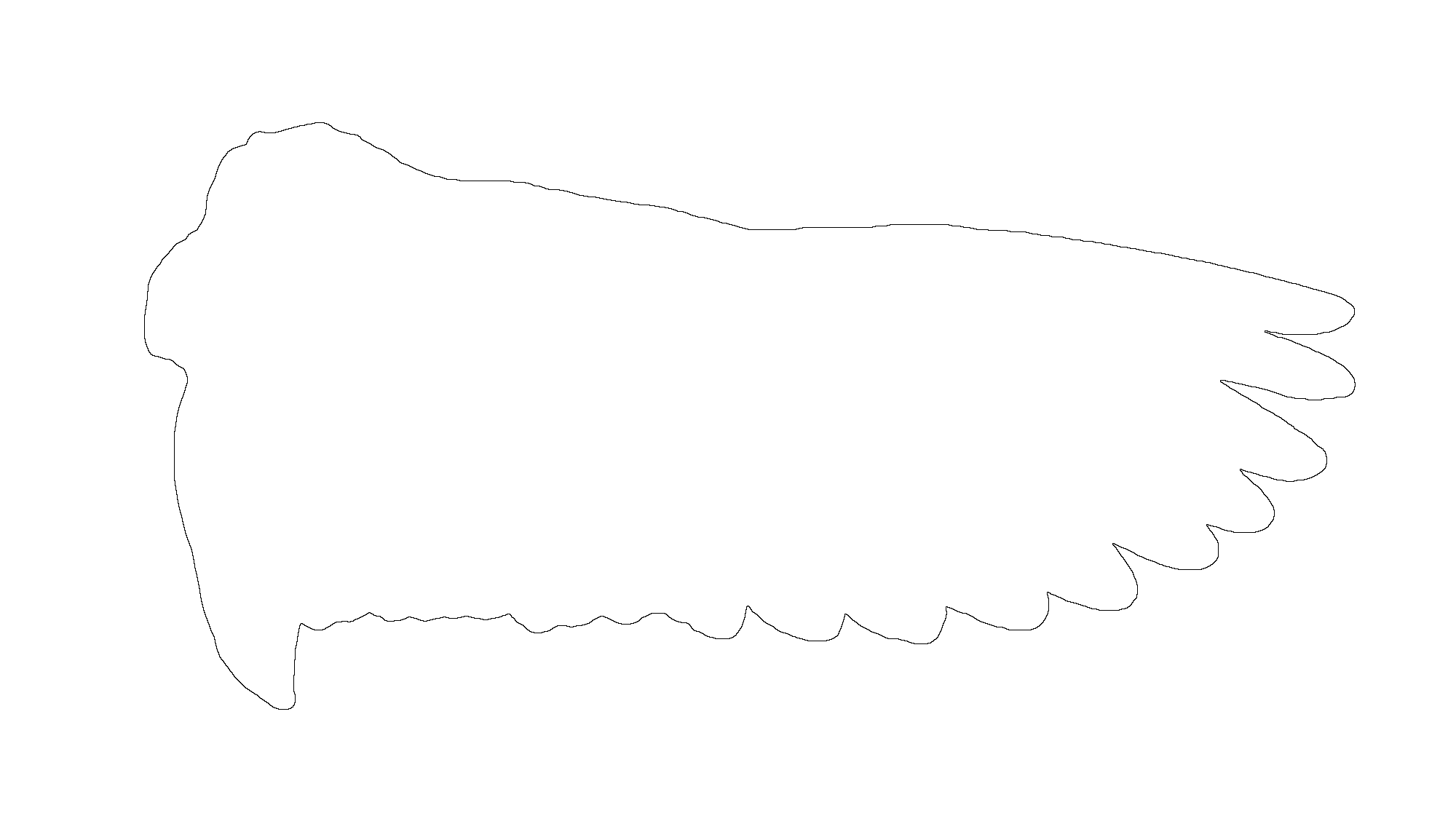

Supplement: Supplementary file 6 — Supplementary Data 4 [file 41467_2026_70692_MOESM6_ESM.zip › Supplementary Data 4/Anas_capensis.tif]

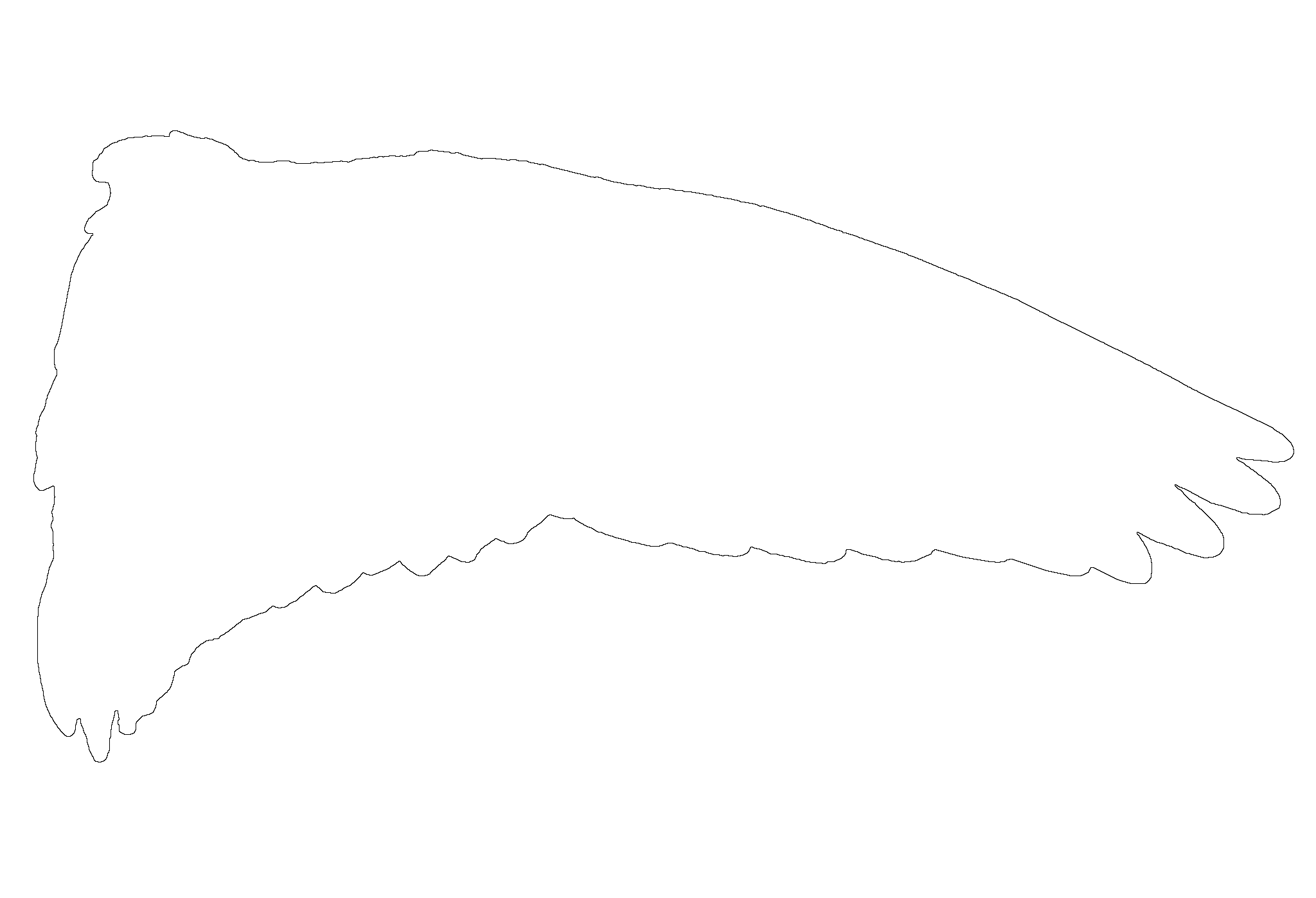

Supplement: Supplementary file 6 — Supplementary Data 4 [file 41467_2026_70692_MOESM6_ESM.zip › Supplementary Data 4/Anas_clypeata.tif]

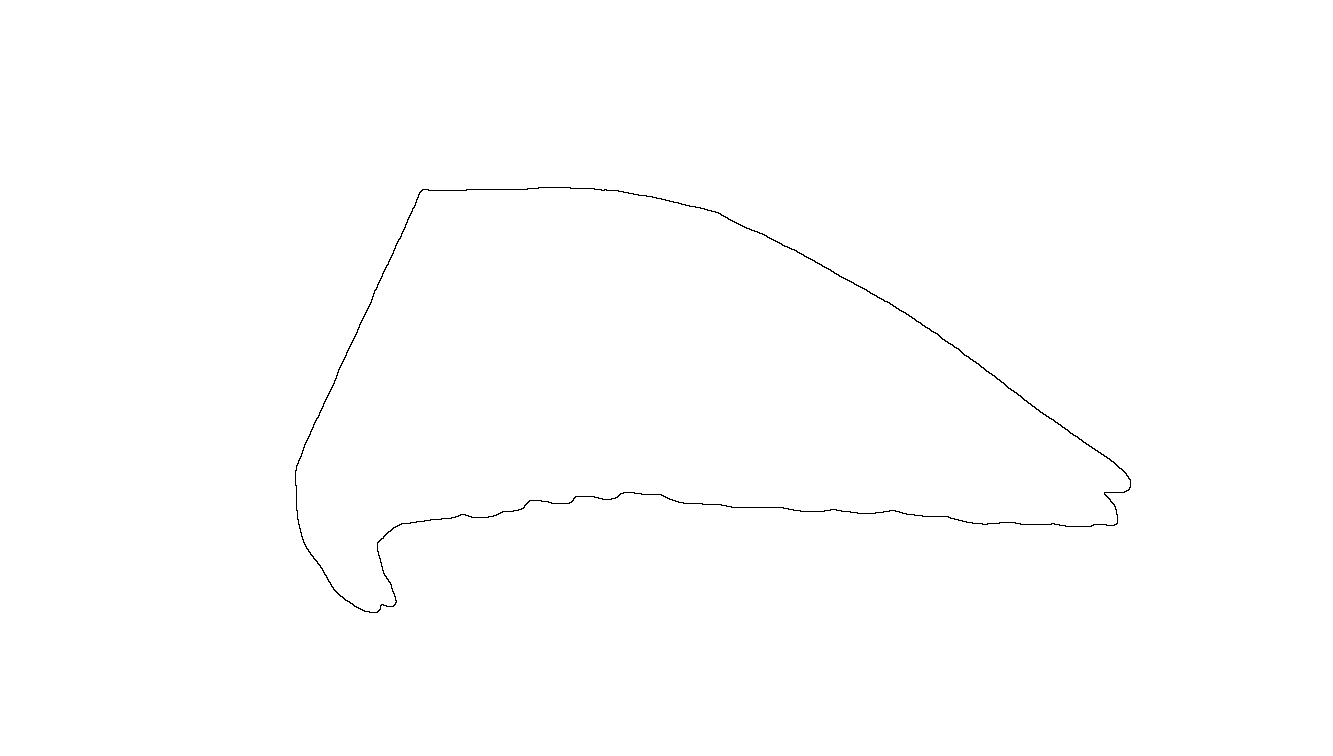

Supplement: Supplementary file 6 — Supplementary Data 4 [file 41467_2026_70692_MOESM6_ESM.zip › Supplementary Data 4/Anas_falcata.tif]

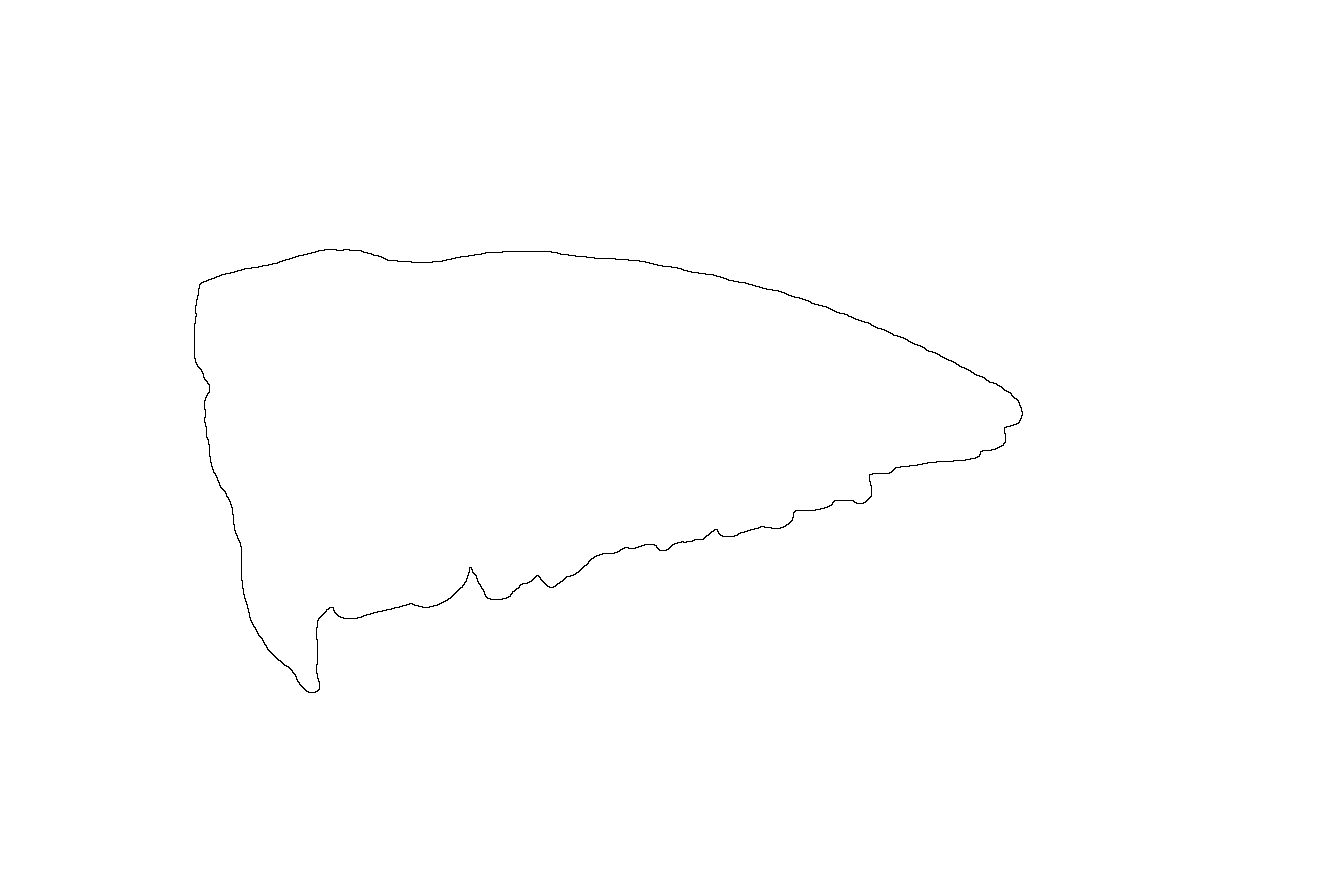

Supplement: Supplementary file 6 — Supplementary Data 4 [file 41467_2026_70692_MOESM6_ESM.zip › Supplementary Data 4/Anas_gibberifons.tif]

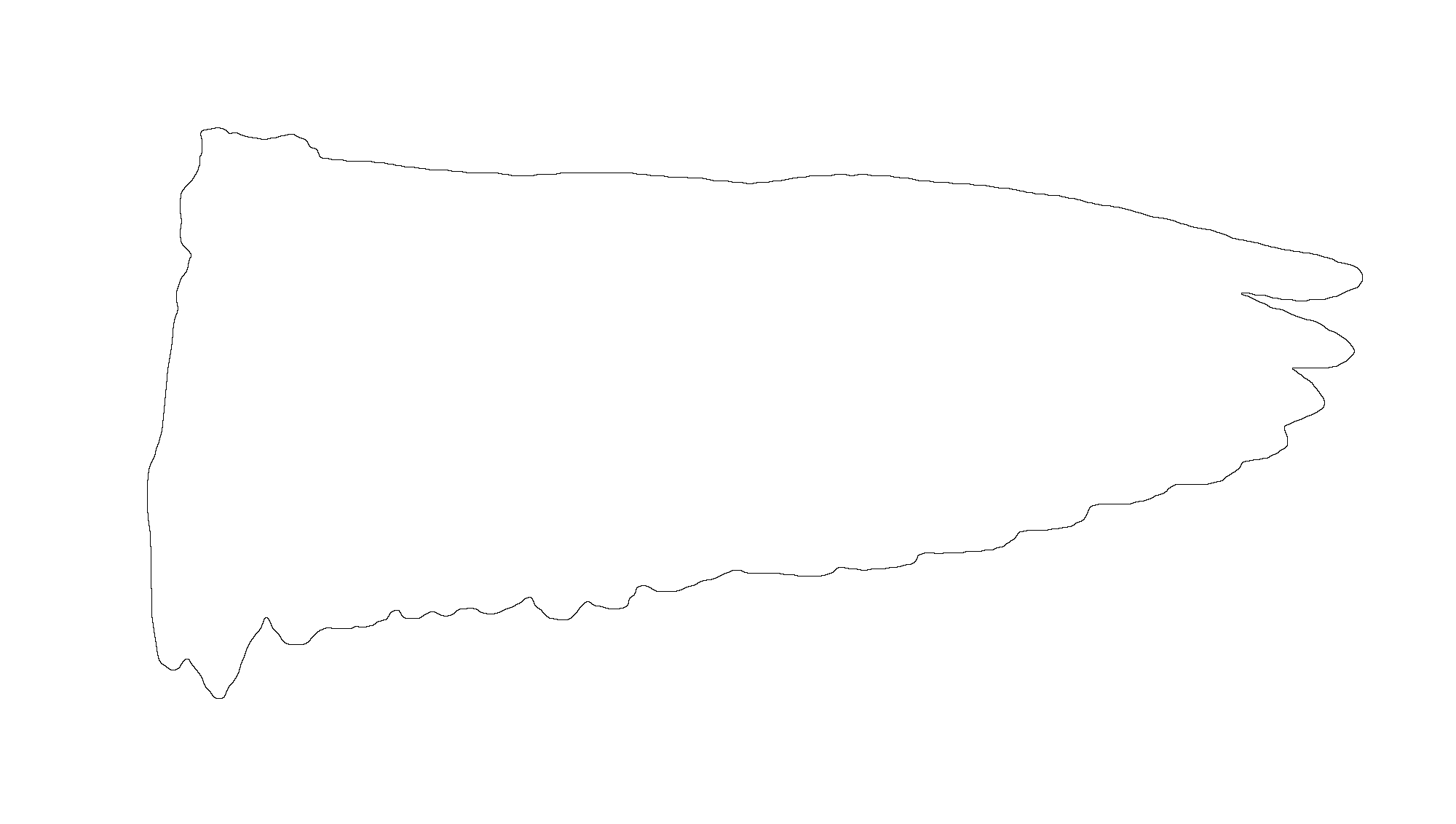

Supplement: Supplementary file 6 — Supplementary Data 4 [file 41467_2026_70692_MOESM6_ESM.zip › Supplementary Data 4/Anas_gibberifrons.tif]

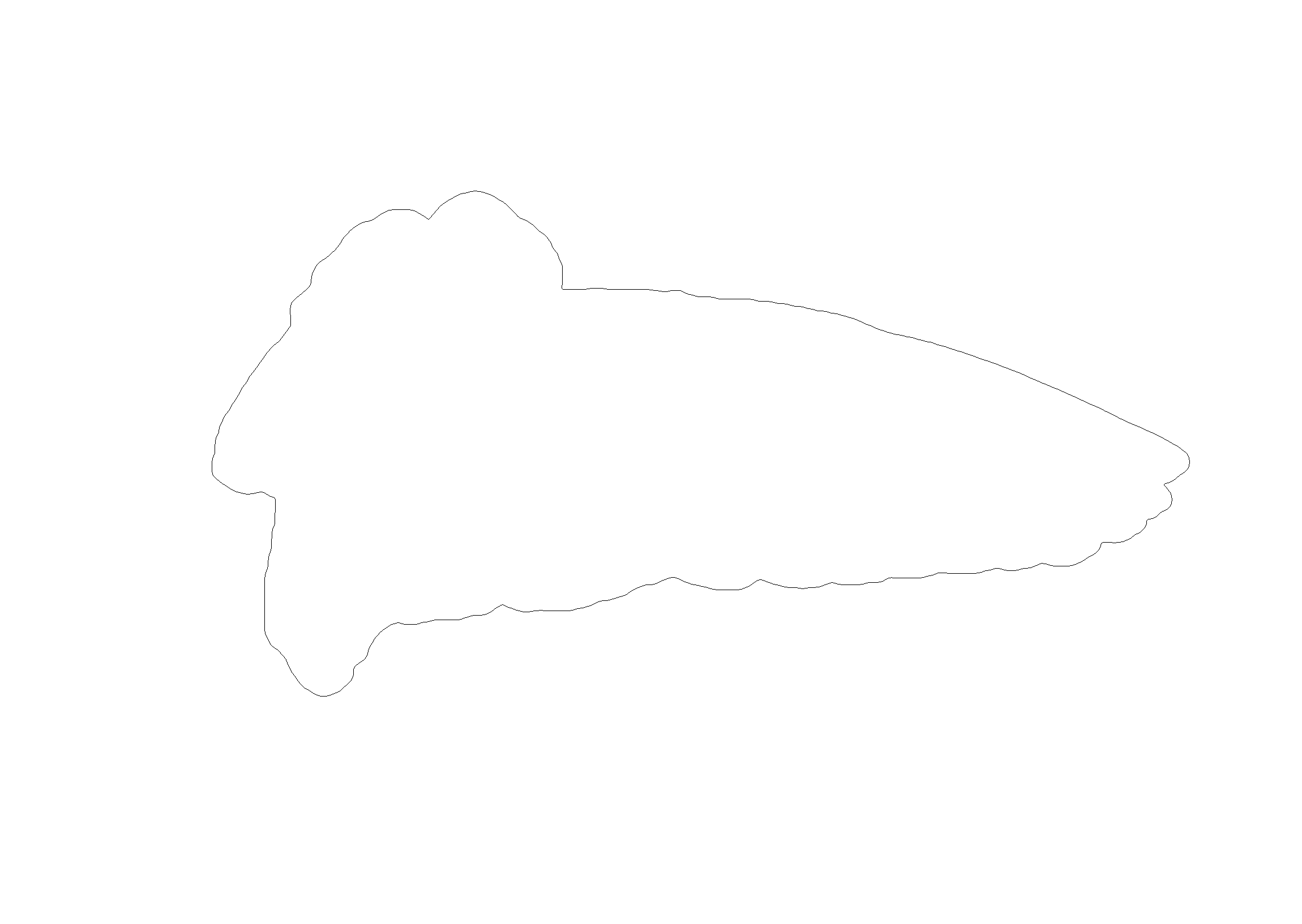

Supplement: Supplementary file 6 — Supplementary Data 4 [file 41467_2026_70692_MOESM6_ESM.zip › Supplementary Data 4/Anas_querquedula.tif]

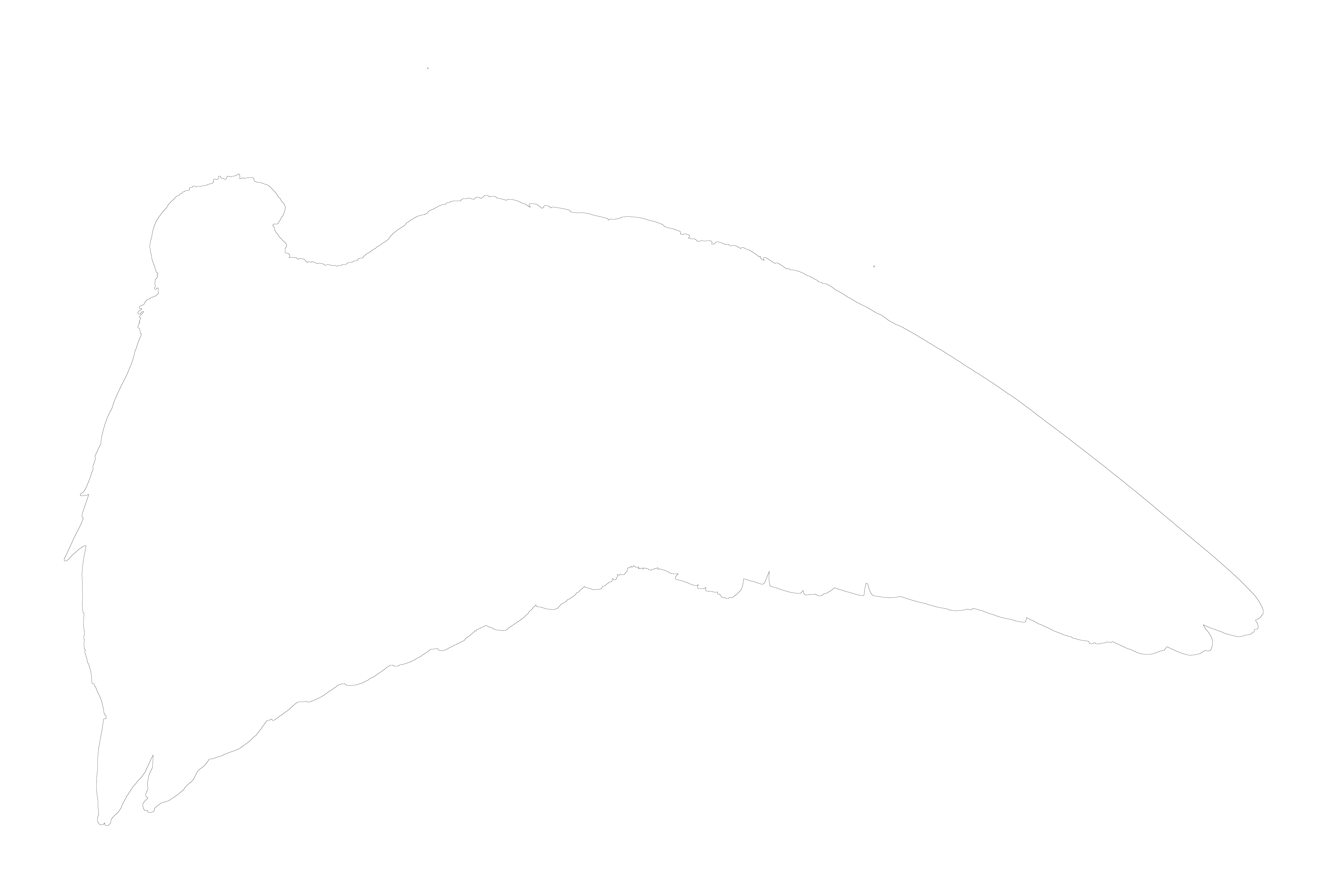

Supplement: Supplementary file 6 — Supplementary Data 4 [file 41467_2026_70692_MOESM6_ESM.zip › Supplementary Data 4/Anas_rhynchotis.tif]

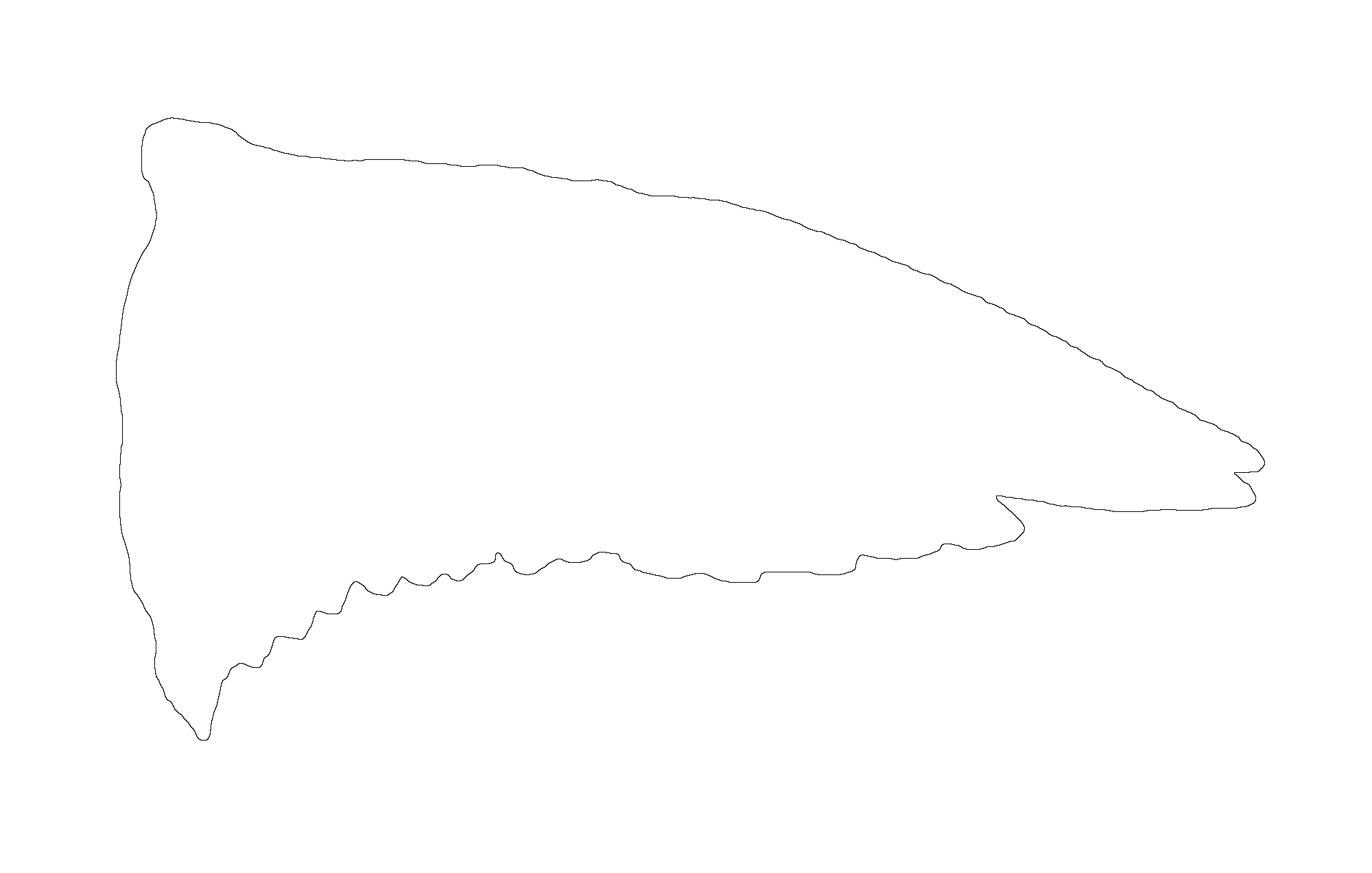

Supplement: Supplementary file 6 — Supplementary Data 4 [file 41467_2026_70692_MOESM6_ESM.zip › Supplementary Data 4/Anas_rubripes.tif]

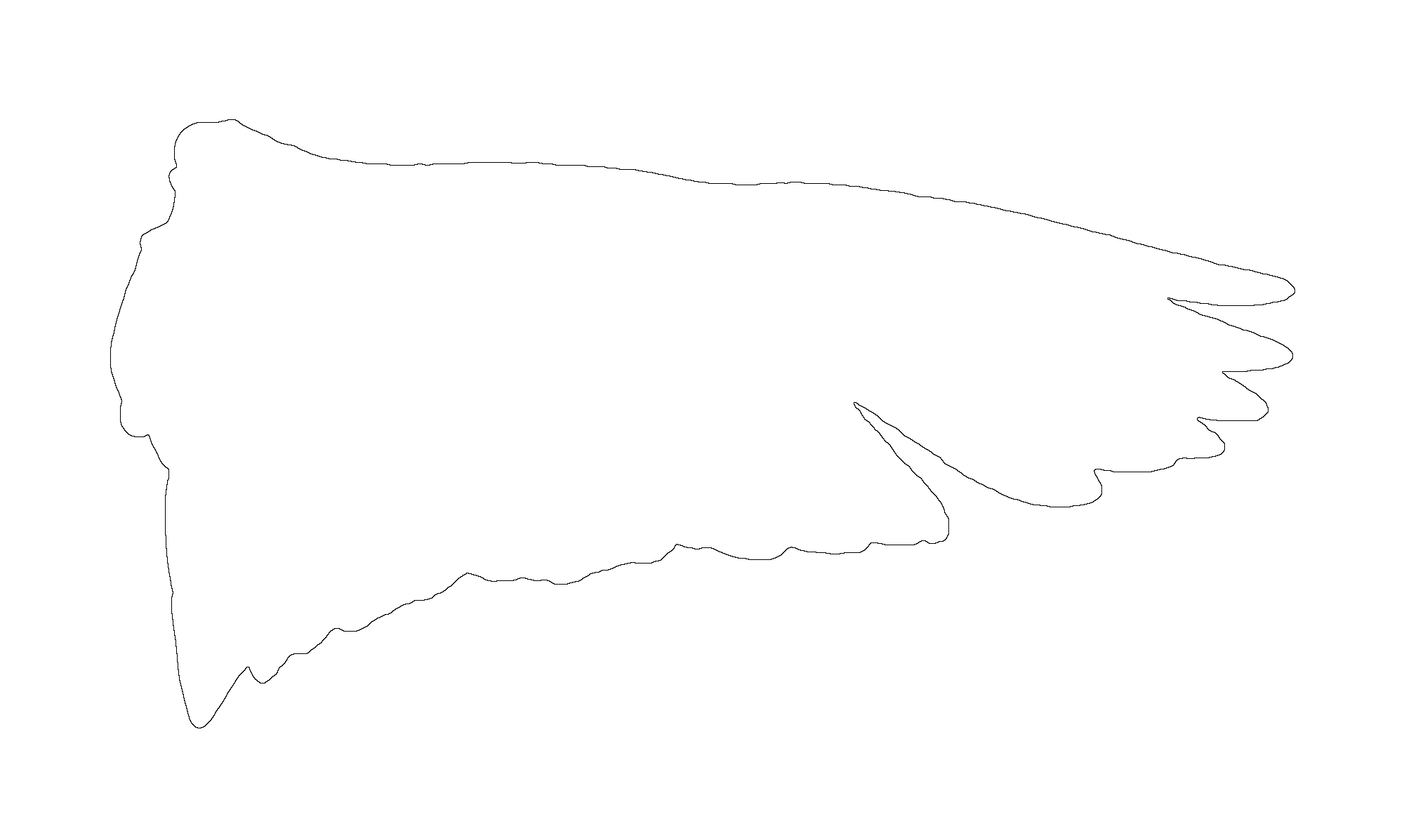

Supplement: Supplementary file 6 — Supplementary Data 4 [file 41467_2026_70692_MOESM6_ESM.zip › Supplementary Data 4/Anas_undulata.tif]

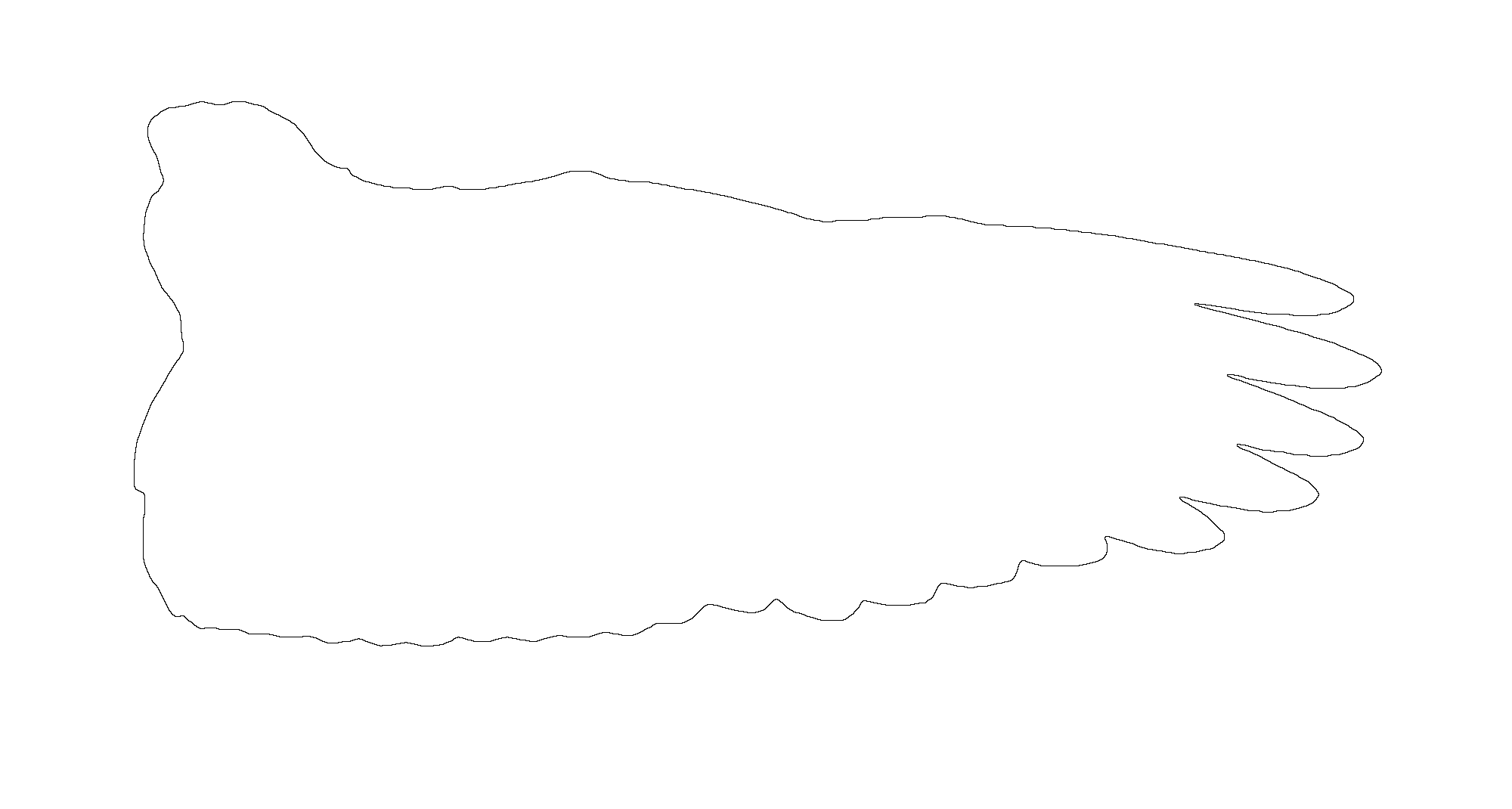

Supplement: Supplementary file 6 — Supplementary Data 4 [file 41467_2026_70692_MOESM6_ESM.zip › Supplementary Data 4/Anhinga_melanogaster.tif]

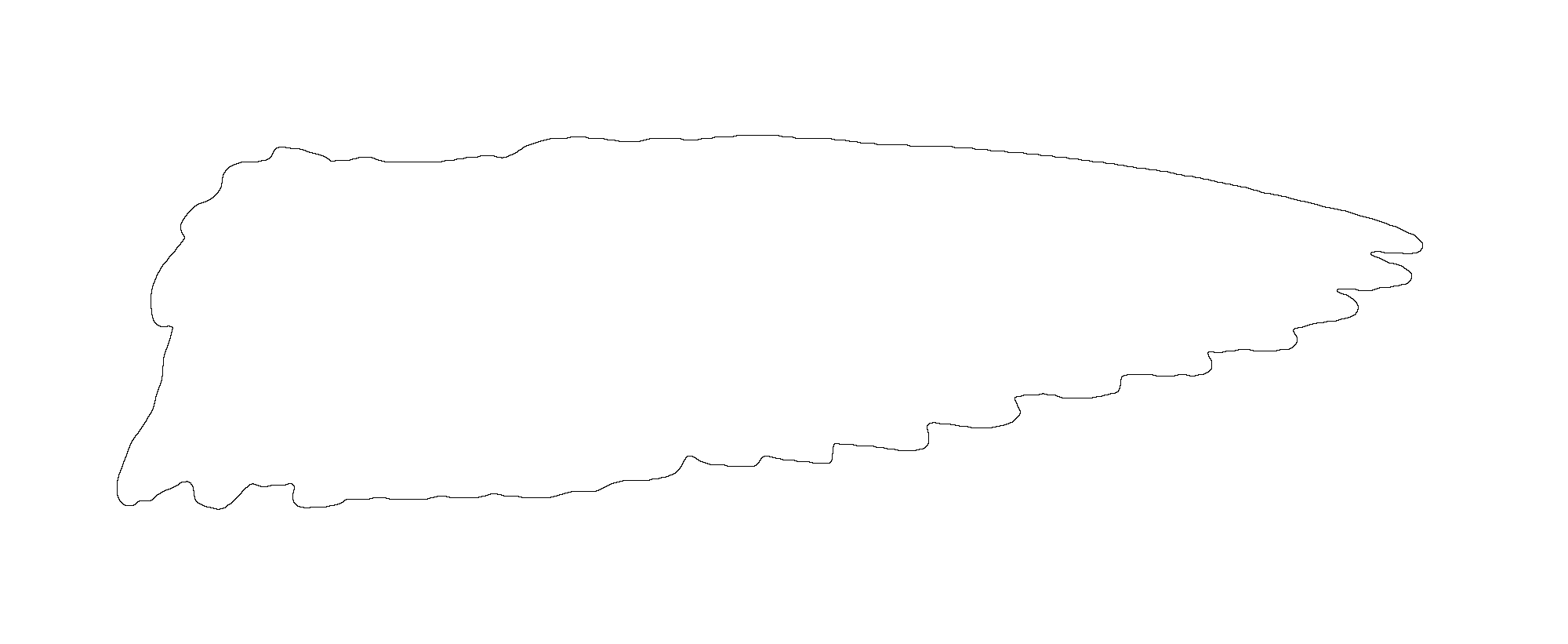

Supplement: Supplementary file 6 — Supplementary Data 4 [file 41467_2026_70692_MOESM6_ESM.zip › Supplementary Data 4/Anous_minutus.tif]

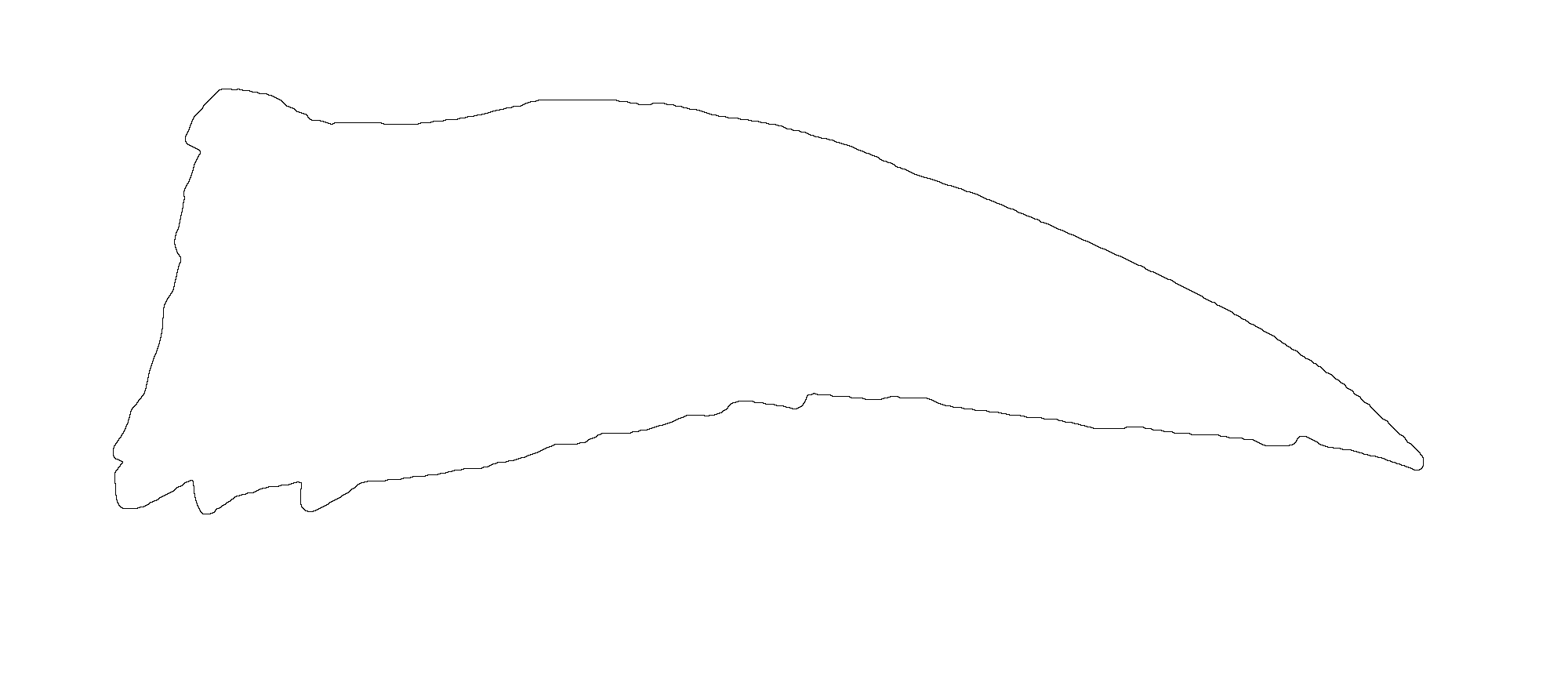

Supplement: Supplementary file 6 — Supplementary Data 4 [file 41467_2026_70692_MOESM6_ESM.zip › Supplementary Data 4/Anous_stolidus.tif]

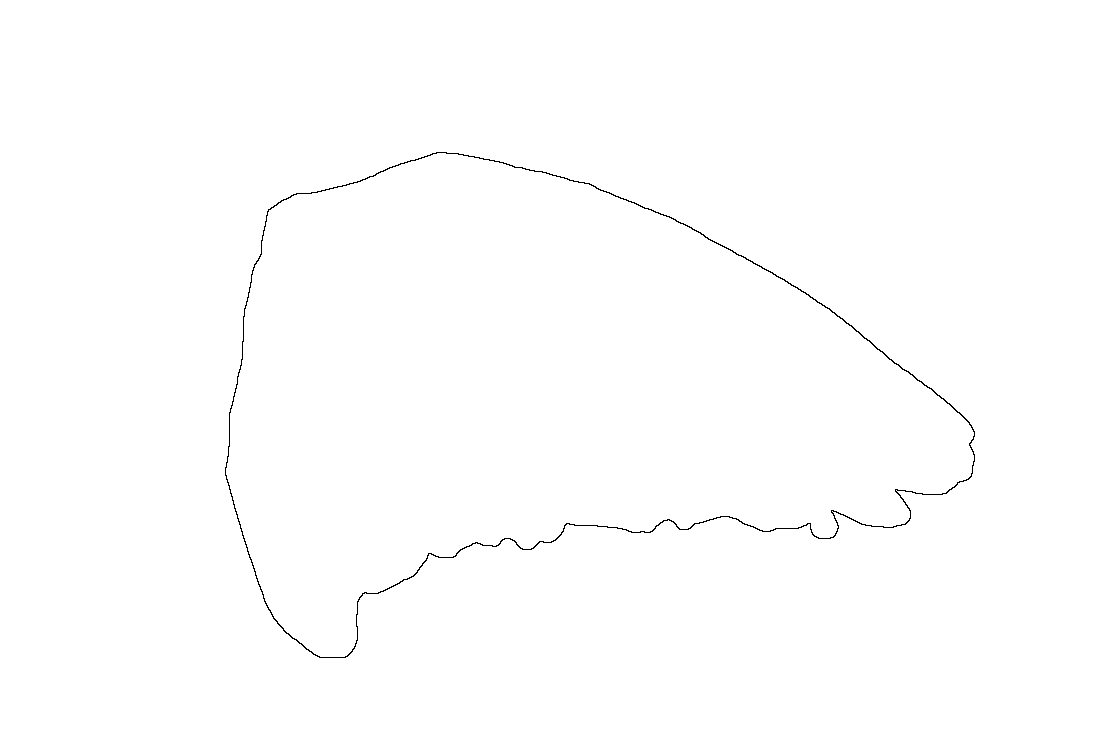

Supplement: Supplementary file 6 — Supplementary Data 4 [file 41467_2026_70692_MOESM6_ESM.zip › Supplementary Data 4/Anseranas_semipalmata.tif]

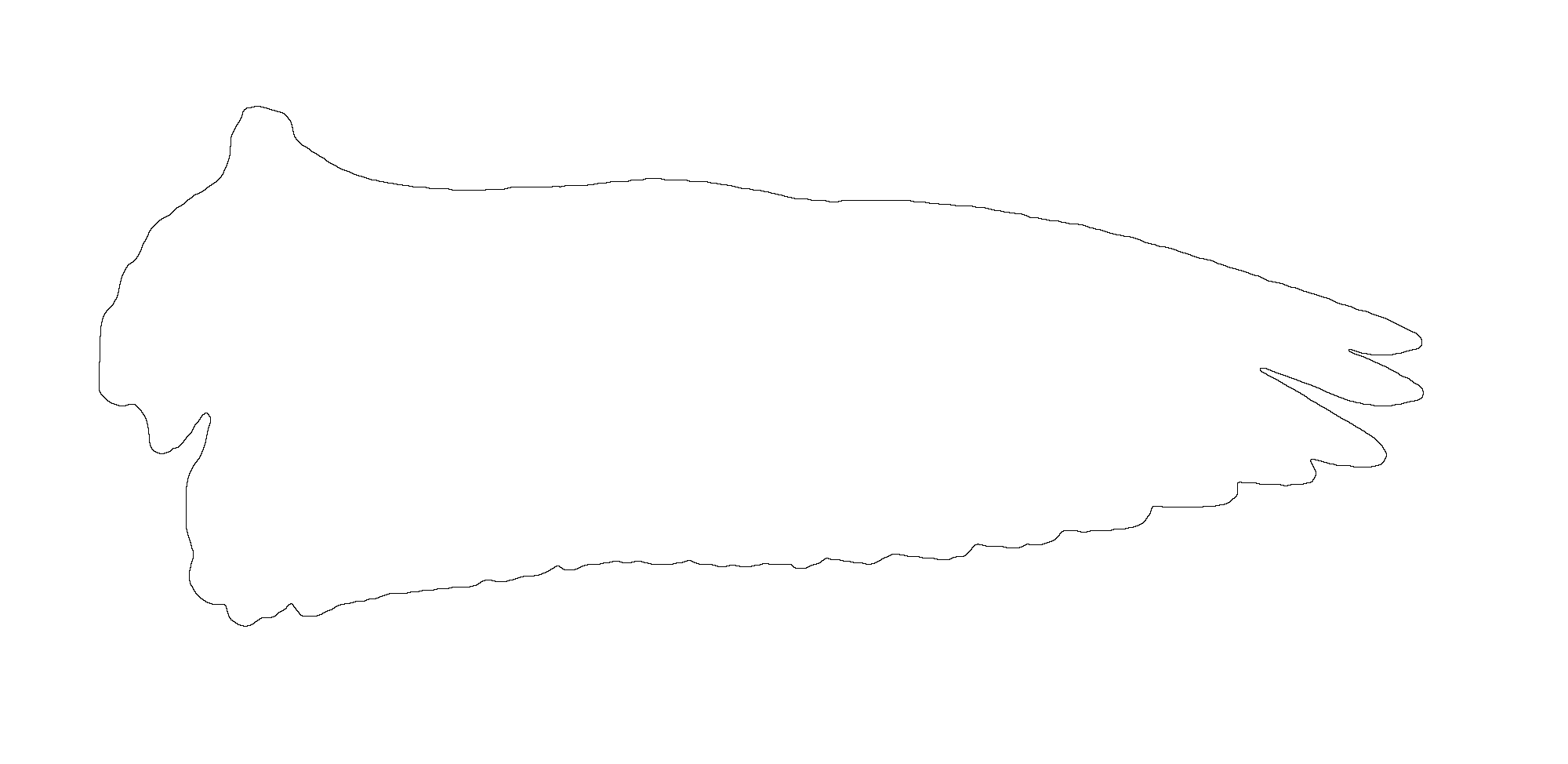

Supplement: Supplementary file 6 — Supplementary Data 4 [file 41467_2026_70692_MOESM6_ESM.zip › Supplementary Data 4/Anser_albifrons.tif]

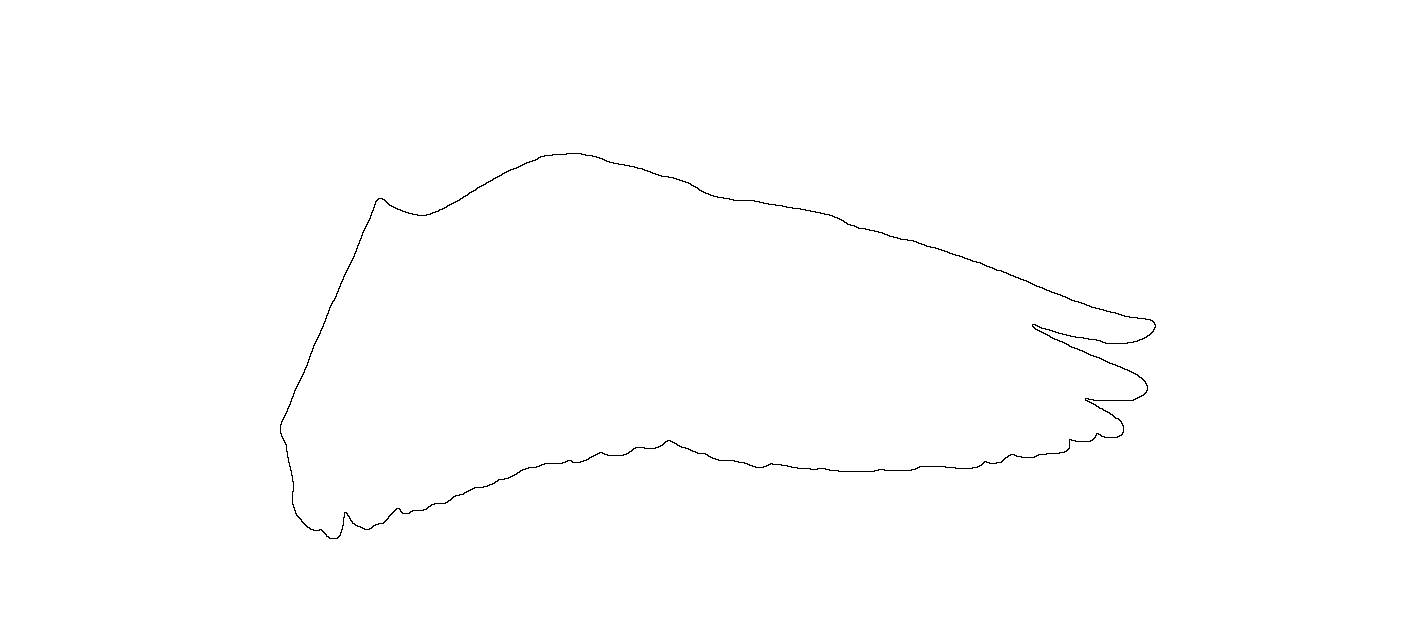

Supplement: Supplementary file 6 — Supplementary Data 4 [file 41467_2026_70692_MOESM6_ESM.zip › Supplementary Data 4/Anser_anser.tif]

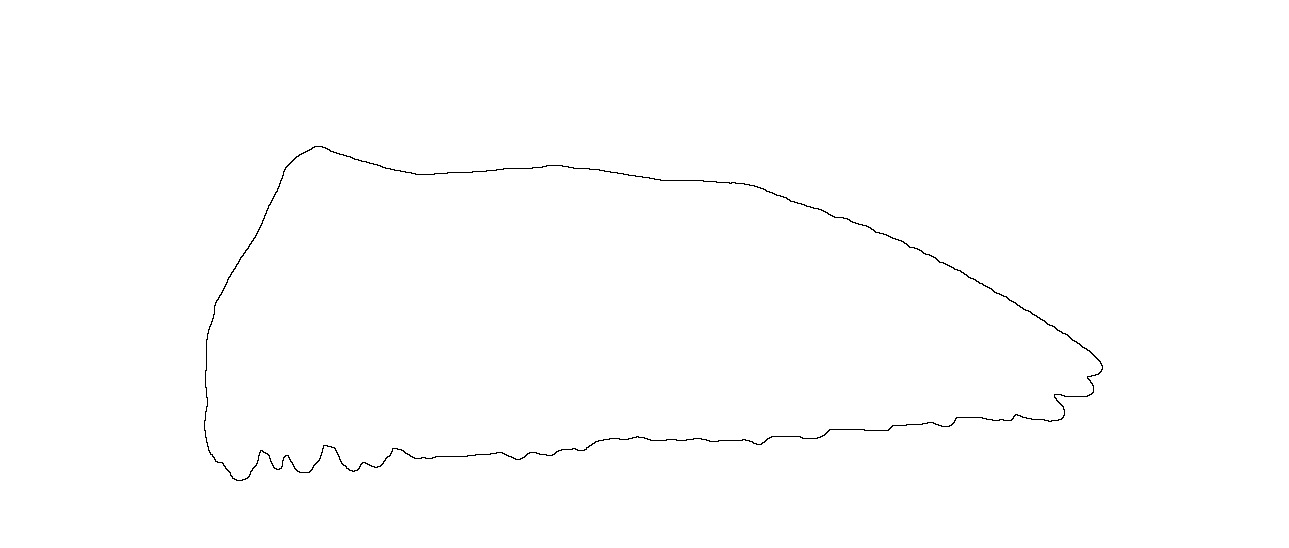

Supplement: Supplementary file 6 — Supplementary Data 4 [file 41467_2026_70692_MOESM6_ESM.zip › Supplementary Data 4/Anser_erythropus.tif]

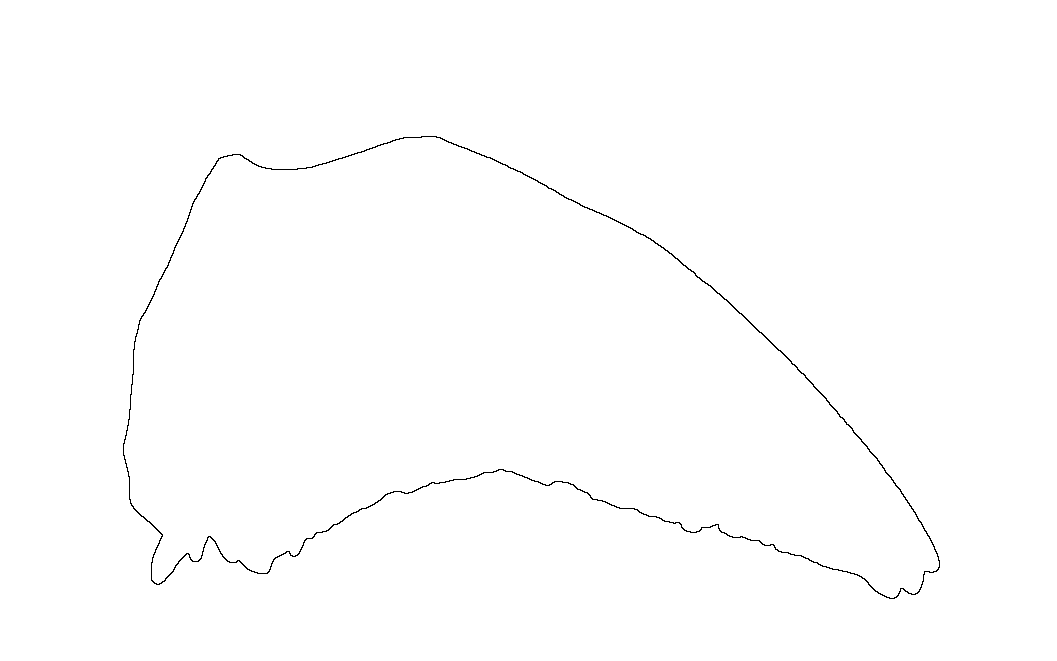

Supplement: Supplementary file 6 — Supplementary Data 4 [file 41467_2026_70692_MOESM6_ESM.zip › Supplementary Data 4/Anser_fabalis.tif]

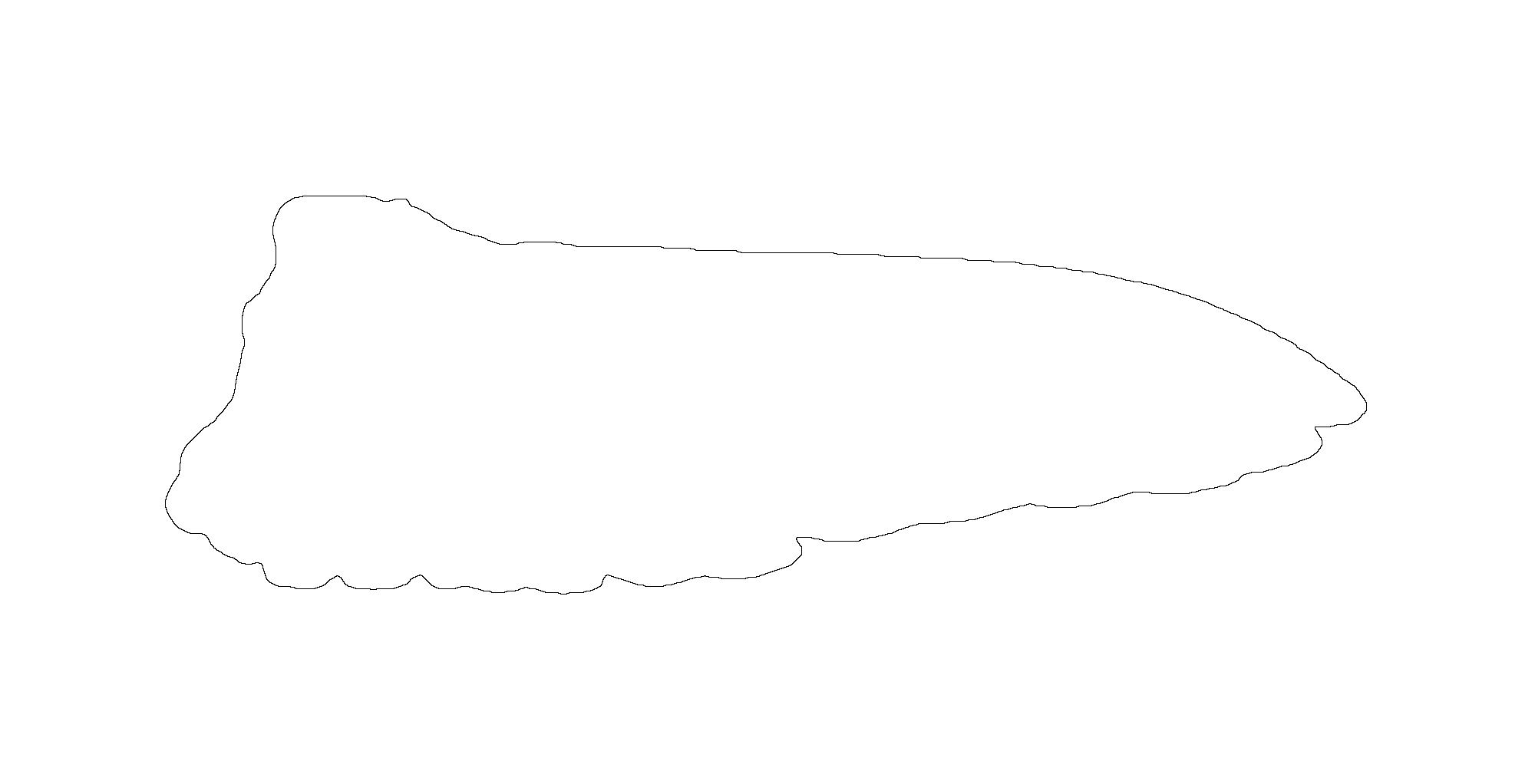

Supplement: Supplementary file 6 — Supplementary Data 4 [file 41467_2026_70692_MOESM6_ESM.zip › Supplementary Data 4/Anthracothorax_viridis.tif]

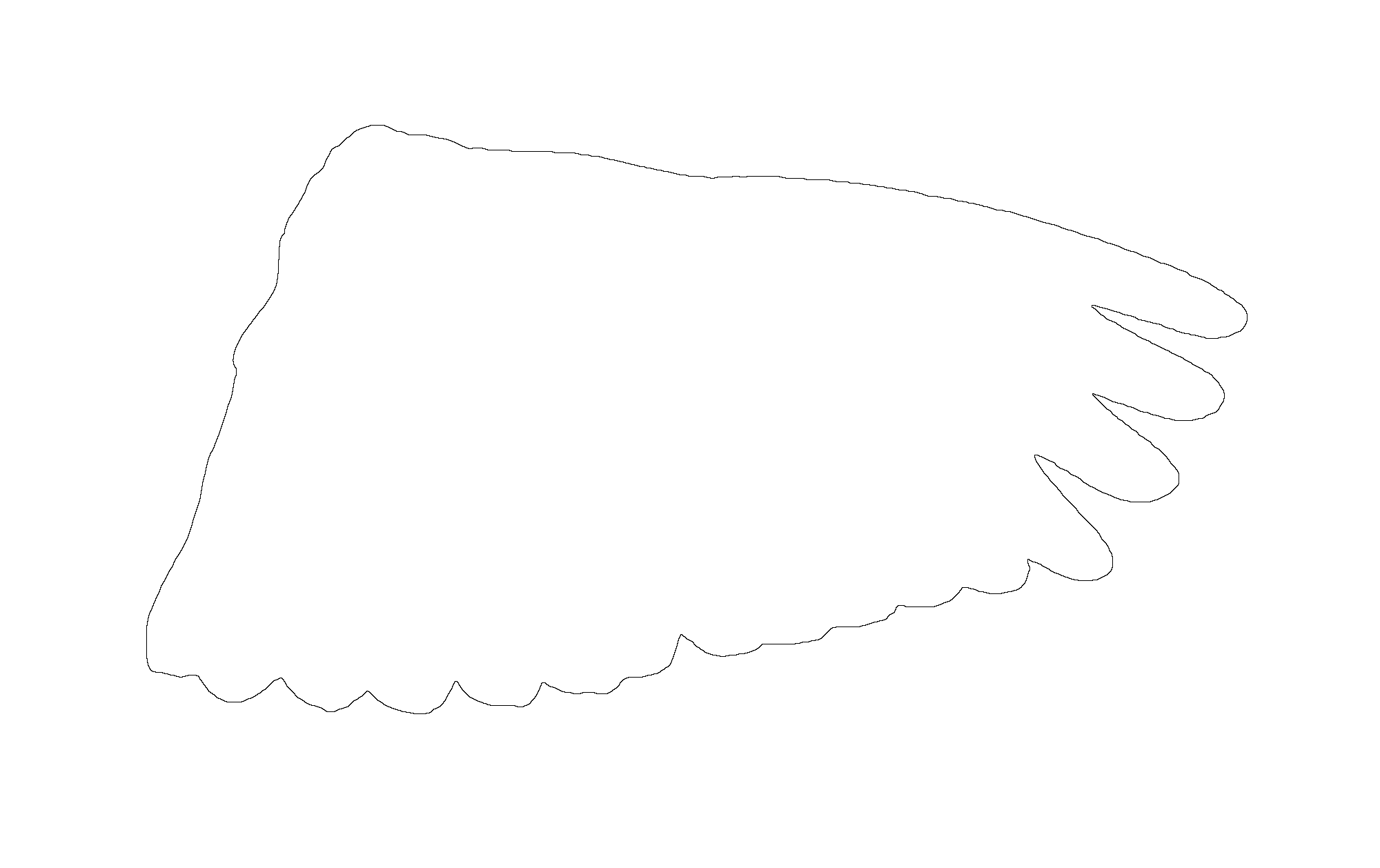

Supplement: Supplementary file 6 — Supplementary Data 4 [file 41467_2026_70692_MOESM6_ESM.zip › Supplementary Data 4/Anthus_lutescens.tif]

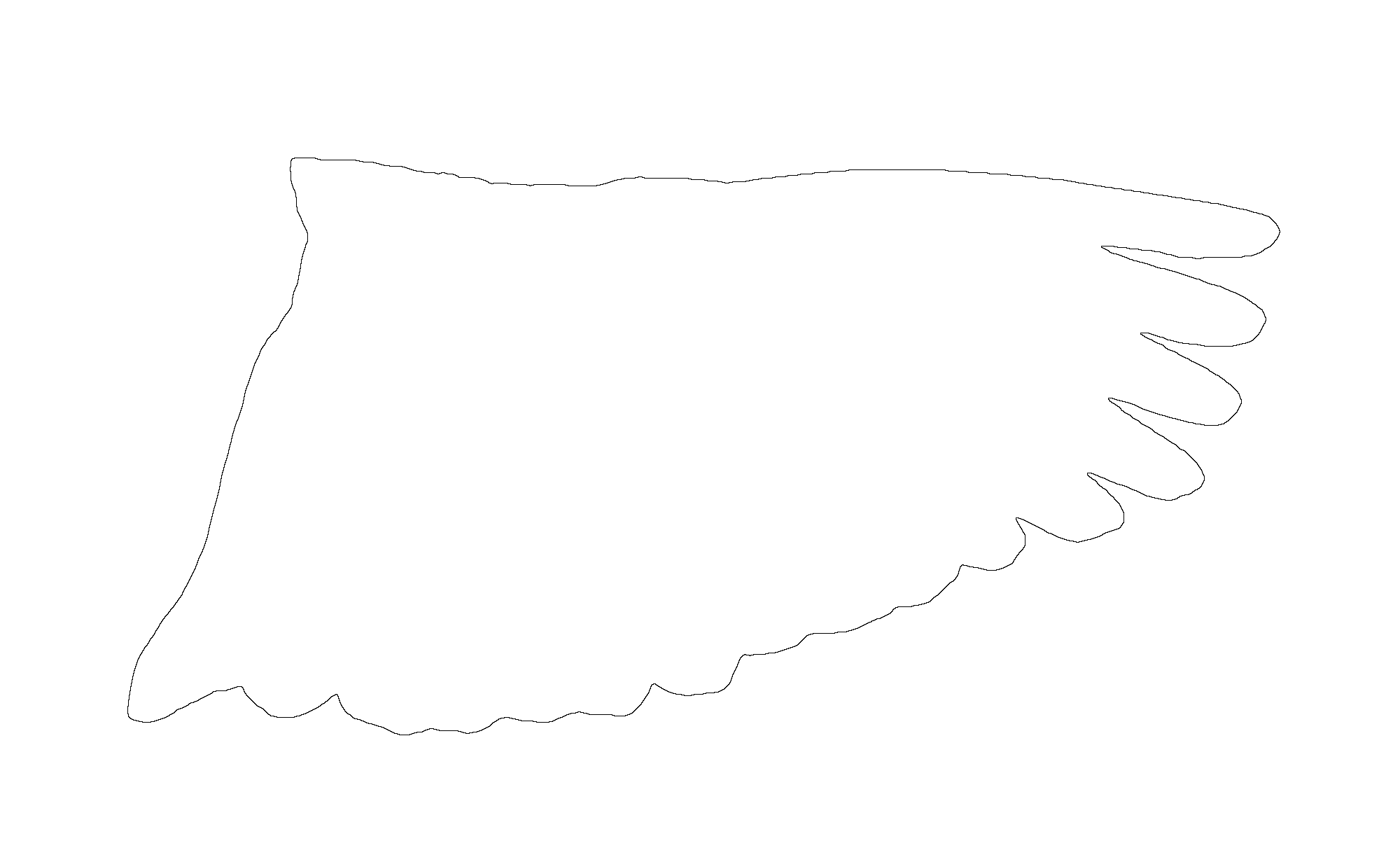

Supplement: Supplementary file 6 — Supplementary Data 4 [file 41467_2026_70692_MOESM6_ESM.zip › Supplementary Data 4/Anthus_novaeseelandiae.tif]

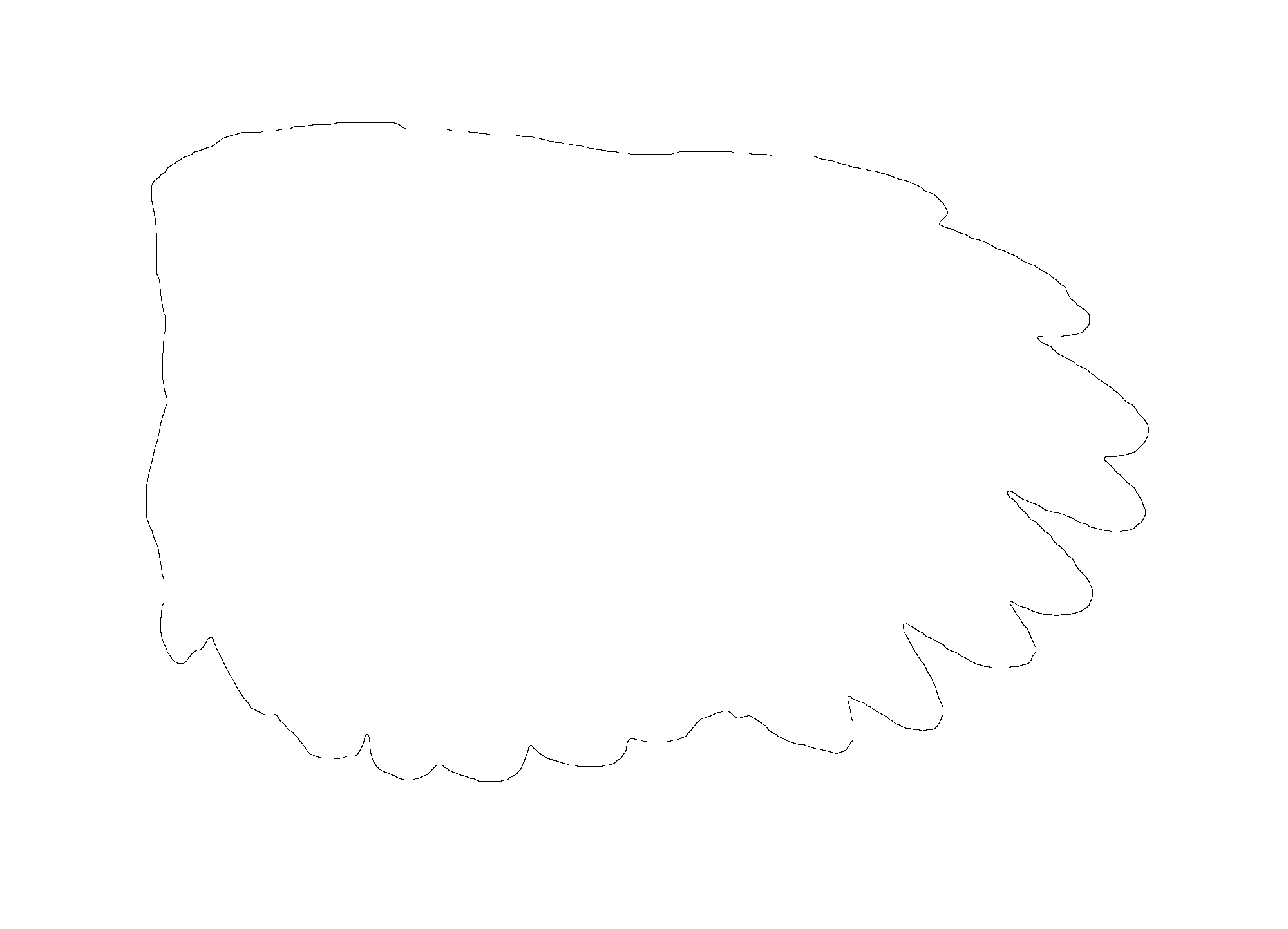

Supplement: Supplementary file 6 — Supplementary Data 4 [file 41467_2026_70692_MOESM6_ESM.zip › Supplementary Data 4/Aphelocoma_californica.tif]

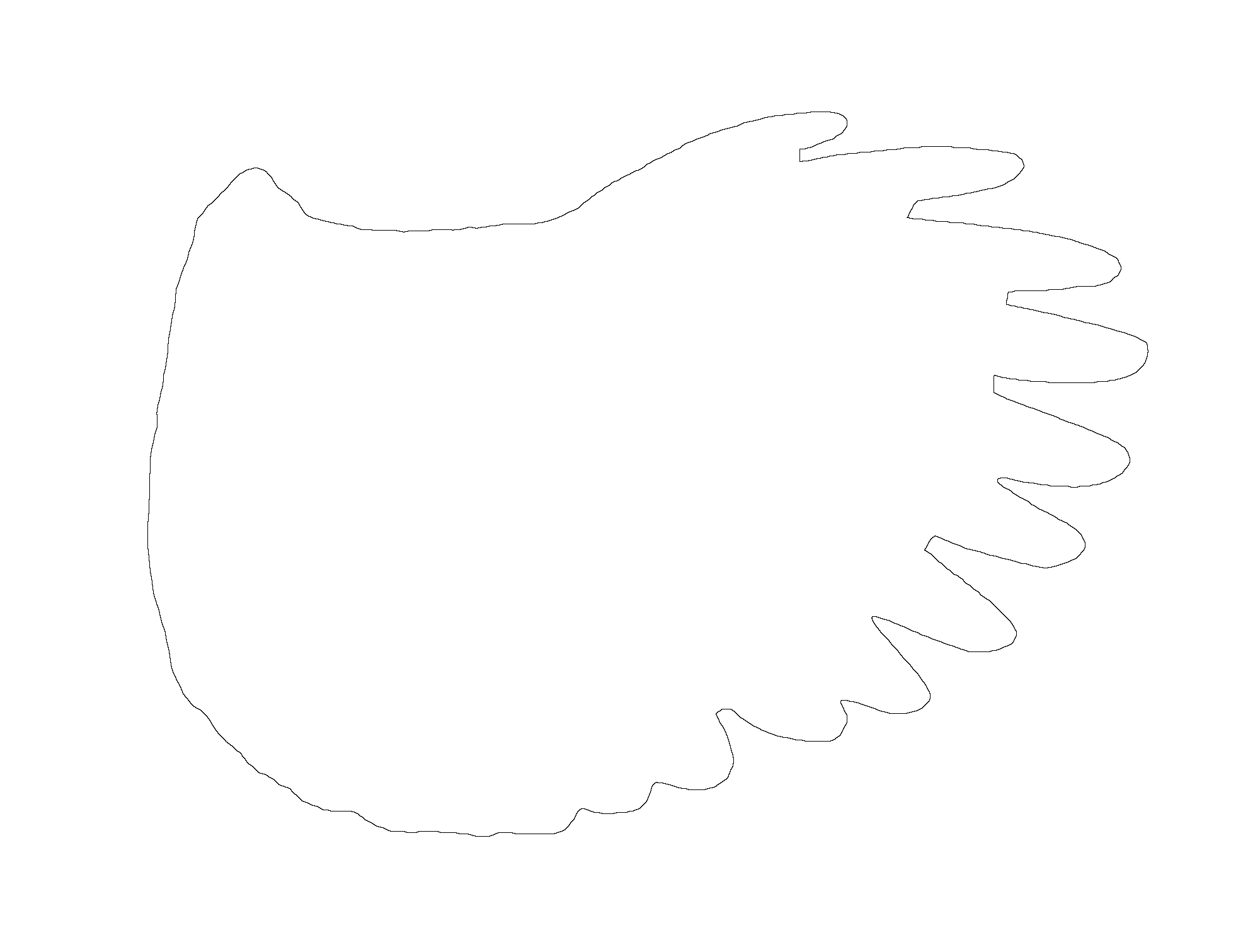

Supplement: Supplementary file 6 — Supplementary Data 4 [file 41467_2026_70692_MOESM6_ESM.zip › Supplementary Data 4/Aphelocoma_ultramarina.tif]

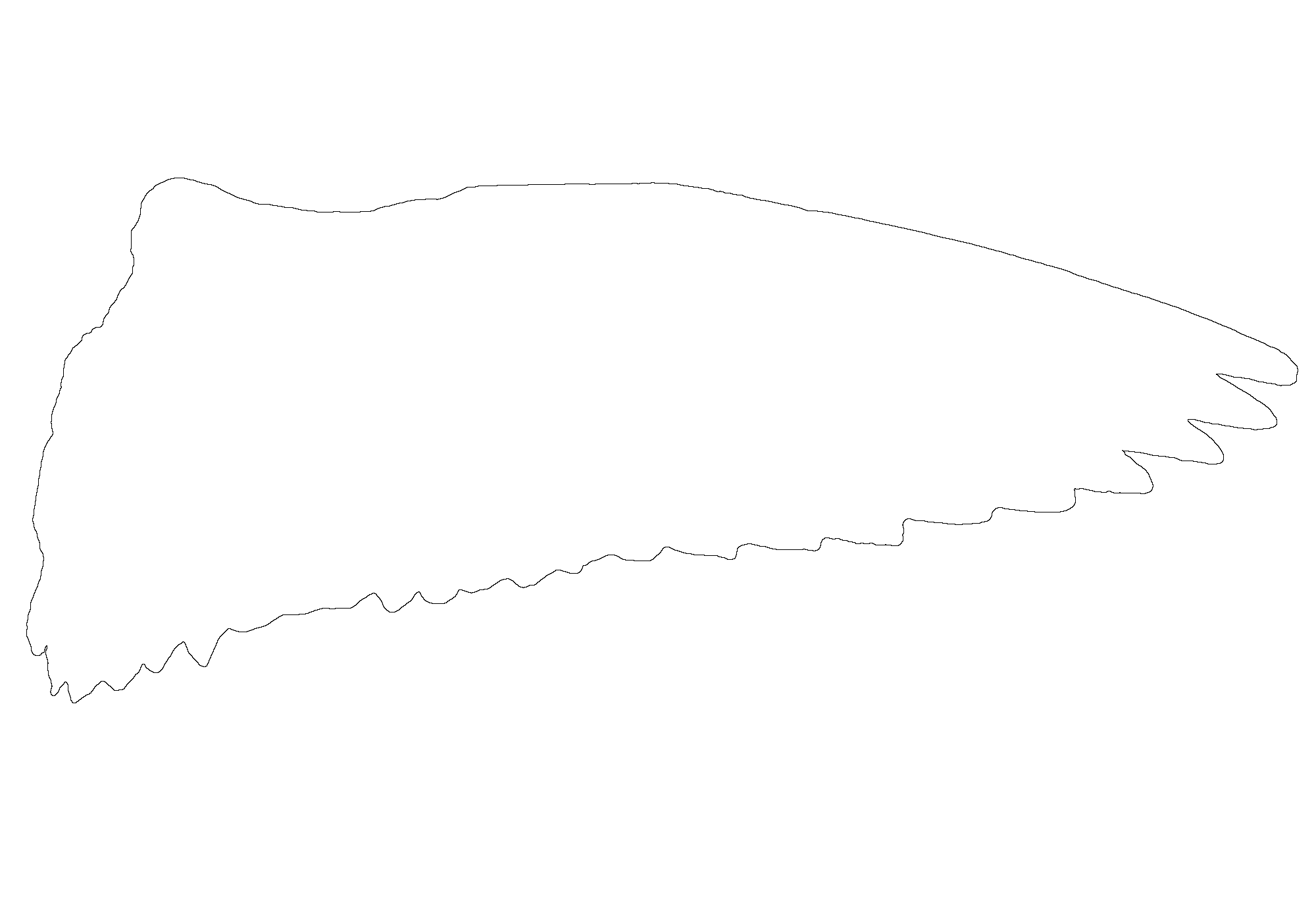

Supplement: Supplementary file 6 — Supplementary Data 4 [file 41467_2026_70692_MOESM6_ESM.zip › Supplementary Data 4/Aphriza_virgata.tif]

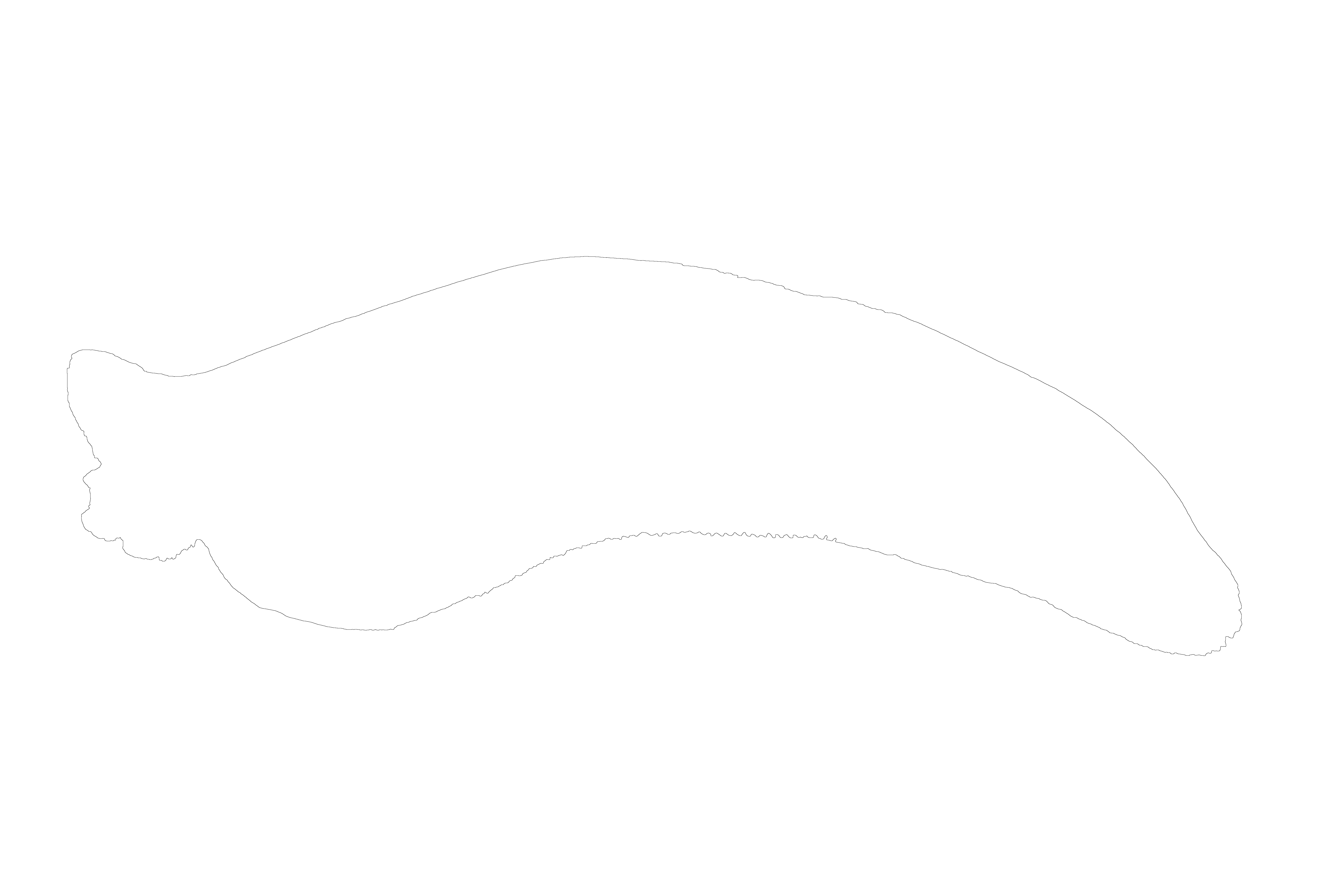

Supplement: Supplementary file 6 — Supplementary Data 4 [file 41467_2026_70692_MOESM6_ESM.zip › Supplementary Data 4/Aptenodytes_patagonicus.tif]

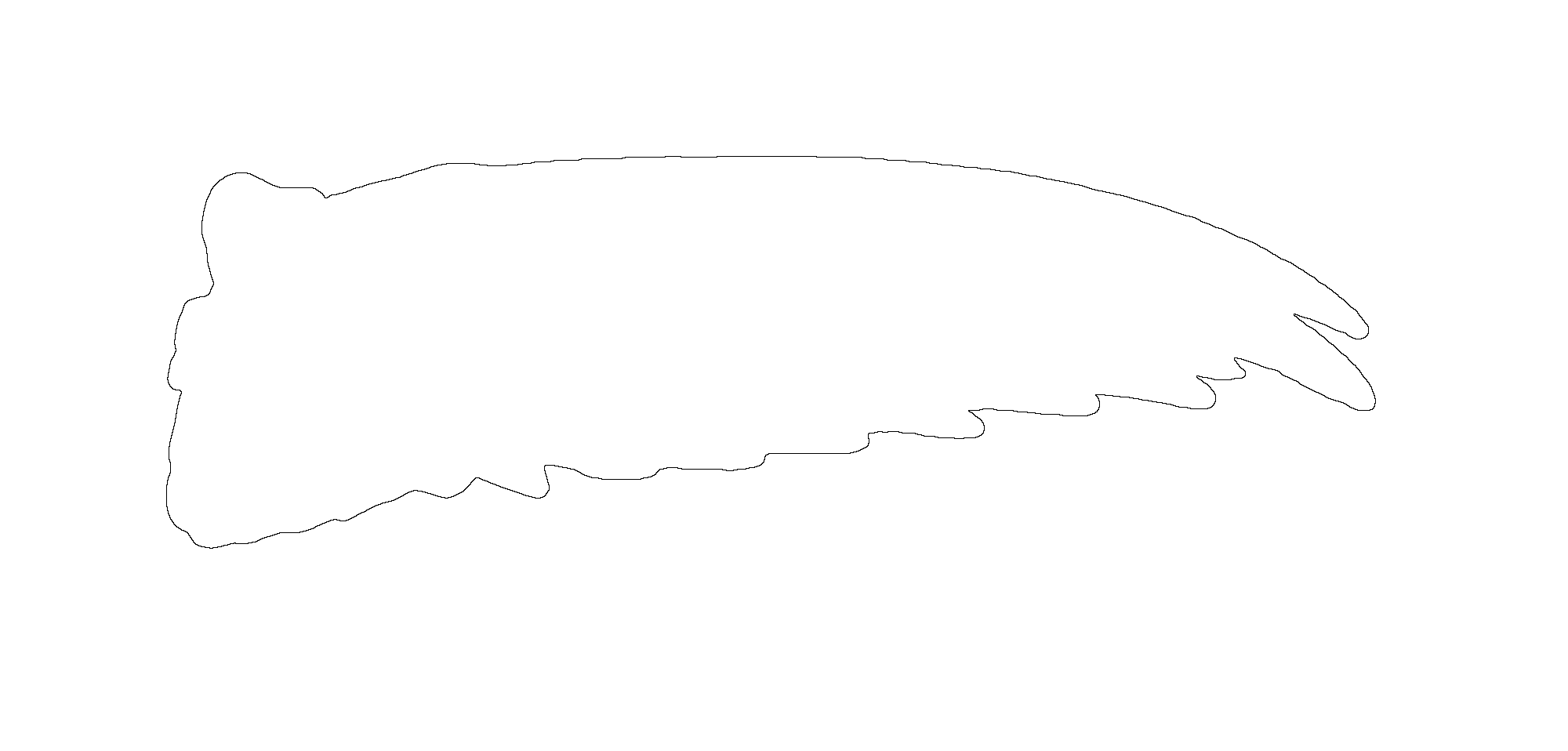

Supplement: Supplementary file 6 — Supplementary Data 4 [file 41467_2026_70692_MOESM6_ESM.zip › Supplementary Data 4/Apus_apus.tif]

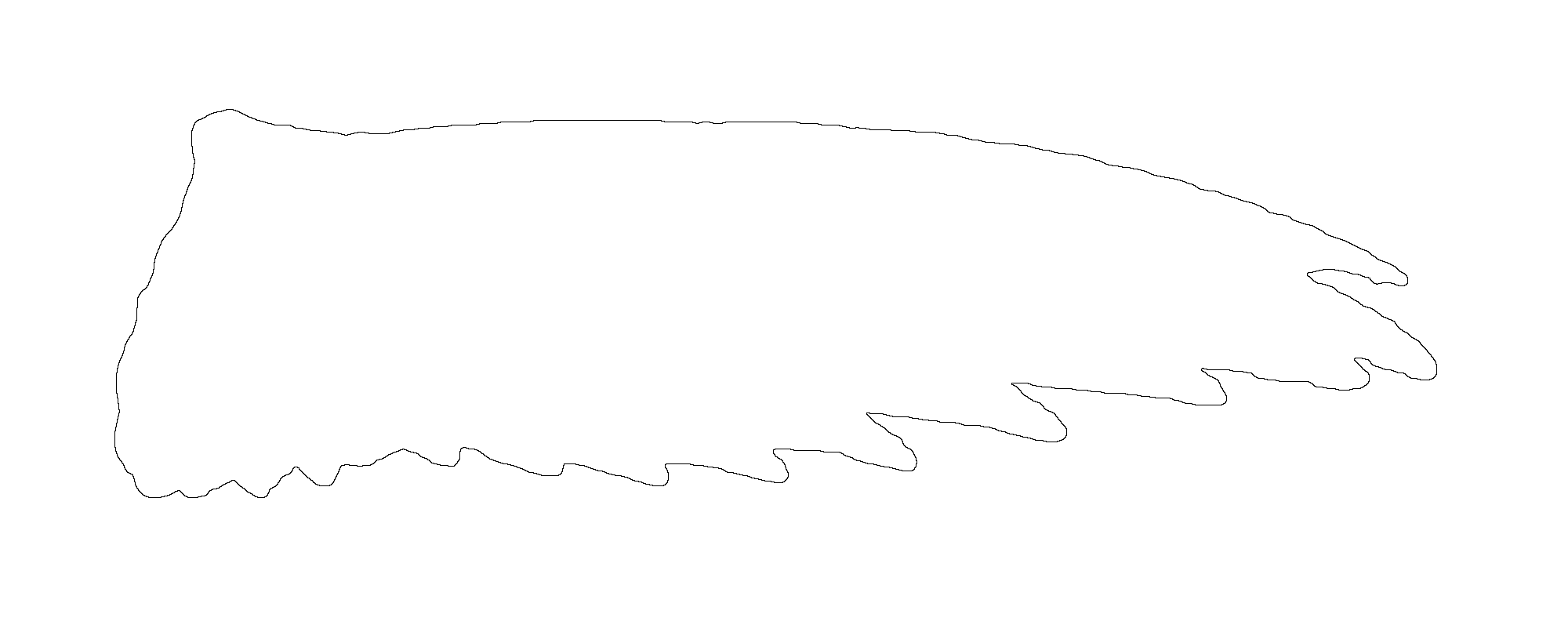

Supplement: Supplementary file 6 — Supplementary Data 4 [file 41467_2026_70692_MOESM6_ESM.zip › Supplementary Data 4/Apus_pacificus.tif]

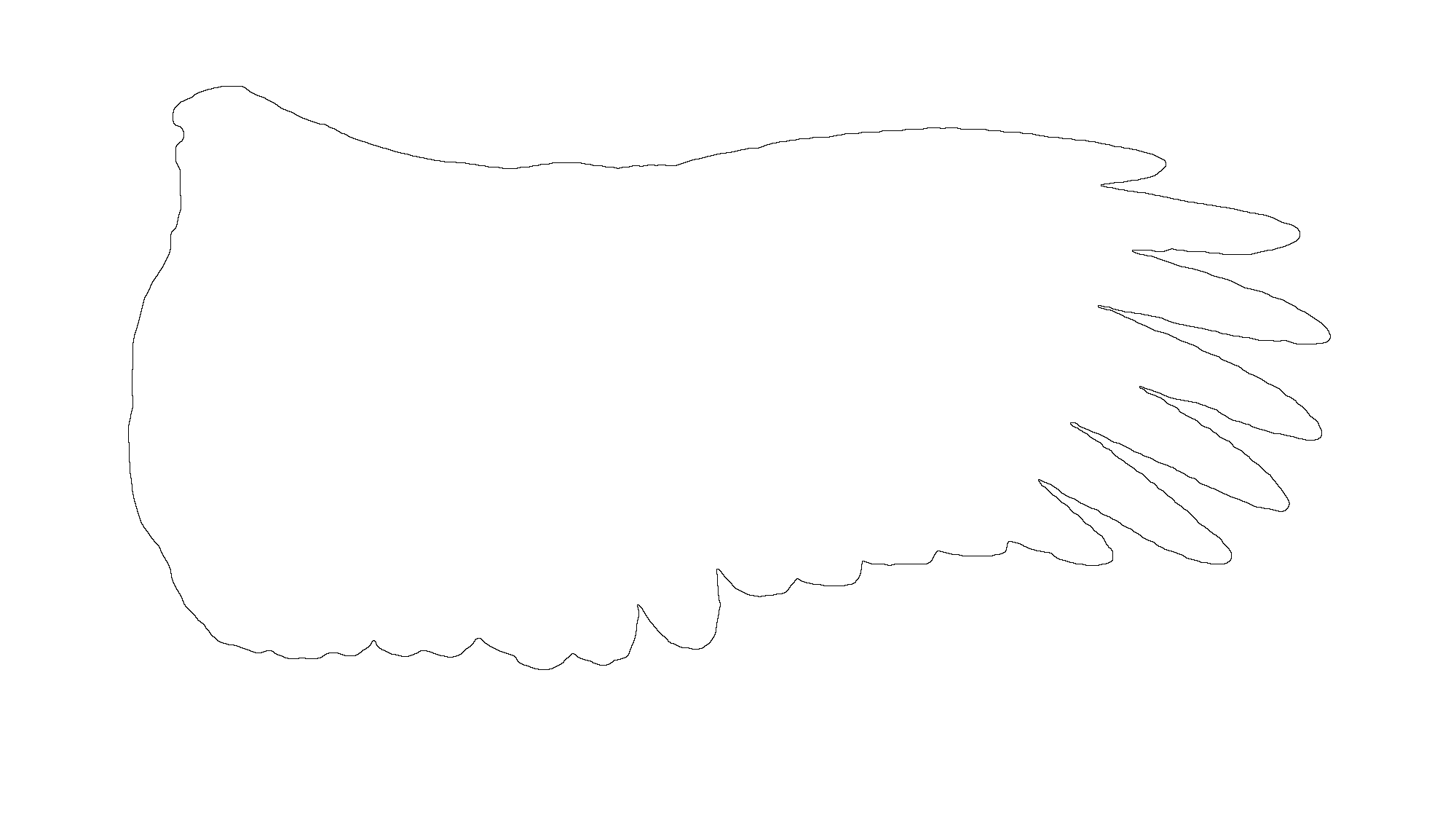

Supplement: Supplementary file 6 — Supplementary Data 4 [file 41467_2026_70692_MOESM6_ESM.zip › Supplementary Data 4/Aquila_audax.tif]

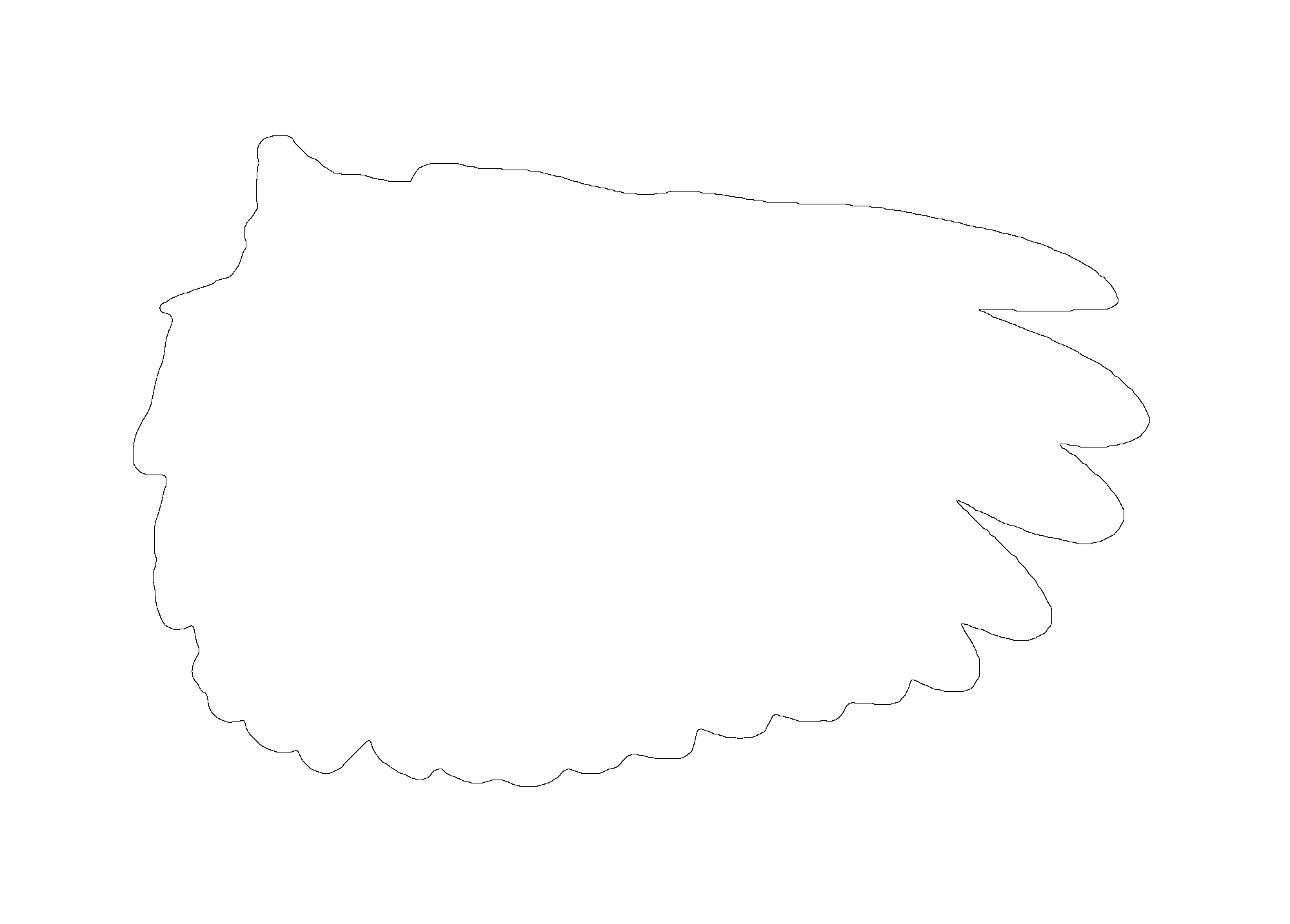

Supplement: Supplementary file 6 — Supplementary Data 4 [file 41467_2026_70692_MOESM6_ESM.zip › Supplementary Data 4/Arachnothera_longirostra.tif]

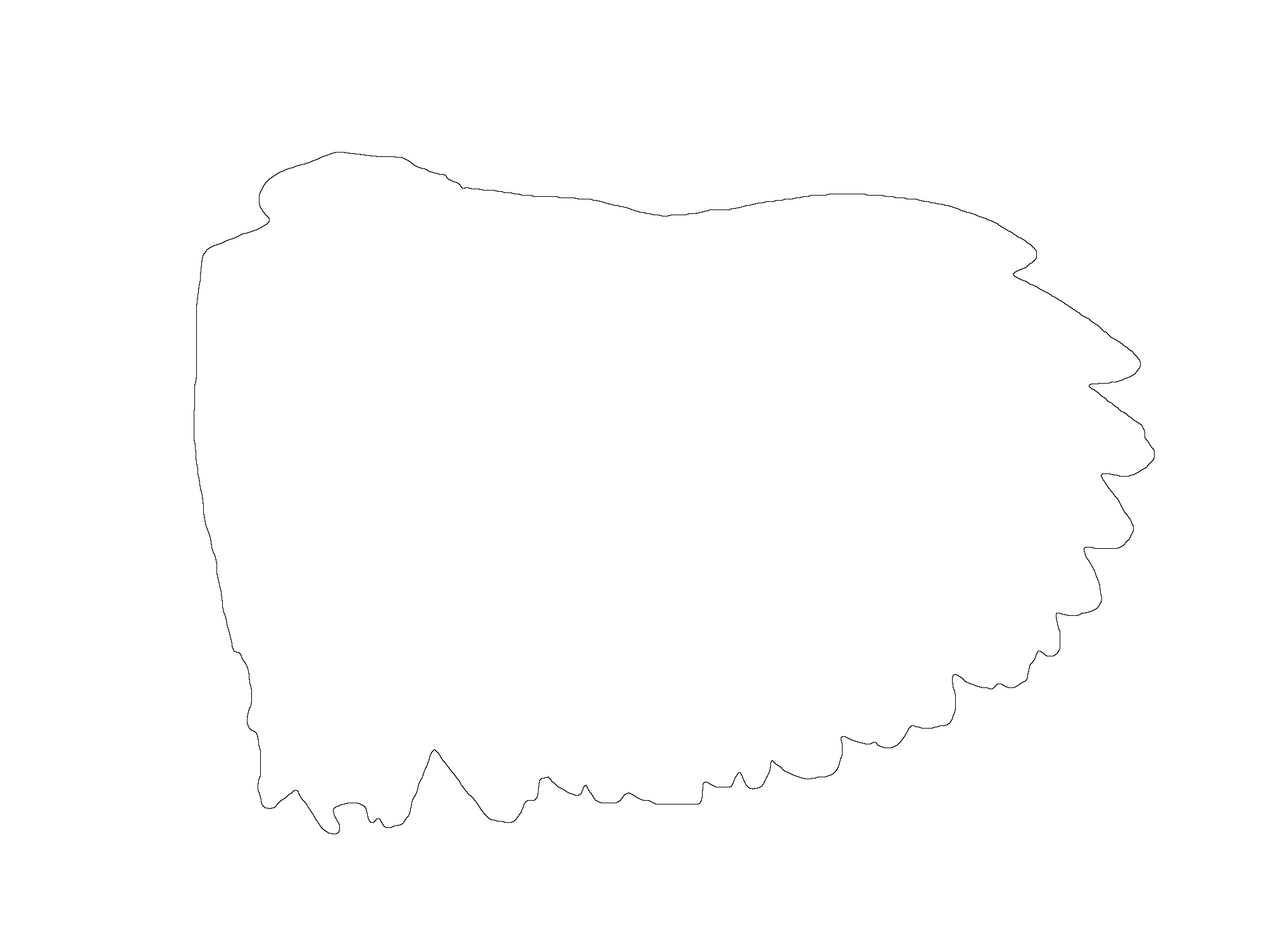

Supplement: Supplementary file 6 — Supplementary Data 4 [file 41467_2026_70692_MOESM6_ESM.zip › Supplementary Data 4/Aramides_cajanea.tif]

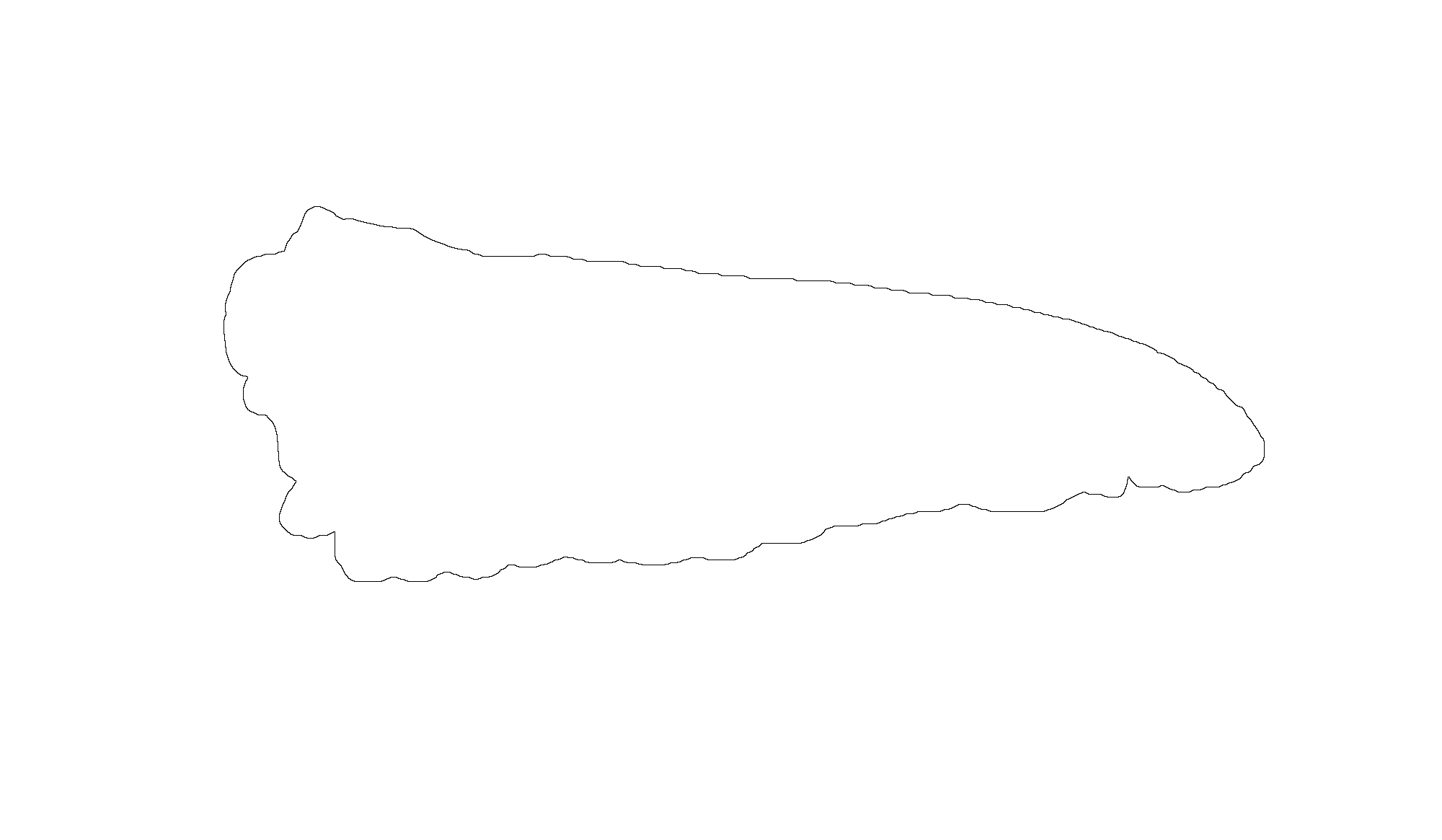

Supplement: Supplementary file 6 — Supplementary Data 4 [file 41467_2026_70692_MOESM6_ESM.zip › Supplementary Data 4/Archilochus_colubris.tif]

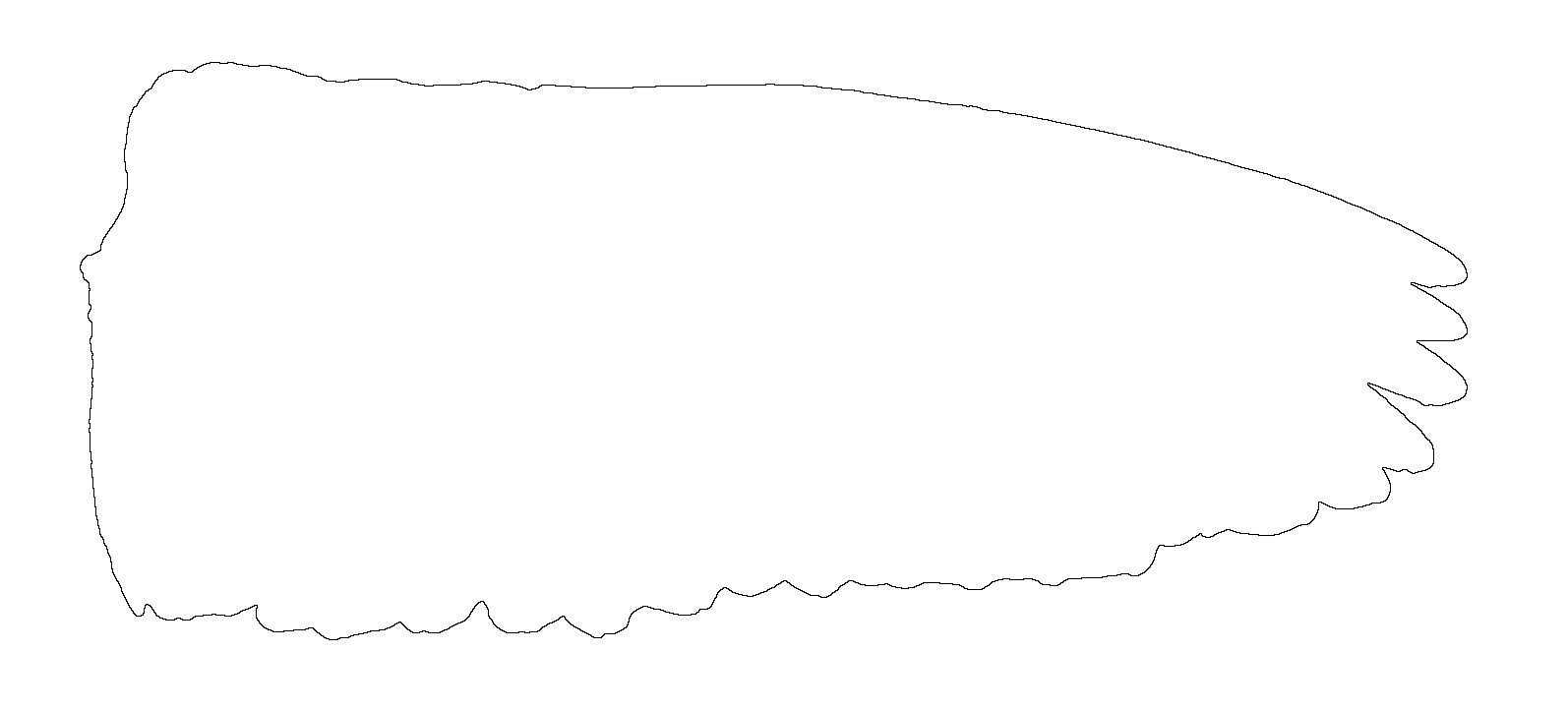

Supplement: Supplementary file 6 — Supplementary Data 4 [file 41467_2026_70692_MOESM6_ESM.zip › Supplementary Data 4/Ardea_cinerea.tif]

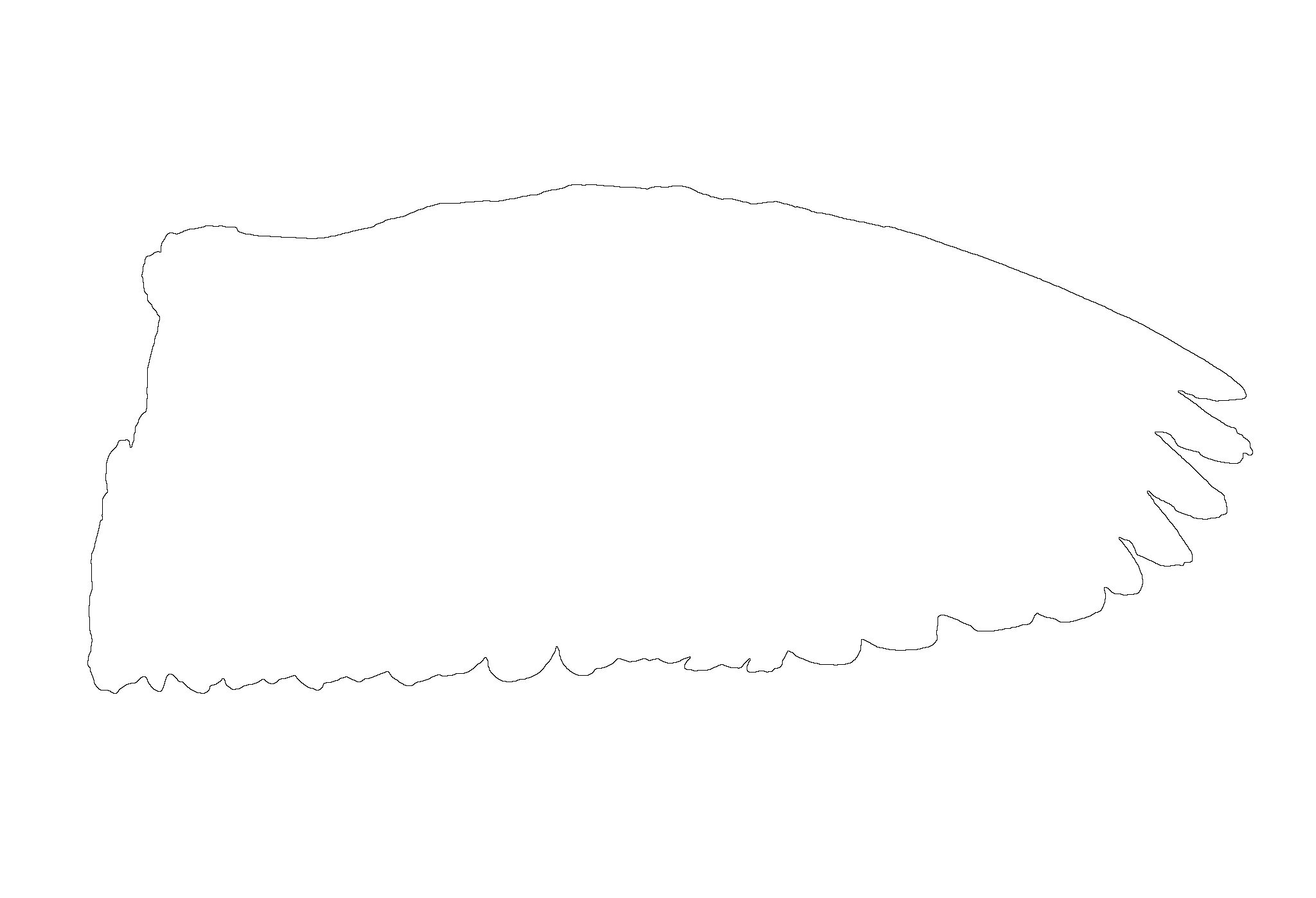

Supplement: Supplementary file 6 — Supplementary Data 4 [file 41467_2026_70692_MOESM6_ESM.zip › Supplementary Data 4/Ardea_herodias.tif]

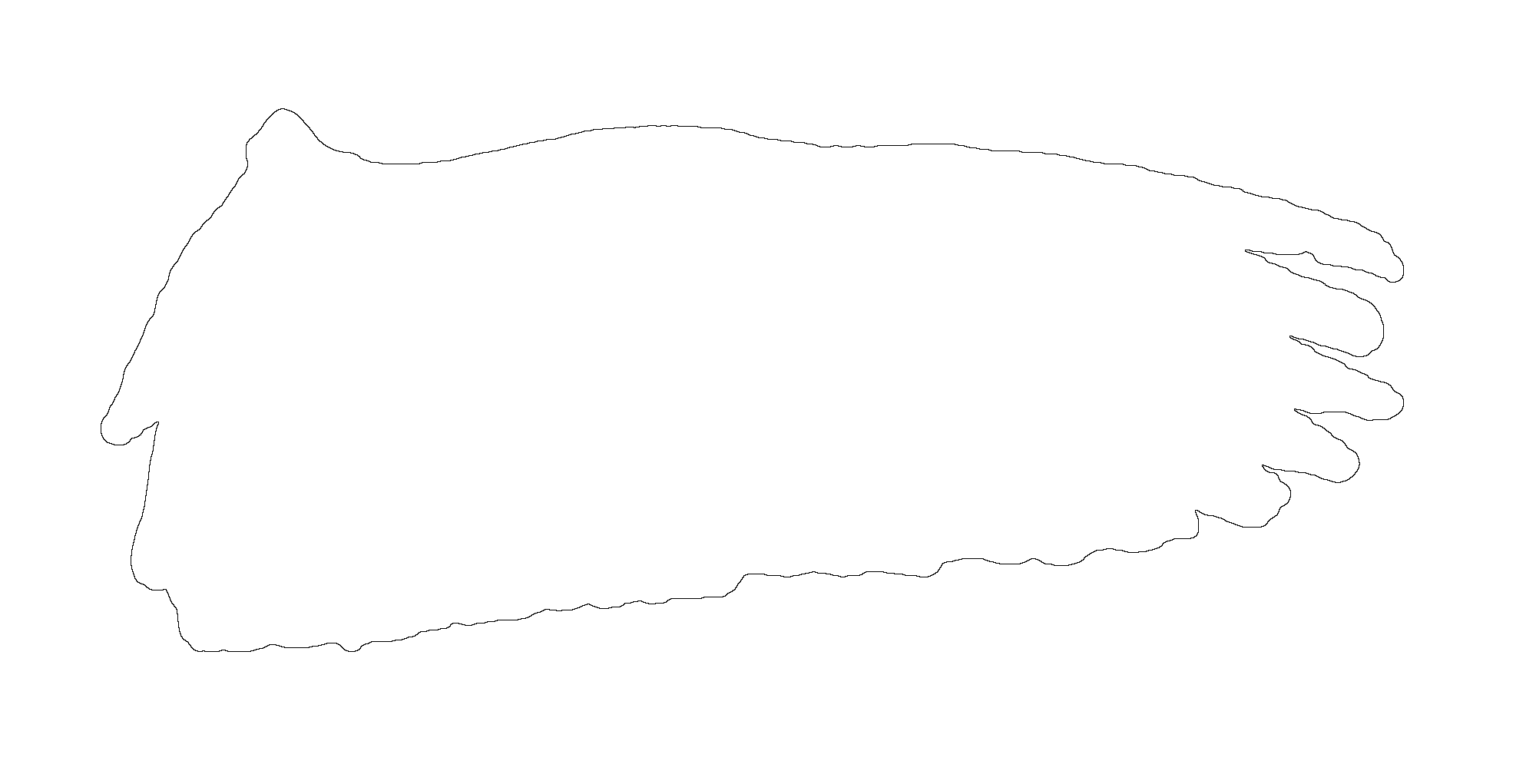

Supplement: Supplementary file 6 — Supplementary Data 4 [file 41467_2026_70692_MOESM6_ESM.zip › Supplementary Data 4/Ardea_pacifica.tif]

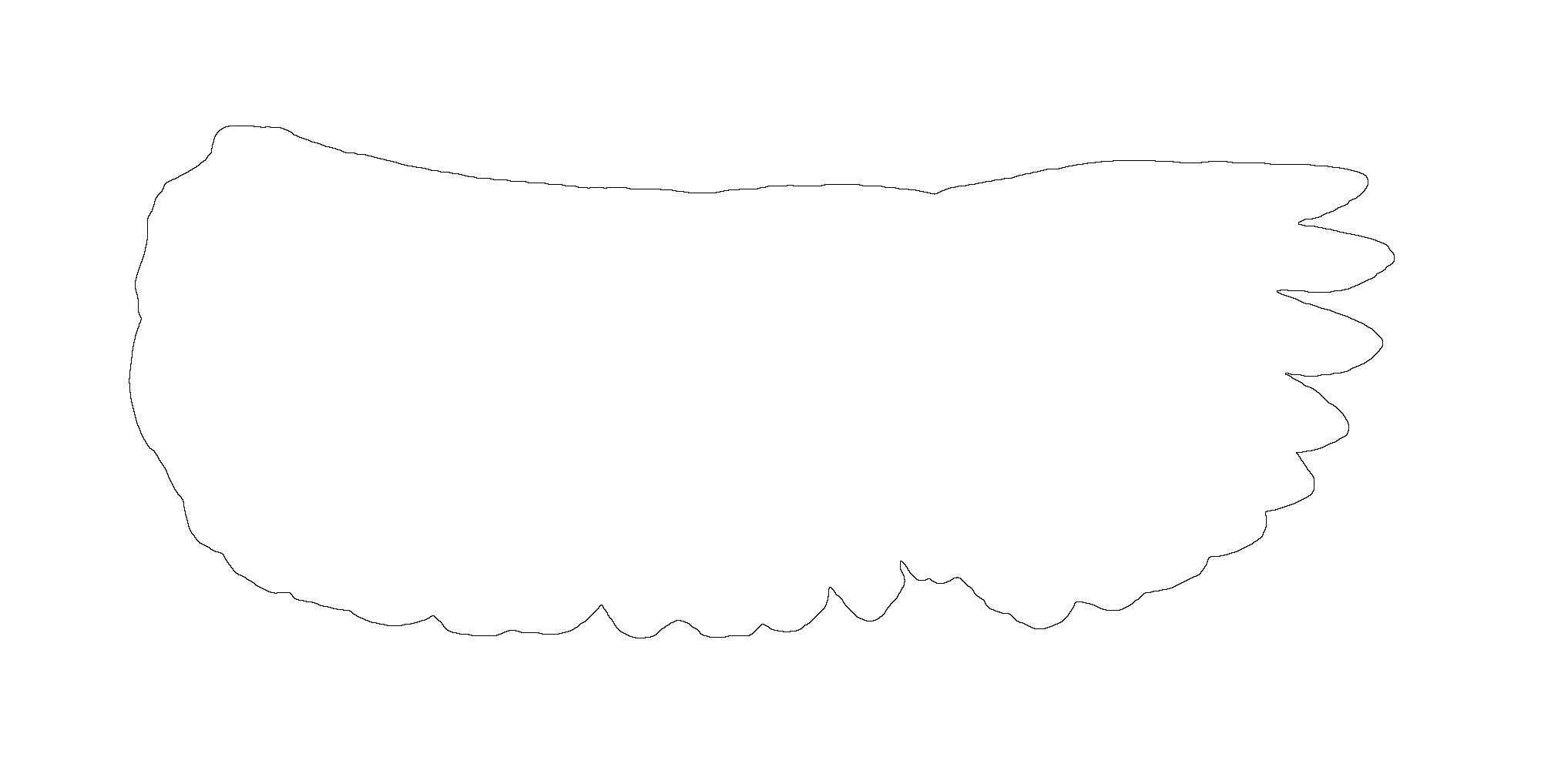

Supplement: Supplementary file 6 — Supplementary Data 4 [file 41467_2026_70692_MOESM6_ESM.zip › Supplementary Data 4/Ardea_purpurea.tif]

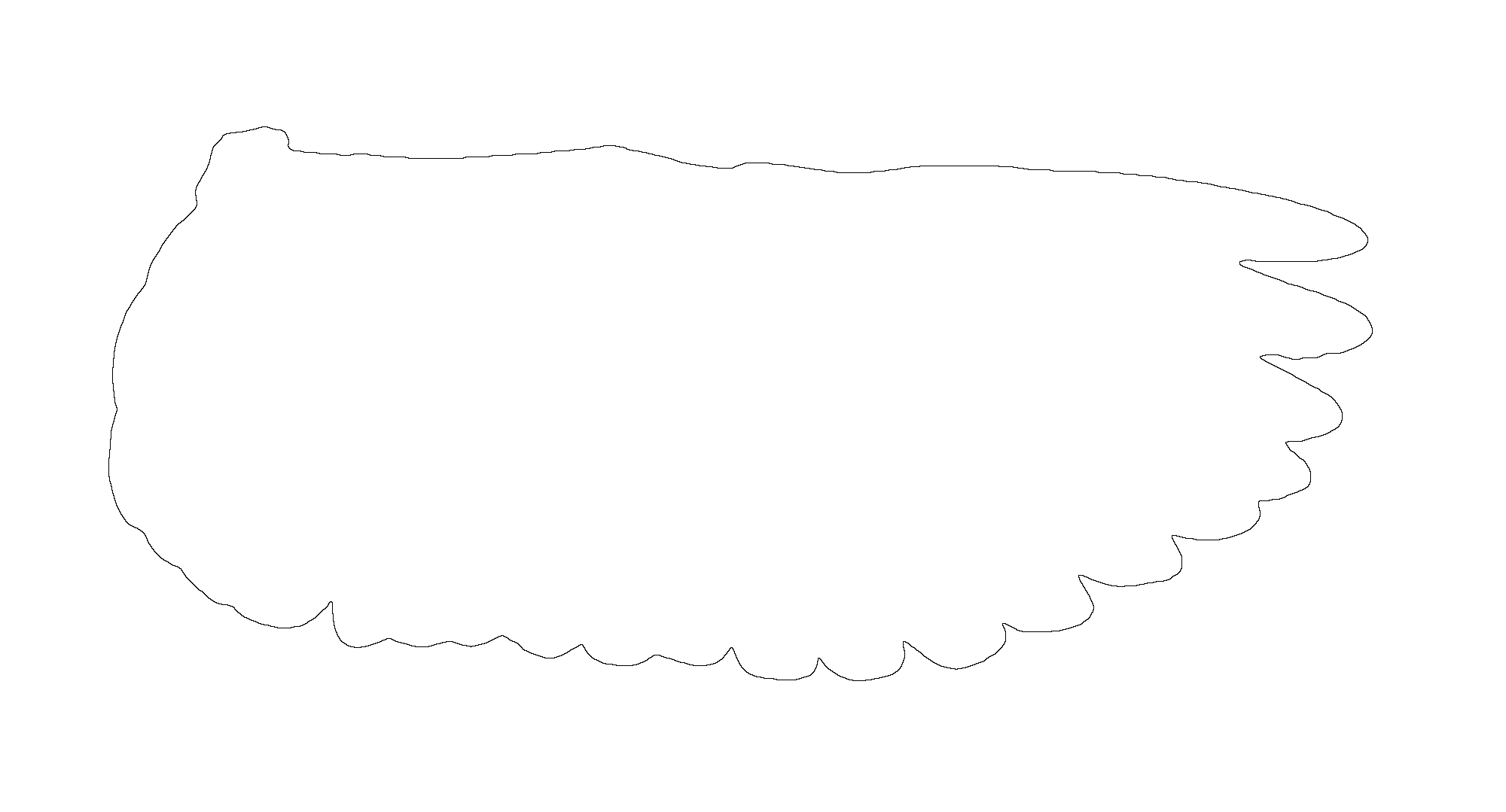

Supplement: Supplementary file 6 — Supplementary Data 4 [file 41467_2026_70692_MOESM6_ESM.zip › Supplementary Data 4/Ardeola_bacchus.tif]

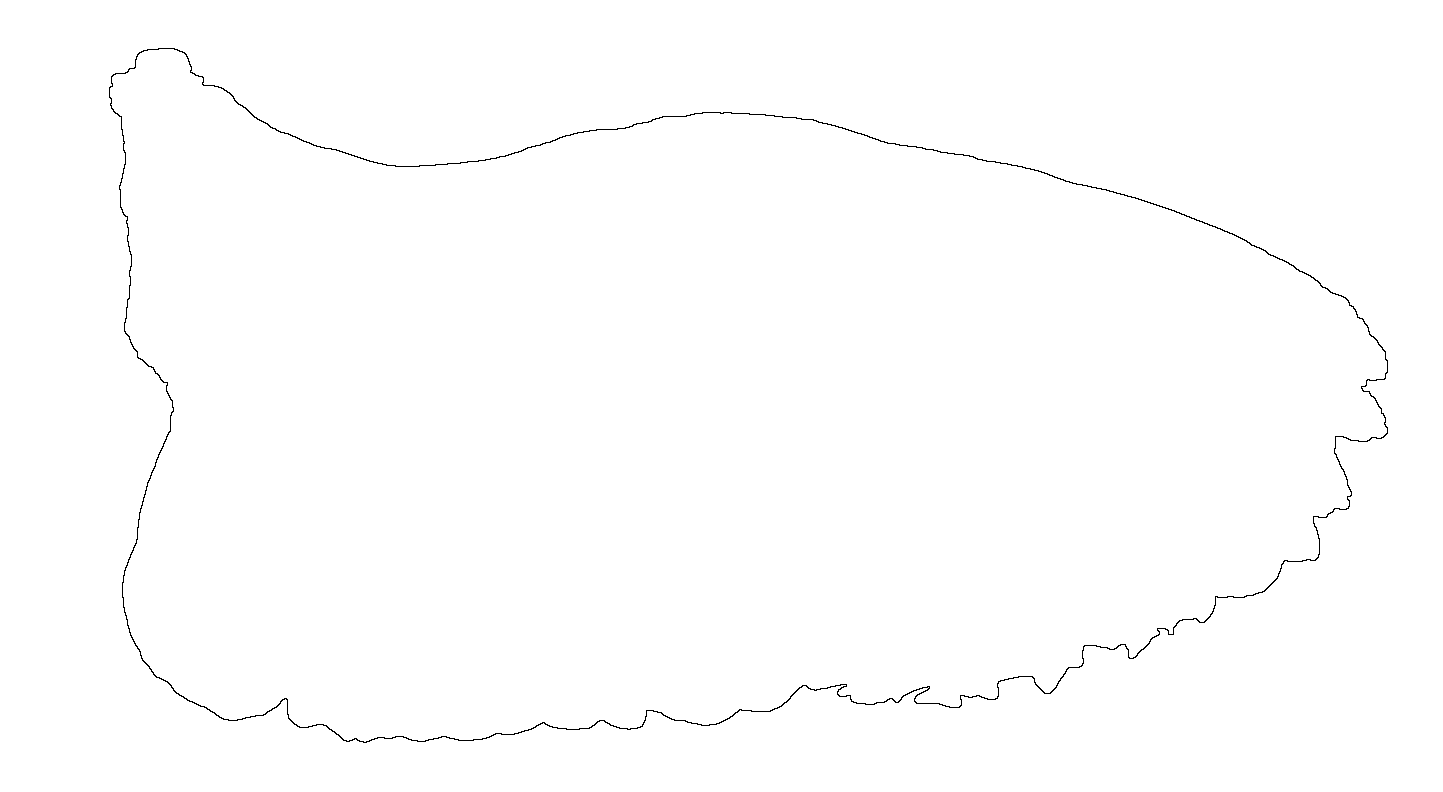

Supplement: Supplementary file 6 — Supplementary Data 4 [file 41467_2026_70692_MOESM6_ESM.zip › Supplementary Data 4/Ardeola_grayii.tif]

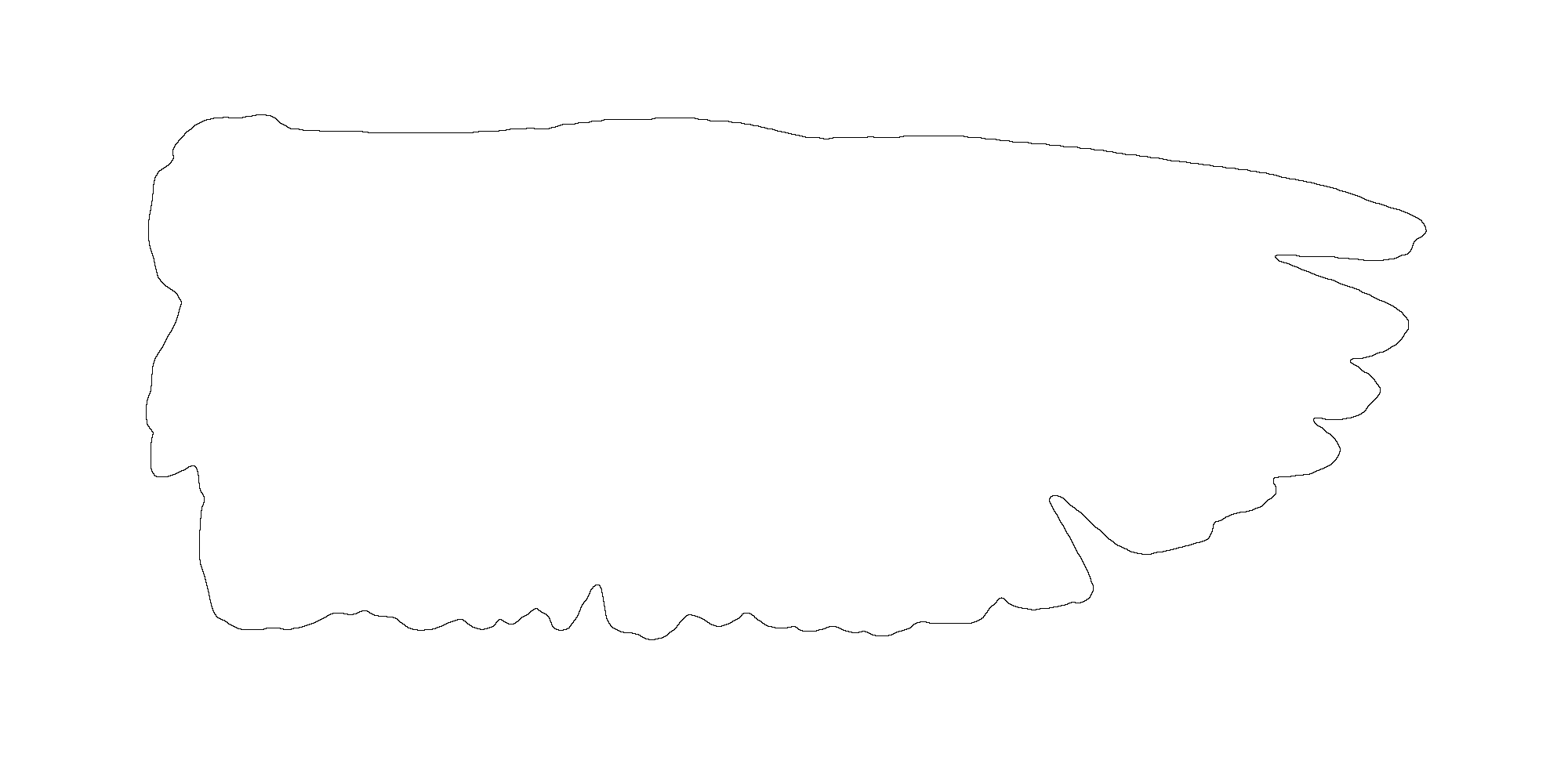

Supplement: Supplementary file 6 — Supplementary Data 4 [file 41467_2026_70692_MOESM6_ESM.zip › Supplementary Data 4/Ardeola_ralloides.tif]

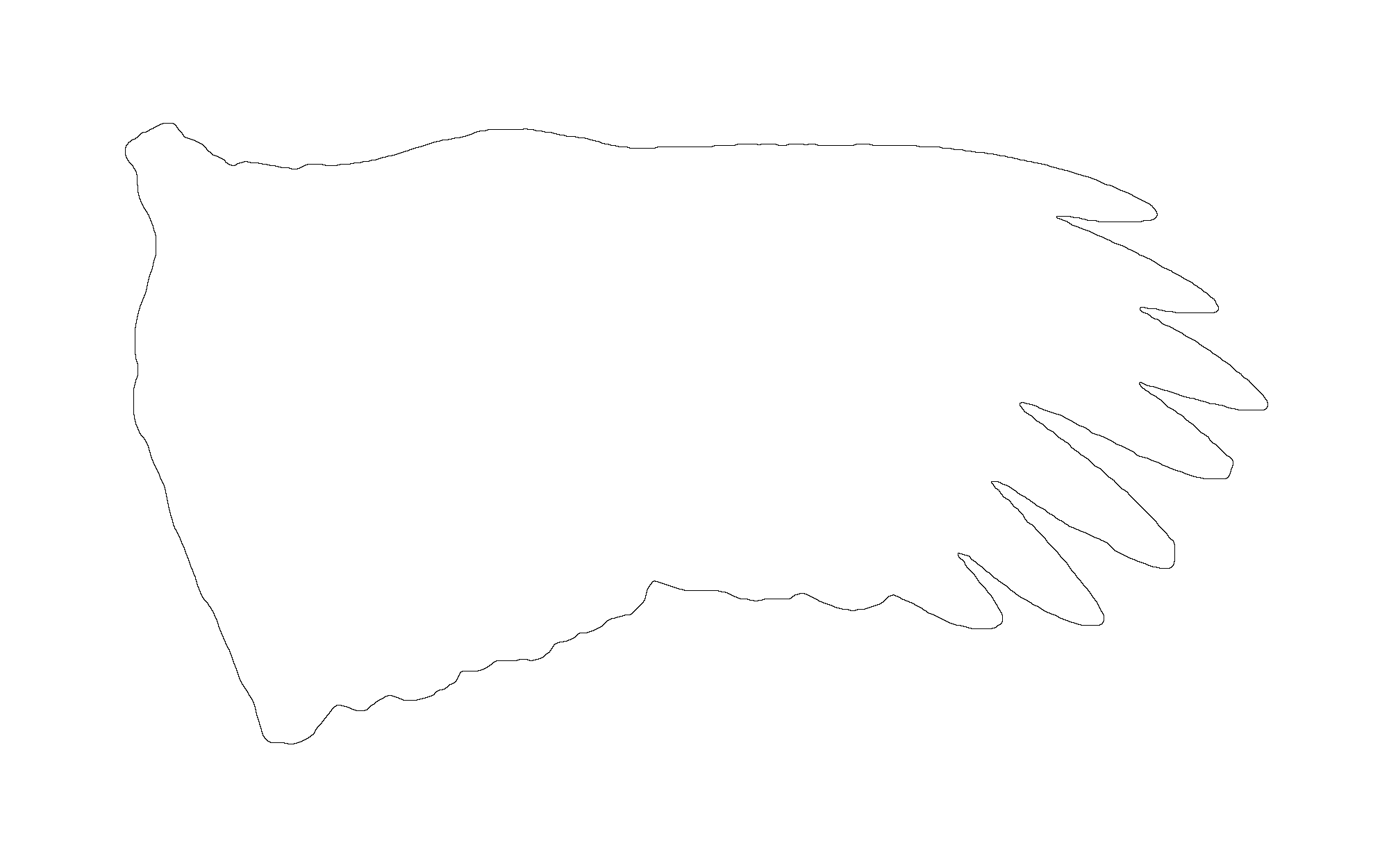

Supplement: Supplementary file 6 — Supplementary Data 4 [file 41467_2026_70692_MOESM6_ESM.zip › Supplementary Data 4/Ardeotis_kori.tif]

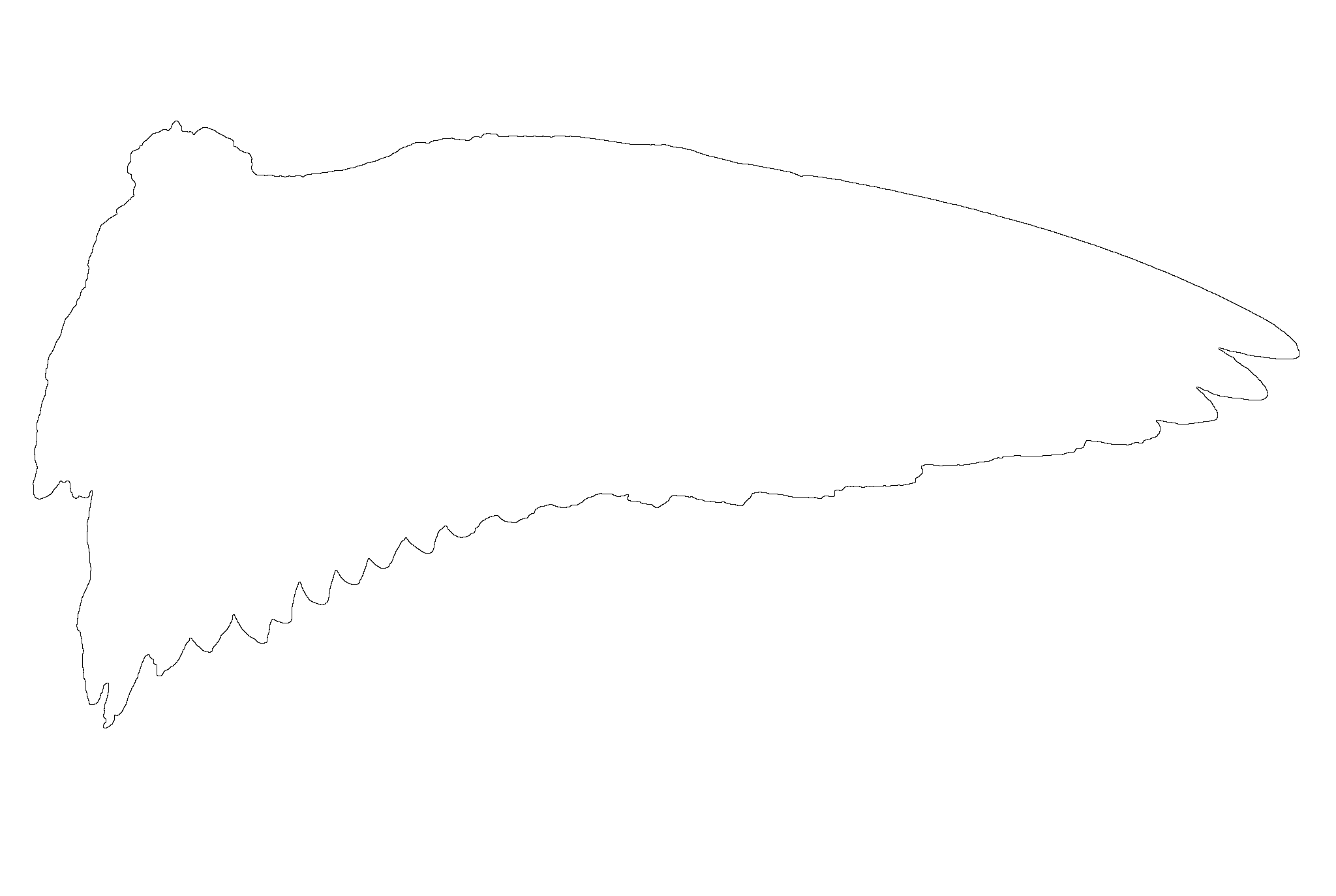

Supplement: Supplementary file 6 — Supplementary Data 4 [file 41467_2026_70692_MOESM6_ESM.zip › Supplementary Data 4/Arenaria_interpres.tif]

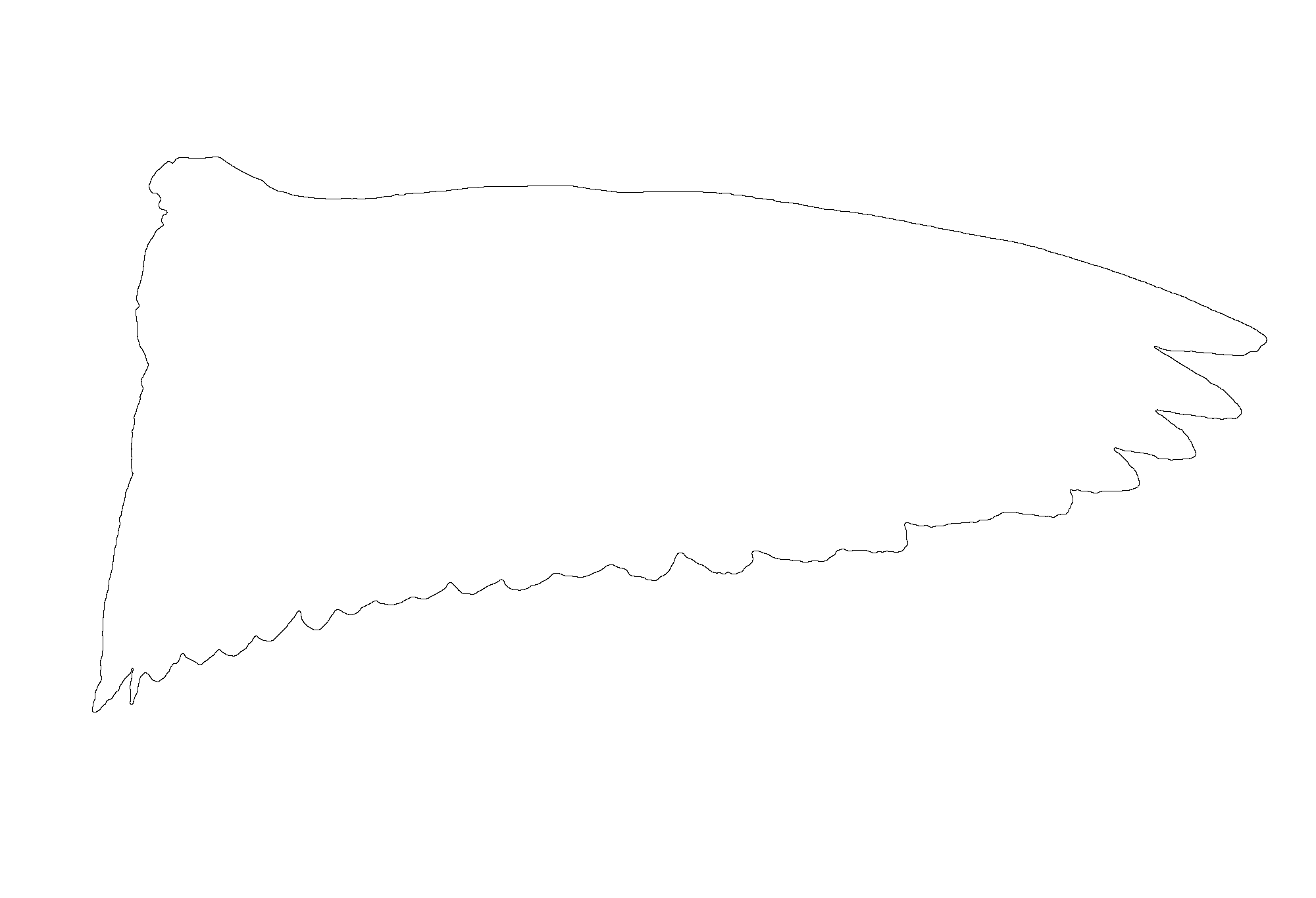

Supplement: Supplementary file 6 — Supplementary Data 4 [file 41467_2026_70692_MOESM6_ESM.zip › Supplementary Data 4/Arenaria_melanocephala.tif]

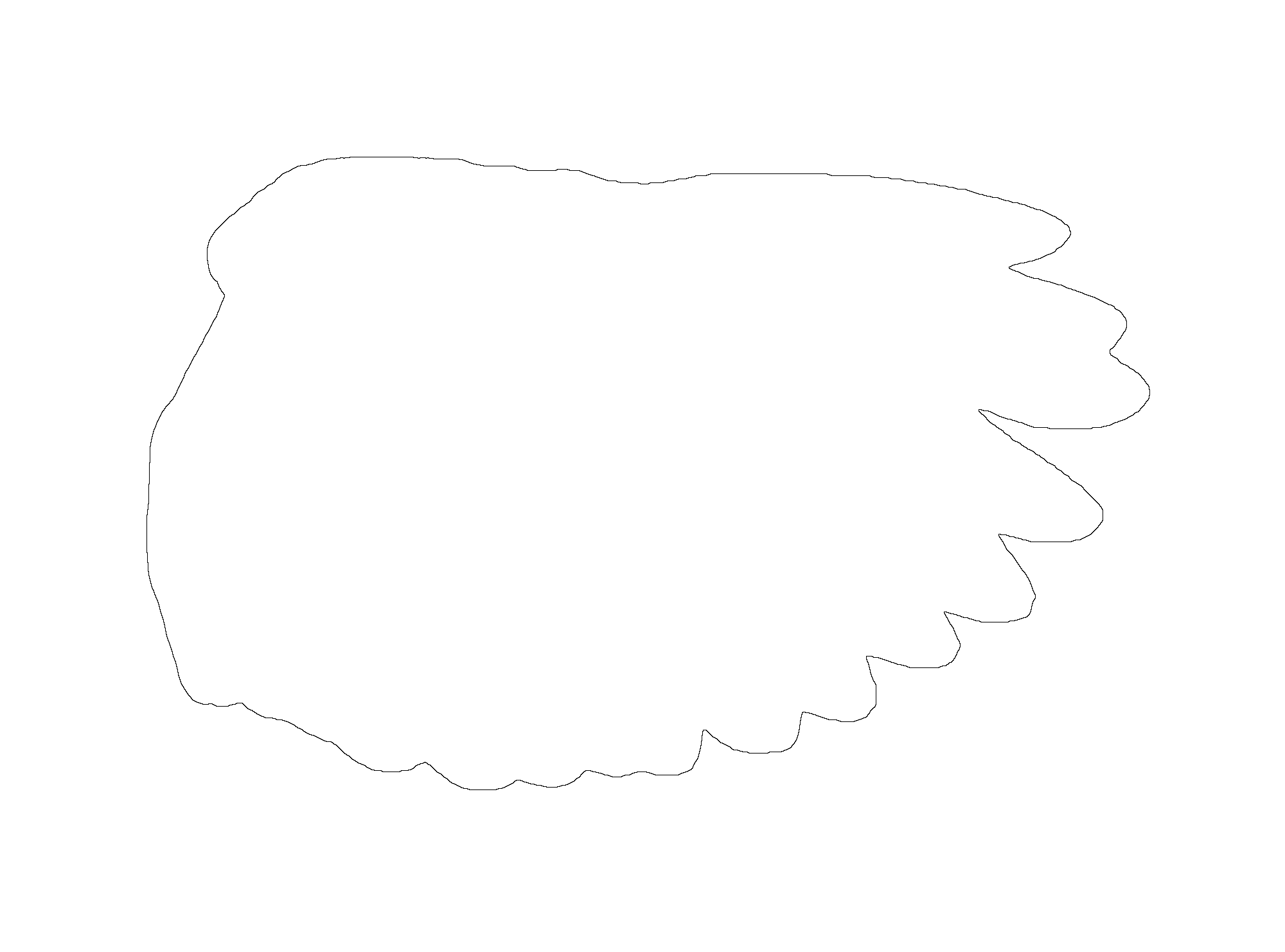

Supplement: Supplementary file 6 — Supplementary Data 4 [file 41467_2026_70692_MOESM6_ESM.zip › Supplementary Data 4/Arremonops_conirostris.tif]

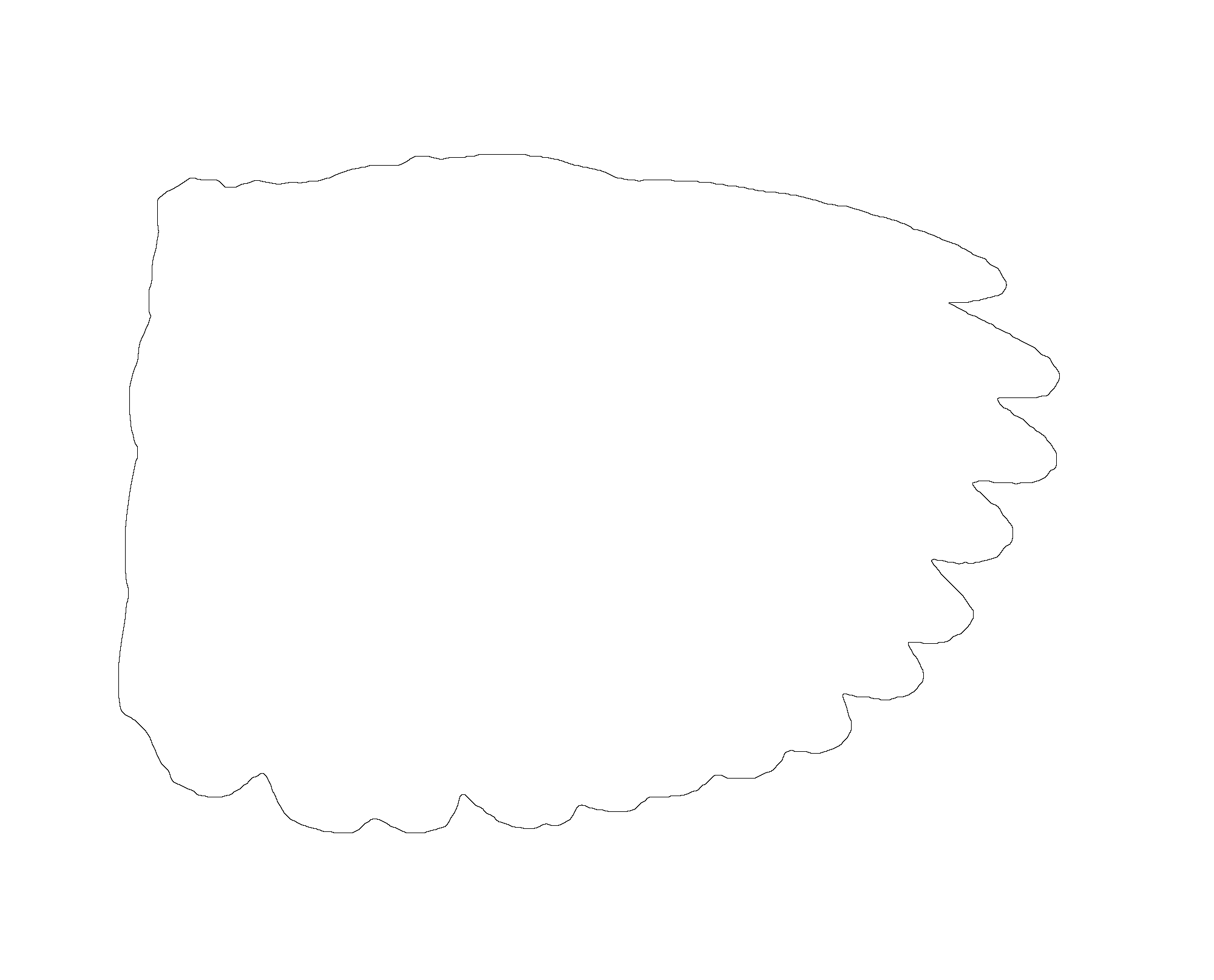

Supplement: Supplementary file 6 — Supplementary Data 4 [file 41467_2026_70692_MOESM6_ESM.zip › Supplementary Data 4/Arremonops_rufivirgatus.tif]

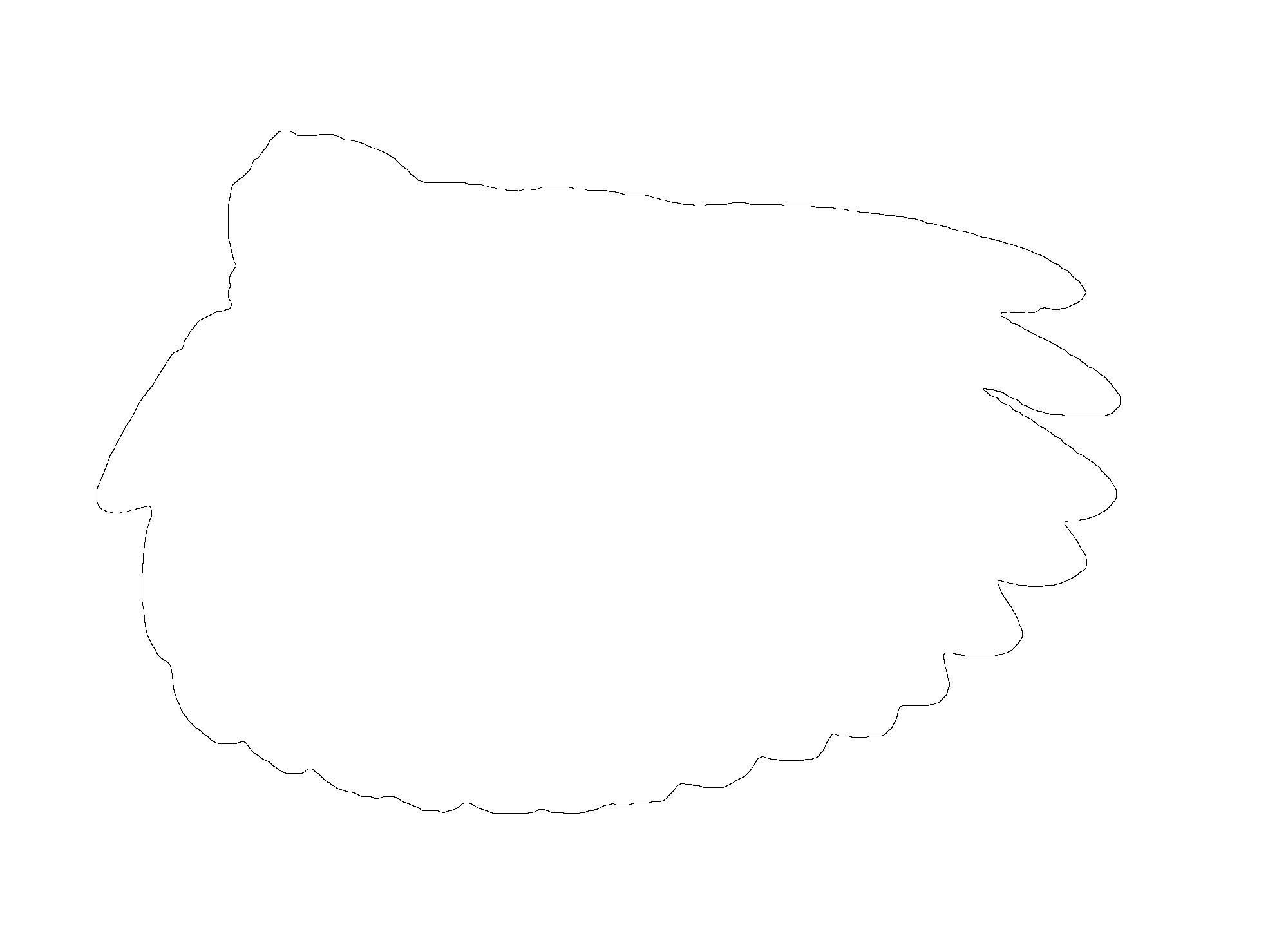

Supplement: Supplementary file 6 — Supplementary Data 4 [file 41467_2026_70692_MOESM6_ESM.zip › Supplementary Data 4/Arremon_aurantiirostris.tif]

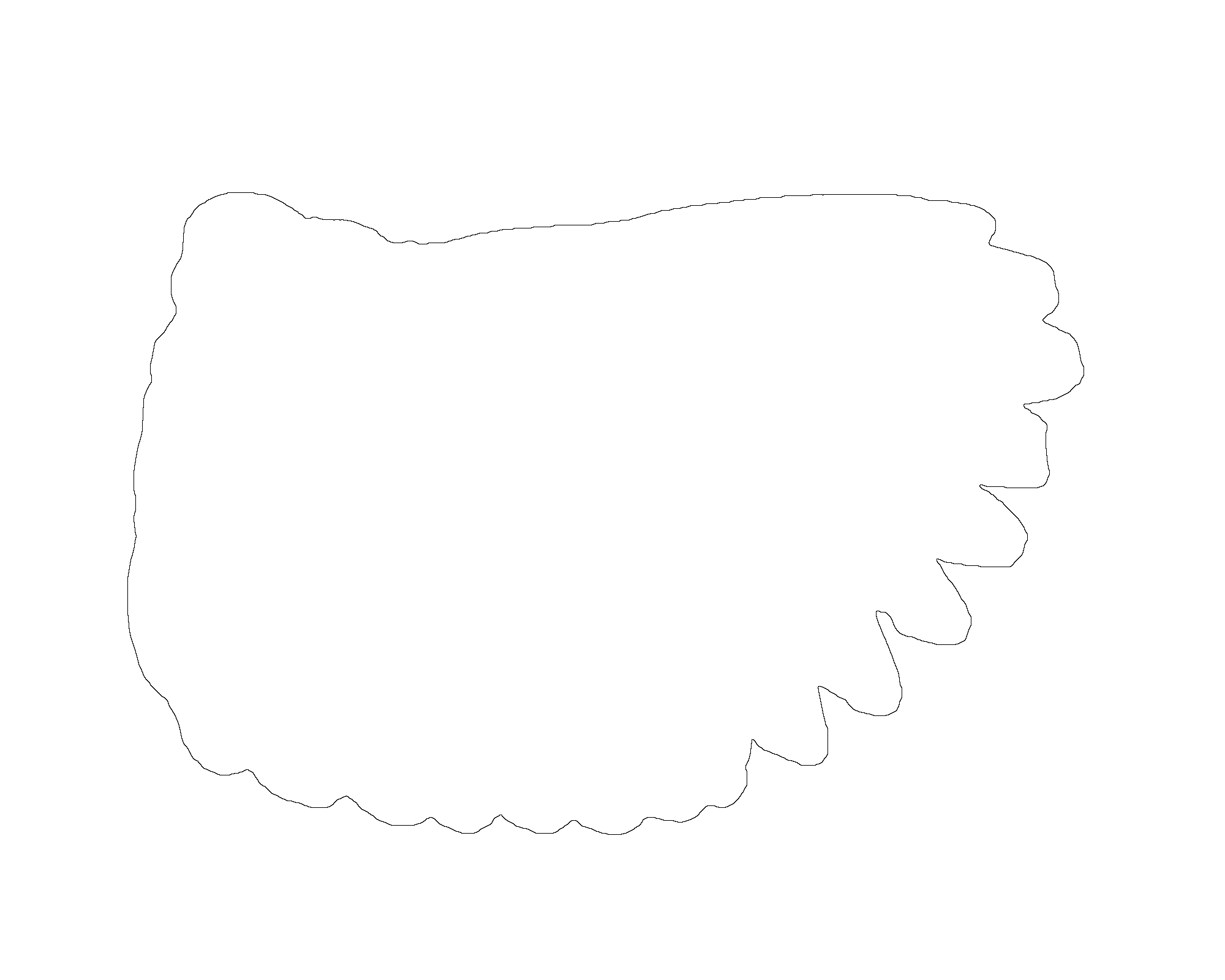

Supplement: Supplementary file 6 — Supplementary Data 4 [file 41467_2026_70692_MOESM6_ESM.zip › Supplementary Data 4/Arremon_brunneinucha.tif]

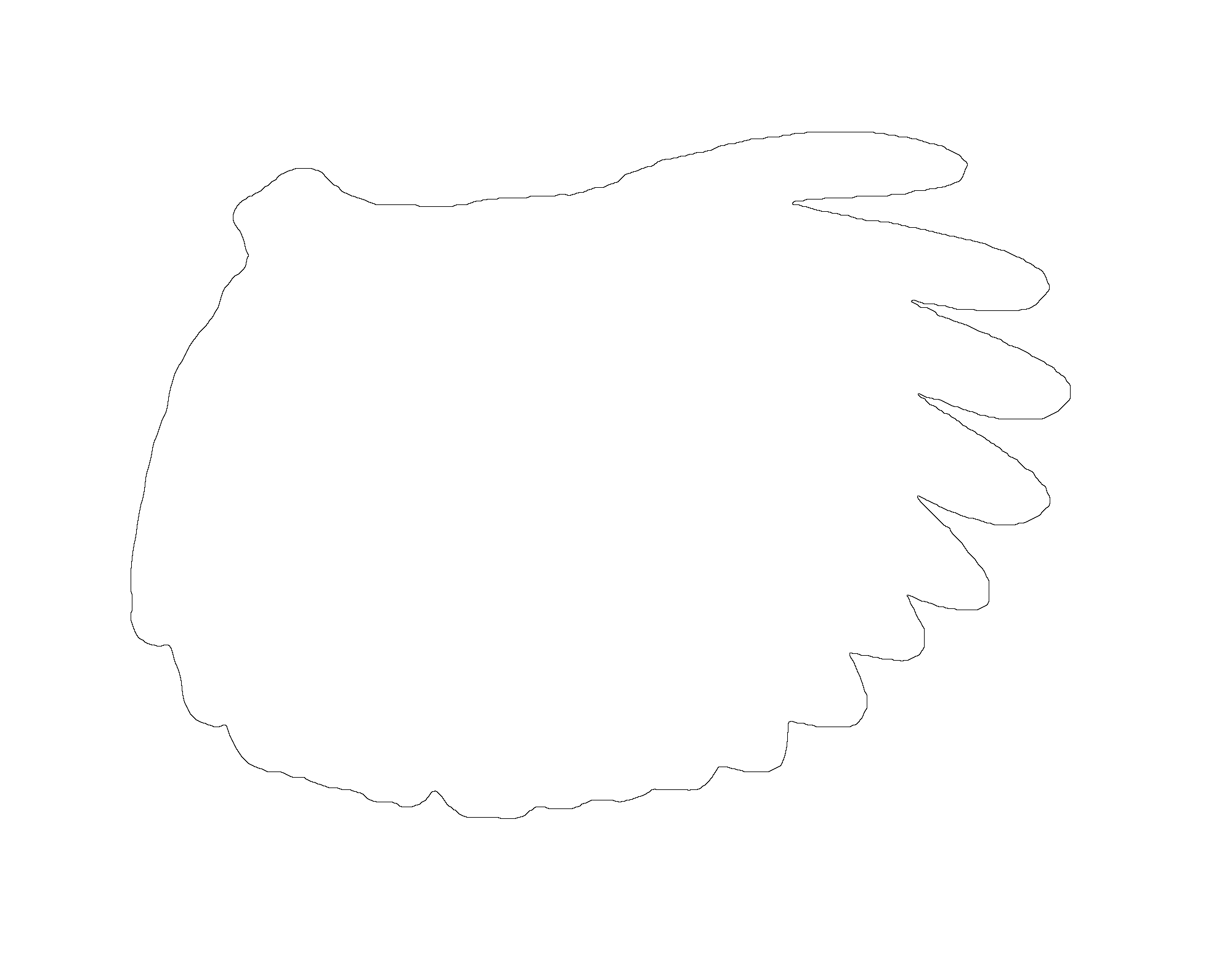

Supplement: Supplementary file 6 — Supplementary Data 4 [file 41467_2026_70692_MOESM6_ESM.zip › Supplementary Data 4/Arremon_virenticeps.tif]

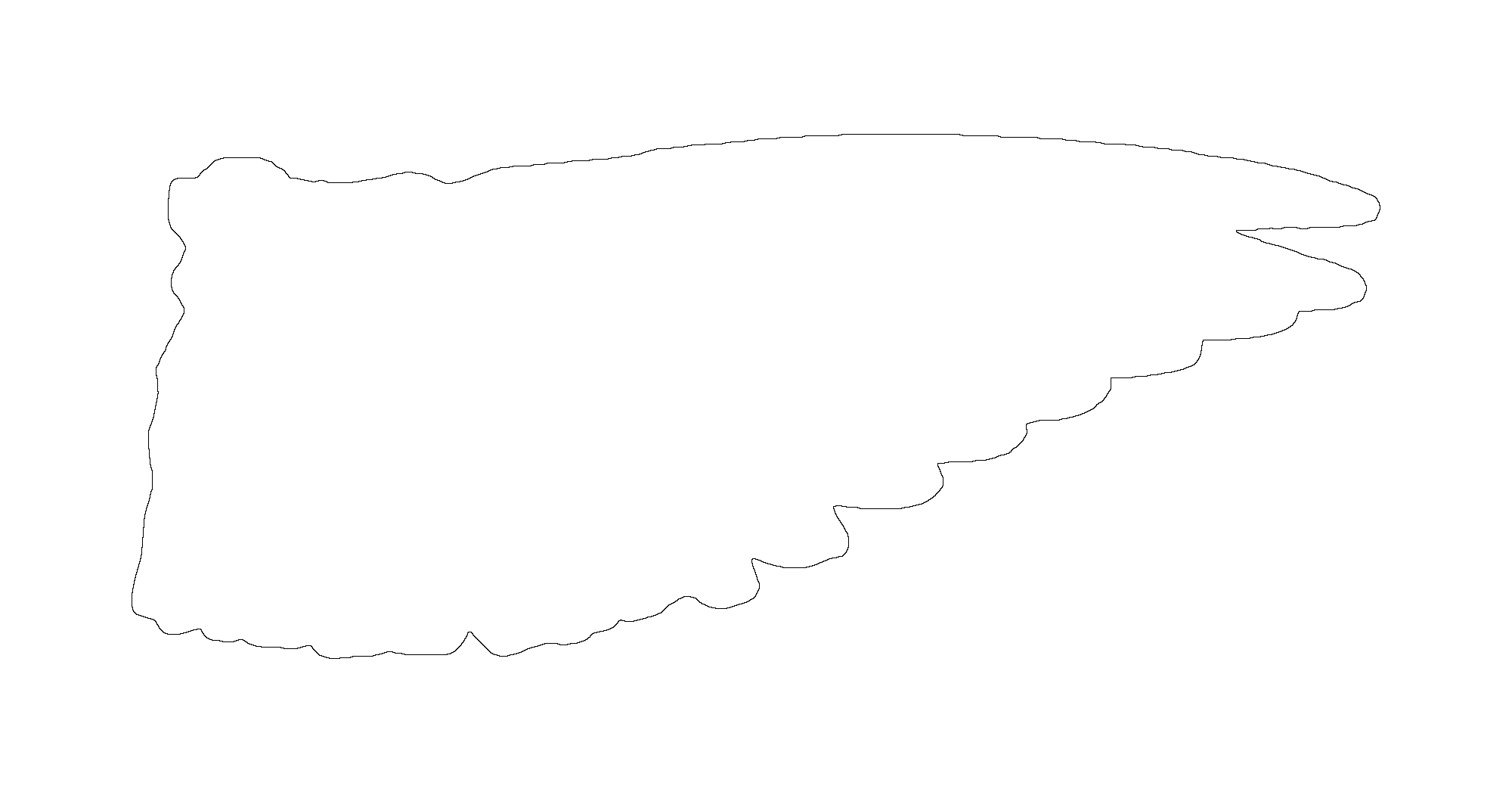

Supplement: Supplementary file 6 — Supplementary Data 4 [file 41467_2026_70692_MOESM6_ESM.zip › Supplementary Data 4/Artamus_leucorynchus.tif]

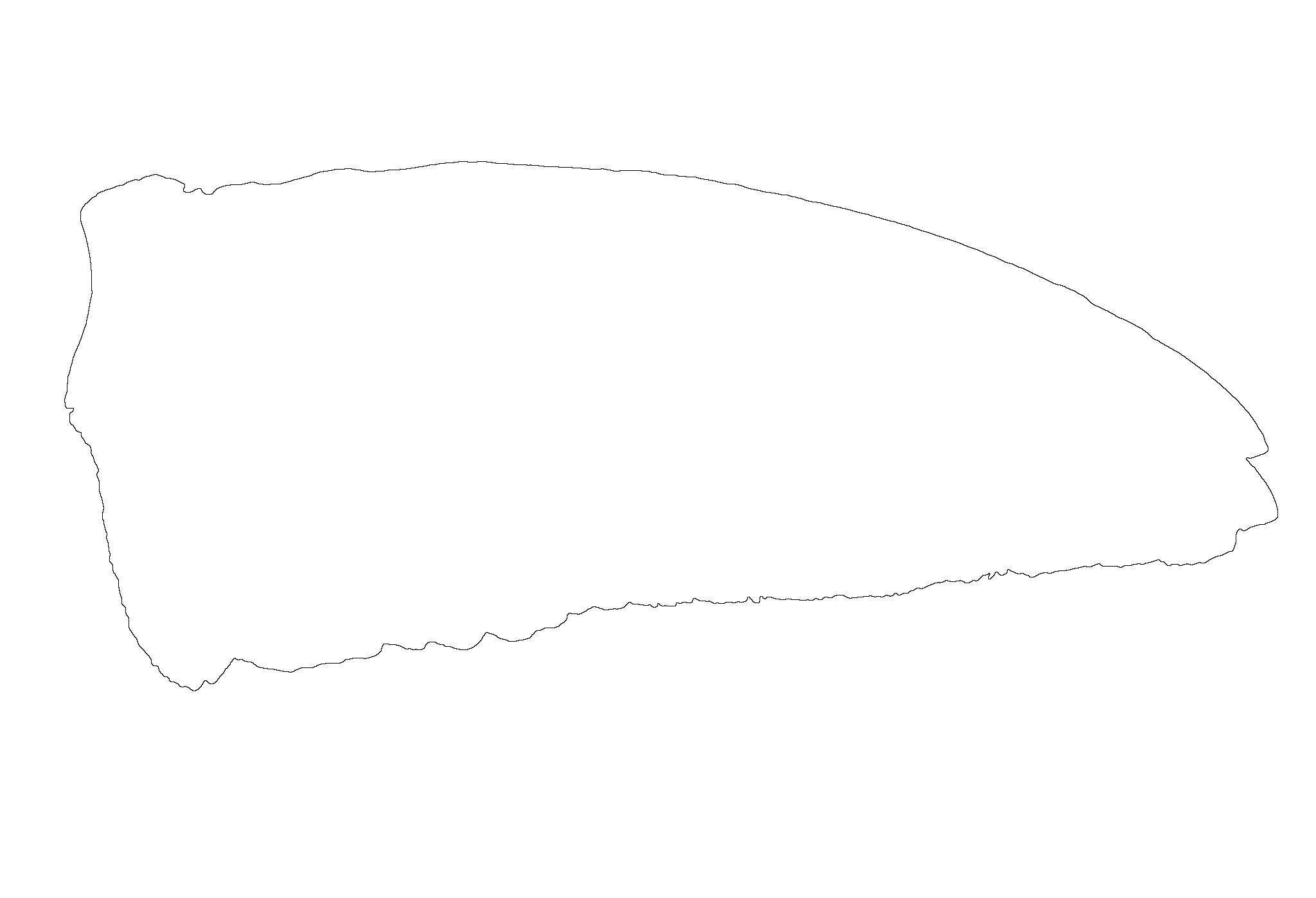

Supplement: Supplementary file 6 — Supplementary Data 4 [file 41467_2026_70692_MOESM6_ESM.zip › Supplementary Data 4/Asio_flammeus.tif]

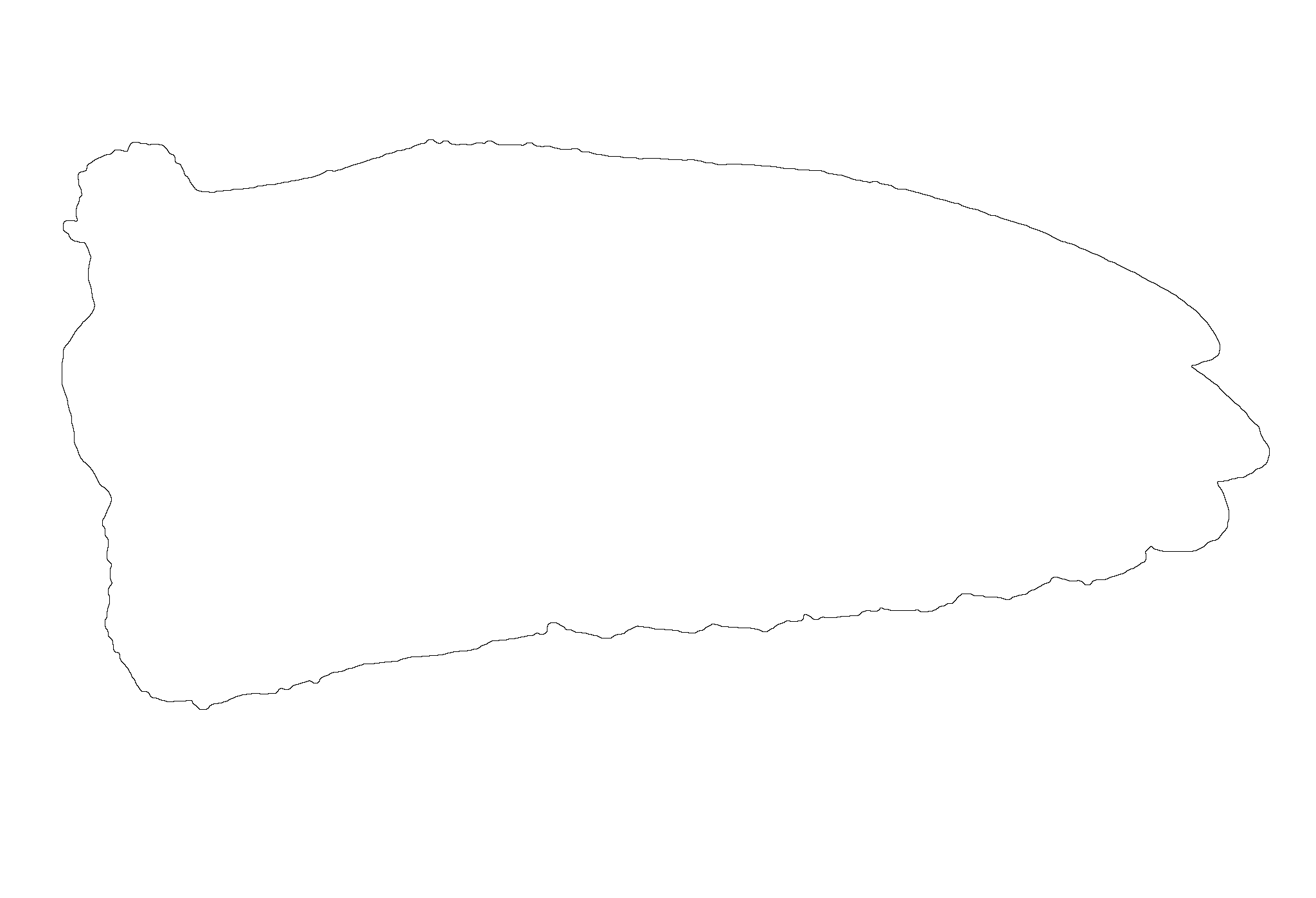

Supplement: Supplementary file 6 — Supplementary Data 4 [file 41467_2026_70692_MOESM6_ESM.zip › Supplementary Data 4/Asio_otus.tif]

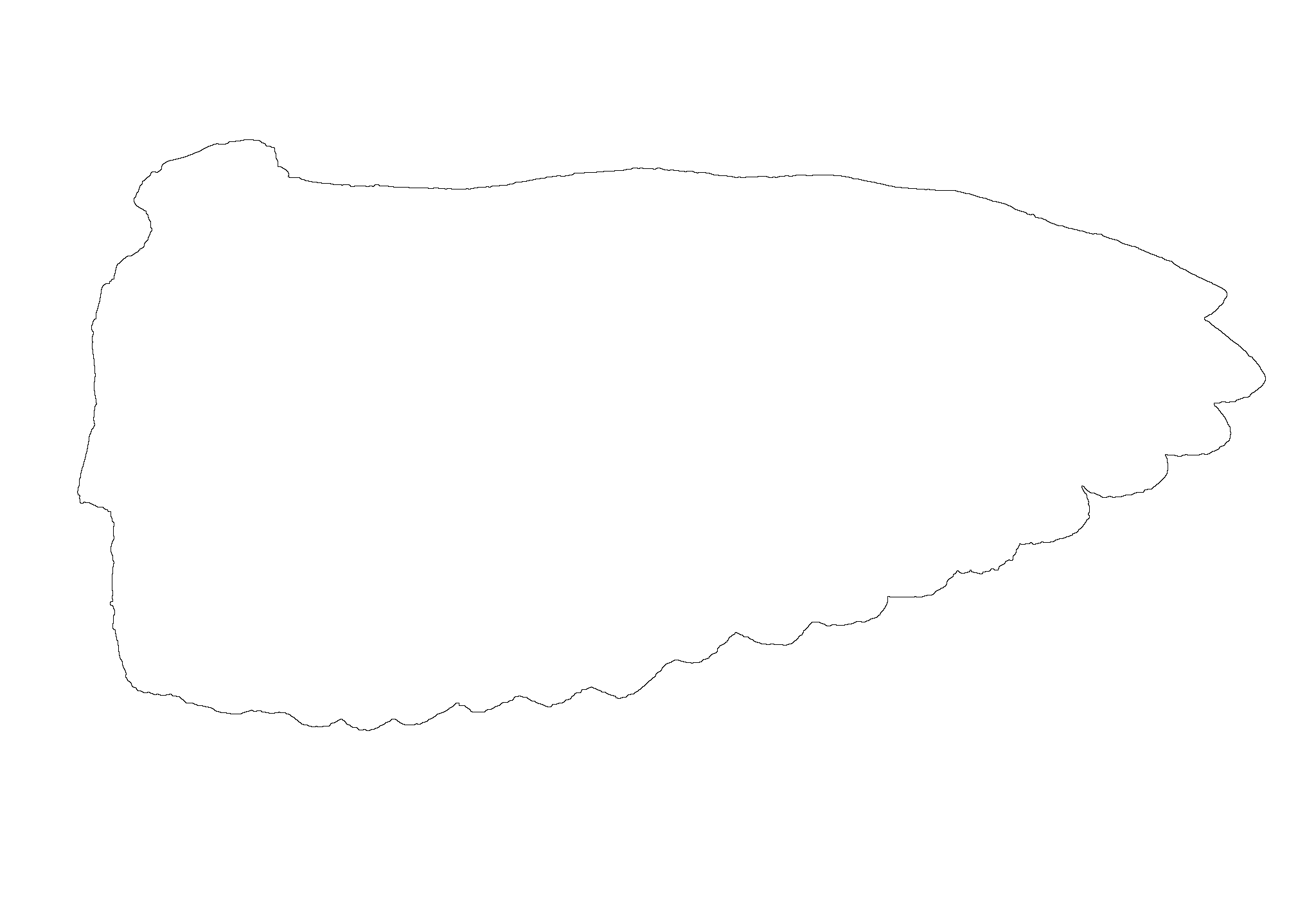

Supplement: Supplementary file 6 — Supplementary Data 4 [file 41467_2026_70692_MOESM6_ESM.zip › Supplementary Data 4/Athene_cunicularia.tif]

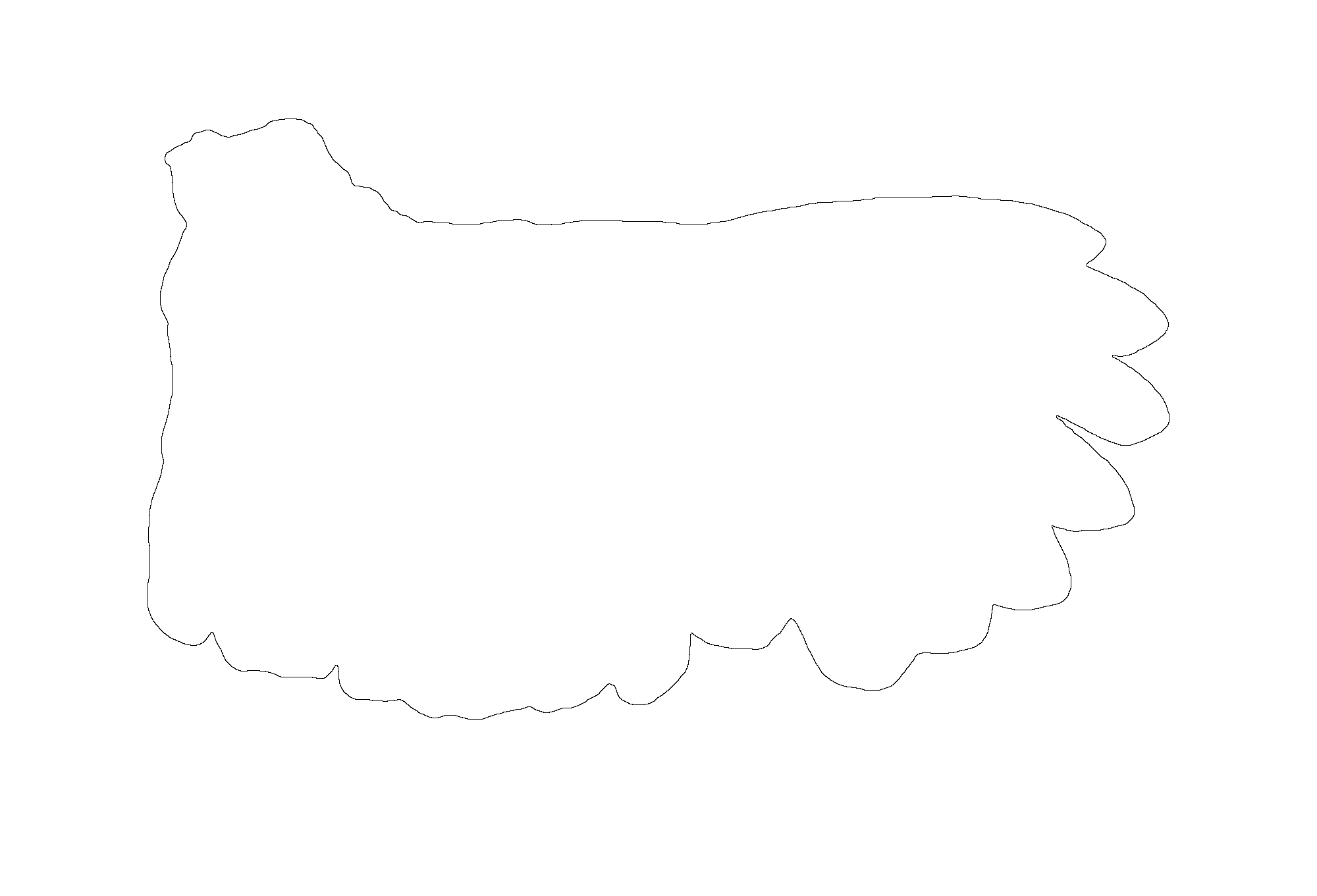

Supplement: Supplementary file 6 — Supplementary Data 4 [file 41467_2026_70692_MOESM6_ESM.zip › Supplementary Data 4/Athene_noctua.tif]

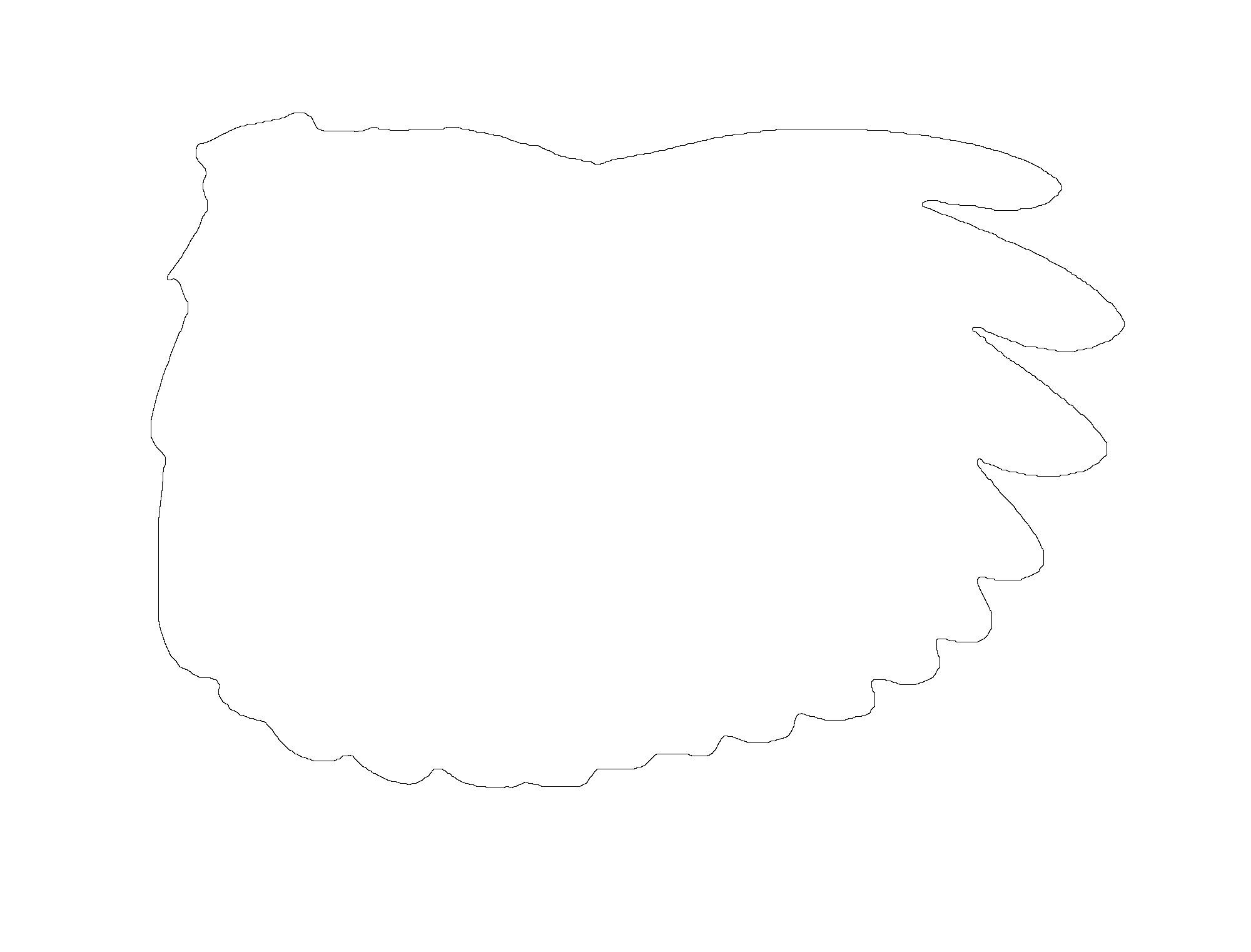

Supplement: Supplementary file 6 — Supplementary Data 4 [file 41467_2026_70692_MOESM6_ESM.zip › Supplementary Data 4/Atlapetes_albinucha.tif]

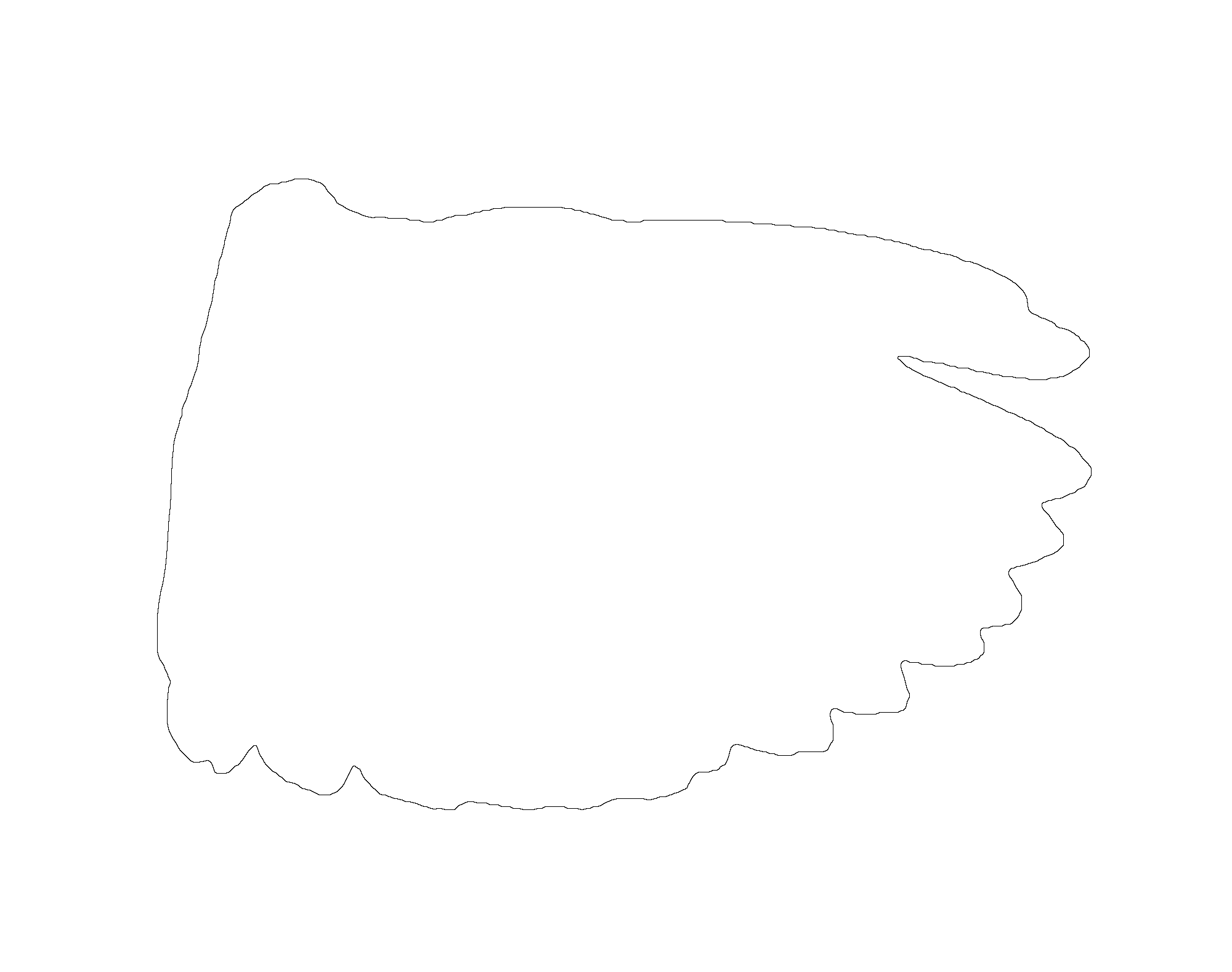

Supplement: Supplementary file 6 — Supplementary Data 4 [file 41467_2026_70692_MOESM6_ESM.zip › Supplementary Data 4/Atlapetes_citrinellus.tif]

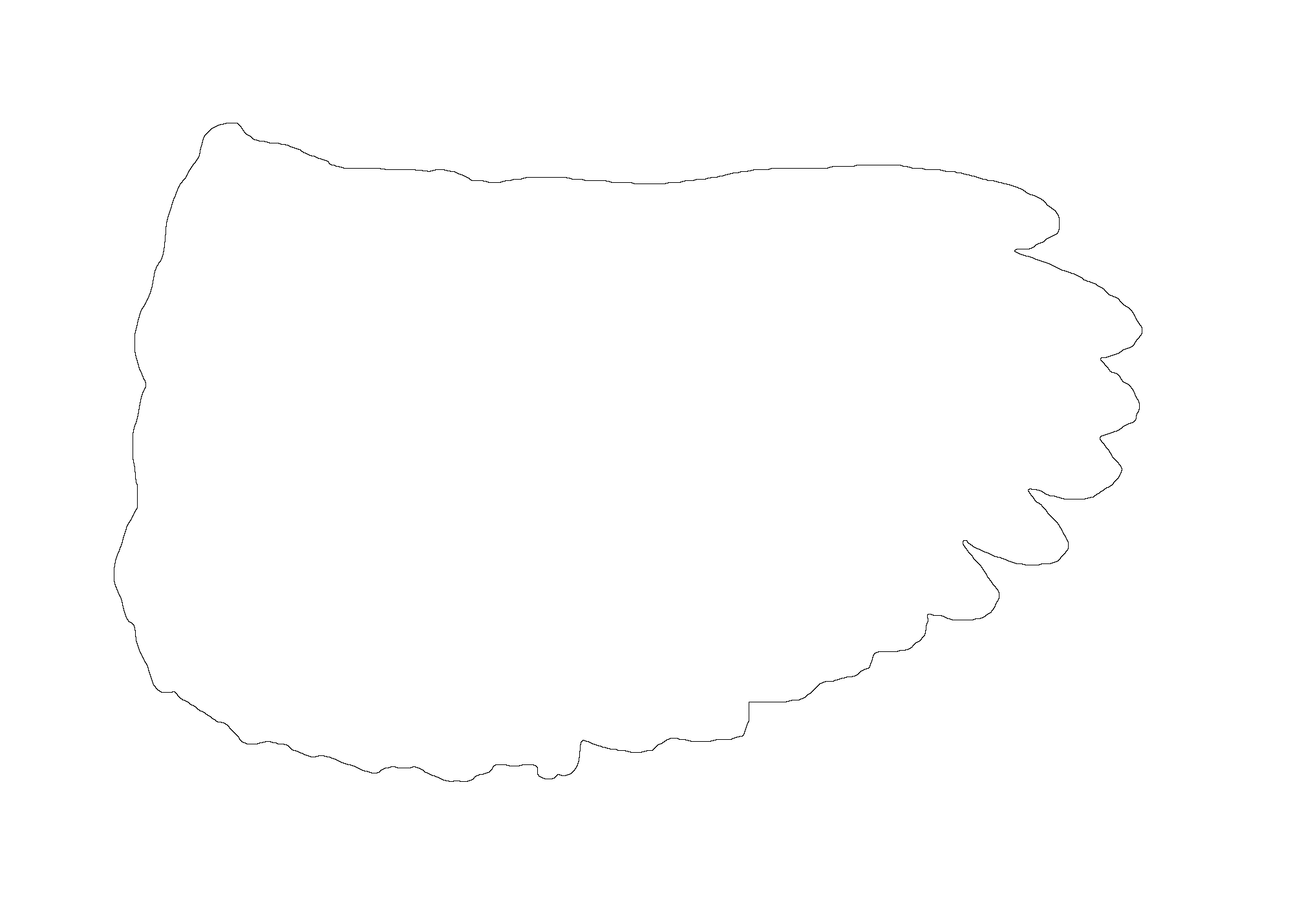

Supplement: Supplementary file 6 — Supplementary Data 4 [file 41467_2026_70692_MOESM6_ESM.zip › Supplementary Data 4/Attila_spadiceus.tif]

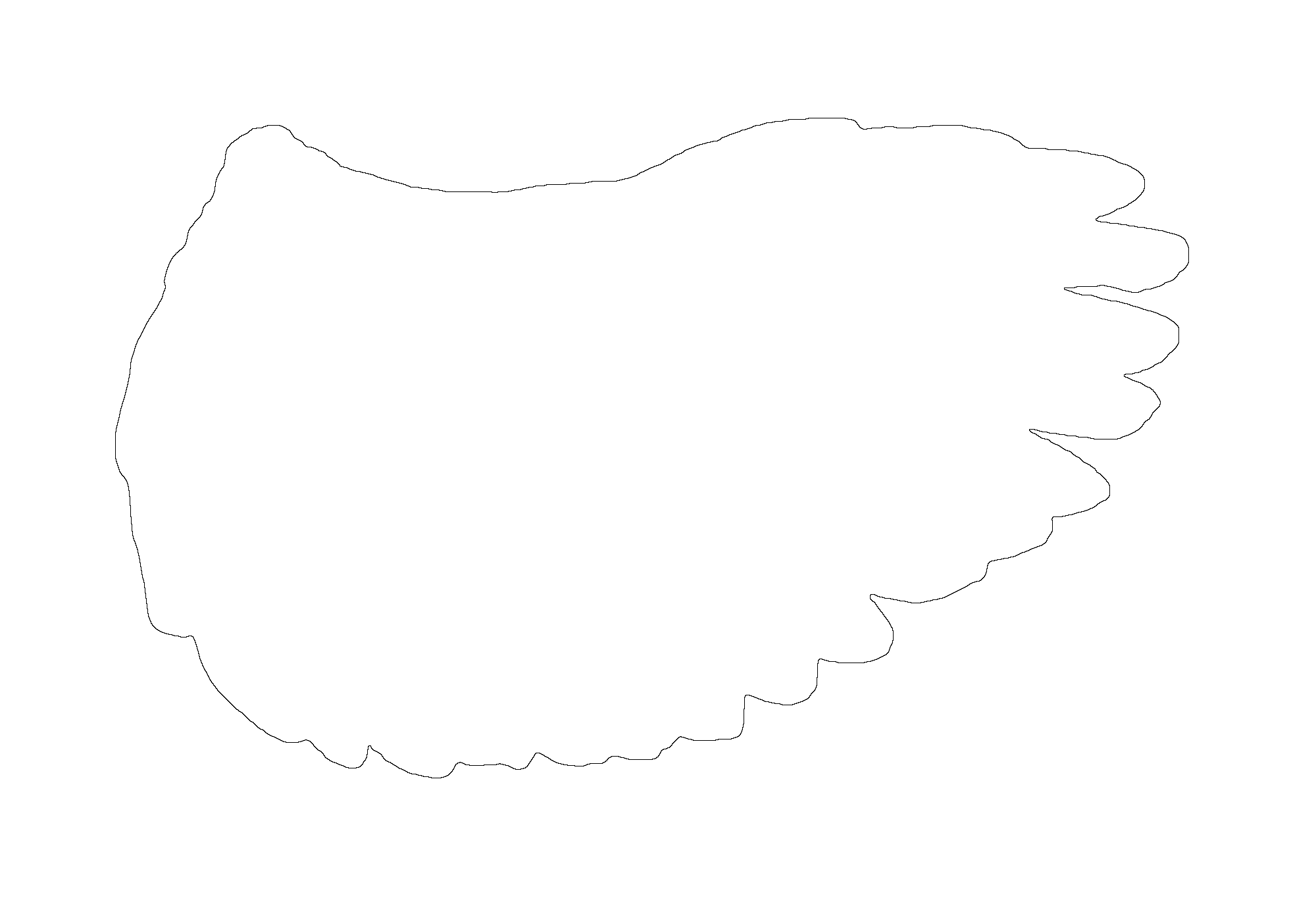

Supplement: Supplementary file 6 — Supplementary Data 4 [file 41467_2026_70692_MOESM6_ESM.zip › Supplementary Data 4/Aulacorhynchus_prasinus.tif]

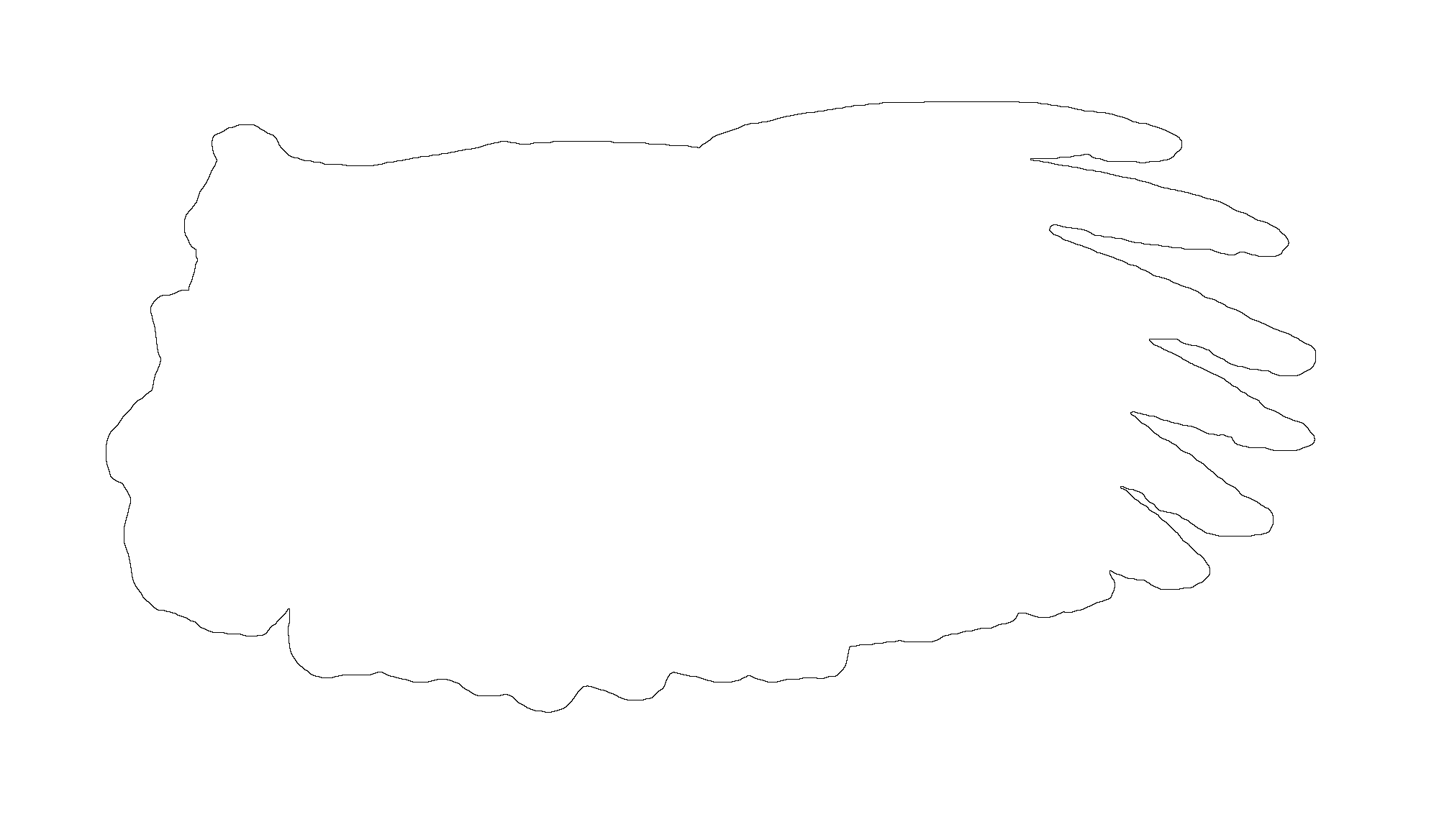

Supplement: Supplementary file 6 — Supplementary Data 4 [file 41467_2026_70692_MOESM6_ESM.zip › Supplementary Data 4/Aviceda_jerdoni.tif]

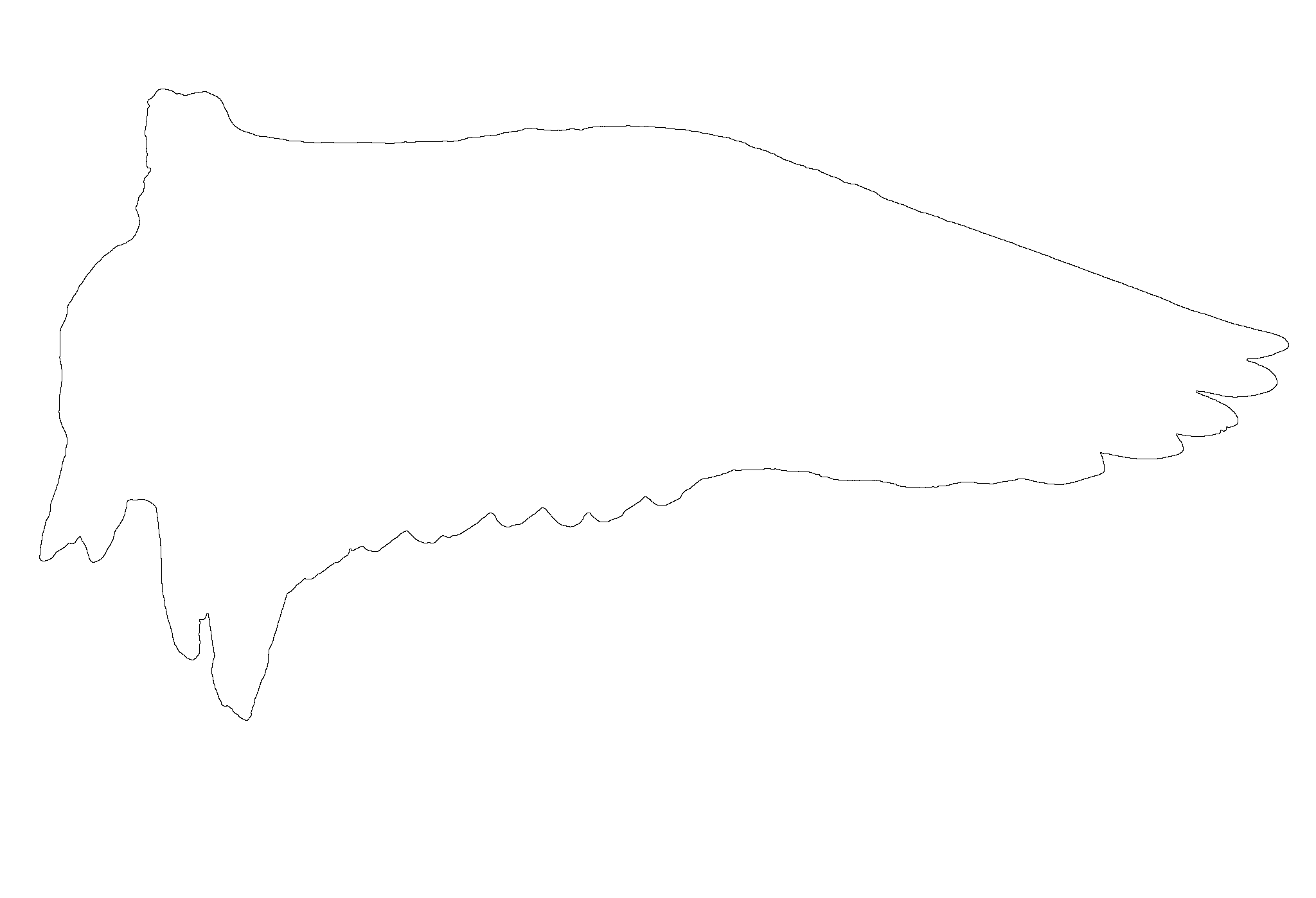

Supplement: Supplementary file 6 — Supplementary Data 4 [file 41467_2026_70692_MOESM6_ESM.zip › Supplementary Data 4/Aythya_affinis.tif]

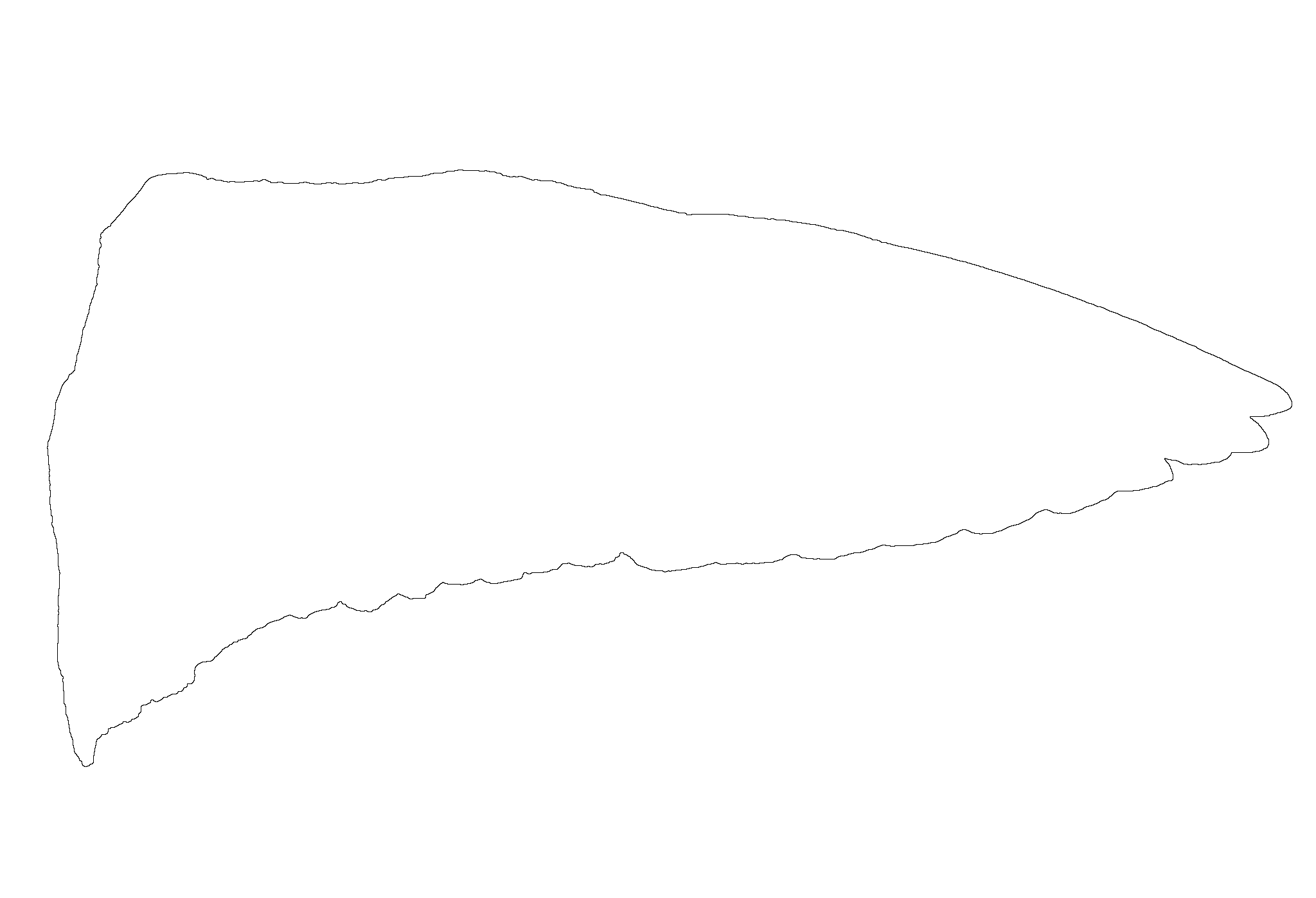

Supplement: Supplementary file 6 — Supplementary Data 4 [file 41467_2026_70692_MOESM6_ESM.zip › Supplementary Data 4/Aythya_americana.tif]

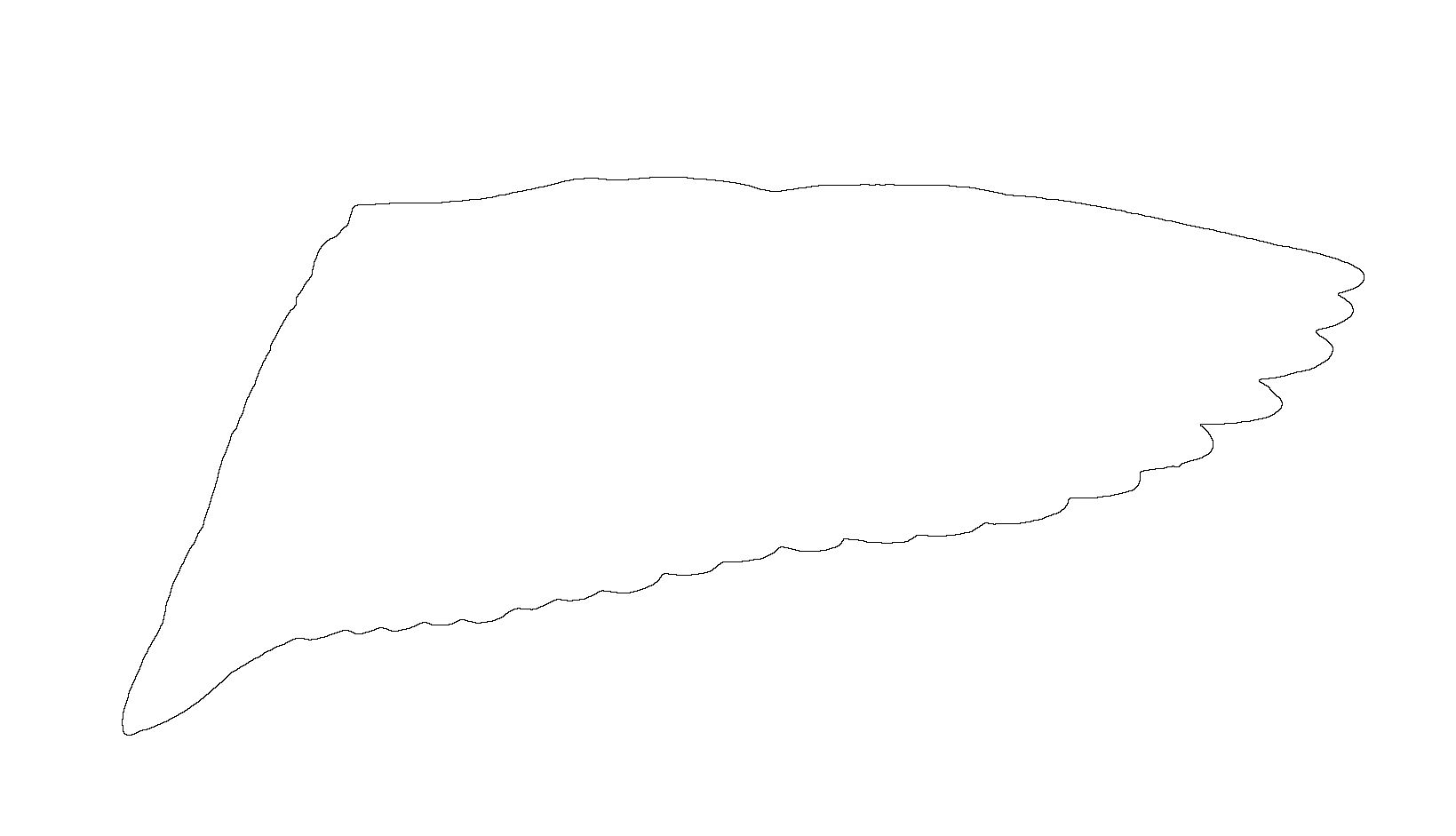

Supplement: Supplementary file 6 — Supplementary Data 4 [file 41467_2026_70692_MOESM6_ESM.zip › Supplementary Data 4/Aythya_australis.tif]

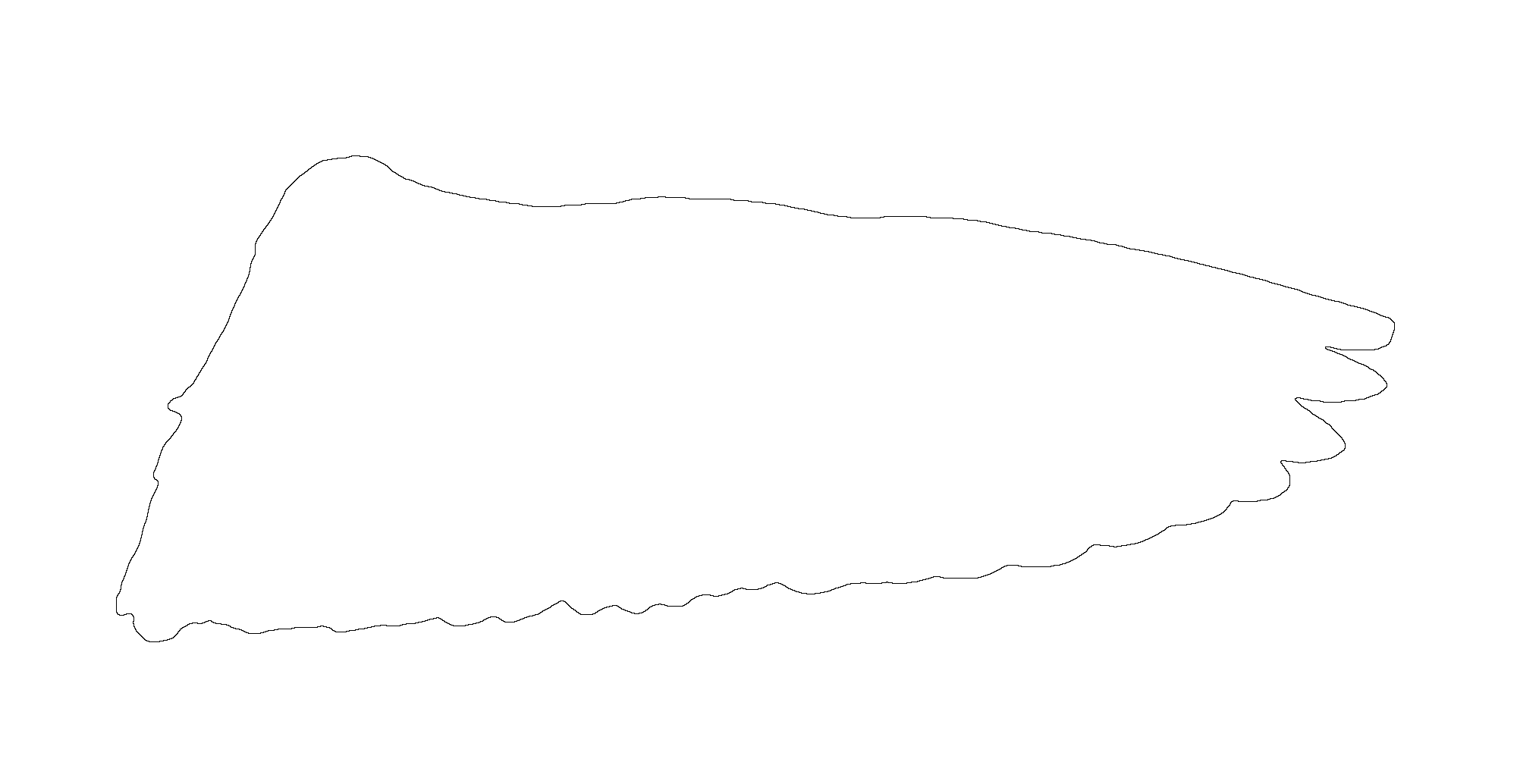

Supplement: Supplementary file 6 — Supplementary Data 4 [file 41467_2026_70692_MOESM6_ESM.zip › Supplementary Data 4/Aythya_collaris.tif]

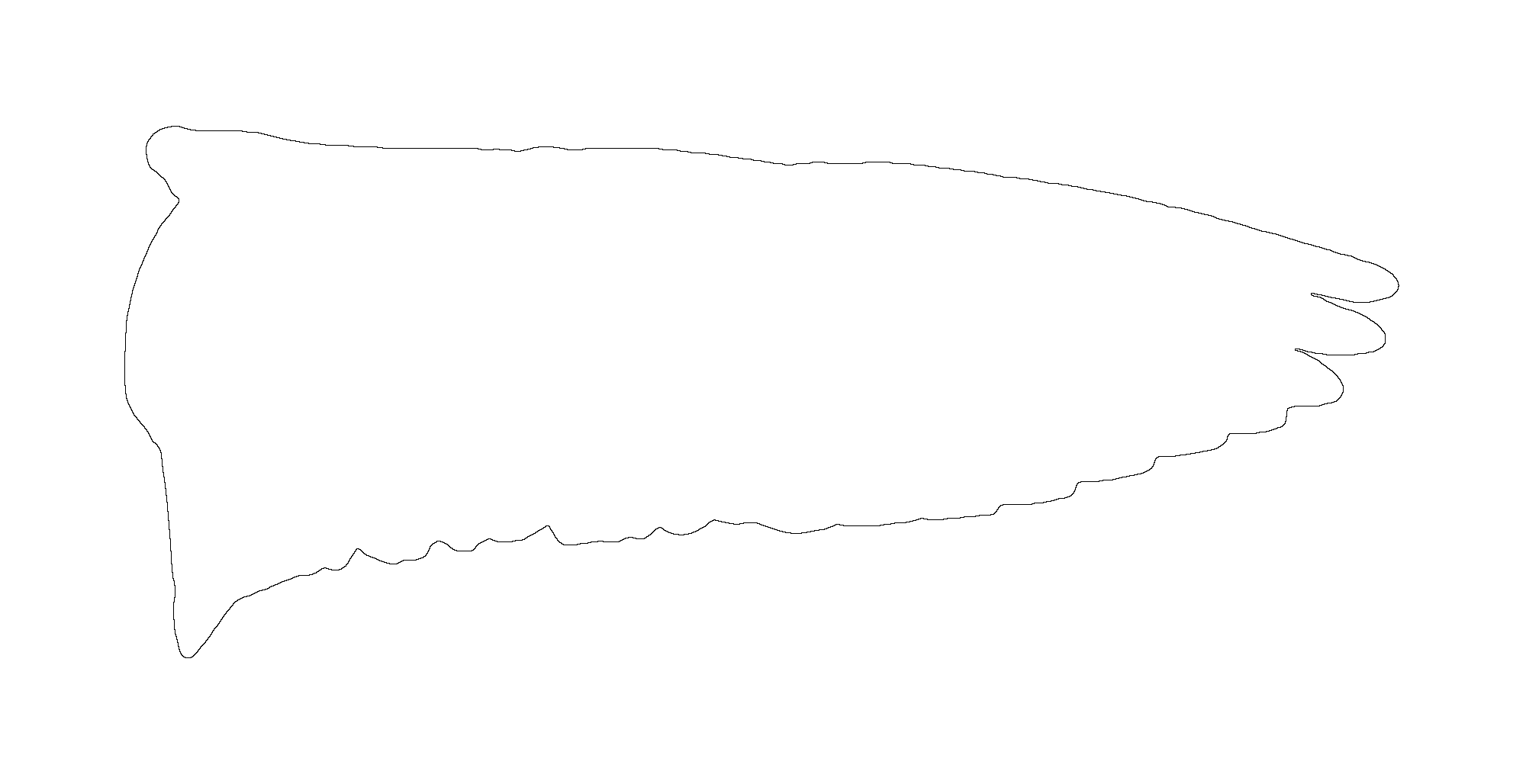

Supplement: Supplementary file 6 — Supplementary Data 4 [file 41467_2026_70692_MOESM6_ESM.zip › Supplementary Data 4/Aythya_fuligula.tif]

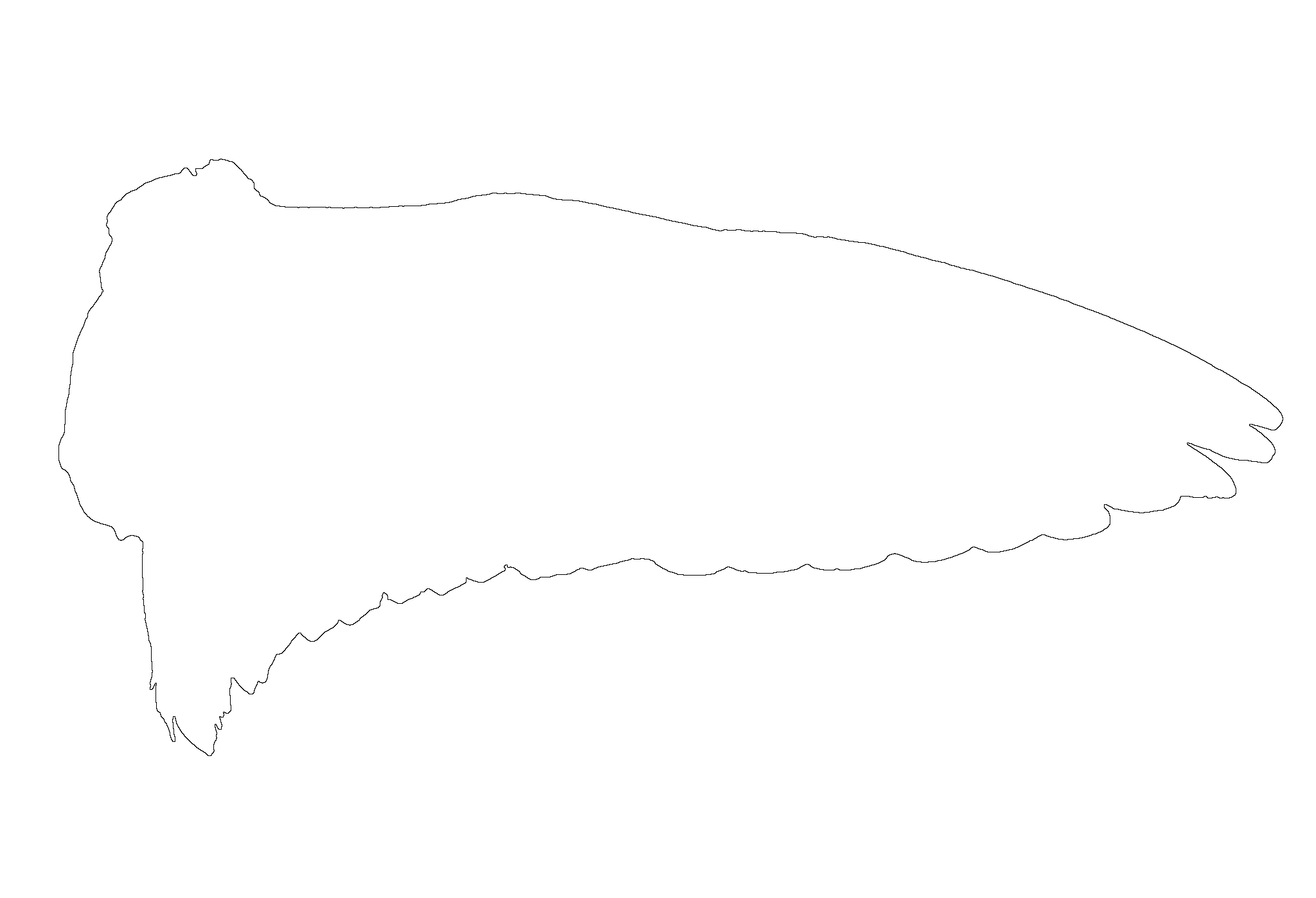

Supplement: Supplementary file 6 — Supplementary Data 4 [file 41467_2026_70692_MOESM6_ESM.zip › Supplementary Data 4/Aythya_marila.tif]

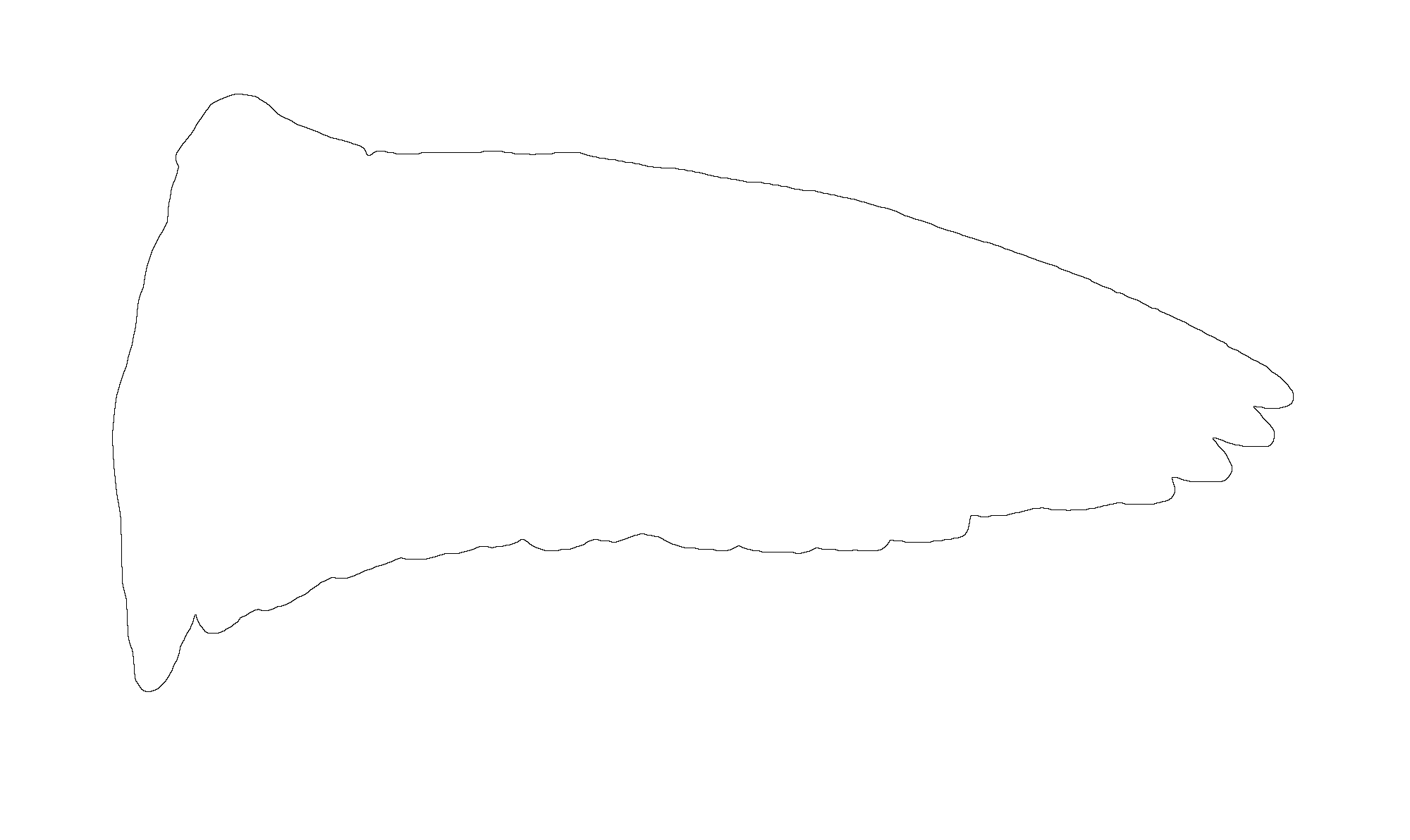

Supplement: Supplementary file 6 — Supplementary Data 4 [file 41467_2026_70692_MOESM6_ESM.zip › Supplementary Data 4/Aythya_nyroca.tif]

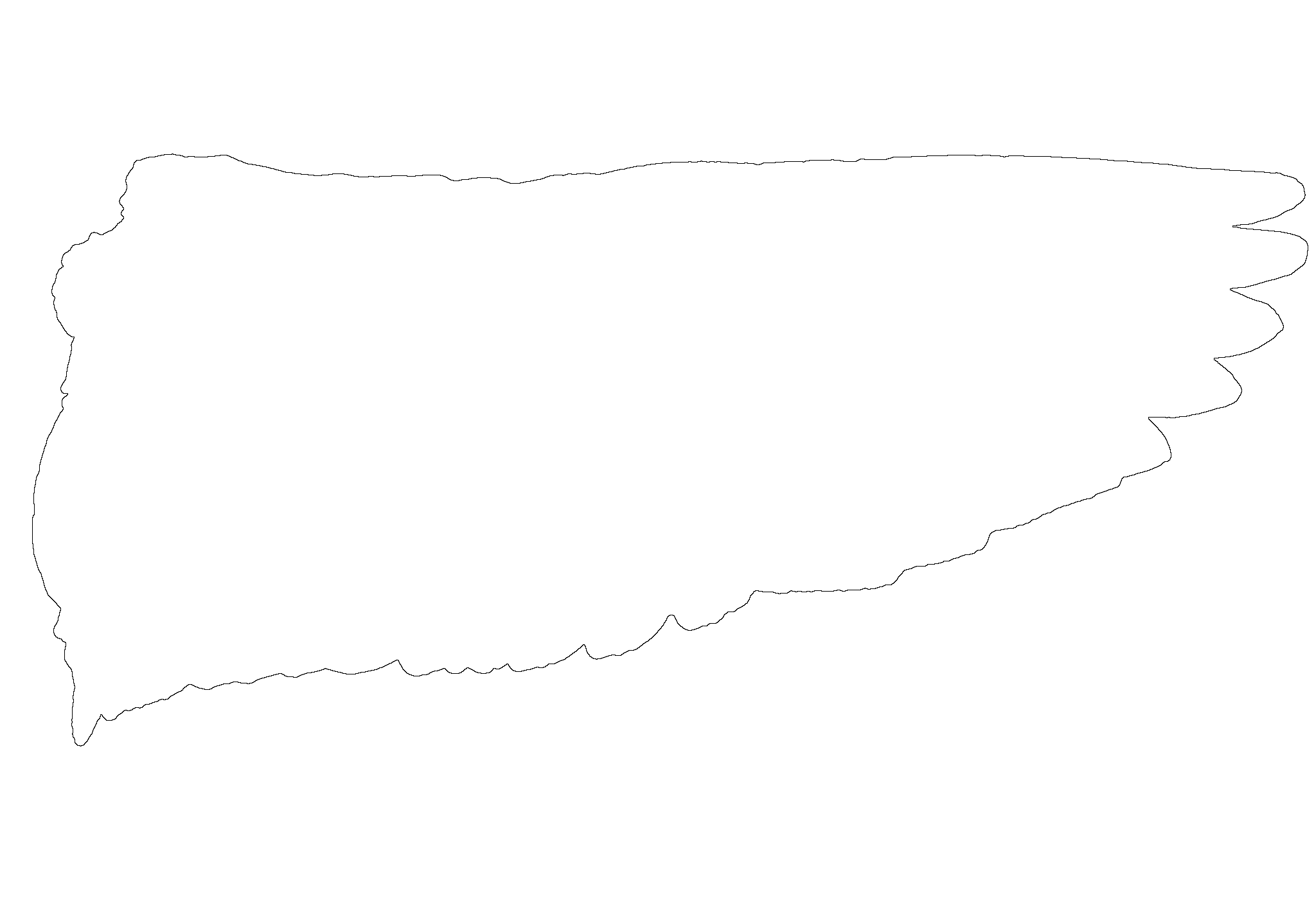

Supplement: Supplementary file 6 — Supplementary Data 4 [file 41467_2026_70692_MOESM6_ESM.zip › Supplementary Data 4/Aythya_valisineria.tif]

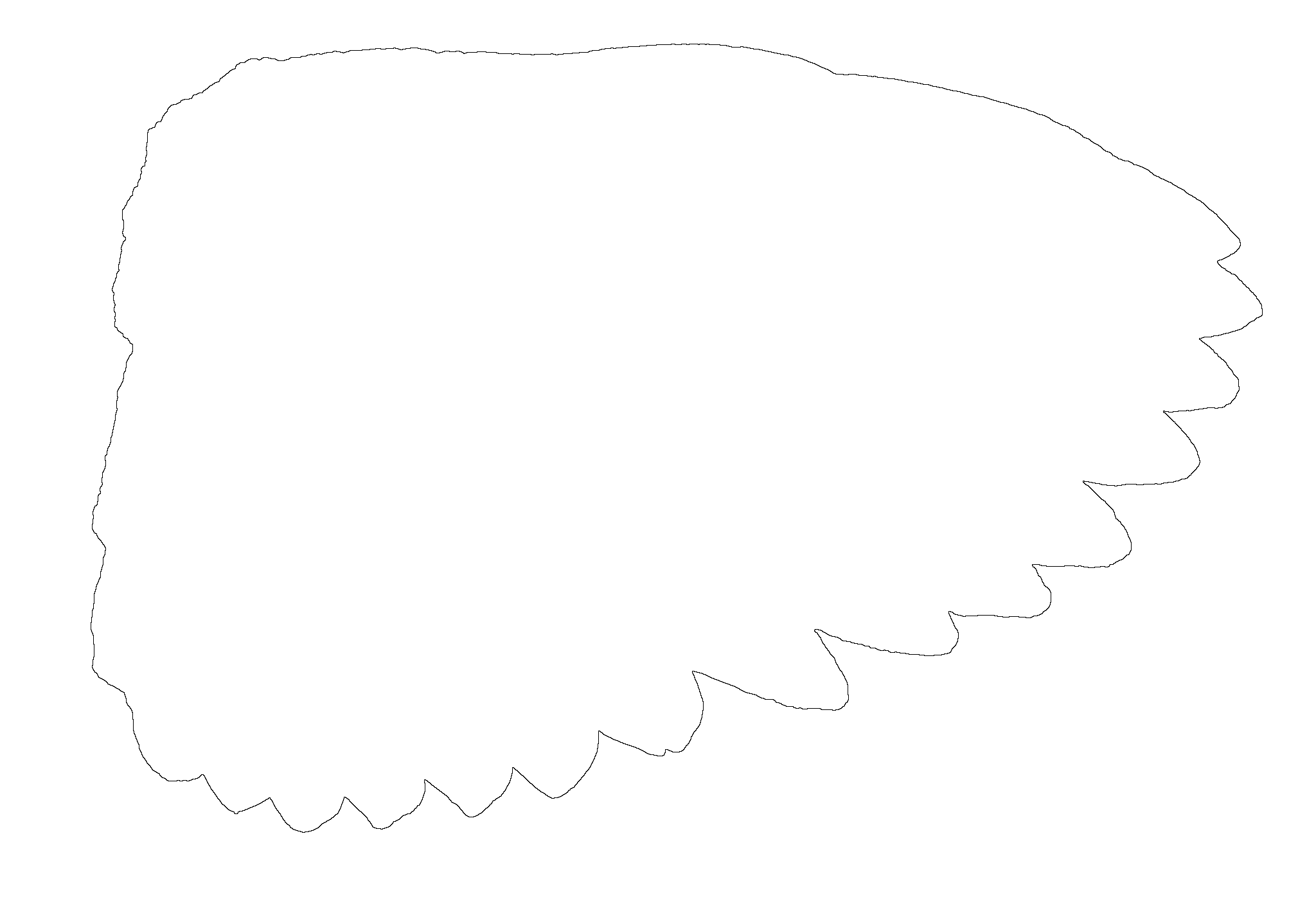

Supplement: Supplementary file 6 — Supplementary Data 4 [file 41467_2026_70692_MOESM6_ESM.zip › Supplementary Data 4/Baeolophus_bicolor.tif]

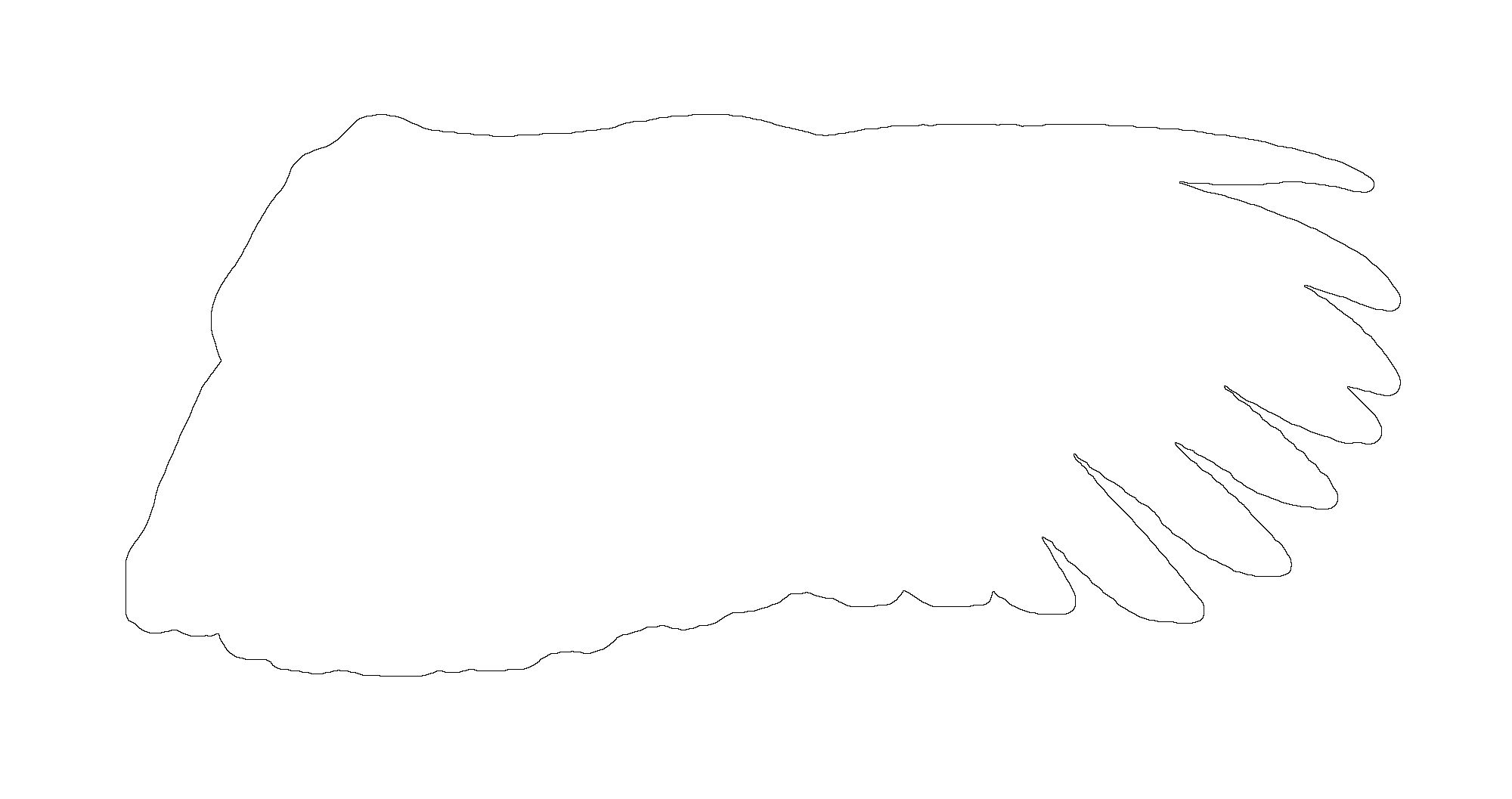

Supplement: Supplementary file 6 — Supplementary Data 4 [file 41467_2026_70692_MOESM6_ESM.zip › Supplementary Data 4/Balearica_regulorum.tif]

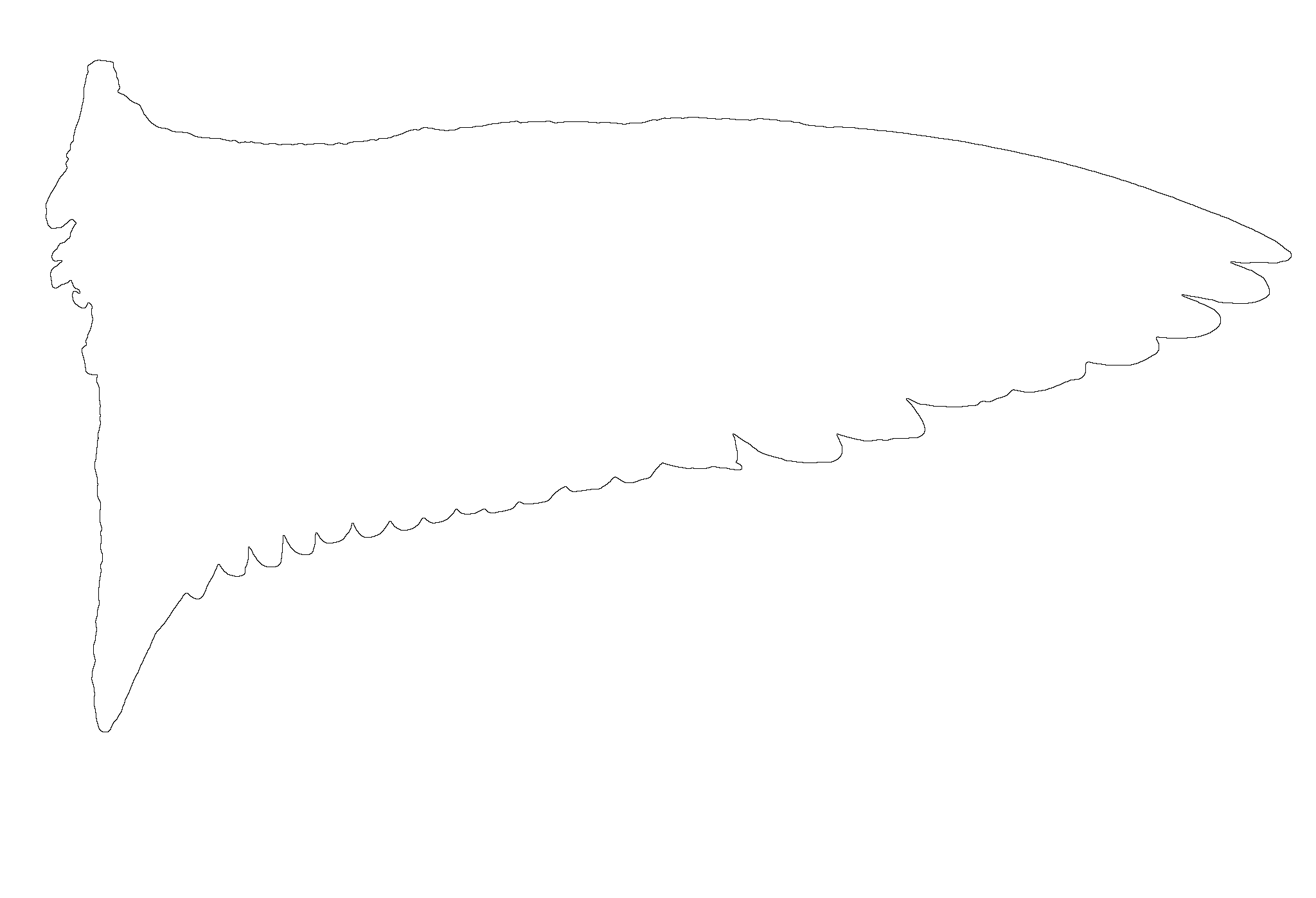

Supplement: Supplementary file 6 — Supplementary Data 4 [file 41467_2026_70692_MOESM6_ESM.zip › Supplementary Data 4/Bartramia_longicauda.tif]

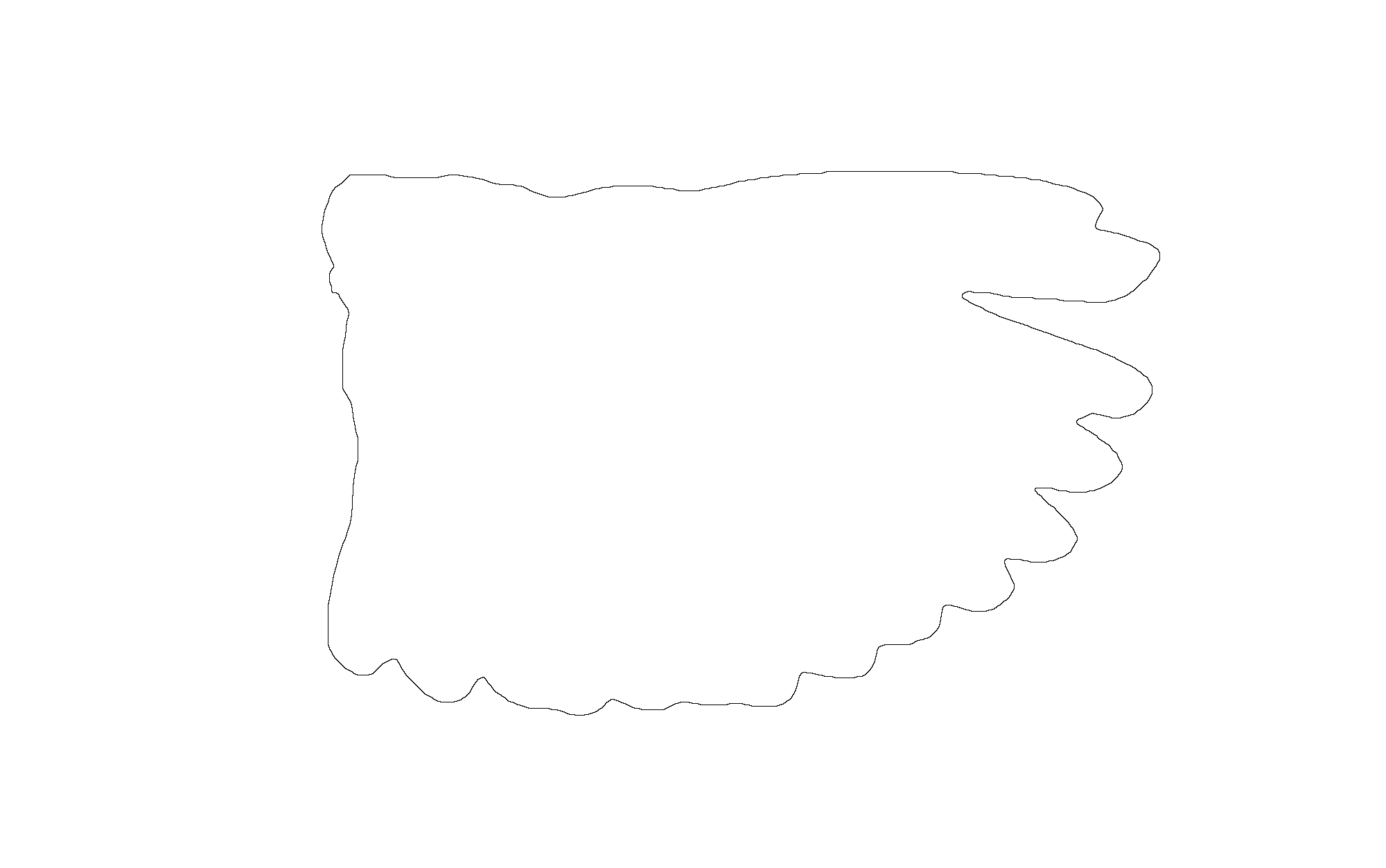

Supplement: Supplementary file 6 — Supplementary Data 4 [file 41467_2026_70692_MOESM6_ESM.zip › Supplementary Data 4/Basileuterus_culicivorus.tif]

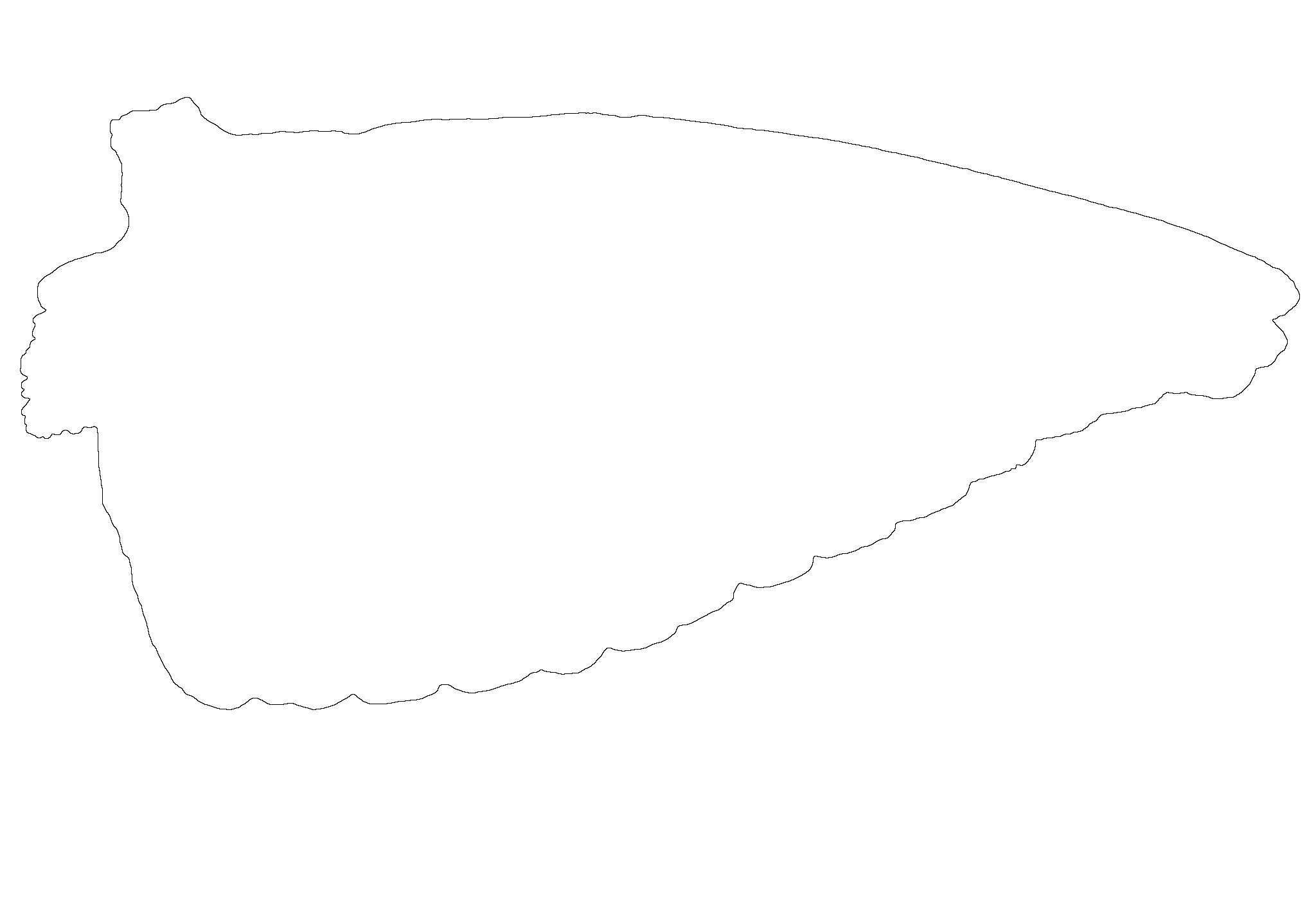

Supplement: Supplementary file 6 — Supplementary Data 4 [file 41467_2026_70692_MOESM6_ESM.zip › Supplementary Data 4/Bombycilla_cedrorum.tif]

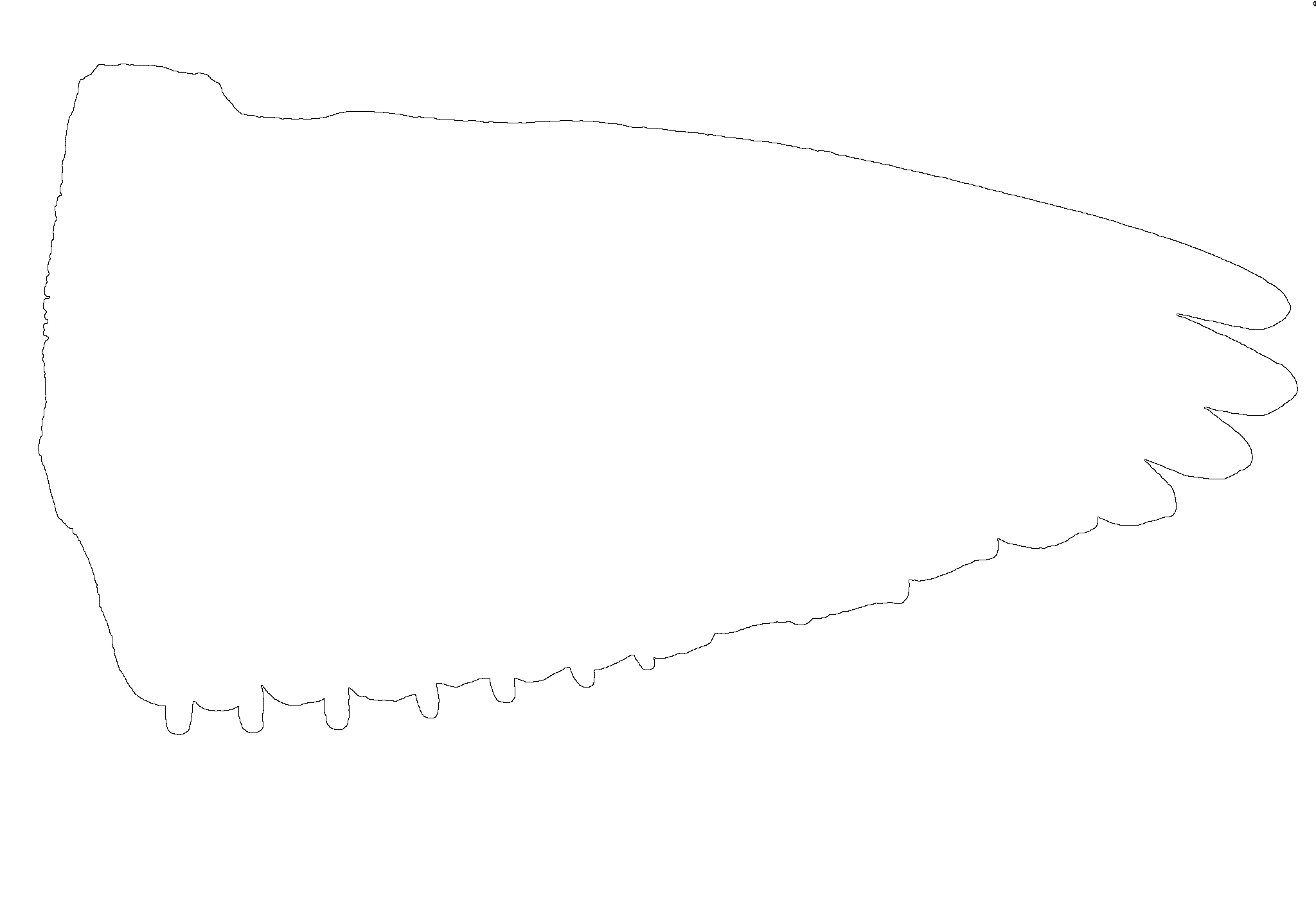

Supplement: Supplementary file 6 — Supplementary Data 4 [file 41467_2026_70692_MOESM6_ESM.zip › Supplementary Data 4/Bombycilla_garrulus.tif]
