# Supplementary material for: Theoretical morphospace reveals mixed optimisation of the avian wing planform for flight style
Source: Nat Commun. 2026 Mar 31;17:3902. doi: 10.1038/s41467-026-70692-w (PMC13128903; doi:10.1038/s41467-026-70692-w)
Supplement: Supplementary file 9 — Reporting Summary [file 41467_2026_70692_MOESM9_ESM.pdf]

nature portfolio

Corresponding author(s): Benton Walters

Last updated by author(s): 18/02/2026

# Reporting Summary

Nature Portfolio wishes to improve the reproducibility of the work that we publish. This form provides structure for consistency and transparency in reporting. For further information on Nature Portfolio policies, see our Editorial Policies and the Editorial Policy Checklist.

## Statistics

For all statistical analyses, confirm that the following items are present in the figure legend, table legend, main text, or Methods section.

| n/a | Confirmed | |
|---|---|---|
| ☐ | ☒ | The exact sample size ($n$) for each experimental group/condition, given as a discrete number and unit of measurement |
| ☐ | ☒ | A statement on whether measurements were taken from distinct samples or whether the same sample was measured repeatedly |
| ☐ | ☒ | The statistical test(s) used AND whether they are one- or two-sided<br>*Only common tests should be described solely by name; describe more complex techniques in the Methods section.* |
| ☐ | ☒ | A description of all covariates tested |
| ☒ | ☐ | A description of any assumptions or corrections, such as tests of normality and adjustment for multiple comparisons |
| ☐ | ☒ | A full description of the statistical parameters including central tendency (e.g. means) or other basic estimates (e.g. regression coefficient) AND variation (e.g. standard deviation) or associated estimates of uncertainty (e.g. confidence intervals) |
| ☒ | ☐ | For null hypothesis testing, the test statistic (e.g. $F$, $t$, $r$) with confidence intervals, effect sizes, degrees of freedom and $P$ value noted<br>*Give P values as exact values whenever suitable.* |
| ☒ | ☐ | For Bayesian analysis, information on the choice of priors and Markov chain Monte Carlo settings |
| ☒ | ☐ | For hierarchical and complex designs, identification of the appropriate level for tests and full reporting of outcomes |
| ☒ | ☐ | Estimates of effect sizes (e.g. Cohen's $d$, Pearson's $r$), indicating how they were calculated |

*Our web collection on statistics for biologists contains articles on many of the points above.*

## Software and code

Policy information about availability of computer code

| | |
|---|---|
| Data collection | No software was used for data collection |
| Data analysis | Image outlines were processed in FIJI (imageJ) version 1.54f, with main workflow completed in MATLAB version R2022b. Phylogenetic analysis was completed in R version 4.4.1 with RStudio version 2024.04.2 and the following packages: ape (version 5.8), geomorph (version 4.0.8), geoscale (version 2.0.1), maps (version 3.4.2), paleotree (version 3.4.5), phangorn (version 2.11.1), phytools (version 2.3-0), rgl (version 1.3.1), RRPP (version 2.0.3), strap (version 1.6-1).<br><br>The base code used in this article is accessible for download from https://github.com/Bristol-Palaeobiology/theofun. ). All executable files and modifications of the base code have been made available in Supplementary Code 1, accessible on Figshare at https://doi.org/10.6084/m9.figshare.31353850. All code files uploaded are freely available without restrictions. |

For manuscripts utilizing custom algorithms or software that are central to the research but not yet described in published literature, software must be made available to editors and reviewers. We strongly encourage code deposition in a community repository (e.g. GitHub). See the Nature Portfolio guidelines for submitting code & software for further information.

## Data

Policy information about availability of data

All manuscripts must include a data availability statement. This statement should provide the following information, where applicable:

- Accession codes, unique identifiers, or web links for publicly available datasets
- A description of any restrictions on data availability
- For clinical datasets or third party data, please ensure that the statement adheres to our policy

All data used in and generated by this study have been deposited and are accessible on Figshare at https://doi.org/10.6084/m9.figshare.31353850. Additionally, the previously unpublished Rayner dataset of birds has been made available as Supplementary Data 5. All data uploaded are freely available without restrictions. The original location and institution of all wing data collected for this study is available in the 'Taxa Set' file, located with Supplementary Data 1.

## Research involving human participants, their data, or biological material

Policy information about studies with human participants or human data. See also policy information about sex, gender (identity/presentation), and sexual orientation and race, ethnicity and racism.

| | |
|---|---|
| Reporting on sex and gender | None |
| Reporting on race, ethnicity, or other socially relevant groupings | NA |
| Population characteristics | NA |
| Recruitment | NA |
| Ethics oversight | NA |

Note that full information on the approval of the study protocol must also be provided in the manuscript.

# Field-specific reporting

Please select the one below that is the best fit for your research. If you are not sure, read the appropriate sections before making your selection.

☒ Life sciences ☐ Behavioural & social sciences ☐ Ecological, evolutionary & environmental sciences

For a reference copy of the document with all sections, see nature.com/documents/nr-reporting-summary-flat.pdf

# Life sciences study design

All studies must disclose on these points even when the disclosure is negative.

| | |
|---|---|
| Sample size | A 10 percent sample of extant diversity was chosen, covering as many extant bird orders as possible. The main dataset is modeled after that of Rayner 1988, with additional taxa added to improve the spread of diversity in birds. This sample size was determined as sufficient as it exceeds the amount necessary to reach a theoretical morpospace where further additions did not change theoretical shape generation. |
| Data exclusions | No data was excluded by this analysis. Control for covariates was not relevant as the tested elements of the analysis are theoretical representations of shape and are not directly linked to biological elements. |
| Replication | As all experiments in this analysis are computational tests were conducted multiple times to ensure consistent results. |
| Randomization | this study did not contain a random element. All samples studied come from established museum collections. |
| Blinding | Blinding was not relevant as the method involves the creation of theoretical shapes via an automated pathway, thus removing specific user input. |

# Reporting for specific materials, systems and methods

We require information from authors about some types of materials, experimental systems and methods used in many studies. Here, indicate whether each material, system or method listed is relevant to your study. If you are not sure if a list item applies to your research, read the appropriate section before selecting a response.

## Materials & experimental systems

| n/a | Involved in the study |
|---|---|
| ☒ | ☐ Antibodies |
| ☒ | ☐ Eukaryotic cell lines |
| ☒ | ☐ Palaeontology and archaeology |
| ☒ | ☐ Animals and other organisms |
| ☒ | ☐ Clinical data |
| ☒ | ☐ Dual use research of concern |
| ☒ | ☐ Plants |

## Methods

| n/a | Involved in the study |
|---|---|
| ☒ | ☐ ChIP-seq |
| ☒ | ☐ Flow cytometry |
| ☒ | ☐ MRI-based neuroimaging |

## Plants

| | |
|---|---|
| Seed stocks | NA |
| Novel plant genotypes | NA |
| Authentication | NA |
